# Activation of *gem*-Dichloroacetamides and Epoxides Using Elemental Sulfur and Amines: A Route to Monothiooxalamides and α‑Ketothioamides

**DOI:** 10.1021/acs.joc.5c01684

**Published:** 2025-10-02

**Authors:** Alageswaran Jayaram, Yu- Ming Liu, Nian-Qi Chen, Genin Gary Huang, Gopal Chandru Senadi, Wei-Yu Lin

**Affiliations:** a Department of Medicinal and Applied Chemistry, 38023 Kaohsiung Medical University, Kaohsiung 80708, Taiwan; b Department of Chemistry, Faculty of Engineering and Technology, 93104 SRM Institute of Science and Technology, Kattankulathur, Tamil Nadu 603203, India; c Department of Medical Research, Kaohsiung Medical University Hospital, Kaohsiung 80708, Taiwan; d Drug Development and Value Creation Research Centre, 38023 Kaohsiung Medical University, Kaohsiung 80708, Taiwan

## Abstract

The selective activation of C–X bonds to generate value-added products via transition metal-free methodologies remains a formidable challenge in modern synthetic chemistry. Herein, we report a metal-free didechlorinative strategy for the construction of unsymmetrical monothiooxalamides through C–S and C–N bond formation. This transformation proceeds via a one-pot functionalization of *gem*-dichloroacetamides with various amines and elemental sulfur (S_8_) under ambient conditions in an open-air atmosphere, offering a sustainable and operationally simple approach. Additionally, a regioselective epoxide ring-opening approach was implemented using I_2_/DMSO, enabling the efficient synthesis of α-ketothioamides through the incorporation of S_8_ and diverse amine nucleophiles. Furthermore, ligand studies revealed that monothiooxalamides exhibit high efficacy as ligands in Cu-catalyzed C–N cross-coupling reactions. The method’s synthetic utility was further demonstrated through gram-scale synthesis and the preparation of natural product derivatives and drug analogues, highlighting its potential for industrial applications.

## Graphical abstract

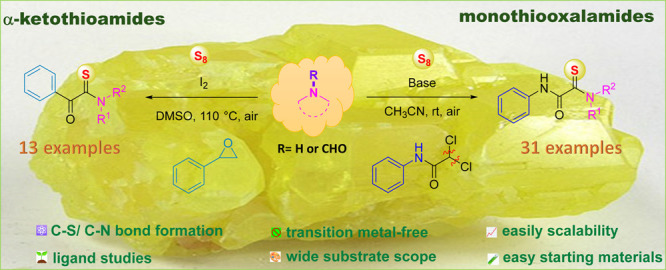

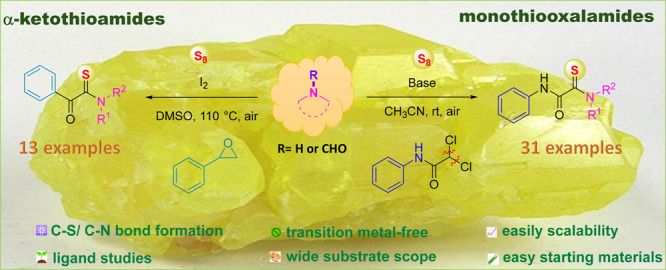

## Introduction

Geminal dihalo compounds exhibit distinctive reactivity in C–X (X= F, Br, and Cl) bond activation, primarily due to the strong electron-withdrawing nature of halogen atoms, which facilitates nucleophilic attack at the geminal carbon.^1–3^ This intrinsic property enables *gem*-dichloroacetamides to serve as versatile precursors for a wide range of organic transformations, involving the cleavage of both C–Cl bonds and the formation of diverse functional linkages such as C–S, C–N, and C–O bonds.^4,5^ However, carbon–chlorine bond activation presents a significant challenge compared to C–I and C–Br bonds, as the C–Cl bond possesses a high bond dissociation energy (∼330 kJ/mol), making its selective transformation more demanding.^6^ Unraveling new synthetic strategies for these functional groups could unlock valuable pathways for chemical transformations.

Over the past few decades, sulfur-containing compounds have garnered considerable attention in organic synthesis, owing to their broad spectrum of biological activities, ready availability, low toxicity, operational simplicity, and chemical stability to undergo a wide range of fascinating and diverse transformations (Scheme 1i).^7,8^ Monothiooxalamides constitute a pivotal class of sulfur-containing compounds that exhibit a broad spectrum of pharmacological activities, including potent cytotoxicity against human cancer cell lines, anti-inflammatory properties, and significant utility in asymmetric metallocatalysis.^8^ Despite their considerable applicability in medicinal applications and high potentials in various fields, their exploration remains largely underdeveloped, primarily due to the absence of efficient and generalizable synthetic methodologies. To date, only a limited number of retrosynthetic strategies have been documented for the construction of unsymmetrical monothiooxalamides (Scheme 1ii).^9^ In 2022, Ma et al. delineated a strategy for the synthesis of monothiooxalamides utilizing a bromodifluoro reagent, amines, and elemental sulfur (Scheme 1iii).^10^ However, this protocol necessitated stringent reaction conditions, including high temperatures (120 °C) and an inert atmosphere, and faced difficulties in the preparation of starting materials. More recently, our research group established a metal-free protocol for the synthesis of unsymmetrical oxalamides via triple CCl_2_Br bond cleavage employing *gem*-dichloroacetamides and amines under mild conditions.^5^ In continuation of our ongoing endeavors in C–X bond functionalization, we herein disclose a novel, metal-free synthetic strategy for the construction of unsymmetrical monothiooxalamides via C–S/C–N bond formation. This protocol leverages a one-pot, didechlorinative functionalization of *gem*-dichloroacetamides with varying amines and elemental sulfur as a sulfur source, proceeding at ambient temperature under an open-air atmosphere (Scheme 1v).

**Scheme 1:**
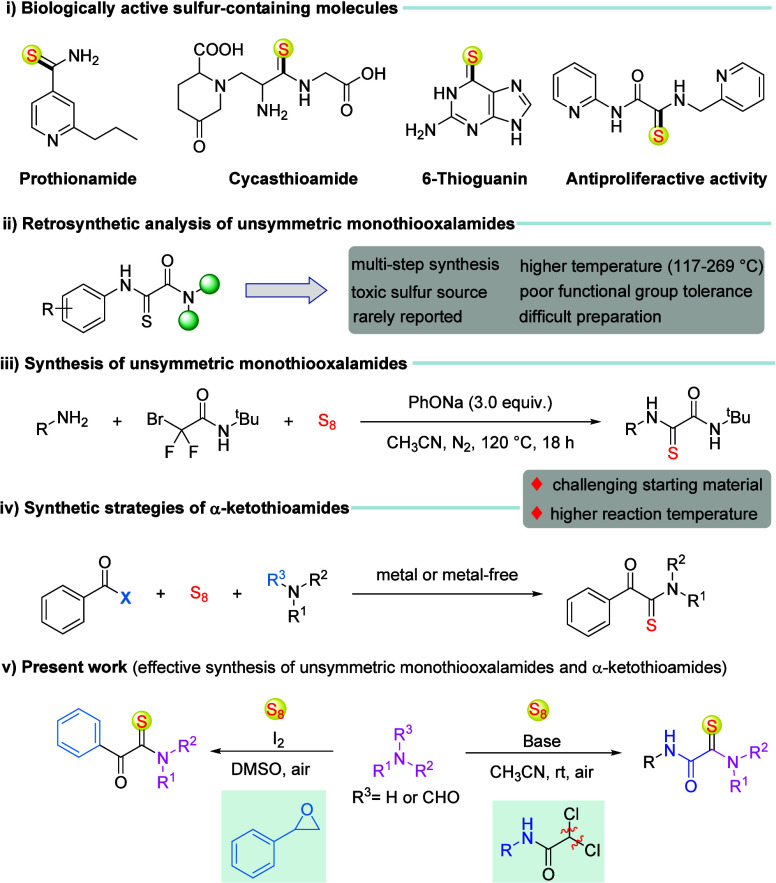
(i–v) Background Information and Innovation of Present Work

In addition, ketothioamides are pivotal synthetic intermediates with broad applications in medicinal chemistry and materials science.^11,12^ The transformation of amines and sulfur into α-ketothioamides via various acyl-functionalized precursors has been extensively investigated under both metal-catalyzed and metal-free conditions. Several synthetic strategies have been developed, utilizing precursors such as alkynes,^13^ enaminones,^14^ azido ketones,^15^ sulfoxonium ylides,^16^ arylglyoxal hydrates,^17^ and methyl ketones^18^ in conjunction with amines and elemental sulfur (S_8_) (Scheme 1iv). Despite these advancements, existing methodologies often suffer from inherent drawbacks, including reliance on transition metal catalysts, multistep procedures, and harsh reaction conditions. Consequently, the development of a streamlined, transition metal-free, toxic reagent- or additive-free, and highly efficient strategy for α-ketothioamide synthesis remains a compelling objective in modern organic synthesis. In this study, we introduce a rapid and versatile approach for the synthesis of biologically relevant α-ketothioamides via the regioselective ring-opening of epoxides (Scheme 1v). This transformation, facilitated by molecular iodine (I_2_), enables the concurrent formation of C–S and C–N bonds in a single-step process. To the best of our knowledge, there have been no reported instances of using epoxides as acyl precursors for α-ketothioamide synthesis, providing a novel and chemically diverse platform for the construction of these valuable compounds.

## Results and Discussion

To establish optimal reaction conditions for the synthesis of *N*-phenyl-2-(pyrrolidin-1-yl)-2-thioxoacetamide (**3a**), a model reaction between 2,2-dichloro-*N*-phenylacetamide (**1a**) and pyrrolidine (**2a**) was investigated in the presence of elemental sulfur (S_8_) under open-air conditions (Table 1).

**Table 1:**
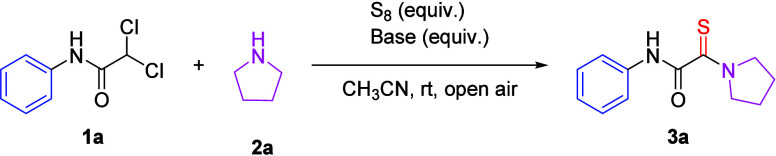
Optimization for the *N*-Phenyl-2-(pyrrolidin-1-yl)-2-thioxoacetamide Derivative^a^ ^–^ ^d^

**entry**	**base (equiv)**	**S**_**8**_ **(equiv)**	**solvent**	**yield (%)** ^b^
1	LiOH	0.5	CH_3_CN	92
2	NaOH	0.5	CH_3_CN	66
3	K_3_PO_4_	0.5	CH_3_CN	97
4	Cs_2_CO_3_	0.5	CH_3_CN	98 (94)^c^
5	Et_3_N	0.5	CH_3_CN	94
6		0.5	CH_3_CN	38
7	Cs_2_CO_3_	0.5	H_2_O	28
8	Cs_2_CO_3_	0.5	MeOH	44
9	Cs_2_CO_3_	0.5	2-Me-THF	92
10	Cs_2_CO_3_	0.5	DMSO	97
11	Cs_2_CO_3_	0.5	cyrene	53
12	Cs_2_CO_3_	0.5	HFIP	NR
13	Cs_2_CO_3_	0.3	CH_3_CN	89
14	Cs_2_CO_3_	0.4	CH_3_CN	93
15	Cs_2_CO_3_	0.5	CH_3_CN	97^d^
16	Cs_2_CO_3_	0.5	CH_3_CN	45^e^
17	Cs_2_CO_3_	0.5	CH_3_CN	16^f^

a The reactions were performed with **1a** (0.1 mmol), **2a** (2.0 equiv), S_8_ (0.5 equiv), base (2.0 equiv), and solvent (1 mL) at room temperature under open air for 12 h.

b Yields were determined by ^1^H NMR spectroscopy using 1,3,5- trimethoxybenzene as the internal standard.

c Isolated yield.

d 80 °C.

e P_4_S_10_ instead of S_8_.

f Na_2_S instead of S_8_, NR = no reaction, and cyrene = dihydrolevoclucosenone.

Various inorganic and organic bases were initially evaluated in CH_3_CN at room temperature. Among them, Cs_2_CO_3_ emerged as the most efficient base, affording the desired product in a 94% isolated yield (entry 4). In contrast, alternative bases such as LiOH, NaOH, K_3_PO_4_, and Et_3_N gave comparatively lower yields ranging from 66 to 97% (entries 1–3 and 5). Notably, performing the reaction in the absence of a base resulted in a substantial decrease in the yield (entry 6). A solvent screening study further confirmed CH_3_CN as the most suitable reaction medium. When other solvents such as H_2_O, MeOH, 2-Me-THF, DMSO, cyrene, and HFIP were employed, the yields dropped significantly or the reaction failed to proceed (entries 7–12). Additionally, the effect of sulfur loading was examined, revealing that 0.5 equiv of elemental sulfur gave superior results compared to 0.3 and 0.4 equiv (entries 13–14). Finally, conducting the reaction at an elevated temperature (80 °C) did not lead to a notable improvement in the yield (entry 15), indicating that the reaction proceeds efficiently at ambient temperature without the need for thermal activation. To further assess the role of the sulfur source, we examined the reaction under standard conditions using P_4_S_1_0 and Na_2_S as alternatives to elemental sulfur (S_8_). As shown in Table 1, P_4_S_1_0 afforded the desired product in a moderate yield (entry 16), while Na_2_S gave only a low yield (entry 17).

With the optimized reaction conditions established, the substrate scope of this didechlorinative thioamidation protocol was systematically explored by reacting a series of substituted *gem*-dichloroacetamides (**1**) with pyrrolidine (**2a**) and elemental sulfur (S_8_) (Table 1). Beyond **1a**, substrates **1b**–**1i**, featuring electron-donating (−Me, −Et, and −OMe) and electron-withdrawing (−F, −Cl, −Br, −NO_2_, and –OH) substituents at the para position of the benzene ring, exhibited excellent reactivity under the optimized conditions, affording the corresponding monothiooxalamides (**3b**–**3i**) in good to excellent yields (78–92%). Gratifyingly, substrates bearing −I, −CF_3_, and –NO_2_ groups at the C2 and C3 positions (**3j**–**3l**) participated efficiently in the transformation, delivering the target products in 69–93% yields. Furthermore, substrates featuring multiple substituents on the aryl core were examined to assess their reactivity under the optimized conditions. The disubstituted derivative (**1m**) underwent *gem*-dichloro bond cleavage with high efficiency, affording the target monothiooxalamide (**3m**) in a 71% yield. Additionally, the benzyl-functionalized substrate (**1m**) exhibited excellent compatibility with the transformation, furnishing the corresponding thiooxalamide (**3n**) in a good yield. Notably, *N*-heteroaromatic cyclic frameworks, such as benzothiazole derivatives, were well-tolerated under these conditions, as demonstrated by the successful formation of **3o**. However, substrates with bulky structures (**1p**–**1q**) were unsuitable for this transformation and no desired product was obtained. This study encompassed both linear and cyclic secondary amines, which efficiently participated in the transformation, affording the corresponding thiooxalamide derivatives (**3r**–**3u**) in good to excellent yields (71–99%). Notably, primary aliphatic and aromatic amines also demonstrated high efficacy as substrates in this *gem*-dichloro bond cleavage reaction, furnishing the desired products (**3v**–**3aa**) with moderate to good yields (41–76%) under the optimized conditions.

To further broaden the applicability of this transformation, we extended our investigation by utilizing *gem*-dichloro phenylacetamide (**1**) in conjunction with *N*,*N*-dimethylformamide (**6a**) and *N*,*N*-diethylformamide (**6b**) as representative substrates, replacing amines under basic reaction conditions. This strategic modification successfully facilitated the synthesis of the desired monothiooxalamide derivatives (**3r**, **3ab**, **3ac**, and **3s**) in moderate to good yields (63–85%), thereby demonstrating the adaptability of this transformation to alternative nucleophilic systems.

The development of metal-free strategies for C–S and C–N bond formation via epoxide ring-opening reactions remains a formidable challenge in synthetic organic chemistry. In pursuit of advancing this area, we continued our investigation into iodine-mediated thioamidation, employing amines and elemental sulfur (S_8_) as a sulfur source to establish an efficient and practical approach for the synthesis of α-ketothioamide derivatives. Our study commenced with the selection of styrene oxide (**4a**) as a model substrate to optimize the reaction parameters (Table S1, see the SI for details). Gratifyingly, the desired ketoamide product (**5f**) was obtained in an 83% yield when the reaction was conducted under open-air conditions using I_2_ (50 mol %) and S_8_ (1.5 equiv) in DMSO at 110 °C for 2 h (Table S1, entry 7, SI). The difference in sulfur equivalents reflects the differing reactivities of the electrophilic partners. In Table 2, *gem*-dichloroacetamides contain activated methylene groups with good leaving groups (Cl), enabling efficient transformation with 0.5 equiv of sulfur. In contrast, the strained but less activated epoxides in Table 3 required 1.5 equiv of sulfur to achieve comparable reactivity and conversion. With the optimized reaction conditions in hand, we proceeded to evaluate the scope and influence of various amines on the transformation, leading to the synthesis of the anticipated α-ketothioamide derivatives (**5a**–**5k**) in Table 3. Notably, both linear and cyclic secondary amines exhibited excellent compatibility, affording the corresponding products (**5a**–**5f**) in good to excellent yields (71–92%). Furthermore, primary amines also participated efficiently in the transformation, yielding the desired α-ketothioamide derivatives (**5g**–**5j**) in moderate to good yields (51–78%). Interestingly, when the reaction was conducted with piperazine (**2k**), a disubstituted product, 2,2′-(piperazine-1,4-diyl)­bis­(1-phenyl-2-thioxoethan-1-one) (**5k**), was obtained in a 39% yield, highlighting the potential for bis-thioamidation under these conditions. In contrast, the use of an aromatic amine led to significantly diminished reactivity, resulting in a poor yield, thus indicating the limited compatibility of electron-rich aryl amines in this protocol. Gratifyingly, epoxide substrates bearing electron-donating (−OMe) and electron-withdrawing (−Cl) substituents on the phenyl ring underwent the reaction smoothly under the standard conditions, affording the desired products **5l** and **5m** in good yields of 76 and 81%, respectively.

**Table 2:**
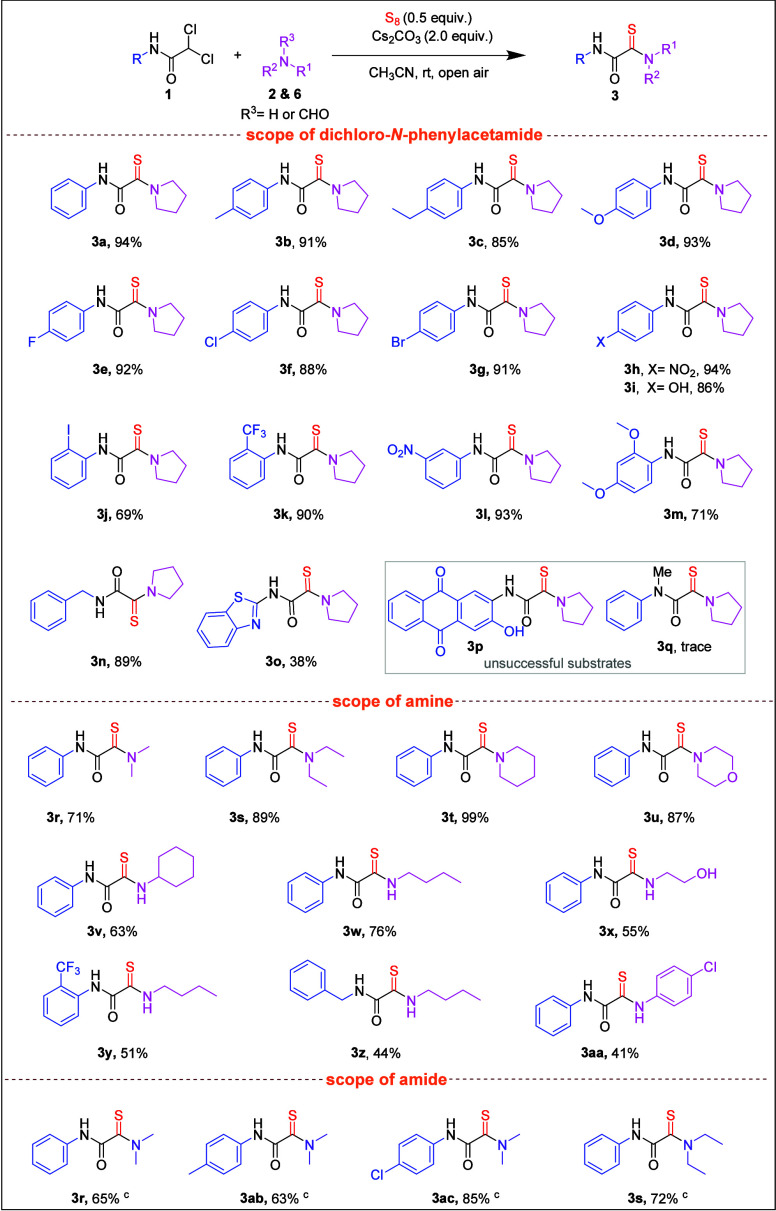
Substrate Scope for Monothiooxalamide Derivatives^a^ ^,^ ^b^ ^,^ ^c^

a Reaction conditions: **1** (0.2 mmol), **2** (2.0 equiv), S_8_ (0.5 equiv), Cs_2_CO_3_ (2.0 equiv), CH_3_CN (2 mL), at room temperature under open air for 12 h.

b Isolated yield.

c Reaction conditions: **1** (0.2 mmol), **6** (2.0 mL), S_8_ (0.5 equiv), NaOH (5.0 equiv), at room temperature under open air for 5 h.

**Table 3:**
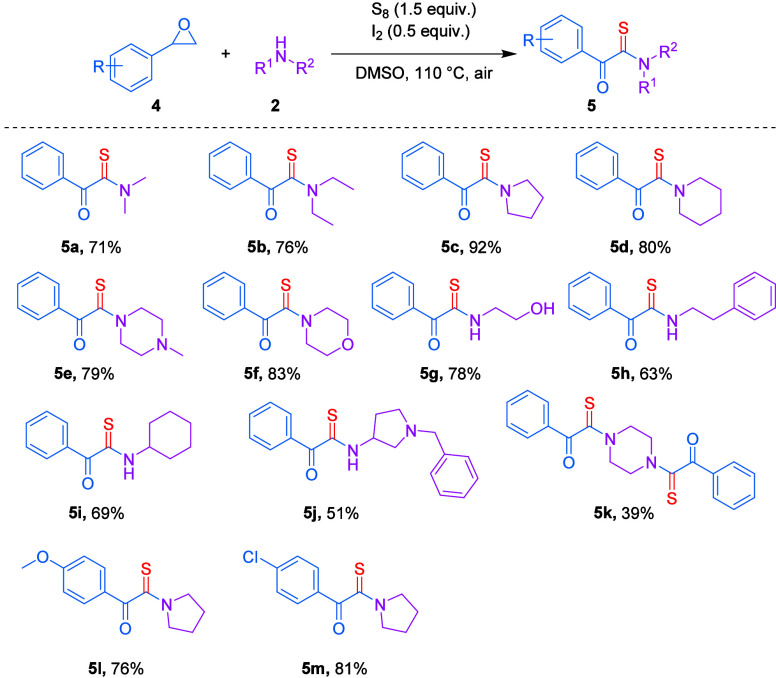
Substrate Scope for α-Ketothioamide Derivatives^a^ ^,^ ^b^

a Reaction conditions: **4** (0.2 mmol scale), **2** (2.0 equiv), S_8_ (1.5 equiv), I_2_ (0.5 equiv) at 110 °C temperature under open air for 2–4 h.

b Isolated yield.

Subsequently, the scalability of the transformation was assessed to validate its practicality for larger-scale synthesis. Gratifyingly, desired products **3a** and **5f** were obtained in good yields of 85 and 73%, respectively, demonstrating the robustness of the protocol (Scheme 2a). Moreover, the synthetic utility of this methodology was further exemplified by the successful construction of a natural product derivative (**3ad**) and a drug analogue (**3ae**), which were isolated in 42 and 49% yields, respectively, thereby underscoring the potential applicability of this approach in medicinal and natural product chemistry (Scheme 2b).

**Scheme 2:**
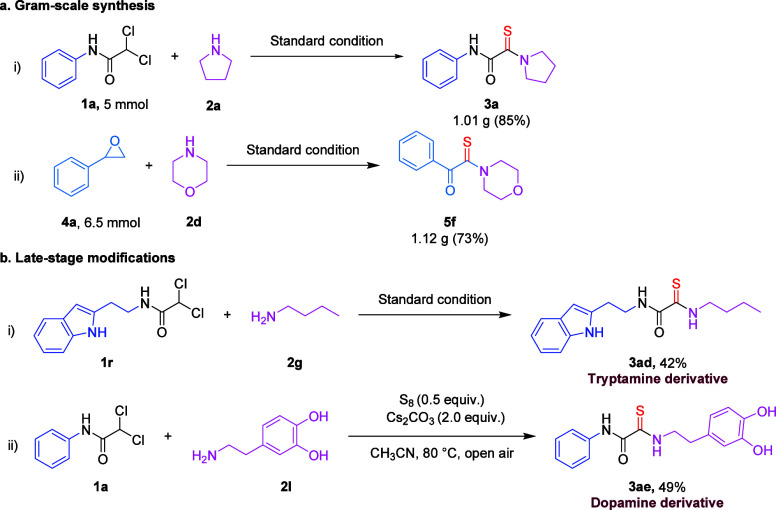
(a, b) Synthetic Applications

To evaluate the ligand efficiency of unsymmetrical monothiooxalamide cross-coupling reactions, we initiated our investigation by employing benzamide (**7**) and 1-iodo-4-methoxybenzene (**8**) as model substrates for C–N bond formation (Scheme 3).^10^ The reaction was systematically examined using copper catalysts, a base, and a diverse set of thiooxalamide derivatives as potential ligands, including **3ab** (**L1**), **3w** (**L2**), **3a** (**L3**), **3y** (**L4**), **3v** (**L5**), **3z** (**L6**), **3x** (**L7**), **3m** (**L8**), and **5i** (**L9**), which are developed in Table 2, alongside conventional oxalamide derivatives (**10** and **11**) and diamine and phosphine ligands for comparative analysis. Among the tested ligands, **3z** (**L6**) exhibited the highest ligand efficiency, significantly facilitating the C–N cross-coupling reaction (Scheme 3). This observation highlights the strong coordinating ability of monothiooxalamides, which likely stabilizes the copper species and enhances the catalytic turnover. The presence of the thiocarbonyl (−C=S) and amide (−C=O–NH) functionalities may contribute to efficient metal coordination, thereby modulating the electronic environment of the active catalytic species. These findings demonstrate that monothiooxalamides represent a promising class of ligands for Cu-catalyzed cross-coupling reactions.

**Scheme 3:**
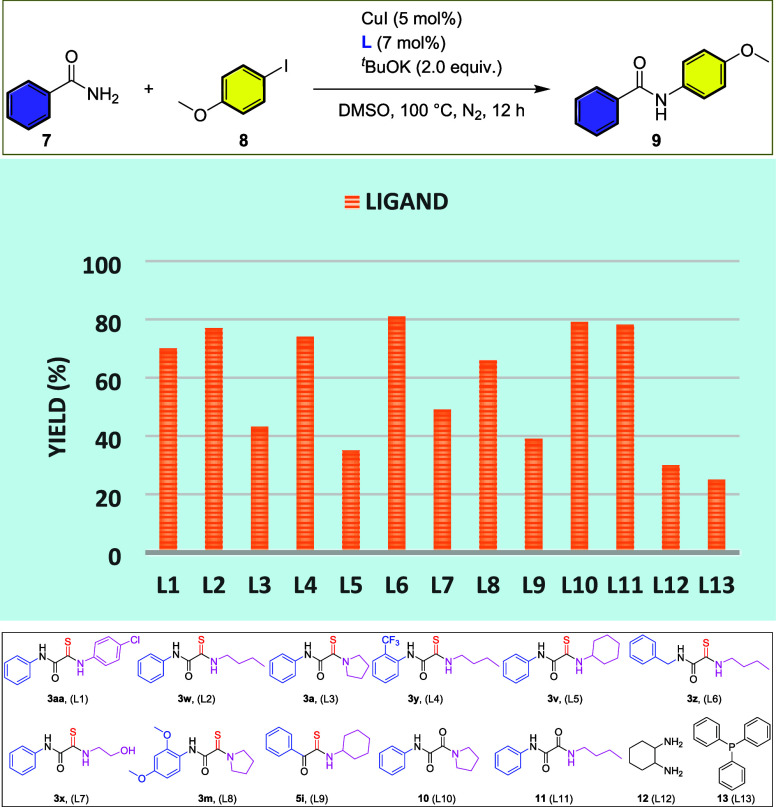
Ligand Study of Unsymmetrical Monothiooxalamides

To gain insight into the plausible reaction mechanism, a series of control experiments were conducted. When 2,2,6,6-tetramethylpiperidinyloxy (TEMPO) and 1,1-diphenylethylene (DPE) were introduced into the reaction mixture alongside **1a** and **4a** under the optimized conditions, the expected monothiooxalamide (**3a**) (Scheme 4a) and α-ketothioamide (**5f**) (Scheme 4b) were obtained smoothly. These observations strongly suggest that the transformation proceeds via a nonradical pathway.

**Scheme 4:**
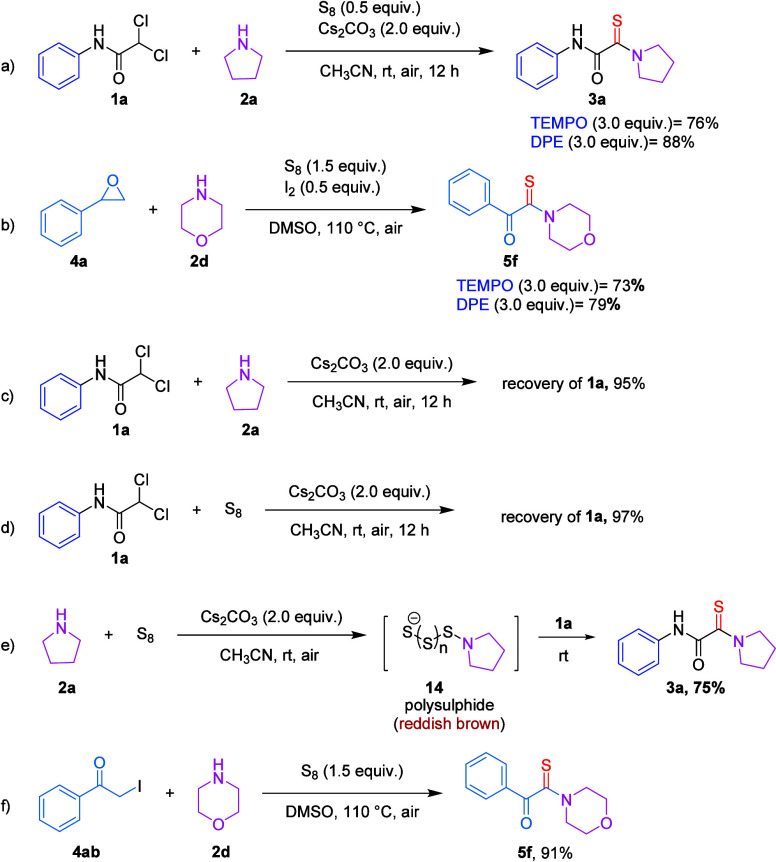
(a–f) Control Experiments

As expected, when the standard reaction was performed in the absence of either elemental sulfur (S_8_) or amine **2**, no conversion of *gem*-dichloroacetamide **1a** was observed, confirming that both components are essential for the transformation (Scheme 4c,d). Interestingly, when amine **2** was stirred with S_8_ in the presence of base, a reddish-brown coloration developed, consistent with the formation of polysulfide intermediates. Upon addition of **1a** to this preformed mixtured polysulfide, the reaction proceeded smoothly to afford the desired monothiooxalamide product **3a** in 75% yield (Scheme 4e).^19^ This result strongly suggests that the amine reacts with elemental sulfur first to generate a nucleophilic aminopolysulfide, which subsequently engages with **1a**, supporting an S_8_-first activation pathway. Furthermore, **4ab** was found to undergo efficient conversion with morpholine (**2d**), yielding the desired α-ketothioamide (**5f**) in 91% yield (Scheme 4f). This outcome indicates that **4ab** may serve as a key intermediate in the reaction mechanism, thereby providing valuable mechanistic insights into the underlying transformation.

Based on our control experiment results and the literature,^13,16,20^ we propose a plausible reaction mechanism for this transformation (Scheme 5). The transformation is initiated by the nucleophilic activation of elemental sulfur (S_8_) by amine **2**, generating a reactive polysulfide intermediate (**14**).^21^ This sulfur-rich species exhibits enhanced nucleophilicity and readily undergoes nucleophilic substitution with **1a**, leading to the formation of intermediate **I** via the elimination of HCl. Subsequently, a second molecule of amine **2** attacks intermediate **I** to afford intermediate **II**,^22^ which then undergoes elimination of the polysulfide, delivering the target monothiooxalamide product **3**. In other hand, styrene oxide (**4**) reacts within the I_2_/DMSO system, leading to the formation of intermediate **4ab**.^23^ This intermediate undergoes nucleophilic addition with an amine (**2**), yielding intermediate **III**, which subsequently tautomerizes to its keto enol form (**IV**). The resulting intermediate undergoes electrophilic addition of S_8_, forming intermediate **V**, which upon desulfhydrylation delivers the targeted α-ketothioamide product (**5**).

**Scheme 5:**
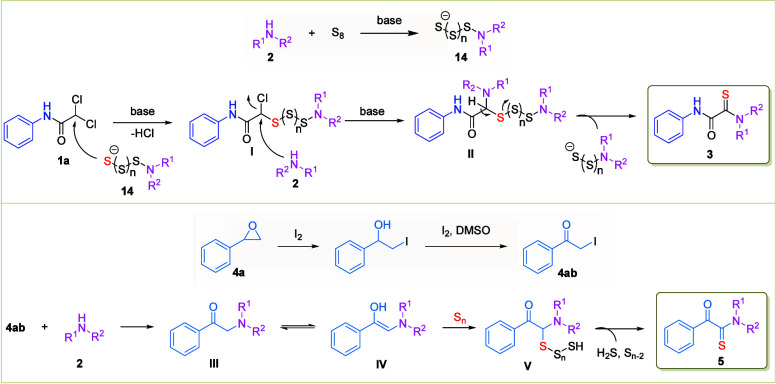
Plausible Reaction Mechanism

## Conclusions

In summary, we have developed an efficient, novel metal-free strategy for the didechlorinative functionalization of *gem*-dichloroacetamides, enabling the synthesis of unsymmetrical monothiooxalamides through C–S and C–N bond formation. This transformation proceeds under mild conditions in an open-air atmosphere utilizing elemental sulfur (S_8_) as a sustainable sulfur source. The methodology exhibits a broad substrate scope, accommodating a diverse range of amines, and delivers the desired products in high yields. Moreover, ligand studies demonstrated that monothiooxalamides serve as effective ligands in Cu-catalyzed C–N cross-coupling reactions, further expanding their synthetic utility. Additionally, an iodine-mediated epoxide ring-opening strategy was employed for the regioselective synthesis of α-ketothioamides, providing a novel pathway for their construction. Overall, this work provides a sustainable, operationally simple, and transition metal-free approach for accessing structurally diverse sulfur-functionalized compounds, reinforcing its significance in organic synthesis, medicinal chemistry, and catalysis. In addition, metal-free C–X bond activation developments are underway in our laboratory.

## Experimental Section

### General Procedure for 2-(Pyrrolidin-1-yl)-2-thioxo Derivatives (**3a**–**3o**) and Amine Derivatives (**3r**–**3aa**)

A 15 mL reaction tube was charged with (**1a**–**1o)** (0.2 mmol, 1.0 equiv), S_8_ (0.1 mmol, 0.5 equiv), CS_2_CO_3_ (0.4 mmol, 2.0 equiv), and amine (**2**) (0.4 mmol, 2.0 equiv), with CH_3_CN (2 mL). The resulting mixture was stirred at room temperature in an open-air atmosphere for about 12 h. After the completion of the reaction, the reaction mixture was diluted with 5 mL of water. The aqueous layer was extracted with ethyl acetate (3 × 10 mL), and the combined organic layer was washed with brine solution (1 × 5 mL). The final organic layer was then dried over MgSO_4_ and concentrated under reduced pressure to get the crude product. The obtained crude product was purified using column chromatography by eluting with ethyl acetate/hexane (3:7) to afford pure 2-(pyrrolidin-1-yl)-2-thioxo derivatives (**3a**–**3o**) up to 38–94% yields and pure amine derivatives (**3r**–**3aa**) up to 41–99% yields.

### General Procedure for 2-Amino-1-phenyl-2-thioxoethan-1-one Derivatives (**5a**–**5m**)

A 15 mL reaction tube was charged with aryl oxiranes (**4**) (0.2 mmol, 1 equiv), iodine (0.1 mmol, 0.5 equiv), and dimethyl sulfoxide (2 mL), and the resulting mixture was stirred at 110 °C; the reaction tube was removed after about 1 h. Then, additional **2** (0.4 mmol, 2.0 equiv) and S_8_ (0.4 mmol, 2.0 equiv) were added at room temperature. The reaction mixture was stirred at 110 °C in an oil bath for about 2–4 h; then, it was allowed to reach room temperature and quenched with a saturated solution of Na_2_S_2_O_3_. After being diluted with 5 mL of water, the aqueous layer was extracted with ethyl acetate (3 × 10 mL), and the combined ethyl acetate layer was washed with a brine solution (1 × 5 mL). The final ethyl acetate layer was then dried over MgSO_4_ and concentrated under reduced pressure to get the crude product. The obtained crude product was purified using column chromatography by eluting with ethyl acetate/hexane (3:7) to afford pure 2-amino-1-phenyl-2-thioxoethan-1-one derivatives **5a**–**5m** up to 39–92% yields.

### General Procedure for Amide Derivatives (**3r**, **3ab**, **3ac**, and **3s**)

A 15 mL reaction tube was charged with (**1a**, **1b**, and **1f**) (0.2 mmol, 1.0 equiv), S_8_ (0.1 mmol, 0.5 equiv), NaOH (1.0 mmol, 5.0 equiv), and amide **6** (2.0 mL). The resulting mixture was stirred at room temperature in an open-air atmosphere for about 5 h. After the completion of the reaction, the reaction mixture was diluted with 5 mL of water. The aqueous layer was extracted with ethyl acetate (3 × 10 mL), and the combined organic layer was washed with brine solution (1 × 5 mL). The final organic layer was then dried over MgSO_4_ and concentrated under reduced pressure to get the crude product. The obtained crude product was purified using column chromatography by eluting with ethyl acetate/hexane (3:7) to afford pure derivatives (**3r**, **3ab**, **3ac**, and **3s**) up to 63–85% yields.

#### *N*-Phenyl-2-(pyrrolidin-1-yl)-2-thioxoacetamide (**3a**)

The title compound was synthesized according to a general procedure. The crude mixture was purified using column chromatography by eluting with ethyl acetate/hexane (3:7) and obtained as a pale-yellow solid (44.0 mg, 94%); mp. 136–138 °C; ^1^H NMR (400 MHz, DMSO-*d*_6_): δ 10.43 (bs, 1H), 7.60 (d, *J* = 8.04 Hz, 2H), 7.30 (t, *J* = 8.16 Hz, 2H), 7.07 (t, *J* = 7.44 Hz, 1H), 3.64 (d, *J* = 7.04 Hz, 4H), 1.96 (d, *J* = 2.36 Hz, 4H); ^13^C­{^1^H} NMR (100 MHz, CDCl_3_): δ 186.0, 160.0, 137.2, 129.1, 125.0, 119.9, 55.8, 54.1, 27.0, 23.6. HRMS (HR-EI) *m*/*z*: [M^+^] calcd for C_12_H_14_N_2_OS, 234.0827; found, 234.0827.

#### 2-(Pyrrolidin-1-yl)-2-thioxo-*N*-(*p*-tolyl)­acetamide (**3b**)

The title compound was synthesized according to the general procedure. The crude mixture was purified using column chromatography by eluting with ethyl acetate/hexane (3:7) and obtained as a yellow solid (45.2 mg, 91%); mp 139–141 °C; ^1^H NMR (400 MHz, CDCl_3_): δ 9.49 (bs, 1H), 7.47 (d, *J* = 8.48 Hz, 2H), 7.14 (d, *J* = 8.16 Hz, 2H), 4.12 (t, *J* = 6.72 Hz, 2H), 3.87 (t, *J* = 7.08 Hz, 2H), 2.32 (s, 3H), 2.09–1.97 (m, 4H); ^13^C­{^1^H} NMR (100 MHz, CDCl_3_): δ 186.1, 159.9, 134.7, 134.6, 129.6, 119.9, 55.8, 54.1, 27.0, 23.6, 21.0. HRMS (HR-ESI) *m*/*z*: [M + Na]^+^ calcd for C_13_H_16_N_2_OSNa, 271.0881; found, 271.0868.

#### *N*-(4-Ethylphenyl)-2-(pyrrolidin-1-yl)-2-thioxoacetamide (**3c**)

The title compound was synthesized according to the general procedure. The crude mixture was purified using column chromatography by eluting with ethyl acetate/hexane (3:7) and obtained as a yellow solid (44.6 mg, 85%); mp 119–121 °C; ^1^H NMR (400 MHz, DMSO-*d*_6_): δ 10.3 (bs, 1H), 7.49 (d, *J* = 8.48 Hz, 2H), 7.13 (d, *J* = 8.52 Hz, 2H), 3.62 (q, *J* = 6.12 Hz, 4H), 2.52 (q, *J* = 7.56 Hz, 2H), 1.97–1.92 (m, 4H), 1.12 (t, *J* = 7.56 Hz, 3H); ^13^C­{^1^H} NMR (100 MHz, DMSO-*d*_6_): δ 188.8, 163.9, 140.0, 136.3, 128.5, 120.2, 52.0, 51.9, 28.1, 26.1, 24.0, 16.2. HRMS (HR-ESI) *m*/*z*: [M + Na]^+^ calcd for C_14_H_18_N_2_OSNa, 285.1038; found, 285.1028.

#### *N*-(4-Methoxyphenyl)-2-(pyrrolidin-1-yl)-2-thioxoacetamide (**3d**)

The title compound was synthesized according to the general procedure. The crude mixture was purified using column chromatography by eluting with ethyl acetate/hexane (3:7) and obtained as a yellow solid (49.1 mg, 93%); mp 136–138 °C; ^1^H NMR (400 MHz, DMSO-*d*_6_): δ 10.3 (bs, 1H), 7.51 (d, *J* = 9.08 Hz, 2H), 6.87 (d, *J* = 9.08 Hz, 2H), 3.69 (s, 3H), 3.63 (d, *J* = 6.76 Hz, 4H), 1.94 (t, *J* = 3.24 Hz, 4H); ^13^C­{^1^H} NMR (100 MHz, CDCl_3_): δ 186.0, 159.8, 156.8, 130.3, 121.5, 114.2, 55.7, 55.5, 54.0, 26.9, 23.5. HRMS (HR-ESI) *m*/*z*: [M + Na]^+^ calcd for C_13_H_16_N_2_O_2_SNa, 287.0830; found, 287.0824.

#### *N*-(4-Fluorophenyl)-2-(pyrrolidin-1-yl)-2-thioxoacetamide (**3e**)

The title compound was synthesized according to the general procedure. The crude mixture was purified using column chromatography by eluting with ethyl acetate/hexane (3:7) and obtained as a pale-yellow solid (46.4 mg, 92%); mp. 136–138 °C; ^1^H NMR (400 MHz, DMSO-*d*_6_): δ 10.5 (bs, 1H), 7.62 (q, *J* = 5.08 Hz, 2H), 7.15 (t, *J* = 8.88 Hz, 2H), 3.63 (q, *J* = 5.80 Hz, 4H), 1.95 (t, *J* = 2.20 Hz, 4H); ^13^C­{^1^H} NMR (100 MHz, CDCl_3_): δ 185.5, 159.8 (d, *J*_C–F_ = 242.9 Hz), 159.7, 133.2, 121.5 (d, *J*_C–F_ = 7.89 Hz), 115.7 (d, *J*_C–F_ = 22.44 Hz), 55.9, 54.1, 26.9, 23.5; ^19^F NMR (376 MHz, DMSO-*d*_6_): δ −116.9. HRMS (HR-ESI) *m*/*z*: [M + Na]^+^ calcd for C_12_H_13_FN_2_OSNa, 275.0631; found, 275.0625.

#### *N*-(4-Chlorophenyl)-2-(pyrrolidin-1-yl)-2-thioxoacetamide (**3f**)

The title compound was synthesized according to the general procedure. The crude mixture was purified using column chromatography by eluting with ethyl acetate/hexane (3:7) and obtained as a yellow solid (47.2 mg, 88%); mp 140–142 °C; ^1^H NMR (400 MHz, DMSO-*d*_6_): δ 10.59 (bs, 1H), 7.63 (d, *J* = 9.08 Hz, 2H), 7.37 (d, *J* = 9.04 Hz, 2H), 3.63 (q, *J* = 7.24 Hz, 4H), 1.95 (t, *J* = 3.44 Hz, 4H); ^13^C­{^1^H} NMR (100 MHz, CDCl_3_): δ 185.4, 159.7, 135.8, 130.0, 129.2, 121.1, 56.0, 54.2, 27.0, 23.6. HRMS (HR-ESI) *m*/*z*: [M + Na]^+^ calcd for C_12_H_13_ClN_2_OSNa, 291.0335; found, 291.0323.

#### 4*N*-(4-Bromophenyl)-2-(pyrrolidin-1-yl)-2-thioxoacetamide (**3g**)

The title compound was synthesized according to the general procedure. The crude mixture was purified using column chromatography by eluting with ethyl acetate/hexane (3:7) and obtained as a yellow solid (56.8 mg, 91%); mp 151–153 °C; ^1^H NMR (400 MHz, DMSO-*d*_6_): δ 10.60 (bs, 1H), 7.58 (d, *J* = 8.96 Hz, 2H), 7.49 (d, *J* = 8.92 Hz, 2H), 3.63 (q, *J* = 5.72 Hz, 4H), 1.97–1.92 (m, 4H); ^13^C­{^1^H} NMR (100 MHz, CDCl_3_): δ 185.2, 159.5, 136.2, 132.0, 121.3, 117.6, 56.0, 54.2, 26.9, 23.5. HRMS (HR-ESI) *m*/*z*: [M + Na]^+^ calcd for C_12_H_13_BrN_2_OSNa, 334.9830; found, 334.9822.

#### *N*-(4-Nitrophenyl)-2-(pyrrolidin-1-yl)-2-thioxoacetamide (**3h**)

The title compound was synthesized according to the general procedure. The crude mixture was purified using column chromatography by eluting with ethyl acetate/hexane (3:7) and obtained as a brownish yellow solid (52.5 mg, 94%); mp 224–226 °C; ^1^H NMR (400 MHz, CDCl_3_): δ 10.10 (bs, 1H), 8.22 (d, *J* = 9.07 Hz, 2H), 7.77 (d, *J* = 9.16 Hz, 2H), 4.14 (t, *J* = 6.36 Hz, 2H), 3.89 (t, *J* = 7.00 Hz, 2H), 2.11–2.01 (m, 4H); ^13^C­{^1^H} NMR (100 MHz, CDCl_3_): δ 184.1, 159.3, 143.9, 142.7, 125.1, 119.2, 56.3, 54.3. HRMS (HR-ESI) *m*/*z*: [M + Na]^+^ calcd for C_12_H_13_N_3_O_3_SNa, 302.0576; found, 302.0562.

#### *N*-(4-Hydroxyphenyl)-2-(pyrrolidin-1-yl)-2-thioxoacetamide (**3i**)

The title compound was synthesized according to the general procedure. The crude mixture was purified using column chromatography by eluting with ethyl acetate/hexane (3:7) and obtained as a white solid (43.2 mg, 86%); mp 134–136 °C; ^1^H NMR (400 MHz, DMSO-*d*_6_): δ 10.15 (bs, 1H), 9.24 (bs, 1H), 7.38 (d, *J* = 8.92 Hz, 2H), 6.68 (d, *J* = 8.92 Hz, 2H), 3.62 (d, *J* = 6.56 Hz, 4H), 1.94 (t, *J* = 3.72 Hz, 4H); ^13^C­{^1^H} NMR (100 MHz, DMSO-*d*_6_): δ 189.1, 163.5, 154.3, 130.2, 121.8, 115.6, 52.0, 51.9, 26.1, 24.0. HRMS (HR-ESI) *m*/*z*: [M + Na]^+^ calcd for C_12_H_14_N_2_O_2_SNa, 250.0776; found, 250.0768.

#### *N*-(2-Iodophenyl)-2-(pyrrolidin-1-yl)-2-thioxoacetamide (**3j**)

The title compound was synthesized according to the general procedure. The crude mixture was purified using column chromatography by eluting with ethyl acetate/hexane (3:7) and obtained as a white solid (49.7 mg, 69%); mp 156–158 °C; ^1^H NMR (400 MHz, DMSO-*d*_6_): δ 10.01 (bs, 1H), 7.87 (d, *J* = 7.92 Hz, 1H), 7.55 (d, *J* = 7.80 Hz, 1H), 7.39 (t, *J* = 7.52 Hz, 1H), 6.99 (t, *J* = 7.64 Hz, 1H), 3.84 (t, *J* = 6.36 Hz, 2H), 3.68 (t, *J* = 6.56 Hz, 2H), 2.00–1.93 (m,4H); ^13^C­{^1^H} NMR (100 MHz, CDCl_3_): δ 185.4, 160.1, 139.3, 138.3, 129.1, 126.5, 121.3, 90.2, 55.9, 54.1, 27.0, 23.6. HRMS (HR-ESI) *m*/*z*: [M + Na]^+^ calcd for C_12_H_13_IN_2_OSNa, 382.9691; found, 382.9689.

#### 2-(Pyrrolidin-1-yl)-2-thioxo-*N*-(2-(trifluoromethyl)­phenyl)­acetamide (**3k**)

The title compound was synthesized according to the general procedure. The crude mixture was purified using column chromatography by eluting with ethyl acetate/hexane (3:7) and obtained as a yellow semisolid (54.4 mg, 90%); ^1^H NMR (400 MHz, CDCl_3_): δ 9.99 (bs, 1H), 8.22 (d, *J* = 8.24 Hz, 1H), 7.63 (d, *J* = 7.92 Hz, 1H), 7.56 (t, *J* = 7.72 Hz, 1H), 7.24 (t, *J* = 7.84 Hz, 1H), 4.09 (t, *J* = 6.44 Hz, 2H), 3.88 (t, *J* = 7.00 Hz, 2H), 2.10–1.97 (m, 4H); ^13^C­{^1^H} NMR (100 MHz, CDCl_3_): δ 185.0, 160.0, 134.6, 132.8, 126.3 (q, *J*_C–F_ = 271.9 Hz, 124.9 (d, *J*_C–F_ = 20.7 Hz), 123.4, 122.4 (d, *J*_C–F_ = 99.8 Hz), 121.1 (d, *J*_C–F_ = 142.5 Hz), 55.9, 54.0, 26.9, 23.5. ^19^F NMR (376 MHz, CDCl_3_): δ −60.8. HRMS (HR-ESI) *m*/*z*: [M + Na]^+^ calcd for C_13_H_13_F_3_N_2_OSNa, 325.0599; found, 325.0593.

#### *N*-(3-Nitrophenyl)-2-(pyrrolidin-1-yl)-2-thioxoacetamide (**3l**)

The title compound was synthesized according to the general procedure. The crude mixture was purified using column chromatography by eluting with ethyl acetate/hexane (3:7) and obtained as a yellow solid (51.9 mg, 93%); mp 194–196 °C; ^1^H NMR (400 MHz, DMSO-*d*_6_): δ 10.98 (bs, 1H), 8.62 (t, *J* = 2.16 Hz, 1H), 7.94 (dd, *J* = 8.16 Hz, 2.20 Hz, 2H), 7.61 (t, *J* = 8.16 Hz, 1H), 3.66 (t, *J* = 6.00 Hz, 4H), 2.00–1.93 (m, 4H); ^13^C­{^1^H} NMR (100 MHz, CDCl_3_): δ 184.4, 159.6, 148.8, 138.3, 129.9, 125.4, 119.5, 114.6, 56.3, 54.4, 27.0, 23.6. HRMS (HR-ESI) *m*/*z*: [M + Na]^+^ calcd for C_12_H_13_N_3_O_3_SNa, 302.0576; found, 302.0562.

#### *N*-(2,4-Dimethoxyphenyl)-2-(pyrrolidin-1-yl)-2-thioxoacetamide (**3m**)

The title compound was synthesized according to the general procedure. The crude mixture was purified using column chromatography by eluting with ethyl acetate/hexane (3:7) and obtained as a yellow solid (41.8 mg, 71%); mp 130–132 °C; ^1^H NMR (400 MHz, DMSO-*d*_6_): δ 9.51 (bs, 1H), 7.71 (d, *J* = 8.80 Hz, 1H), 6.62 (d, *J* = 2.68 Hz, 1H), 6.49 (dd, *J* = 8.80 Hz, 2.68 Hz, 1H), 3.79 (s, 3H) 3.76 (d, *J* = 6.84 Hz, 2H), 3.73 (s, 3H), 3.64 (t, *J* = 7.16 Hz, 2H), 1.97–1.91 (m, 4H); ^13^C­{^1^H} NMR (100 MHz, CDCl_3_): δ 186.8, 160.0, 157.2, 150.3, 120.8, 120.4, 103.8, 98.7, 55.9, 55.5, 55.3, 53.8, 26.9, 23.7. HRMS (HR-ESI) *m*/*z*: [M + Na]^+^ calcd for C_14_H_18_N_2_O_3_SNa, 317.0936; found, 317.0922.

#### *N*-Benzyl-2-(pyrrolidin-1-yl)-2-thioxoacetamide (**3n**)

The title compound was synthesized according to the general procedure. The crude mixture was purified using column chromatography by eluting with ethyl acetate/hexane (3:7) and obtained as a brown liquid (44.2 mg, 89%); ^1^H NMR (400 MHz, DMSO-*d*_6_): δ 8.86 (bs, 1H), 7.32–7.19 (m, 5H), 4.30 (d, *J* = 6.20 Hz, 2H), 3.61–3.54 (m, 4H), 1.91 (d, *J* = 6.76 Hz, 4H); ^13^C­{^1^H} NMR (100 MHz, CDCl_3_): δ 186.6, 163.1, 137.5, 128.9, 127.7, 127.7, 55.0, 23.7, 44.0, 26.8, 23.7. HRMS (HR-ESI) *m*/*z*: [M + Na]^+^ calcd for C_13_H_16_N_2_OSNa, 271.0881; found, 271.0868.

#### *N*-(Benzo­[*d*]­thiazol-2-yl)-2-(pyrrolidin-1-yl)-2-thioxoacetamide (**3o**)

The title compound was synthesized according to the general procedure. The crude mixture was purified using column chromatography by eluting with ethyl acetate/hexane (3:7) and obtained as a yellow solid (22.1 mg, 38%); mp 179–181 °C; ^1^H NMR (400 MHz, CDCl_3_): δ 7.82 (d, *J* = 9.08 Hz, 2H), 7.45 (t, *J* = 7.28 Hz, 1H), 7.32 (t, *J* = 7.88 Hz, 1H), 4.17 (t, *J* = 6.76 Hz, 2H), 3.89 (t, *J* = 6.96 Hz, 2H), 2.15–1.98 (m, 4H); ^13^C­{^1^H} NMR (100 MHz, CDCl_3_): δ 182.1, 148.9, 134.2, 127.7, 126.5, 124.4, 121.6, 121.5, 114.6, 56.3, 54.3, 27.0, 23.5. HRMS (HR-ESI) *m*/*z*: [M + H]^+^ calcd for C_13_H_13_N_3_OS_2_Na, 292.0578; found, 292.0573.

#### 2-(Dimethylamino)-*N*-phenyl-2-thioxoacetamide (**3r**)^24^

The title compound was synthesized according to the general procedure. The crude mixture was purified using column chromatography by eluting with ethyl acetate/hexane (3:7) and obtained as a yellow solid (29.5 mg, 71%); ^1^H NMR (400 MHz, DMSO-*d*_6_): δ 10.48 (bs, 1H), 7.59 (d, *J* = 8.60 Hz, 2H), 7.30 (t, *J* = 8.92 Hz, 2H), 7.44 (t, *J* = 7.40 Hz, 1H), 3.34 (s, 3H), 3.25 (s, 3H); ^13^C­{^1^H} NMR (100 MHz, CDCl_3_): δ 192.1, 161.7, 137.1, 129.2, 125.1, 120.0, 43.9, 43.6.

#### 2-(Diethylamino)-*N*-phenyl-2-thioxoacetamide (**3s**)^24^

The title compound was synthesized according to the general procedure. The crude mixture was purified using column chromatography by eluting with ethyl acetate/hexane (3:7) and obtained as a yellow solid (42.0 mg, 89%); ^1^H NMR (400 MHz, CDCl_3_): δ 8.45 (bs, 1H), 7.55 (d, *J* = 7.64 Hz, 2H), 7.34 (t, *J* = 7.60 Hz, 2H), 7.14 (t, *J* = 7.40 Hz, 1H), 3.97 (q, *J* = 7.16 Hz, 2H), 3.77 (q, *J* = 7.12 Hz, 2H), 1.38–1.31 (m, 6H); ^13^C­{^1^H} NMR (100 MHz, CDCl_3_): δ 191.3, 162.0, 137.2, 129.2, 125.0, 120.0, 49.0, 47.3, 14.3, 10.9.

#### *N*-Phenyl-2-(piperidin-1-yl)-2-thioxoacetamide (**3t**)

The title compound was synthesized according to the general procedure. The crude mixture was purified using column chromatography by eluting with ethyl acetate/hexane (3:7) and obtained as a yellow solid (49.1 mg, 99%); mp 132–134 °C; ^1^H NMR (400 MHz, CDCl_3_): δ 8.34 (bs, 1H), 7.56 (d, *J* = 7.64 Hz, 2H), 7.33 (t, *J* = 7.64 Hz, 2H), 7.14 (s, 1H), 4.17 (s, 2H), 3.84 (s, 2H), 1.76 (s, 6H); ^13^C­{^1^H} NMR (100 MHz, CDCl_3_): δ 190.7, 162.4, 162.3, 137.2, 129.1, 125.0, 119.9, 53.8, 50.4, 27.0, 25.4, 24.2. HRMS (HR-ESI) *m*/*z*: [M + Na]^+^ calcd for C_13_H_16_N_2_OSNa, 271.0881; found, 271.0871.

#### 2-Morpholino-*N*-phenyl-2-thioxoacetamide (**3u**)^25^

The title compound was synthesized according to the general procedure. The crude mixture was purified using column chromatography by eluting with ethyl acetate/hexane (7:3) and obtained as a yellow solid (43.5 mg, 87%); ^1^H NMR (400 MHz, DMSO-*d*_6_): δ 10.59 (bs, 1H), 7.59 (d, *J* = 7.52 Hz, 2H), 7.31 (t, *J* = 7.44 Hz, 2H), 7.08 (t, *J* = 7.40 Hz, 1H), 4.09 (t, *J* = 4.80 Hz, 2H), 3.72 (t, *J* = 5.08 Hz, 2H), 3.65 (s, 4H); ^13^C­{^1^H} NMR (100 MHz, DMSO-*d*_6_): δ 191.6, 163.5, 138.7, 129.4, 124.6, 120.1, 66.5, 66.0, 52.6, 47.5.

#### 2-(Cyclohexylamino)-*N*-phenyl-2-thioxoacetamide (**3v**)^26^

The title compound was synthesized according to the general procedure. The crude mixture was purified using column chromatography by eluting with ethyl acetate/hexane (3:7) and obtained as a yellow solid (33.0 mg, 63%); ^1^H NMR (400 MHz, CDCl_3_): δ 9.42 (bs, 1H), 7.64 (d, *J* = 7.56 Hz, 2H), 7.37 (t, *J* = 7.52 Hz, 2H), 7.17 (t, *J* = 7.40 Hz, 1H), 4.28–4.19 (m, 1H), 2.08 (d, *J* = 12.0 Hz, 2H), 1.82–1.76 (m, 2H), 1.70–1.65 (m, 1H), 1.49–1.22 (m, 5H); ^13^C­{^1^H} NMR (100 MHz, CDCl_3_): δ 184.4, 156.1, 136.8, 129.3, 125.3, 119.8, 55.0, 31.0, 25.5, 24.6.

#### 2-(Butylamino)-*N*-phenyl-2-thioxoacetamide (**3w**)^27^

The title compound was synthesized according to the general procedure. The crude mixture was purified using column chromatography by eluting with ethyl acetate/hexane (3:7) and obtained as a yellow solid (35.9 mg, 76%); ^1^H NMR (400 MHz, CDCl_3_): δ 9.54 (s, 1H), 7.65 (d, *J* = 8.72 Hz, 2H), 7.37 (t, *J* = 7.68 Hz, 2H), 7.18 (t, *J* = 7.48 Hz, 1H), 3.70 (q, *J* = 7.52 Hz, 2H), 1.73 (t, *J* = 7.84 Hz, 2H), 1.44 (q, *J* = 7.60 Hz, 2H), 0.97 (t, *J* = 7.48 Hz, 3H); ^13^C­{^1^H} NMR (100 MHz, CDCl_3_): δ 186.0, 156.0, 136.7, 129.3, 125.4, 119.9, 46.0, 46.3, 29.7, 20.3, 13.8.

#### 2-((2-Hydroxyethyl)­amino)-*N*-phenyl-2-thioxoacetamide (**3x**)

The title compound was synthesized according to the general procedure. The crude mixture was purified using column chromatography by eluting with ethyl acetate/hexane (3:7) and obtained as a yellow sticky solid (24.6 mg, 55%); ^1^H NMR (400 MHz, CDCl_3_): δ 10.77 (bs, 1H), 10.35 (bs, 1H), 7.73 (d, *J* = 8.08 Hz, 2H), 7.34 (t, *J* = 7.64 Hz, 2H), 7.13 (t, *J* = 7.28 Hz, 1H), 4.89 (s, 1H), 3.65 (s, 4H); ^13^C­{^1^H} NMR (100 MHz, CDCl_3_): δ 186.9, 156.2, 136.5, 129.3, 125.6, 120.1, 59.7, 48.6. HRMS (HR-ESI) *m*/*z*: [M + Na]^+^ calcd for C_10_H_12_N_2_O_2_SNa, 247.0517; found, 247.0509.

#### 2-(Butylamino)-2-thioxo-*N*-(2-(trifluoromethyl)­phenyl)­acetamide (**3t**)

The title compound was synthesized according to the general procedure. The crude mixture was purified using column chromatography by eluting with ethyl acetate/hexane (3:7) and obtained as a yellow solid (31.0 mg, 51%); mp. 45–47 °C; ^1^H NMR (400 MHz, CDCl_3_): δ 9.46 (bs, 1H), 8.29 (d, *J* = 8.28 Hz, 1H), 7.66 (d, *J* = 8.52 Hz, 1H), 7.59 (t, *J* = 7.48 Hz, 1H), 7.28 (t, *J* = 7.68 Hz, 1H), 3.70 (q, *J* = 7.28 Hz, 2H), 1.72 (p, *J* = 7.40 Hz, 2H), 1.48–1.39 (m, 2H), 0.97 (t, *J* = 7.36 Hz, 3H); ^13^C­{^1^H} NMR (100 MHz, CDCl_3_): δ 185.4, 156.5, 134.4, 133.0 (d, *J*_C–F_ = 139.3 Hz), 126.5, 125.6 (q, *J*_C–F_ = 240.0 Hz), 125.2, 122.8, 121.1 (d, *J*_C–F_ = 30.3 Hz). ^19^F NMR (376 MHz, CDCl_3_): δ −65.7. HRMS (HR-EI) *m*/*z*: [M^+^] calcd for C_13_H_15_F_3_N_2_OS, 304.0857; found, 304.0852.

#### *N*-Benzyl-2-(butylamino)-2-thioxoacetamide (**3z**)

The title compound was synthesized according to the general procedure. The crude mixture was purified using column chromatography by eluting with ethyl acetate/hexane (3:7) and obtained as a yellow solid (22.0 mg, 44%); mp 47–49 °C; ^1^H NMR (400 MHz, CDCl_3_): δ 9.51 (bs, 1H), 8.57 (bs, 1H), 7.36–7.25 (m, 5H), 4.50 (d, *J* = 6.20 Hz, 2H), 3.65 (q, *J* = 7.24 Hz, 2H), 1.69 (p, *J* = 7.32 Hz, 2H), 1.46–1.37 (m, 2H), 0.95 (t, *J* = 7.36 Hz, 3H); ^13^C­{^1^H} NMR (100 MHz, CDCl_3_): δ 185.6, 158.6, 136.9, 128.9, 1127.9, 127.8, 46.0, 44.7, 29.7, 20.3, 13.8. HRMS (HR-ESI) *m*/*z*: [M + Na]^+^ calcd for C_13_H_18_N_2_OSNa, 273.1027; found, 273.1038.

#### 2-((4-Chlorophenyl)­amino)-*N*-phenyl-2-thioxoacetamide (**3aa**)^28^

The title compound was synthesized according to the general procedure. The crude mixture was purified using column chromatography by eluting with ethyl acetate/hexane (3:7) and obtained as a pale-yellow solid (23.8 mg, 41%); ^1^H NMR (400 MHz, DMSO-*d*_6_): δ 12.44 (bs, 1H), 10.49 (bs, 1H), 7.97 (d, *J* = 8.48 Hz, 2H), 7.75 (q, *J* = 8.20 Hz, 2H), 7.50 (d, *J* = 8.44 Hz, 2H), 7.36 (t, *J* = 7.68 Hz, 2H), 7.14 (t, *J* = 7.16 Hz, 1H); ^13^C­{^1^H} NMR (100 MHz, CDCl_3_): δ 182.5, 156.2, 136.6, 136.2, 132.77, 129.5, 129.4, 125.7, 123.3, 119.9.

#### 2-(Dimethylamino)-2-thioxo-*N*-(*p*-tolyl)­acetamide (**3ab**)^24^

The title compound was synthesized according to the general procedure. The crude mixture was purified using column chromatography by eluting with ethyl acetate/hexane (3:7) and obtained as a white solid (28 mg, 63%); ^1^H NMR (400 MHz, DMSO-*d*_6_): δ 10.41 (bs, 1H), 7.50 (d, *J* = 8.48 Hz, 2H), 7.14 (d, *J* = 8.12 Hz, 2H), 3.36 (s, 3H), 3.27 (s, 3H), 2.26 (s, 3H); ^13^C­{^1^H} NMR (100 MHz, DMSO-*d*_6_): δ 192.7, 163.9, 136.2, 133.5, 129.7, 120.0, 42.9, 20.9.

#### *N*-(4-Chlorophenyl)-2-(dimethylamino)-2-thioxoacetamide (**3ac**)^24^

The title compound was synthesized according to the general procedure. The crude mixture was purified using column chromatography by eluting with ethyl acetate/hexane (3:7) and obtained as a white solid (41.3 mg, 85%); ^1^H NMR (400 MHz, DMSO-*d*_6_): δ 10.67 (bs, 1H), 7.77 (t, *J* = 3.08 Hz, 1H), 7.64 (t, *J* = 2.20 Hz, 1H), 7.41 (t, *J* = 3.04 Hz, 1H), 7.39 (t, *J* = 2.20 Hz, 1H), 3.37 (s, 3H), 3.28 (s, 3H); ^13^C­{^1^H} NMR (100 MHz, DMSO-*d*_6_): δ 192.1, 164.0, 137.6, 129.2, 128.1, 121.6, 43.0.

#### 2*N*-(2-(1*H*-Indol-2-yl)­ethyl)-2-(butylamino)-2-thioxoacetamide (**3ad**)

The title compound was synthesized according to the general procedure. The crude mixture was purified using column chromatography by eluting with ethyl acetate/hexane (3:7) and obtained as a brown sticky solid (25.5 mg, 42%); ^1^H NMR (400 MHz, CDCl_3_): δ 9.50 (bs, 1H), 8.37 (bs, 1H), 8.13 (bs, 1H), 7.62 (d, *J* = 7.84 Hz, 1H), 7.36 (d, *J* = 8.12 Hz, 1H), 7.21 (t, *J* = 8.12 Hz, 1H), 7.13 (t, *J* = 7.96 Hz, 1H), 7.06 (s, 1H), 3.67–3.61 (m, 4H), 3.04 (t, *J* = 6.84 Hz, 2H), 1.71–1.63 (m, 2H), 1.43–1.38 (m, 2H), 0.947 (t, *J* = 7.40 Hz, 3H); ^13^C­{^1^H} NMR (100 MHz, CDCl_3_): δ 186.1, 158.6, 136.5, 127.2, 122.4, 122.3, 119.6, 118.8, 112.5, 111.3, 45.9, 40.9, 29.8, 25.1, 20.3. HRMS (HR-ESI) *m*/*z*: [M + Na]^+^ calcd for C_16_H_21_N_3_OSNa, 326.1303; found, 326.1304.

#### 2-((3,4-Dihydroxyphenethyl)­amino)-*N*-phenyl-2-thioxoacetamide (**3ae**)

The title compound was synthesized according to the general procedure. The crude mixture was purified using column chromatography by eluting with ethyl acetate/hexane (3:7) and obtained as a yellow solid (31.0 mg, 49%); mp 109–111 °C; ^1^H NMR (400 MHz, DMSO-*d*_6_): δ 10.94 (bs, 1H), 10.35 (bs, 1H), 8.76 (s, 1H), 8.65 (s, 1H), 7.72 (d, *J* = 7.64 Hz, 2H), 7.34 (t, *J* = 7.56 Hz, 2H), 7.13 (t, *J* = 7.44 Hz, 1H), 6.62 (t, *J* = 5.12 Hz, 2H), 6.46 (dd, *J* = 8.08 Hz, 2.08 Hz, 1H), 3.71 (q, *J* = 6.00 Hz, 2H), 2.74 (t, *J* = 7.92 Hz, 2H); ^13^C­{^1^H} NMR (100 MHz, CDCl_3_): δ 187.3, 158.8, 145.6, 144.2, 137.8, 129.7, 129.3, 125.1, 120.6, 119.6, 116.3, 116.0, 47.8, 32.2. HRMS (HR-ESI) *m*/*z*: [M + Na]^+^ calcd for C_16_H_16_N_2_O_3_SNa, 339.0780; found, 339.0769.

#### *N*,*N*-Dimethyl-2-oxo-2-phenylethanethioamide (**5a**)^29^

The title compound was synthesized according to the general procedure. The crude mixture was purified using column chromatography by eluting with ethyl acetate/hexane (3:7) and obtained as a yellow solid (27.1 mg, 71%); ^1^H NMR (400 MHz, CDCl_3_): δ 7.86–7.84 (m, 2H), 7.70–7.66 (m, 1H), 7.57–7.52 (m, 2H), 3.45 (s, 3H), 3.20 (s, 3H); ^13^C­{^1^H} NMR (100 MHz, CDCl_3_): δ 196.9, 188.5, 134.3, 133.3, 130.0, 128.9, 42.5, 40.5.

#### *N*,*N*-Diethyl-2-oxo-2-phenylethanethioamide (**5b**)^18^

The title compound was synthesized according to the general procedure. The crude mixture was purified using column chromatography by eluting with ethyl acetate/hexane (3:7) and obtained as a yellow solid (33.6 mg, 76%); ^1^H NMR (400 MHz, CDCl_3_): δ 7.96 (d, *J* = 7.96 Hz, 2H), 7.59–7.43 (m, 3H), 4.05 (t, *J* = 6.96 Hz, 2H), 3.47 (d, *J* = 7.12 Hz, 2H), 1.39 (t, *J* = 7.16 Hz, 3H), 1.27–1.19 (m, 3H); ^13^C­{^1^H} NMR (100 MHz, CDCl_3_): δ 195.7, 187.6, 134.1, 133.6, 130.0, 128.8, 48.0, 44.6, 13.7, 11.3.

#### 1-Phenyl-2-(pyrrolidin-1-yl)-2-thioxoethan-1-one (**5c**)^18^

The title compound was synthesized according to the general procedure. The crude mixture was purified using column chromatography by eluting with ethyl acetate/hexane (3:7) and obtained as a brown solid (40.3 mg, 92%); ^1^H NMR (400 MHz, DMSO-*d*_6_): δ 7.88 (d, *J* = 8.08 Hz, 2H), 7.68 (t, *J* = 6.92 Hz, 1H), 7.54 (t, *J* = 7.40 Hz, 2H), 3.79 (t, *J* = 6.32 Hz, 2H), 3.43 (t, *J* = 6.52 Hz, 2H), 1.96 (p, *J* = 7.16 Hz, 4H); ^13^C­{^1^H} NMR (100 MHz, DMSO-*d*_6_): δ 191.0, 188.0, 134.4, 132.4, 129.6, 129.0, 51.3, 50.1, 25.5, 23.2.

#### 1-Phenyl-2-(piperidin-1-yl)-2-thioxoethan-1-one (**5d**)^18^

The title compound was synthesized according to the general procedure. The crude mixture was purified using column chromatography by eluting with ethyl acetate/hexane (3:7) and obtained as a brown solid (37.3 mg, 80%); ^1^H NMR (400 MHz, CDCl_3_): δ 7.98 (d, *J* = 7.08 Hz, 2H), 7.58 (t, *J* = 7.40 Hz, 1H), 7.47 (t, *J* = 7.84 Hz, 2H), 4.24 (t, *J* = 5.84 Hz, 2H), 3.52 (t, *J* = 6.40 Hz, 2H), 1.82–1.73 (m, 6H); ^13^C­{^1^H} NMR (100 MHz, CDCl_3_): δ 194.5, 188.1, 134.2, 133.5, 129.9, 128.9, 53.1, 48.2, 29.8, 26.5, 25.4, 24.2.

#### 2-(4-Methylpiperazin-1-yl)-1-phenyl-2-thioxoethan-1-one (**5e**)^30^

The title compound was synthesized according to the general procedure. The crude mixture was purified using column chromatography by eluting with ethyl acetate/hexane (3:7) and obtained as a brown solid (39.2 mg, 79%); ^1^H NMR (400 MHz, CDCl_3_): δ 7.98 (d, *J* = 8.16 Hz, 2H), 7.60 (t, *J* = 7.44 Hz, 1H), 4.48 (t, *J* = 8.04 Hz, 2H), 4.32 (t, *J* = 5.12 Hz, 2H), 3.58 (t, *J* = 4.88 Hz, 2H), 2.62 (t, *J* = 5.20 Hz, 2H), 2.42 (q, *J* = 4.08 Hz, 2H), 2.33 (s, 3H); ^13^C­{^1^H} NMR (100 MHz, CDCl_3_): δ 195.5, 188.0, 134.4, 133.4, 129.9, 129.0, 54.8, 54.2, 51.5, 46.8, 45.7.

#### 2-Morpholino-1-phenyl-2-thioxoethan-1-one (**5f**)^18^

The title compound was synthesized according to the general procedure. The crude mixture was purified using column chromatography by eluting with ethyl acetate/hexane (3:7) and obtained as a yellow solid (39.0 mg, 83%); ^1^H NMR (400 MHz, CDCl_3_): δ 7.99–7.96 (m, 2H), 7.62–7.58 (m, 1H), 7.50–7.46 (m, 2H), 4.31 (t, *J* = 4.88 Hz, 2H), 3.89 (t, *J* = 5.08 Hz, 2H), 3.69–3.66 (m, 2H), 3.58 (t, *J* = 5.24 Hz, 2H); ^13^C­{^1^H} NMR (100 MHz, CDCl_3_): δ 195.8, 188.0, 134.5, 133.3, 129.9, 129.0, 66.6, 66.5, 52.0, 47.2.

#### 2-Morpholino-1-phenyl-2-thioxoethan-1-one (**5g**)

The title compound was synthesized according to the general procedure. The crude mixture was purified using column chromatography by eluting with ethyl acetate/hexane (3:7) and obtained as a yellow semisolid (32.6 mg, 78%); ^1^H NMR (400 MHz, DMSO-*d*_6_): δ 11.14 (bs, 1H), 7.86 (d, *J* = 7.96 Hz, 2H), 7.64 (t, *J* = 7.40 Hz, 1H), 7.54 (t, *J* = 7.88 Hz, 2H), 4.91 (s, 1H), 3.74 (s, 2H), 3.66 (s, 2H); ^13^C­{^1^H} NMR (100 MHz, CDCl_3_): δ 195.1, 188.5, 134.1, 133.5, 130.8, 128.4, 60.0, 47.1. HRMS (HR-ESI) *m*/*z*: [M + Na]^+^ calcd for C_10_H_11_NO_2_SNa, 232.0408; found, 232.0396.

#### 2-Oxo-*N*-phenethyl-2-phenylethanethioamide (**5h**)^16^

The title compound was synthesized according to the general procedure. The crude mixture was purified using column chromatography by eluting with ethyl acetate/hexane (3:7) and obtained as a yellow solid (33.9 mg, 63%); ^1^H NMR (400 MHz, DMSO-*d*_6_): δ 8.27 (bs, 1H), 7.98 (d, *J* = 7.96 Hz, 2H), 7.56 (t, *J* = 7.32 Hz, 1H), 7.41 (t, *J* = 7.84 Hz, 2H), 7.33 (t, *J* = 7.68 Hz, 2H), 7.25 (d, *J* = 8.80 Hz, 3H), 4.06 (q, *J* = 7.04 Hz, 2H), 3.06 (t, *J* = 7.08 Hz, 2H); ^13^C­{^1^H} NMR (100 MHz, CDCl_3_): δ 193.8, 187.7, 137.8, 133.9, 133.8, 130.8, 129.0, 128.8, 128.2, 127.1, 46.1, 33.8.

#### *N*-Cyclohexyl-2-oxo-2-phenylethanethioamide (**5i**)^31^

The title compound was synthesized according to the general procedure. The crude mixture was purified using column chromatography by eluting with ethyl acetate/hexane (3:7) and obtained as a yellow solid (34.1 mg, 69%); ^1^H NMR (400 MHz, CDCl_3_): δ 8.21 (bs, 1H), 8.00 (dd, *J* = 8.16 Hz, 1.04 Hz, 2H), 7.55 (t, *J* = 7.48 Hz, 1H), 7.41 (t, *J* = 7.96 Hz, 2H), 4.49–4.40 (m, 1H), 2.14 (d, *J* = 11.88 Hz, 2H), 1.81–1.76 (m, 2H), 1.70–1.65 (m, 1H), 1.50–1.34 (m, 4H); ^13^C­{^1^H} NMR (100 MHz, CDCl_3_): δ 192.4, 188.0, 133.9, 133.8, 130.8, 128.2, 53.7, 31.3, 25.4, 24.6.

#### *N*-(1-Benzylpyrrolidin-3-yl)-2-oxo-2-phenylethanethioamide (**5j**)

The title compound was synthesized according to the general procedure. The crude mixture was purified using column chromatography by eluting with ethyl acetate/hexane (3:7) and obtained as a brown semisolid (33.1 mg, 51%); ^1^H NMR (400 MHz, DMSO-*d*_6_): δ 8.02–8.00 (m, 2H), 7.43–7.40 (m, 2H), 7.32–7.28 (m, 5H), 5.05–5.00 (m, 1H), 3.66 (s, 2H), 3.04–2.98 (m, 1H), 2.90 (d, *J* = 10.36 Hz, 1H), 2.69–2.59 (m, 1H), 2.47–2.31 (m, 3H), 1.92–1.84 (m, 1H); ^13^C­{^1^H} NMR (100 MHz, CDCl_3_): δ 192.9, 187.8, 133.9, 133.8, 130.8, 129.5, 129.0, 128.6, 128.3, 127.5, 59.7, 59.3, 54.3, 52.4, 31.5. HRMS (HR-ESI) *m*/*z*: [M + H]^+^ calcd for C_19_H_21_N_2_OS, 325.1374; found, 325.1360.

#### 2,2′-(Piperazine-1,4-diyl)­bis­(1-phenyl-2-thioxoethan-1-one) (**5k**)

The title compound was synthesized according to the general procedure. The crude mixture was purified using column chromatography by eluting with ethyl acetate/hexane (3:7) and obtained as a yellow solid (29.8 mg, 39%); mp 155–157 °C; ^1^H NMR (400 MHz, DMSO-*d*_6_): δ 7.96–7.88 (m, 4H), 7.73–7.65 (m, 2H), 7.59–7.51 (m, 4H), 4.49 (s, 2H), 4.20 (t, *J* = 4.72 Hz, 2H), 3.94 (t, *J* = 5.48 Hz, 2H), 3.66 (s, 2H); ^13^C­{^1^H} NMR (100 MHz, CDCl_3_): δ 197.2, 187.9, 134.9, 134.8, 133.1, 133.0, 130.1, 130.0, 129.2, 129.1, 50.8, 50.1, 46.6, 45.9. HRMS (HR-ESI) *m*/*z*: [M + Na]^+^ calcd for C_20_H_18_N_2_O_2_S_2_Na, 405.0695; found, 405.0708.

#### 1-(4-Methoxyphenyl)-2-(pyrrolidin-1-yl)-2-thioxoethan-1-one (**5l**)^18^

The title compound was synthesized according to the general procedure. The crude mixture was purified using column chromatography by eluting with ethyl acetate/hexane (3:7) and obtained as a yellow solid (37.9 mg, 76%); ^1^H NMR (400 MHz, DMSO-*d*_6_): δ 7.88 (t, *J* = 2.88 Hz, 1H), 7.86 (t, *J* = 2.12 Hz, 1H), 7.10 (t, *J* = 2.88 Hz, 1H), 7.08 (t, *J* = 2.12 Hz, 1H), 3.86 (s, 3H), 3.81 (t, *J* = 6.92 Hz, 2H), 3.43 (t, *J* = 6.56 Hz, 2H), 2.04–1.92 (m,4H); ^13^C­{^1^H} NMR (100 MHz, DMSO-*d*_6_): δ 192.1, 187.8, 164.6, 132.6, 125.6, 114.9, 56.2, 51.7, 51.5, 26.0, 23.7.

#### 1-(4-Chlorophenyl)-2-(pyrrolidin-1-yl)-2-thioxoethan-1-one (**5m**)^32^

The title compound was synthesized according to the general procedure. The crude mixture was purified using column chromatography by eluting with ethyl acetate/hexane (3:7) and obtained as a yellow solid (41.1 mg, 81%); ^1^H NMR (400 MHz, DMSO-*d*_6_): δ 7.93 (t, *J* = 2.48 Hz, 1H), 7.91 (t, *J* = 1.92 Hz, 1H), 7.66 (t, *J* = 2.40 Hz, 1H), 7.64 (t, *J* = 1.80 Hz, 1H), 3.82 (t, *J* = 7.04 Hz, 2H), 3.48 (t, *J* = 6.28 Hz, 2H), 2.05–1.94 (m, 4H); ^13^C­{^1^H} NMR (100 MHz, DMSO-*d*_6_): δ 190.8, 187.3, 139.4, 132.0, 131.8, 129.8, 51.9, 51.6, 26.1, 23.8.

## Supplementary Material



## Data Availability

The data underlying this study are available in the published article and its Supporting Information.

## Notes

Detailed optimization, control experiments, experimental procedure, and ^1^H and ^13^C NMR spectra of compounds (PDF)
