# Supplementary material for: Activation of gem-Dichloroacetamides and Epoxides Using Elemental Sulfur and Amines: A Route to Monothiooxalamides and α‑Ketothioamides
Source: J Org Chem. 2025 Oct 2;90(41):14673–82. doi: 10.1021/acs.joc.5c01684 (PMC12538588; doi:10.1021/acs.joc.5c01684)
Supplement: Supplementary file 1 [file jo5c01684_si_001.pdf]

Supporting Information for

Activation of *Gem*-Dichloroacetamides and Epoxides Using Elemental Sulfur and Amines: A Route to Monothiooxalamides and *α*-Ketothioamides

Alageswaran Jayaram, [a] Yu- Ming Liu, [a] Nian-Qi Chen, [a] Genin Gary Huang [a] Gopal Chandru Senadi [a, b] and Wei-Yu Lin [a,c,d] *

[a] Department of Medicinal and Applied Chemistry, Kaohsiung Medical University, Kaohsiung, Taiwan.

[b] Department of Chemistry, Faculty of Engineering and Technology SRM Institute of Science and Technology Kattankulathur, Tamilnadu – 603203, India.

[c] Department of Medical Research, Kaohsiung Medical University Hospital, Kaohsiung, Taiwan.

[d] Drug Development and Value Creation Research Centre, Kaohsiung Medical University, Kaohsiung, Taiwan.

*Corresponding author: Wei-Yu Lin, wylin@kmu.edu.tw

Table of Contents

1	General information	S3
2	Table S1. Optimization for 2-morpholino-1-phenyl-2-thioxoetha 1-one derivatives	S4
3	Synthesis of starting materials (1a-o)	S5
4	Experimental procedure 2-(pyrrolidin-1-yl)-2-thioxo derivatives (3a-3o), & amine derivatives (3r-3aa)	S5
5	Experimental procedure 2-amino-1-phenyl-2-thioxoethan-1-one derivatives (5a-5m)	S6
6	Experimental procedure amide derivatives (3r, 3ab, 3ac, 3s):	S7
7	General procedure for Gram-scale synthesis	S7-8
8	Experimental procedure for late-stage modifications (3ad & 3ae)	S9-10
9	References	S10
10	^{1}H and ^{13}C spectra	S11-98

1. General information

All chemicals were purchased from commercial providers (Sigma Aldrich, Alfa Aesar, TCI, and matrix scientific) and used directly without further purification, unless otherwise noted. Well cleaned and oven dried glassware was used for the experiments. Reaction was monitored by Thin Layer Chromatography (TLC), purchased as pre-coated with silica gel 60 F254 from Merck. Column chromatography was carried out using silica gel 230-400 mesh (purchased from Merck) with mixture of ethyl acetate/hexane or hexane as the eluent. ^{1}H NMR spectra were recorded on 400 MHz, ^{13}C-NMR spectra were recorded on 100 MHz, Varian mercury spectrometer using $CDCl_3$ or DMSO-d_6 as solvent. The spectra were recorded and presented in chemical shifts (ppm) with tetramethylsilane (TMS) used as internal standard. Multiplicities were provided in s (singlet), d (doublet), t (triplet), q (quartet), bs (broad singlet), m (multiplet) and dd (doublet of doublet). Coupling constants (*J*) were reported in Hz. All the compounds were characterized by EI mass on Thermo Finnigan (TRACEGC- POLARISQ) and HRMS (EI^+ mode) on JMS-700 spectrometer. Melting points were determined using Fargo instruments.

2. Table S1. Optimization for 2-morpholino-1-phenyl-2-thioxoethan-1-one derivatives [a-b]

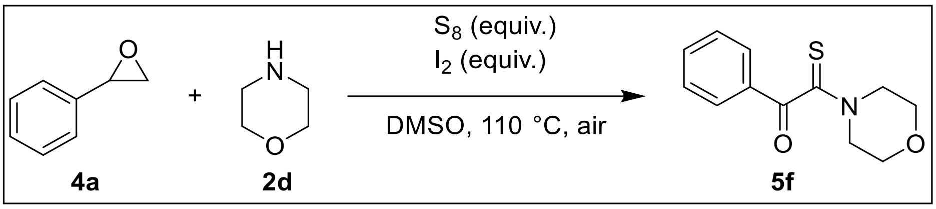

Entry	I_2 (equiv.)	S_8 (equiv.)	Solvent	Temperature (°C)	Yield [b]
1	0.2	2	DMSO	110	77
2	0.5	2	DMSO	110	81
3	1	2	DMSO	110	45
4	-	2	DMSO	110	38
5	0.5	0.5	DMSO	110	66
6	0.5	1	DMSO	110	81
7	0.5	1.5	DMSO	110	83
8	0.5	1.5	DMSO	80	73
9	0.5	1.5	DMSO	100	79
10	0.5	1.5	DMF	110	traces
11	0.5	1.5	1,4-Dioxane	110	N.R.
12	0.5	1.5	H_2O	110	N.R.
13	0.5	1.5	toluene	110	N.R.

[a] Reaction conditions: **4a** (0.2 mmol scale), **2d** (equiv.), **S_8** (equiv.), **I_2** (equiv.) at 110 °C temperature under open air for 2-4 h, [b] Isolated yield, N.R. = no reaction

3. Synthesis of starting materials (1a-o): [1]

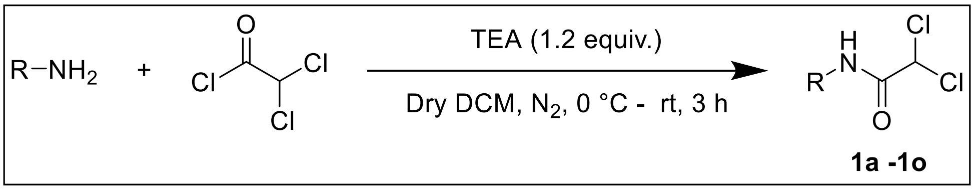

Scheme S1. General procedures for the synthesis of starting materials

All the starting materials (**1a-o**) were synthesized on 3.0 mmol scale, according to the literature procedure, and obtained in 20% - 93% yield, unless otherwise noted. The ^{1}H-NMR spectra of known starting materials were matched with previous literatures.

4. Experimental procedure 2-(pyrrolidin-1-yl)-2-thioxo derivatives (3a-3o), & amine derivatives (3r-3aa):

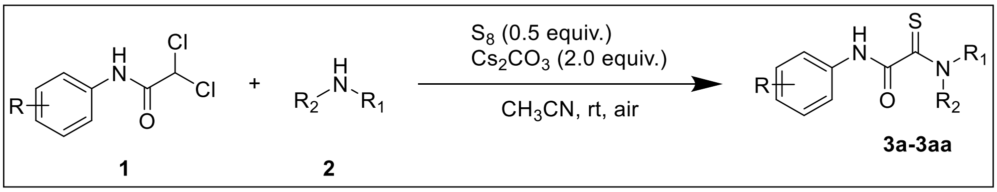

Scheme S2. General procedure for the synthesis of mono thiooxalamide derivatives

A 15 mL reaction tube was charged with **(1a-1o)** (0.2 mmol, 1.0 equiv.), S_8 (0.1 mmol, 0.5 equiv.), CS_2CO_3 (0.4 mmol, 2.0 equiv.), amine **2** (0.4 mmol, 2.0 equiv.), with CH_3CN (2 mL). The resulting mixture was stirred at room temperature in the open-air atmosphere for about 12 h. After the completion of the reaction, reaction mixture was diluted with 5 mL of water. The aqueous layer was extracted with Ethyl acetate (3 × 10 mL), and the combined organic layer was washed with brine solution (1 × 5 mL). The final organic layer was then dried over $MgSO_4$ and concentrated under reduced pressure to get the crude product. The obtained crude product was purified using column chromatography by eluting with ethyl acetate/hexane (3:7) to afford pure 2-

(pyrrolidin-1-yl)-2-thioxo derivatives (**3a-3o)** up to 38-94% yields and pure amine derivatives **(3r-3aa)** up to 41-99% yields.

5. **Experimental procedure 2-amino-1-phenyl-2-thioxoethan-1-one derivatives (5a-5m):**

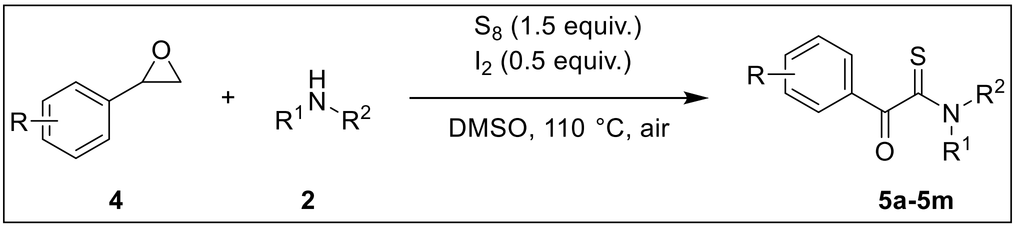

Scheme S3. General procedure for the synthesis of keto thioamide derivatives

A 15 mL reaction tube was charged with aryl epoxides (**4**) (0.2 mmol, 1 equiv.), iodine (0.1 mmol, 0.5 equiv.) and dimethyl sulfoxide (2 mL) and the resulting mixture was stirred at 110 °C, the reaction tube was removed after about 1 hour. Then additional **2** (0.4 mmol, 2.0 equiv.), and S_8 (0.4 mmol, 2.0 equiv.) were added at room temperature. The reaction mixture was stirred at 110 °C in an oil bath for about 2−4 h, then it was allowed to reach room temperature, and quenched with a saturated solution of $Na_2S_2O_3$. After diluting with 5 mL of water, the aqueous layer was extracted with ethyl acetate (3 × 10 mL), and the combined ethyl acetate layer was washed with a brine solution (1 × 5 mL). The final ethyl acetate layer was then dried over $MgSO_4$ and concentrated under reduced pressure to get the crude product. The obtained crude product was purified using column chromatography by eluting with ethyl acetate/hexane (3:7) to afford pure 2-amino-1-phenyl-2-thioxoethan-1-one derivatives **5a**−**5m** up to 39−92% yields.

6. Experimental procedure amide derivatives (3r, 3ab, 3ac, 3s):

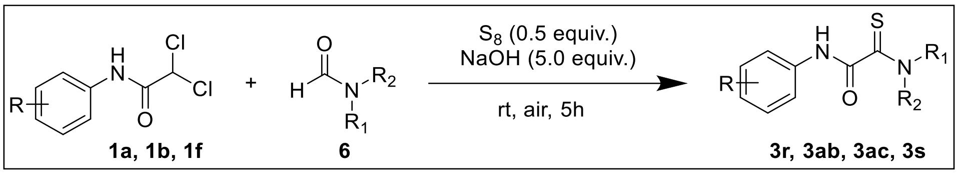

Scheme S4. General procedure for the synthesis of mono thiooxalamide derivatives

A 15 mL reaction tube was charged with (**1a, 1b, 1f)** (0.2 mmol, 1.0 equiv.), S_8 (0.1 mmol, 0.5 equiv.), NaOH (1.0 mmol, 5.0 equiv.), amide **6** (2.0 mL). The resulting mixture was stirred at room temperature in the open-air atmosphere for about 5 h. After the completion of the reaction, reaction mixture was diluted with 5 mL of water. The aqueous layer was extracted with Ethyl acetate (3 × 10 mL), and the combined organic layer was washed with brine solution (1 × 5 mL). The final organic layer was then dried over $MgSO_4$ and concentrated under reduced pressure to get the crude product. The obtained crude product was purified using column chromatography by eluting with ethyl acetate/hexane (3:7) to afford pure derivatives (**3r, 3ab, 3ac, 3s)** up to 63-85% yields.

7. General procedure for Gram-scale synthesis

i) Experimental Procedure for Gram-Scale Synthesis of *N*-phenyl-2-(pyrrolidin-1-yl)-2-thioxoacetamide (3a):

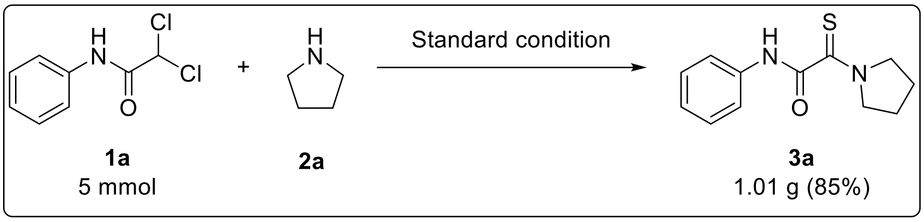

Scheme S5. General procedure for the gram scale synthesis of mono thiooxalamide derivatives

A 100 mL round-bottom flask was charged with (**1a)** (5.0 mmol, 1.0 equiv.), amine (**2a**) (10.0 mmol, 2.0 equiv.) S_8 (2.5 mmol, 0.5 equiv.), CS_2CO_3 (10 mmol, 2.0 equiv.)

with CH_3CN (60 mL). The resulting mixture was stirred at room temperature in the open-air atmosphere for about 12 h. After the completion of the reaction, the reaction mixture was diluted with 100 mL of water. The aqueous layer was extracted with Ethyl acetate (3 × 100 mL), and the combined organic layer was washed with brine solution (1 × 100 mL). The final organic layer was then dried over $MgSO_4$ and concentrated under reduced pressure to get the crude product. The obtained crude product was purified using column chromatography by eluting with ethyl acetate/hexane (3:7) to afford pure *N*-phenyl-2-(pyrrolidin-1-yl)-2-thioxoacetamide (**3a)** 85% yields.

ii) Experimental Procedure for Gram-Scale Synthesis of 2-morpholino-1-phenyl-2-thioxoethan-1-one (5f):

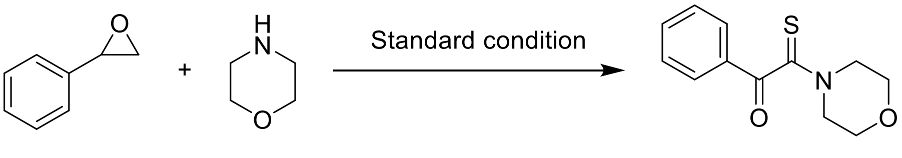

4a 6.5 mmol **2d** **5f** 1.12 g (73%)

Scheme S6. General procedure for the gram scale synthesis of keto thioamide derivatives

A 100 mL round-bottom flask was charged with 2-phenyloxirane (**4a**) (6.5 mmol, 1 equiv.), iodine (3.25 mmol, 0.5 equiv.) and dimethyl sulfoxide (30 mL) and the resulting mixture was stirred at 110 °C, the reaction tube was removed after about 1 hour. Then additional **2** (13 mmol, 2.0 equiv.), and S_8 (13 mmol, 2.0 equiv.) were added at room temperature. The reaction mixture was stirred at 110 °C in an oil bath for about 8 h, then it was allowed to reach room temperature, and quenched with a saturated solution of $Na_2S_2O_3$. After diluting with 100 mL of water, the aqueous layer was extracted with ethyl acetate (3 × 100 mL), and the combined ethyl acetate layer was washed with the brine solution (1 × 100 mL). The final ethyl acetate layer was

then dried over $MgSO_4$ and concentrated under reduced pressure to get the crude product. The obtained crude product was purified using column chromatography by eluting with ethyl acetate/hexane (3:7) to afford pure 2-morpholino-1-phenyl-2-thioxoethan-1-one (**5a**) 73% yield.

8. Experimental procedure for late-stage modifications (3ad & 3ae):

i)

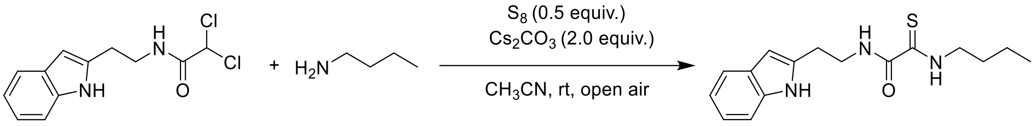

1o **Tryptamine** + **2g** → **3ad,** 42% **Natural product derivative**

A 15 mL reaction tube was charged with **(1o)** (0.2 mmol, 1.0 equiv.), S_8 (0.1 mmol, 0.5 equiv.), CS_2CO_3 (0.4 mmol, 2.0 equiv.), **2g** (0.4 mmol, 2.0 equiv.), with CH_3CN (2 mL). The resulting mixture was stirred at room temperature in the open-air atmosphere for about 12 h. After the completion of the reaction, reaction mixture was diluted with 5 mL of water. The aqueous layer was extracted with Ethyl acetate (3 × 10 mL), and the combined organic layer was washed with brine solution (1 × 5 mL). The final organic layer was then dried over $MgSO_4$ and concentrated under reduced pressure to get the crude product. The obtained crude product was purified using column chromatography by eluting with ethyl acetate/hexane (3:7) to afford *N*-(2-(1H-indol-2-yl)ethyl)-2-(butylamino)-2-thioxoacetamide **(3ad)** up to 42% yield.

ii)

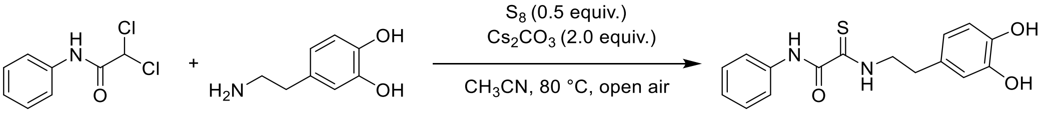

1a + **2l** **Dopamine** → **3ae,** 49% **Drug derivative**

A 15 mL reaction tube was charged with (**1a)** (0.2 mmol, 1.0 equiv.), S_8 (0.1 mmol, 0.5 equiv.), CS_2CO_3 (0.4 mmol, 2.0 equiv.), **2l** (0.4 mmol, 2.0 equiv.), with CH_3CN (2 mL). The resulting mixture was stirred at 80 °C in the open-air atmosphere for about 12 h. After the completion of the reaction, reaction mixture was diluted with 5 mL of water. The aqueous layer was extracted with Ethyl acetate (3 × 10 mL), and the combined organic layer was washed with brine solution (1 × 5 mL). The final organic layer was then dried over $MgSO_4$ and concentrated under reduced pressure to get the crude product. The obtained crude product was purified using column chromatography by eluting with ethyl acetate/hexane (3:7) to afford 2-((3,4-dihydroxyphenethyl)amino)-*N*-phenyl-2-thioxoacetamide (**3ae)** up to 49% yield.

9. Reference

(1) Jayaram, A.; Seenivasan, V. T.; Govindan, K.; Liu, Y.-M.; Chen, N.-Q.; Yeh, T.-W.; Venkatachalam, G.; Li, C.-H.; Leung, T.-F.; Lin, W.-Y. Base-promoted triple cleavage of CCl_2Br: a direct one-pot synthesis of unsymmetrical oxalamide derivatives. *Chem. Comm.* **2024**, *60*, 3079-3082.

Proton and Carbon Spectra

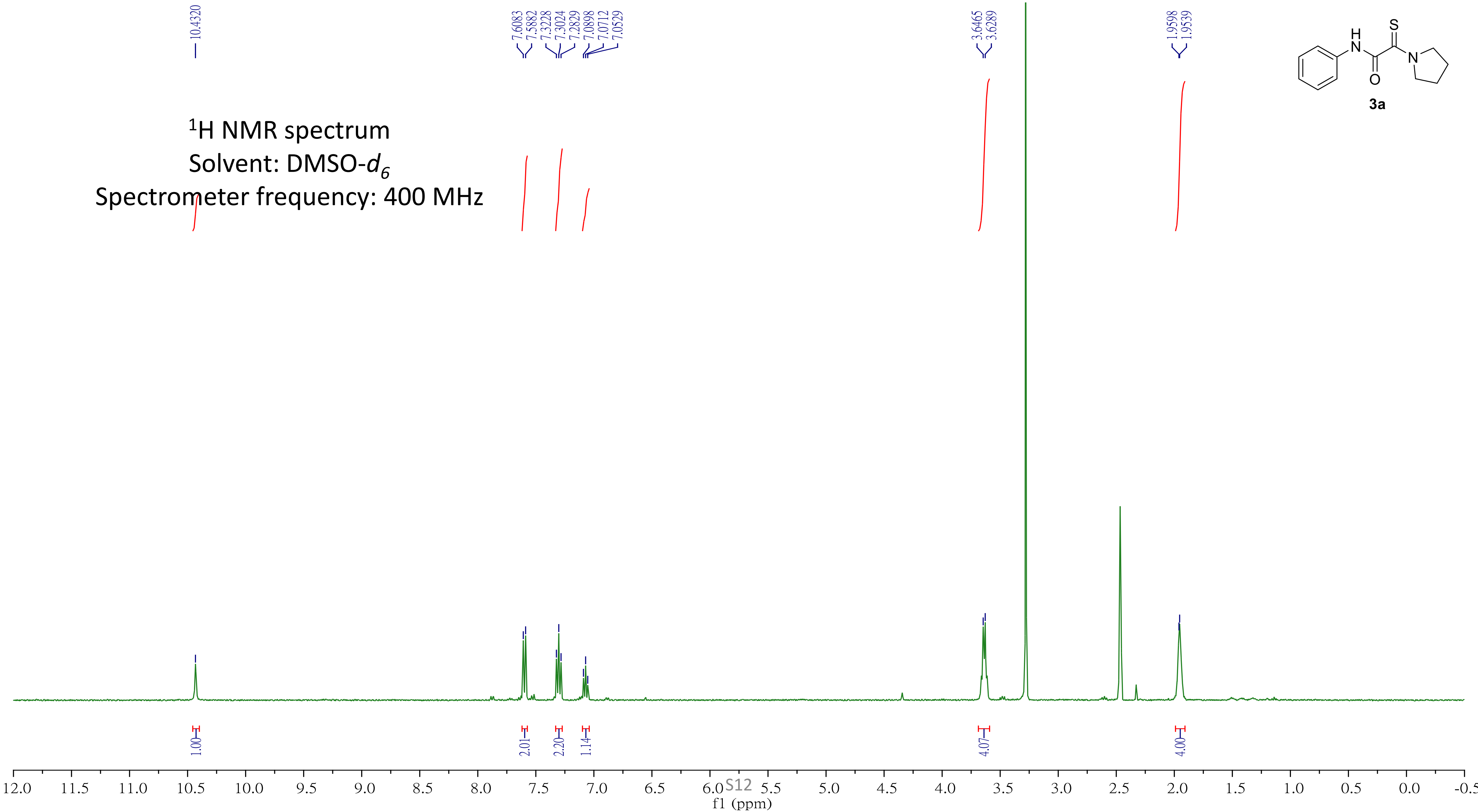

10.4320
7.6083
7.5882
7.3228
7.3024
7.2829
7.0898
7.0712
7.0529
3.6465
3.6289
1.9598
1.9539
^{1}H NMR spectrum
Solvent: DMSO-d_6
Spectrometer frequency: 400 MHz
1.00
2.01
2.20
1.14
4.07
4.00
12.0 11.5 11.0 10.5 10.0 9.5 9.0 8.5 8.0 7.5 7.0 6.5 6.0 5.5 5.0 4.5 4.0 3.5 3.0 2.5 2.0 1.5 1.0 0.5 0.0 -0.5
f1 (ppm)
H
N
S
N
O

3a

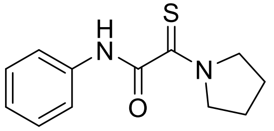

3a

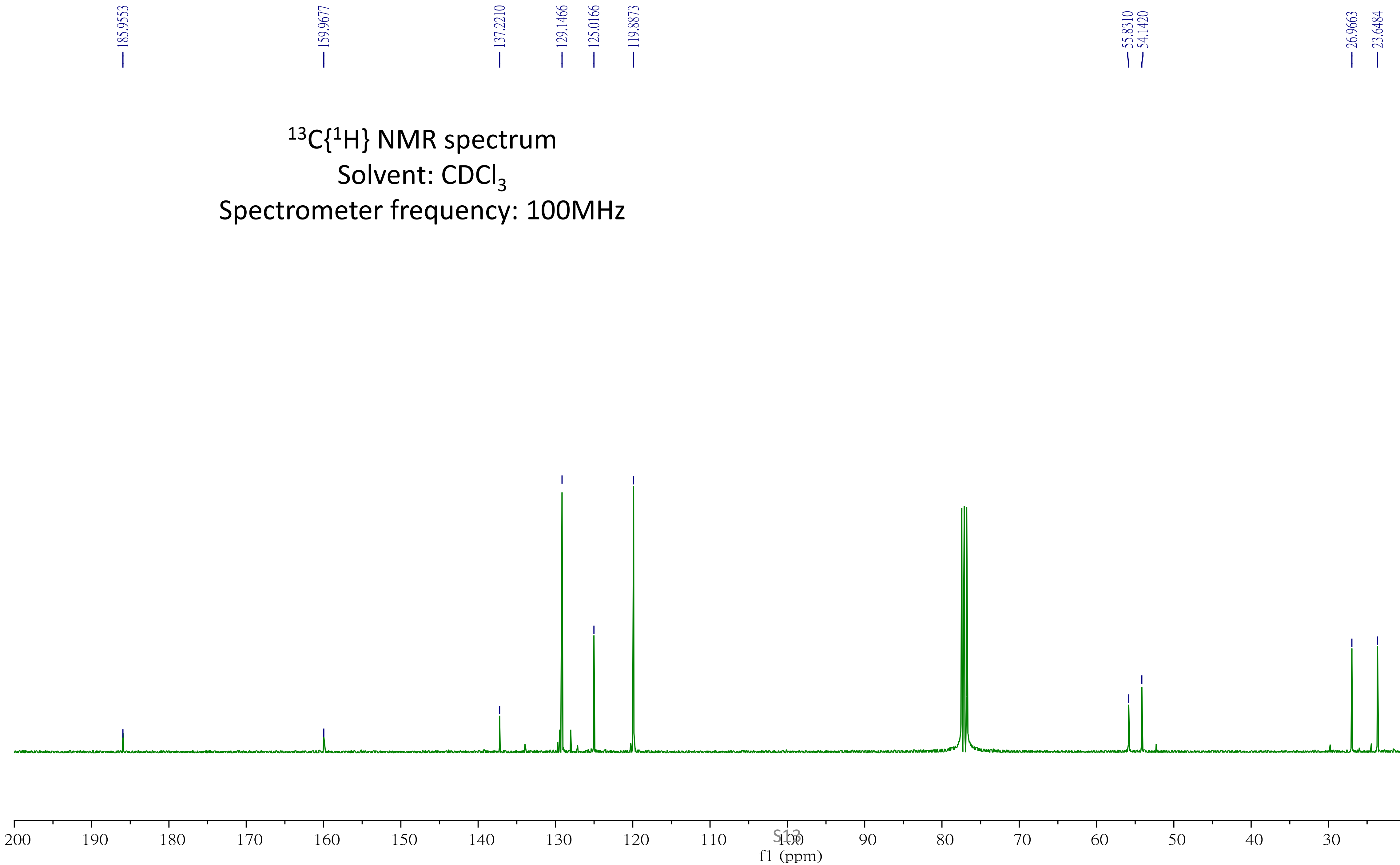

$^{13}C\{^1H\}$ NMR spectrum
Solvent: $CDCl_3$
Spectrometer frequency: 100MHz
185.9553
159.9677
137.2210
129.1466
125.0166
119.8873
55.8310
54.1420
26.9663
23.6484
200 190 180 170 160 150 140 130 120 110 100 90 80 70 60 50 40 30 20 10 0
f1 (ppm)

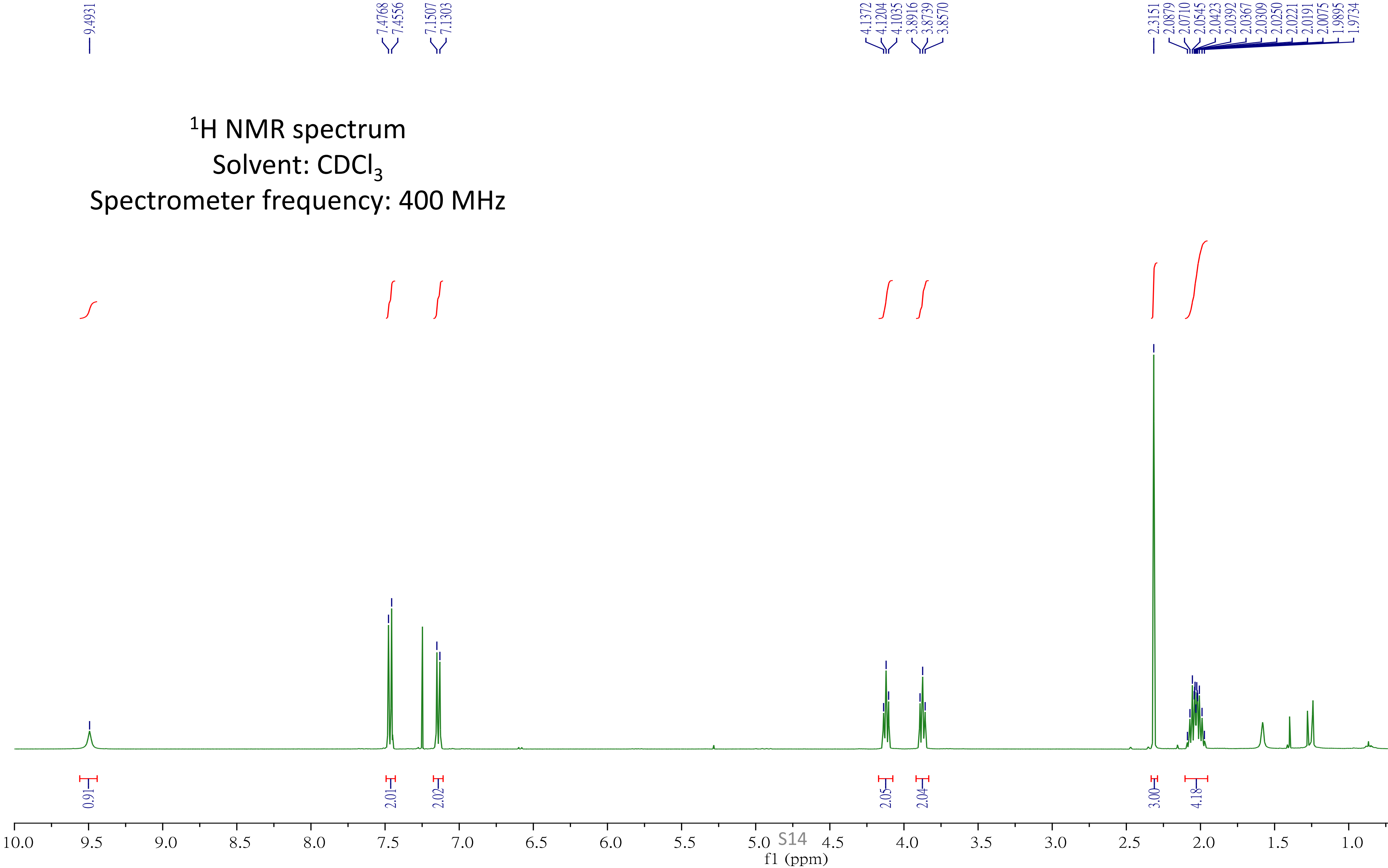

^{1}H NMR spectrum
Solvent: $CDCl_3$
Spectrometer frequency: 400 MHz
9.4931
7.4768
7.4556
7.1507
7.1303
4.1372
4.1204
4.1035
3.8916
3.8739
3.8570
2.3151
2.0879
2.0710
2.0545
2.0423
2.0392
2.0367
2.0309
2.0250
2.0221
2.0191
2.0075
1.9895
1.9734
0.91
2.01
2.02
2.05
2.04
3.00
4.18
10.0 9.5 9.0 8.5 8.0 7.5 7.0 6.5 6.0 5.5 5.0 4.5 4.0 3.5 3.0 2.5 2.0 1.5 1.0 0.5 0.0 -0.5
f1 (ppm)

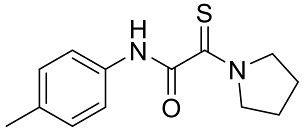

S
H
N
N
O

3b

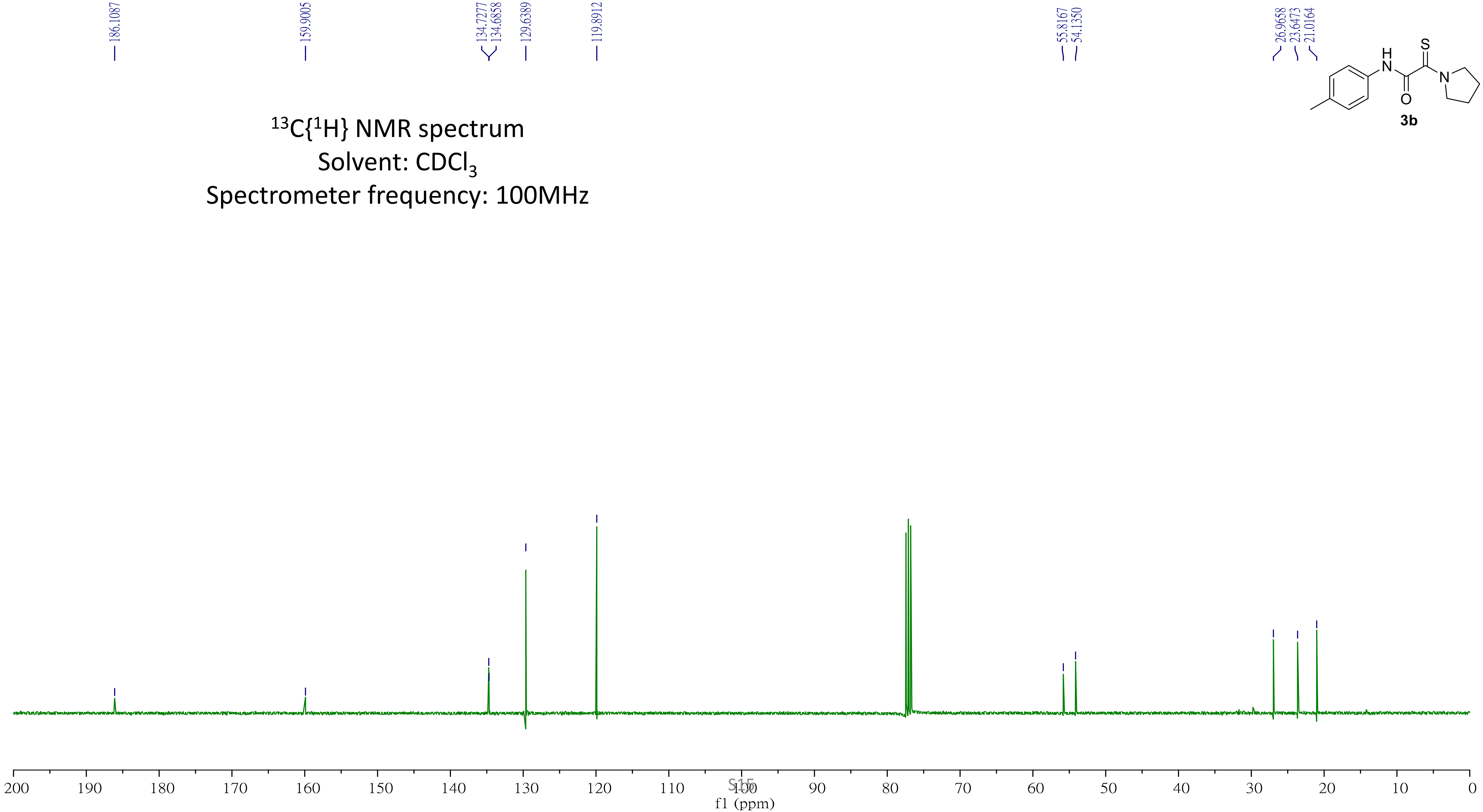
186.1087
159.9005
134.7277
134.6858
129.6389
119.8912
55.8167
54.1350
26.9658
23.6473
21.0164
H
N
S
N
O
3b
$^{13}C\{^1H\}$ NMR spectrum
Solvent: $CDCl_3$
Spectrometer frequency: 100MHz
200 190 180 170 160 150 140 130 120 110 100 90 80 70 60 50 40 30 20 10 0
f1 (ppm)

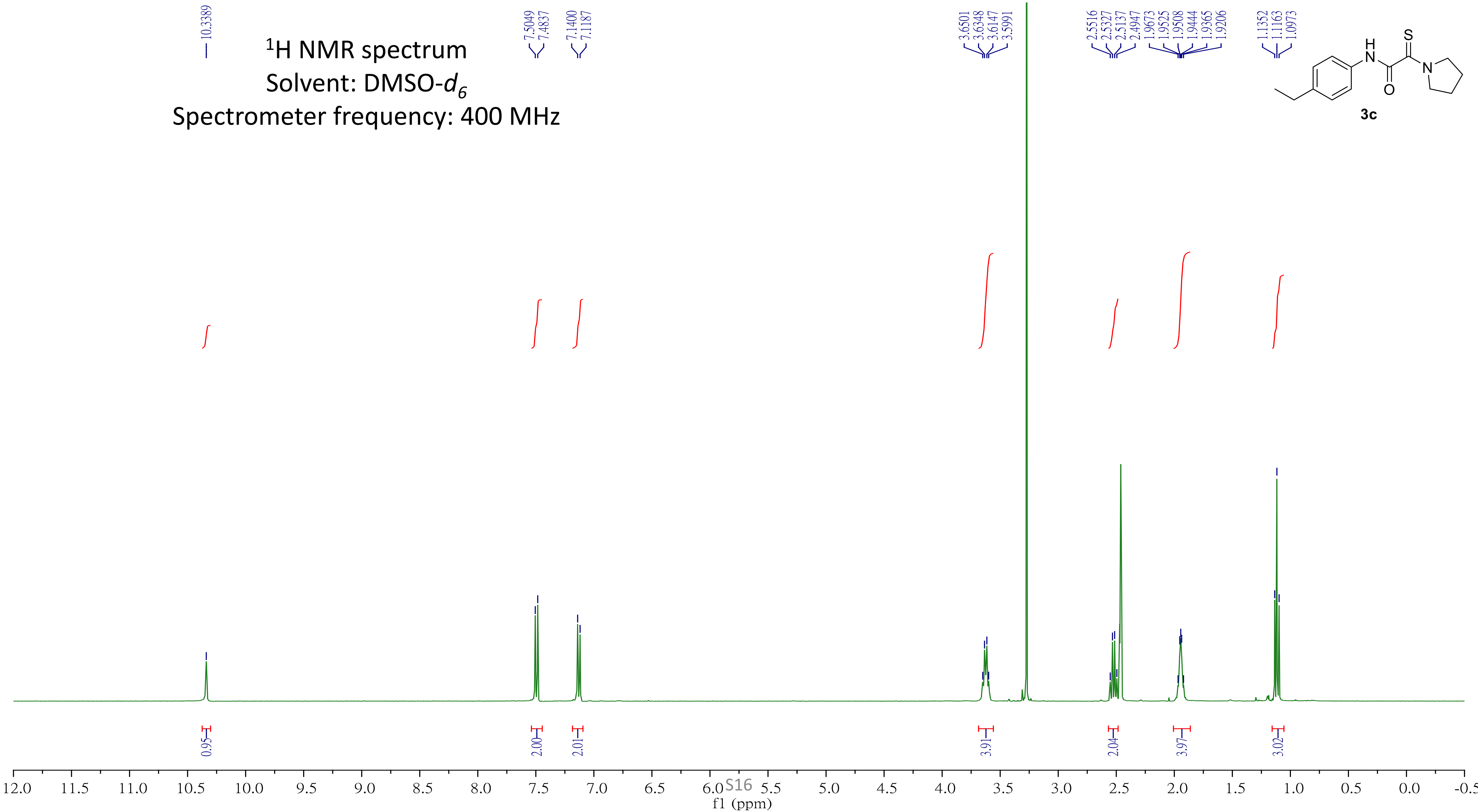
1H NMR spectrum
Solvent: DMSO-d6
Spectrometer frequency: 400 MHz
10.3389
7.5049
7.4837
7.1400
7.1187
3.6501
3.6348
3.6147
3.5991
2.5516
2.5327
2.5137
2.4947
1.9673
1.9525
1.9508
1.9444
1.9365
1.9206
1.1352
1.1163
1.0973
3c
0.95
2.00
2.01
3.91
2.04
3.97
3.02
f1 (ppm)

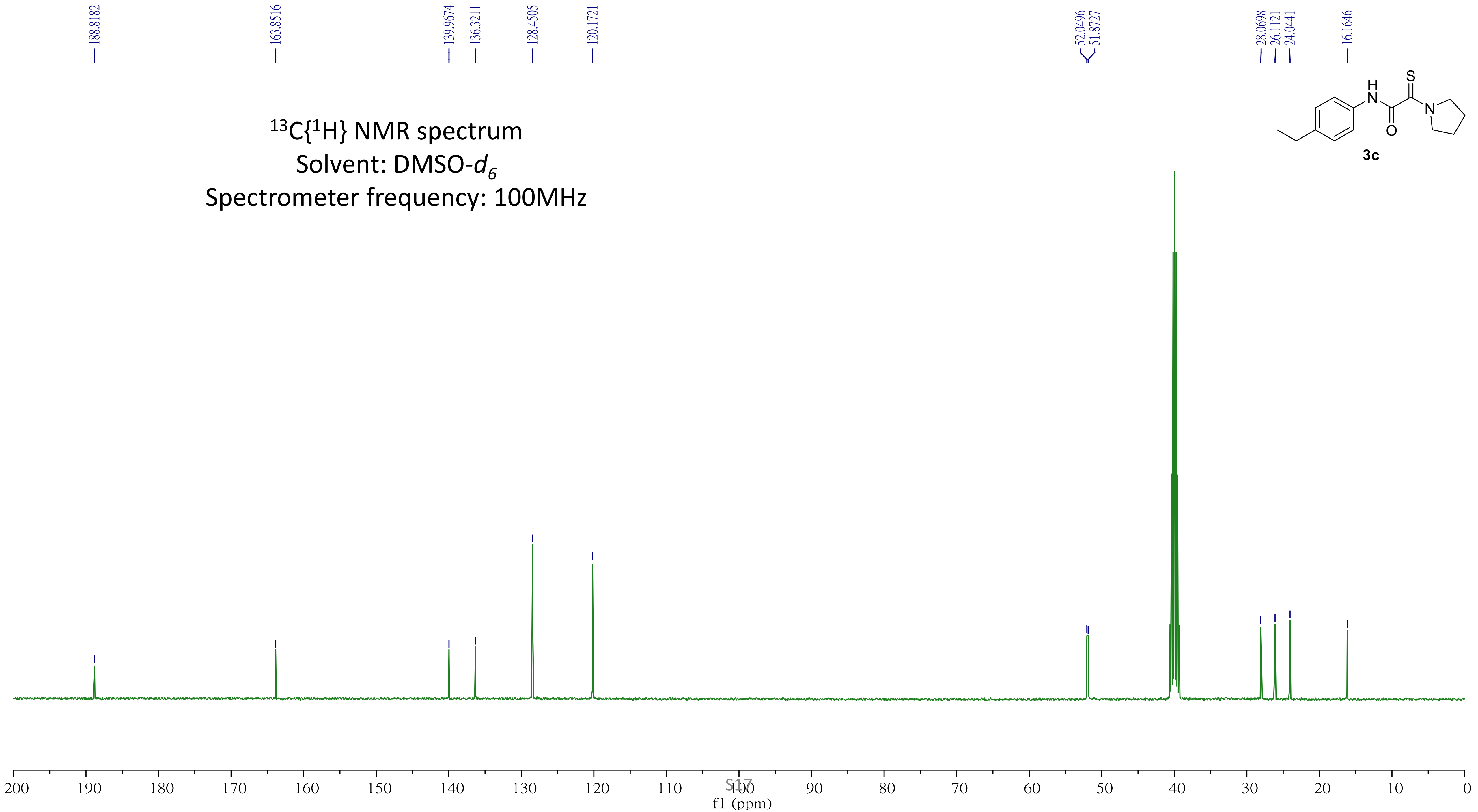
188.8182
163.8516
139.9674
136.3211
128.4505
120.1721
52.0496
51.8727
28.0698
26.1121
24.0441
16.1646
$^{13}C\{^1H\}$ NMR spectrum
Solvent: DMSO-d_6
Spectrometer frequency: 100MHz
3c
200 190 180 170 160 150 140 130 120 110 100 90 80 70 60 50 40 30 20 10 0
f1 (ppm)

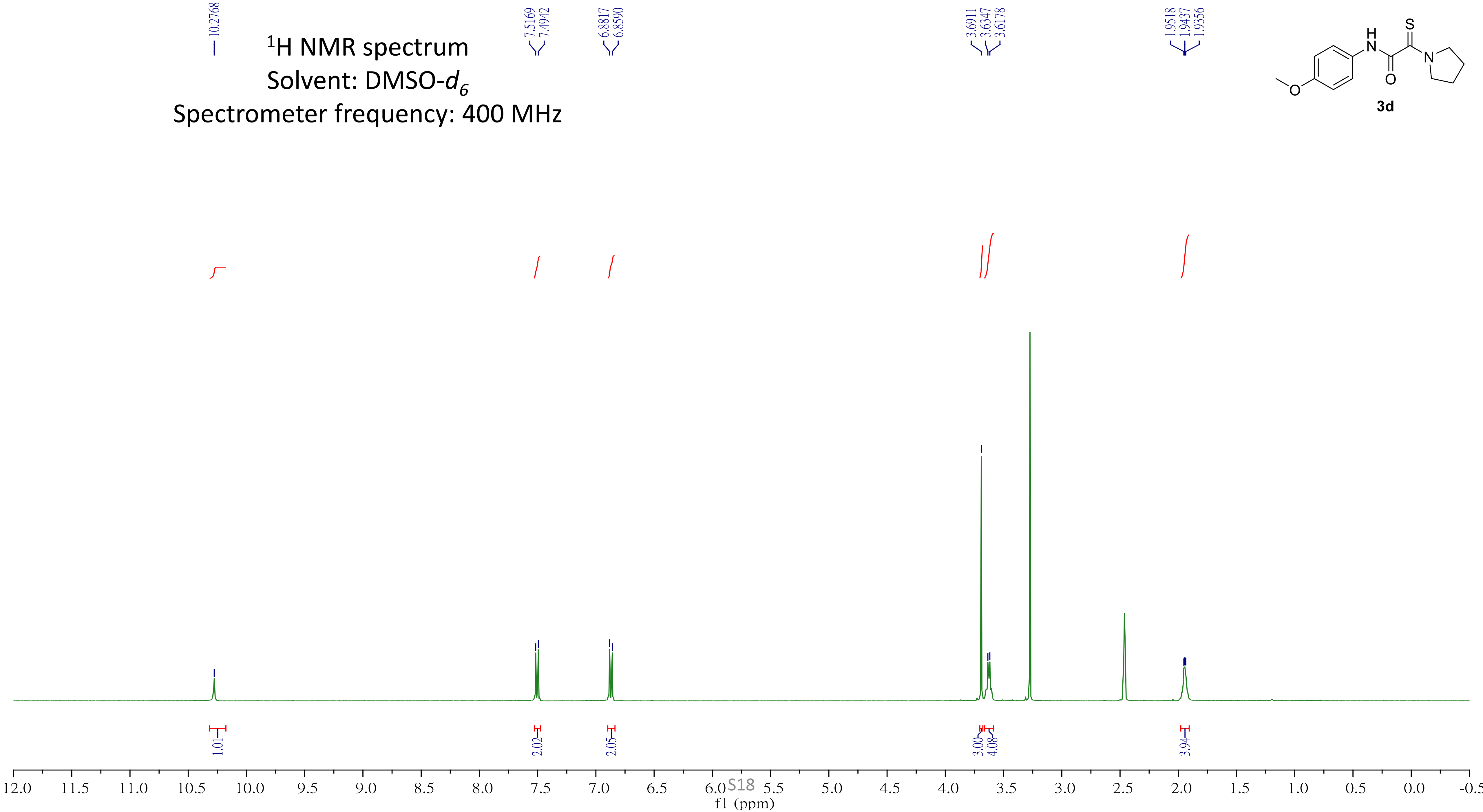

1H NMR spectrum
Solvent: DMSO-d6
Spectrometer frequency: 400 MHz
3d
10.2768
7.5169
7.4942
6.8817
6.8590
3.6911
3.6347
3.6178
1.9518
1.9437
1.9356
1.01
2.02
2.05
3.00
4.08
3.94
f1 (ppm)

$^{13}C\{^{1}H\}$ NMR spectrum
Solvent: $CDCl_3$
Spectrometer frequency: 100MHz

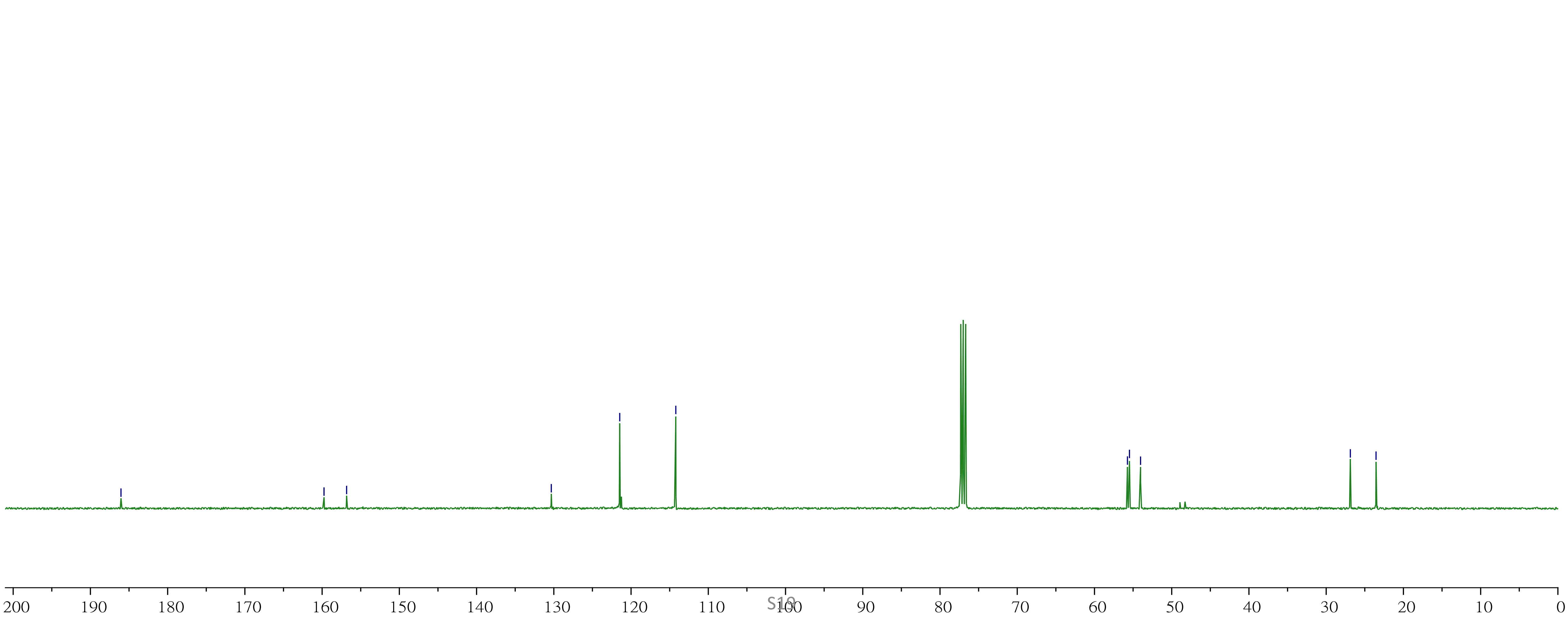

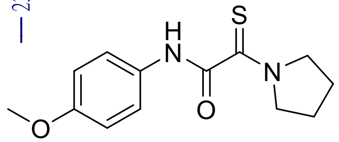

3d

^{1}H NMR spectrum
Solvent: DMSO-d_6
Spectrometer frequency: 400 MHz

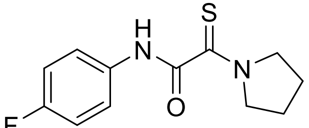

3e

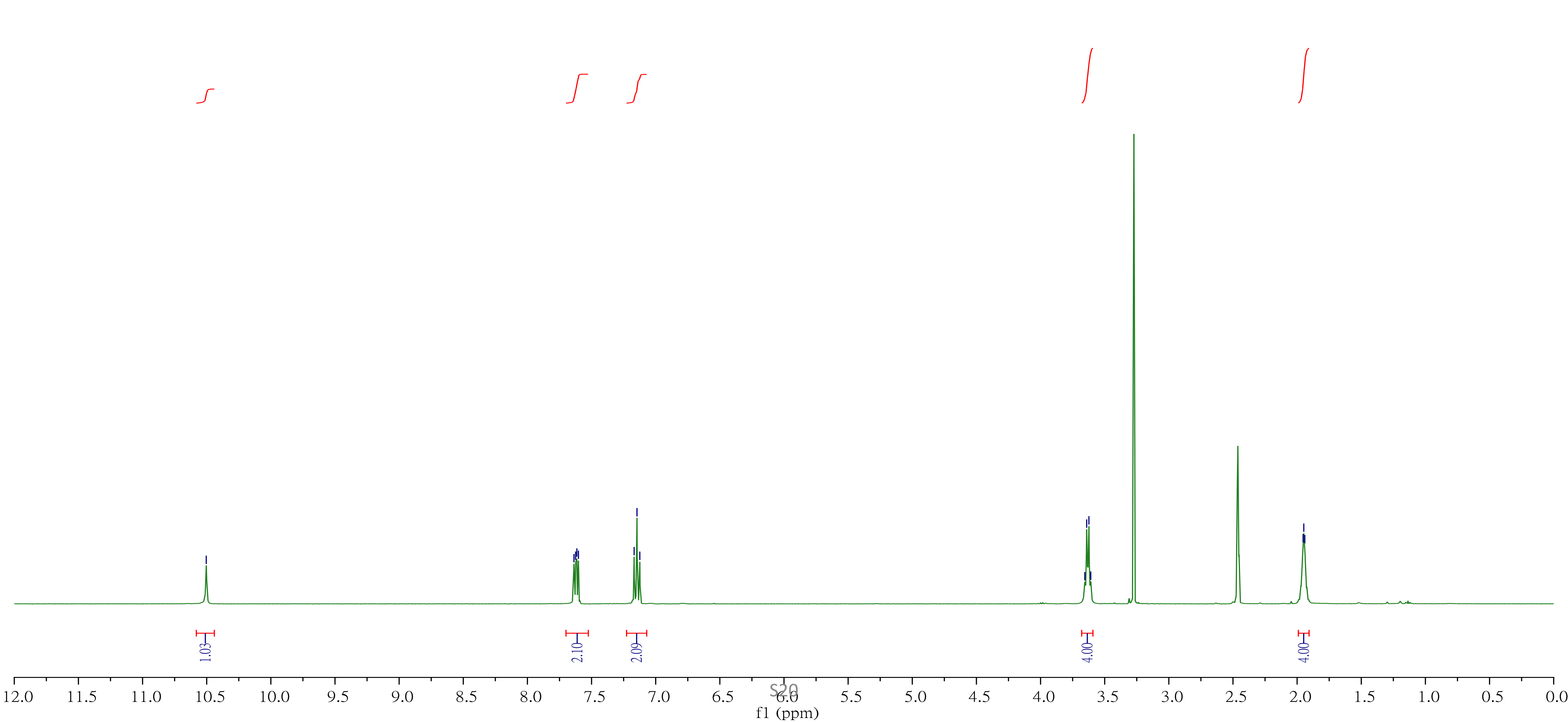

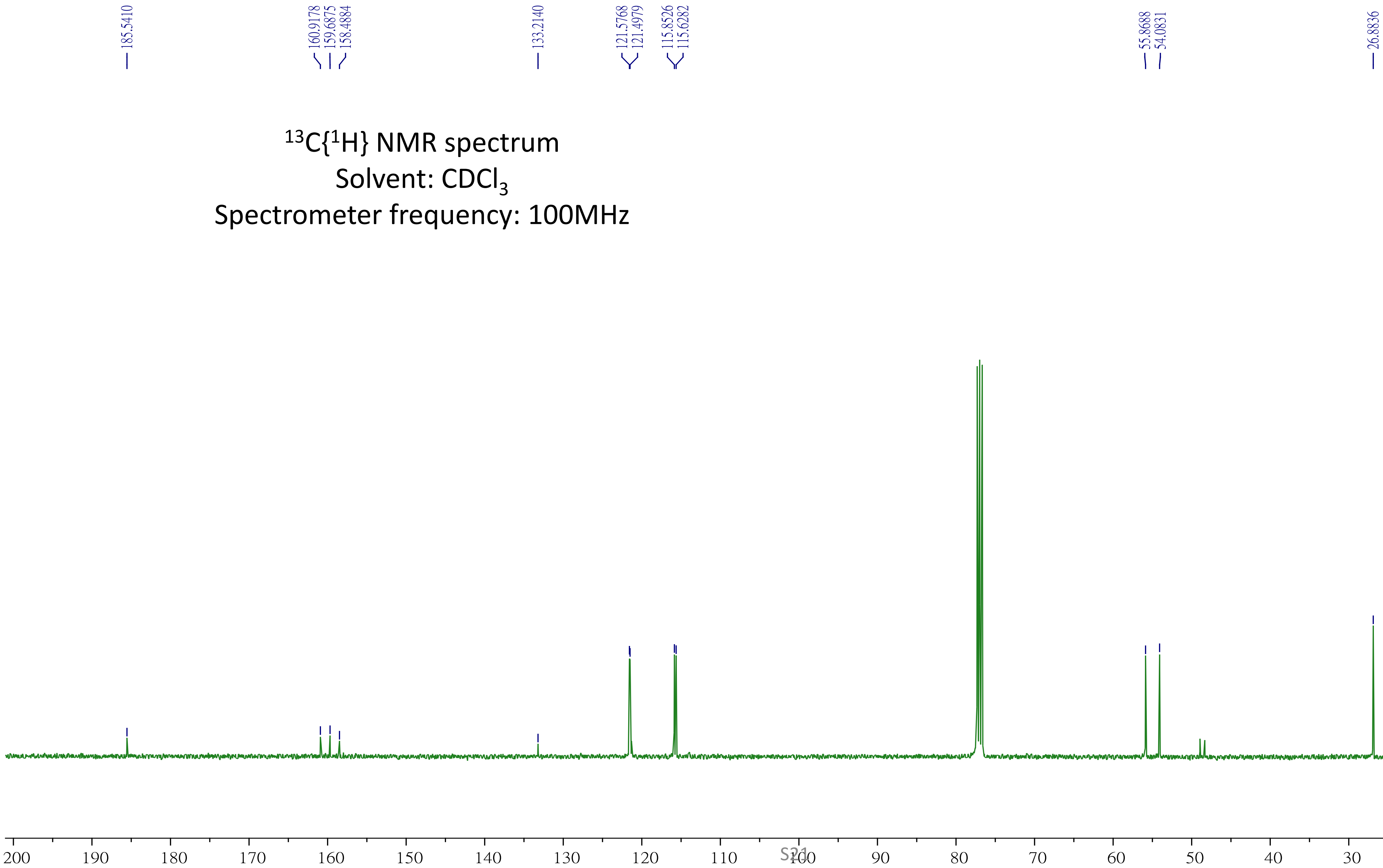
185.5410
160.9178
159.6875
158.4884
133.2140
121.5768
121.4979
115.8526
115.6282
55.8688
54.0831
26.8836
23.5197
$^{13}C\{^{1}H\}$ NMR spectrum
Solvent: $CDCl_3$
Spectrometer frequency: 100MHz
200 190 180 170 160 150 140 130 120 110 100 90 80 70 60 50 40 30 20 10 0
f1 (ppm)

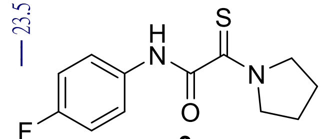
H
N
S
N
O
F

3e

$^{19}F\{^{1}H\}$ NMR spectrum
Solvent: DMSO-d_6
Spectrometer frequency: 376 MHz

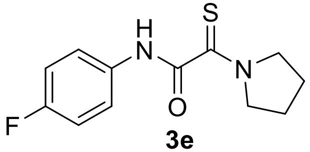

3e

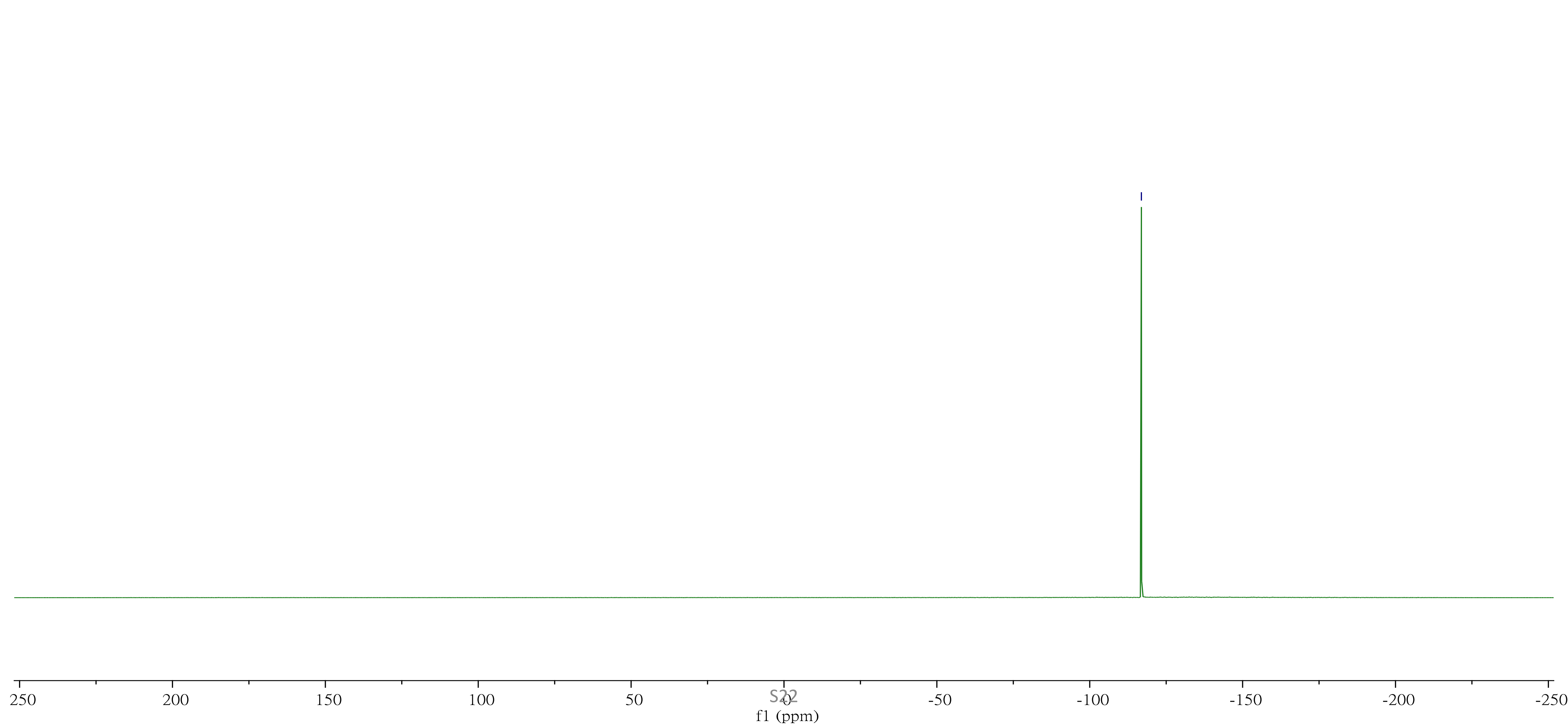

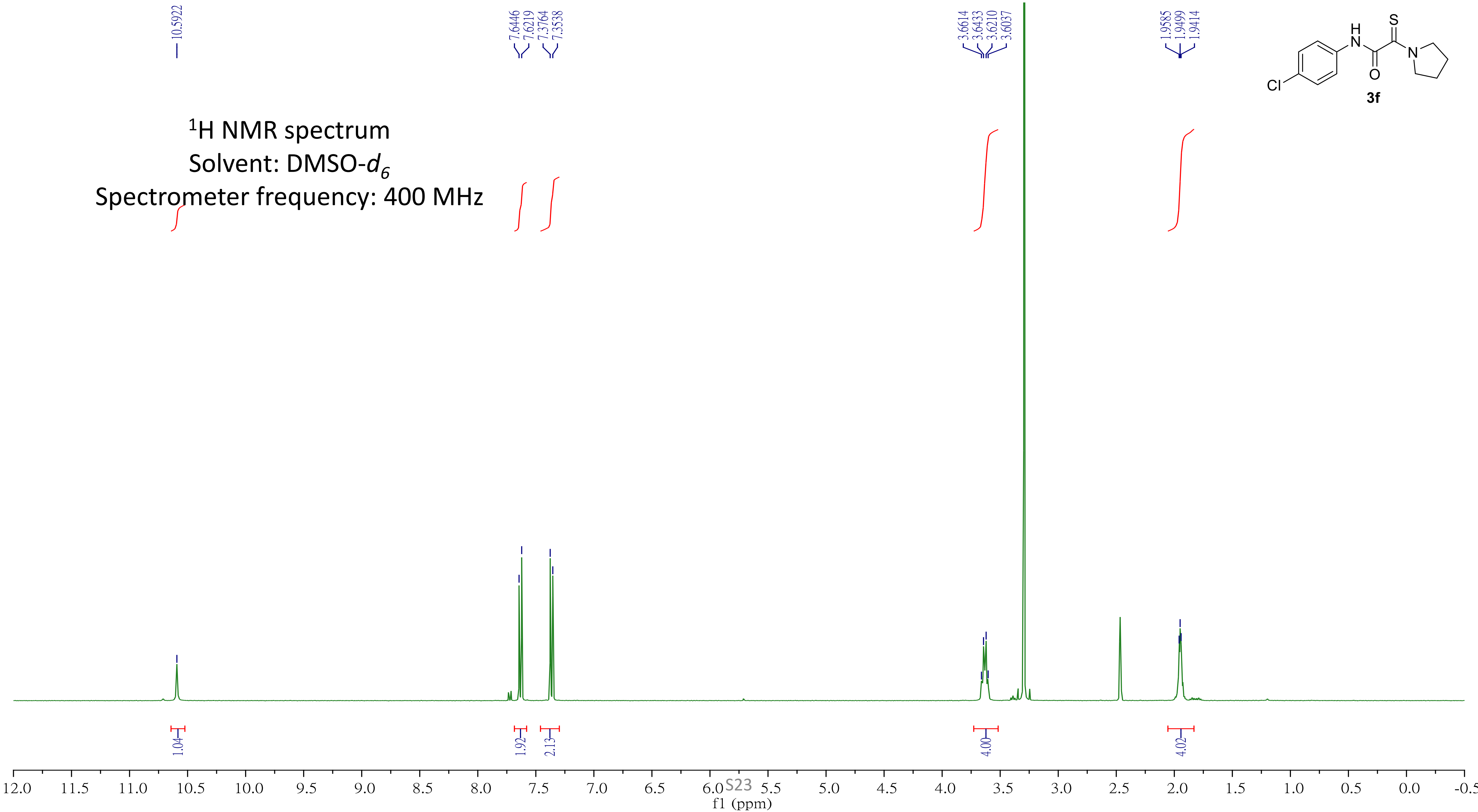

3f
^{1}H NMR spectrum
Solvent: DMSO-d_6
Spectrometer frequency: 400 MHz
10.5922
7.6446
7.6219
7.3764
7.3538
3.6614
3.6433
3.6210
3.6037
1.9585
1.9499
1.9414
1.04
1.92
2.13
4.00
4.02
f1 (ppm)

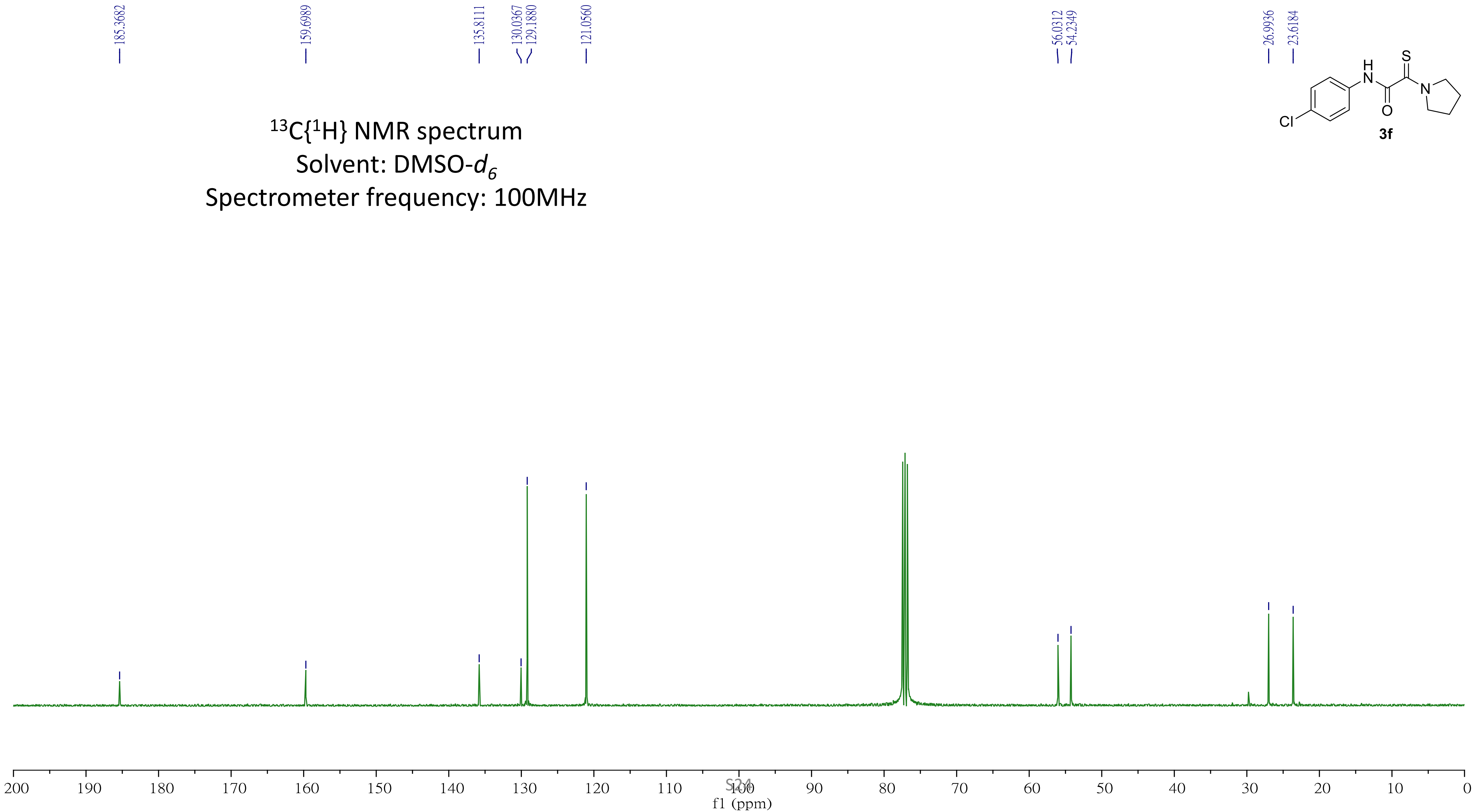
185.3682
159.6989
135.8111
130.0367
129.1880
121.0560
56.0312
54.2349
26.9936
23.6184
$^{13}C\{^1H\}$ NMR spectrum
Solvent: DMSO-d_6
Spectrometer frequency: 100MHz
3f
200 190 180 170 160 150 140 130 120 110 100 90 80 70 60 50 40 30 20 10 0
f1 (ppm)

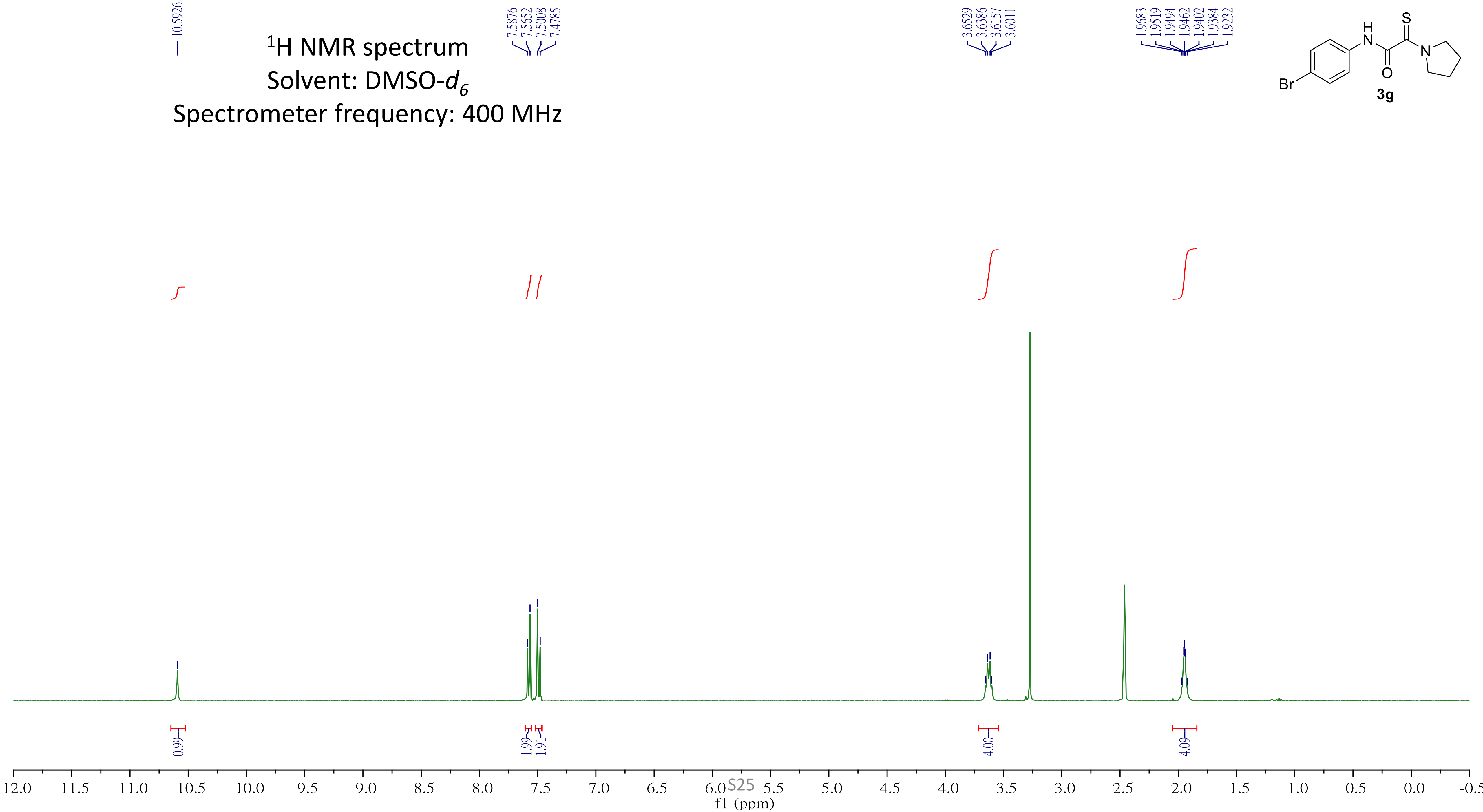

10.5926
7.5876
7.5652
7.5008
7.4785
3.6529
3.6386
3.6157
3.6011
1.9683
1.9519
1.9494
1.9462
1.9402
1.9384
1.9232
^{1}H NMR spectrum
Solvent: DMSO-d_6
Spectrometer frequency: 400 MHz
H
N
S
N
O
Br
3g
0.99
1.99
1.91
4.00
4.09
12.0
11.5
11.0
10.5
10.0
9.5
9.0
8.5
8.0
7.5
7.0
6.5
6.0
5.5
5.0
4.5
4.0
3.5
3.0
2.5
2.0
1.5
1.0
0.5
0.0
-0.5
f1 (ppm)

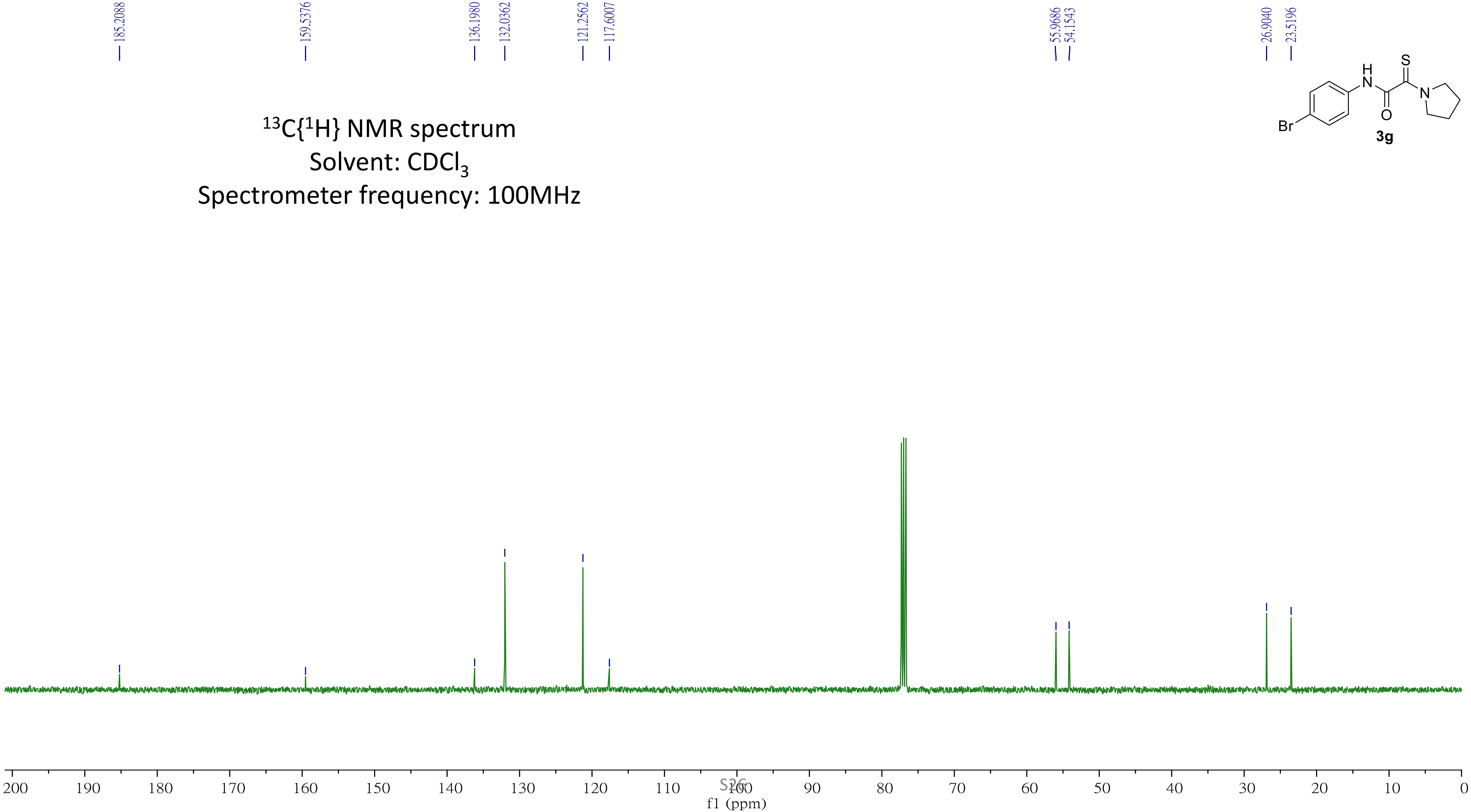
185.2088
159.5376
136.1980
132.0362
121.2562
117.6007
55.9686
54.1543
26.9040
23.5196
$^{13}C\{^{1}H\}$ NMR spectrum
Solvent: $CDCl_3$
Spectrometer frequency: 100MHz
H
N
S
N
O
Br
3g
200 190 180 170 160 150 140 130 120 110 100 90 80 70 60 50 40 30 20 10 0
f1 (ppm)

^{1}H NMR spectrum
Solvent: $CDCl_3$
Spectrometer frequency: 400 MHz

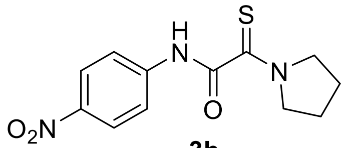

3h

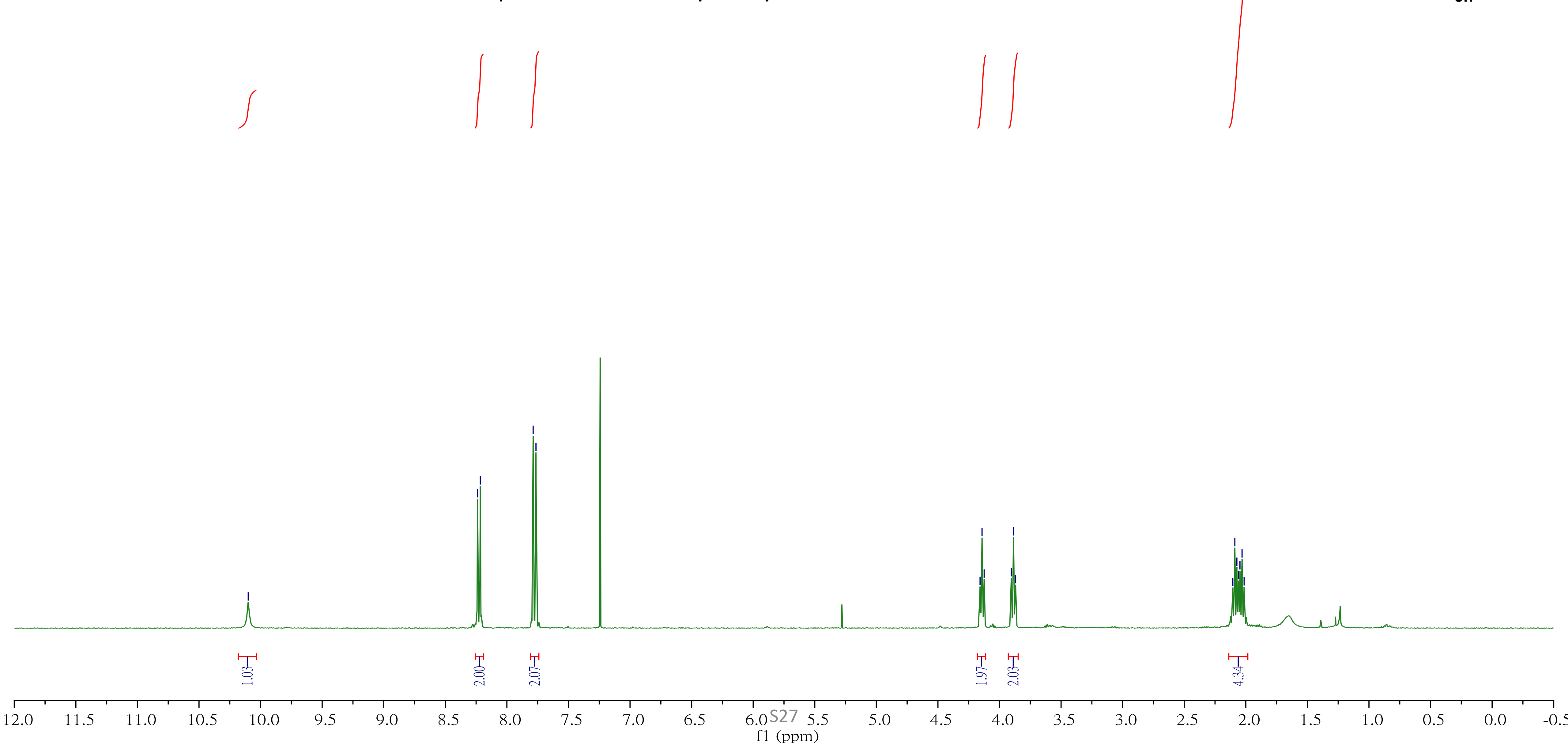

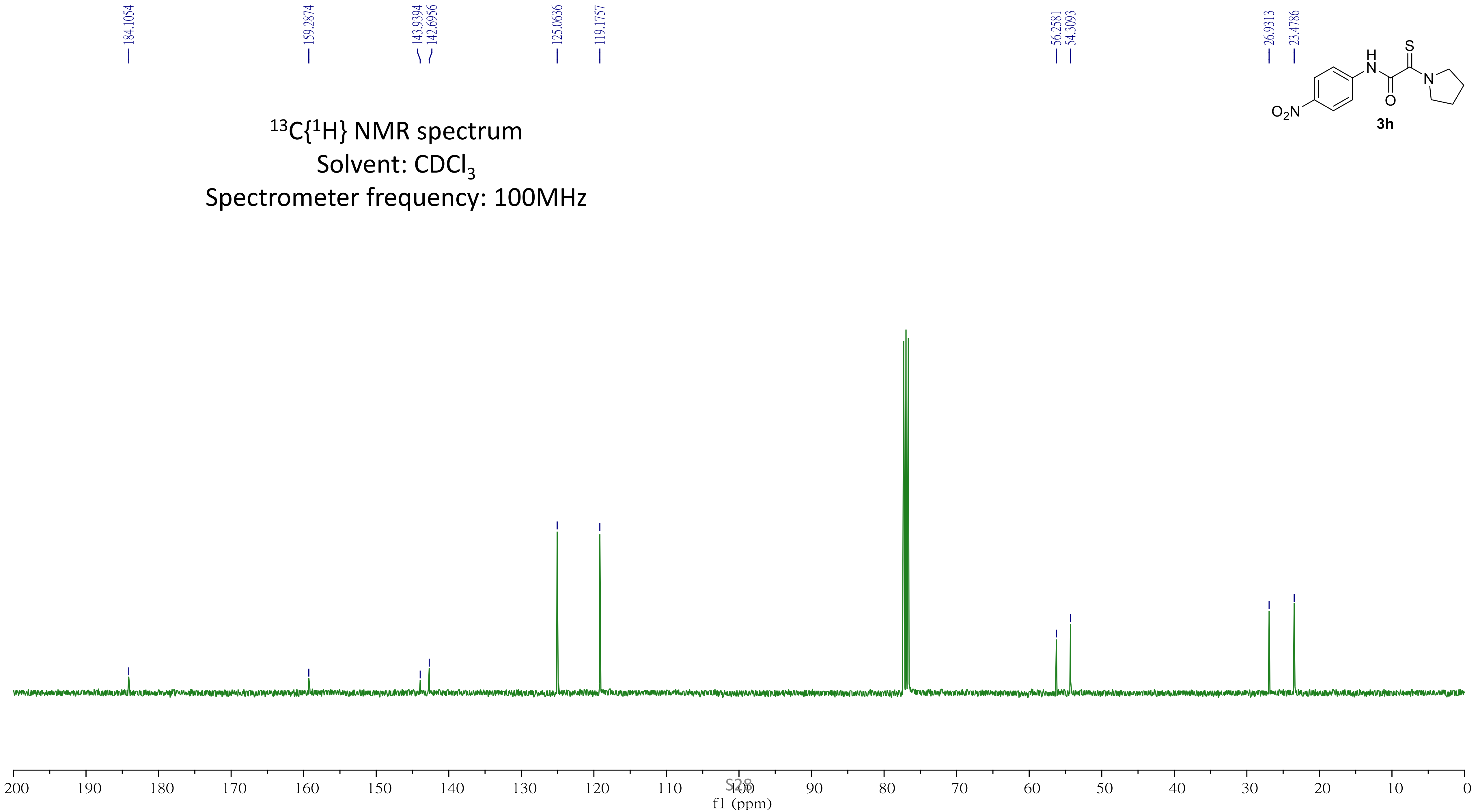
184.1054
159.2874
143.9394
142.6956
125.0636
119.1757
56.2581
54.3093
26.9313
23.4786
13C{1H} NMR spectrum
Solvent: CDCl3
Spectrometer frequency: 100MHz
3h
200 190 180 170 160 150 140 130 120 110 100 90 80 70 60 50 40 30 20 10 0
f1 (ppm)

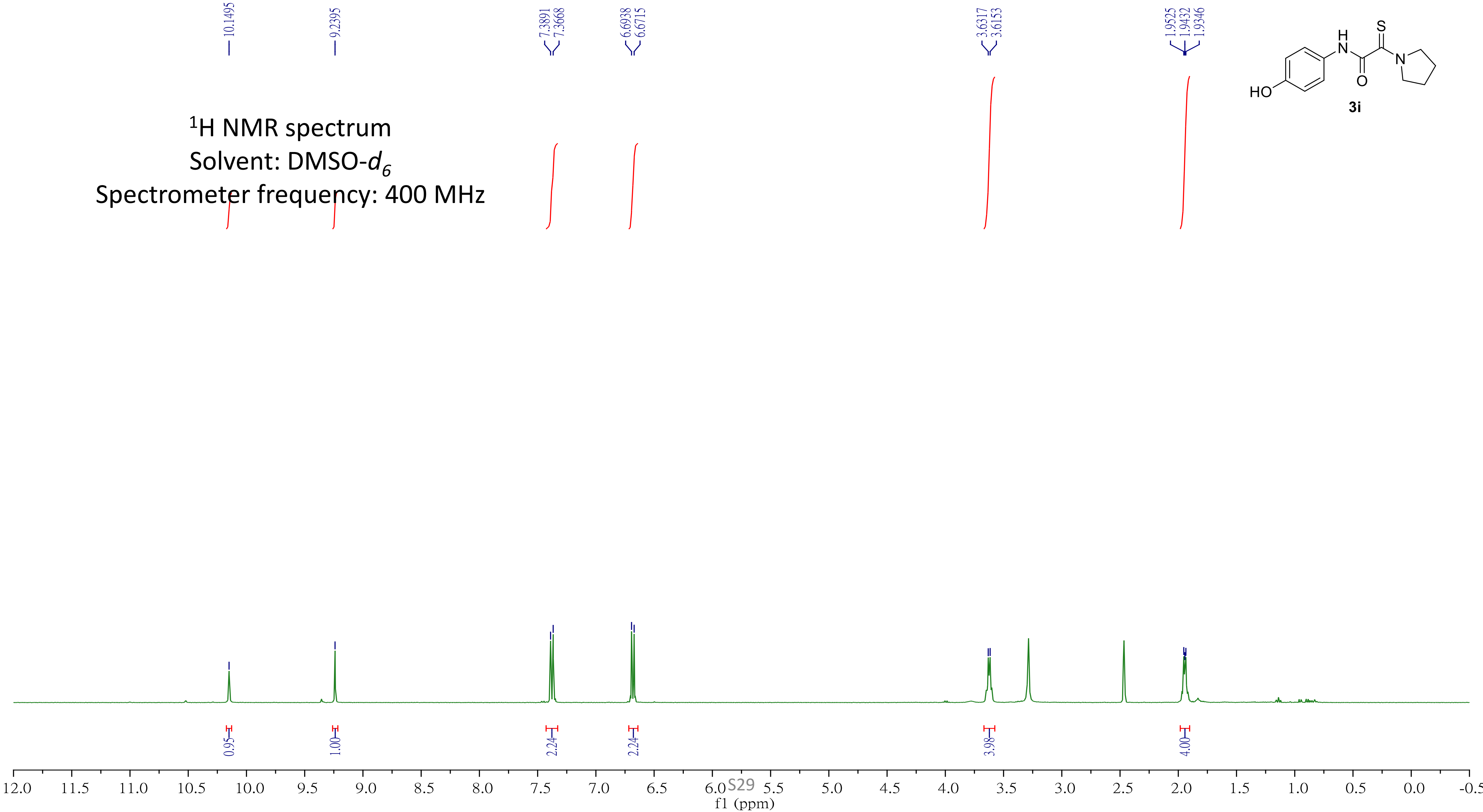

1H NMR spectrum
Solvent: DMSO-d6
Spectrometer frequency: 400 MHz
10.1495
9.2395
7.3891
7.3668
6.6938
6.6715
3.6317
3.6153
1.9525
1.9432
1.9346
HO
H
N
S
N
O
3i
0.95
1.00
2.24
2.24
3.98
4.00
12.0 11.5 11.0 10.5 10.0 9.5 9.0 8.5 8.0 7.5 7.0 6.5 6.0 5.5 5.0 4.5 4.0 3.5 3.0 2.5 2.0 1.5 1.0 0.5 0.0 -0.5
f1 (ppm)

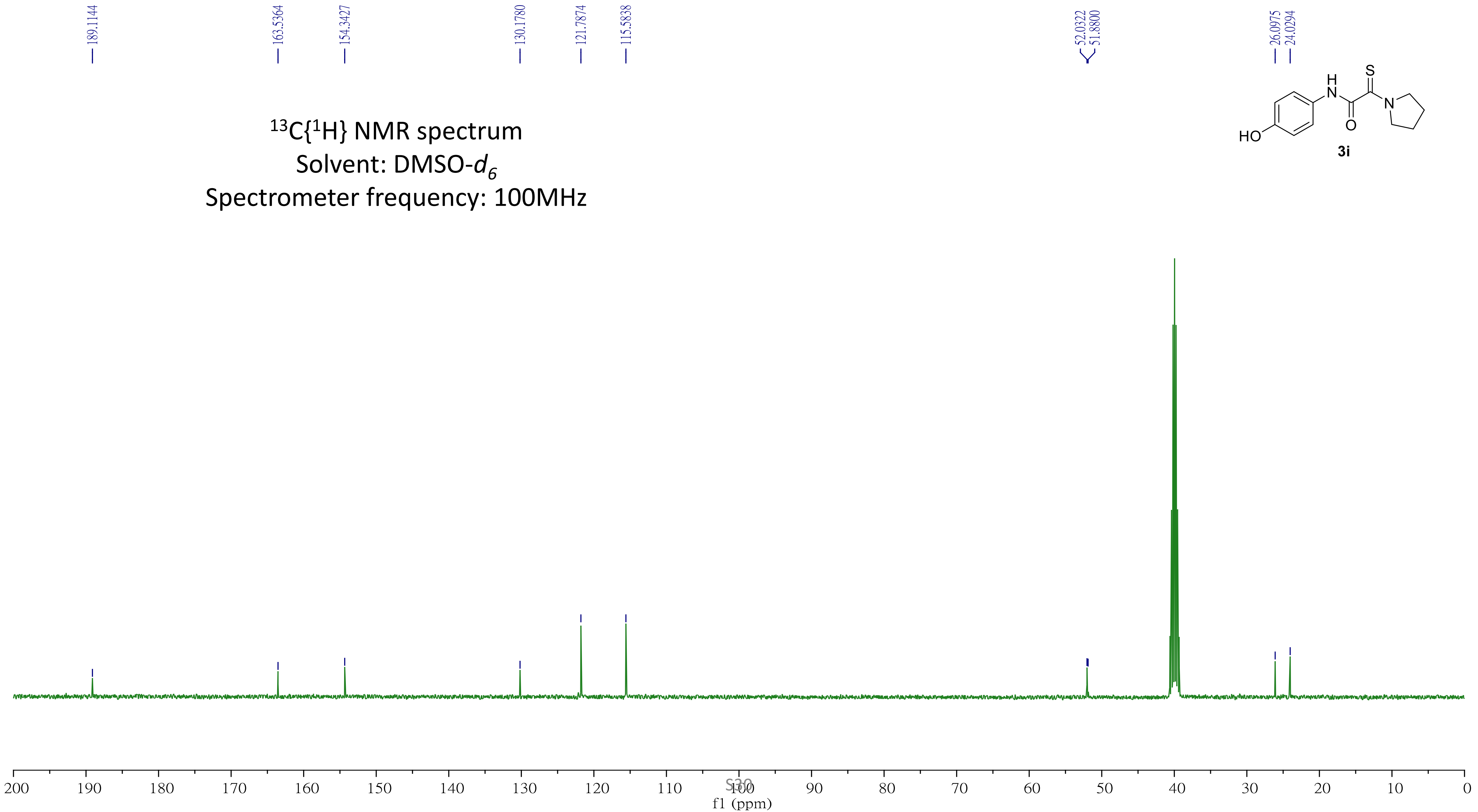

189.1144
163.5364
154.3427
130.1780
121.7874
115.5838
52.0322
51.8800
26.0975
24.0294
$^{13}C\{^{1}H\}$ NMR spectrum
Solvent: DMSO-d_6
Spectrometer frequency: 100MHz
H
N
S
O
N
HO
3i
200 190 180 170 160 150 140 130 120 110 100 90 80 70 60 50 40 30 20 10 0
f1 (ppm)

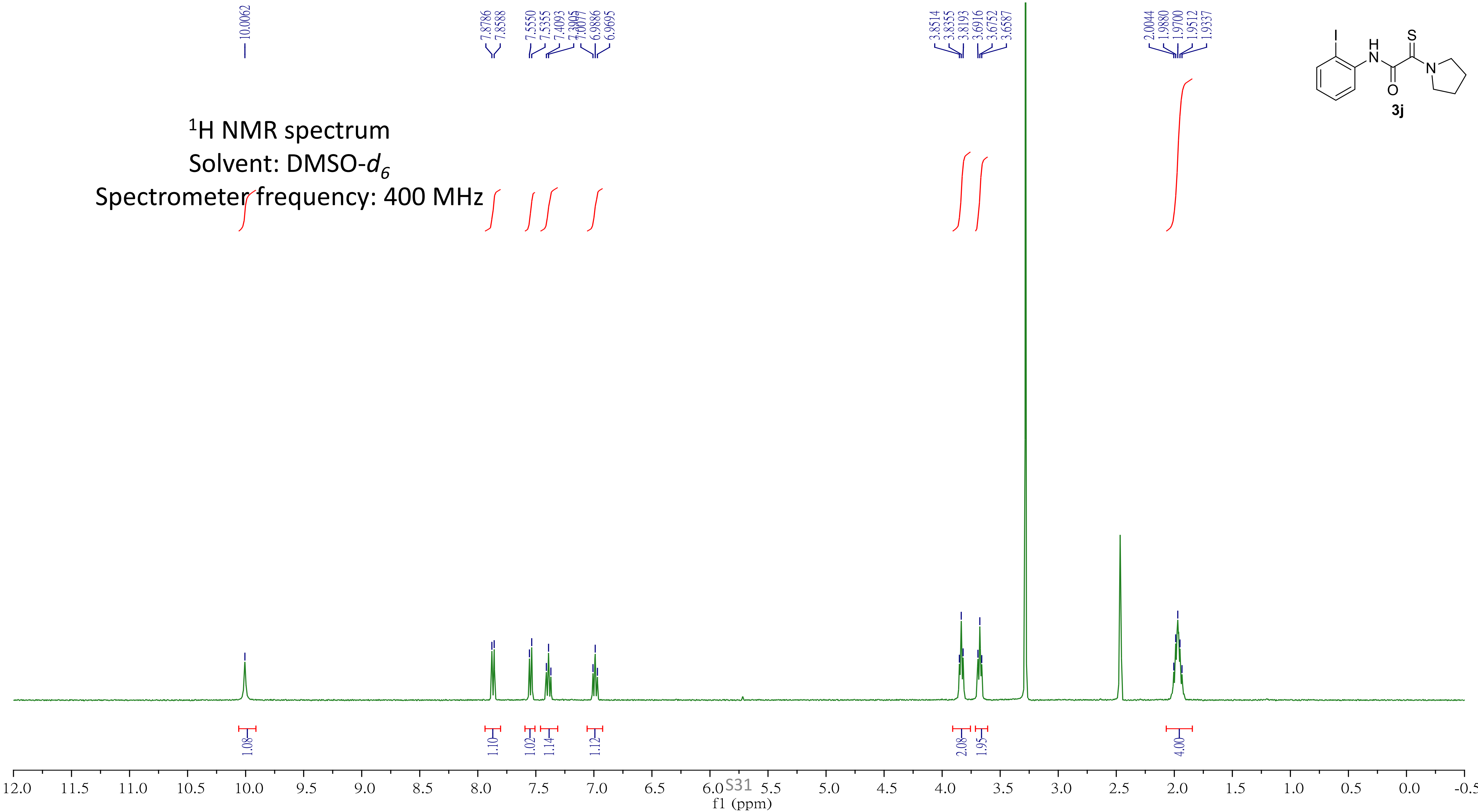

¹H NMR spectrum
Solvent: DMSO-d_6
Spectrometer frequency: 400 MHz
3j
f1 (ppm)

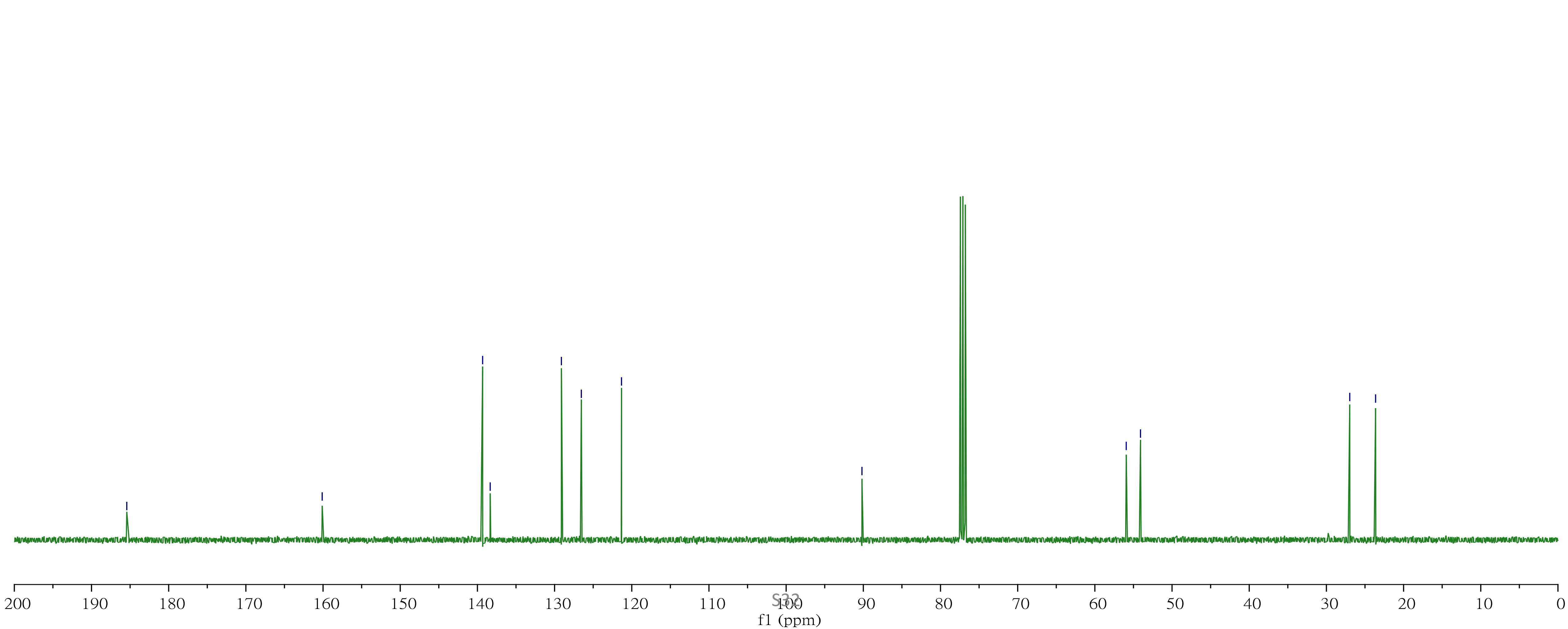

$^{13}C\{^{1}H\}$ NMR spectrum
Solvent: $CDCl_3$
Spectrometer frequency: 100MHz

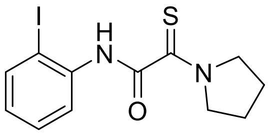

3j

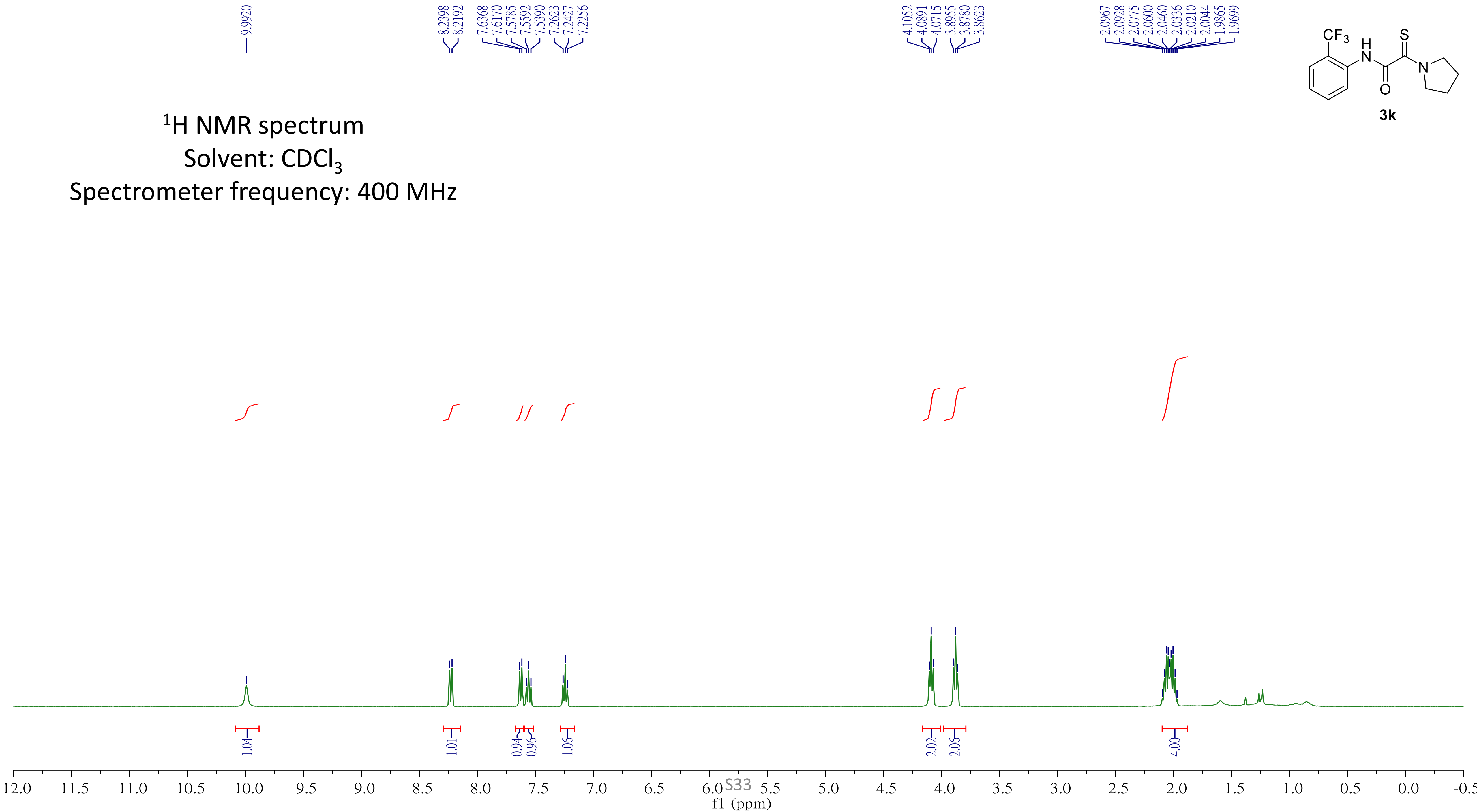

^{1}H NMR spectrum
Solvent: $CDCl_3$
Spectrometer frequency: 400 MHz
3k
f1 (ppm)

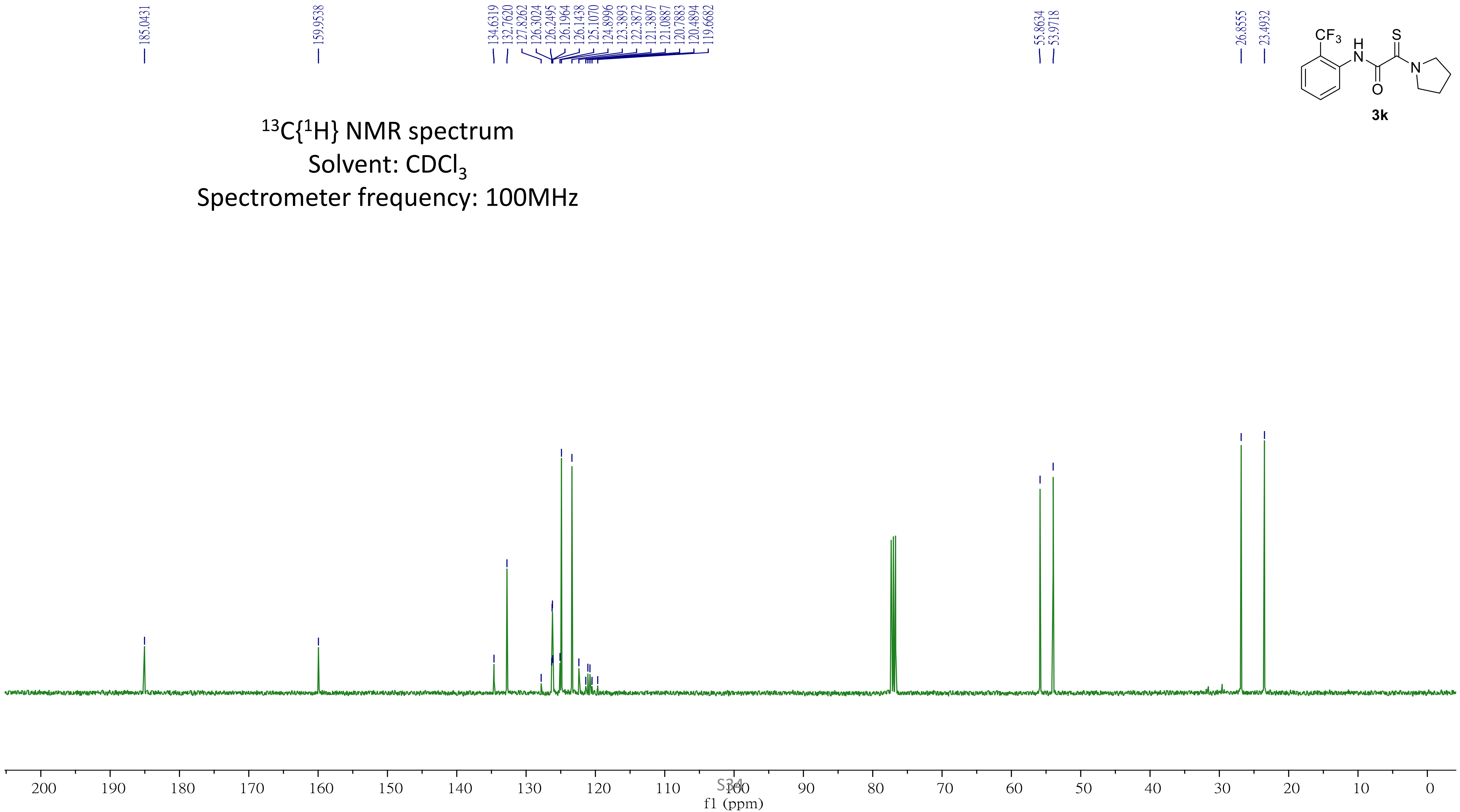

185.0431
159.9538
134.6319
132.7620
127.8262
126.3024
126.2495
126.1964
126.1438
125.1070
124.8996
123.3893
122.3872
121.3897
121.0887
120.7883
120.4894
119.6682
55.8634
53.9718
26.8555
23.4932
CF3
H
N
S
N
O
3k
13C{1H} NMR spectrum
Solvent: CDCl3
Spectrometer frequency: 100MHz
200 190 180 170 160 150 140 130 120 110 100 90 80 70 60 50 40 30 20 10 0
f1 (ppm)

$^{19}F\{^1H\}$ NMR spectrum
Solvent: DMSO-d_6
Spectrometer frequency: 376 MHz

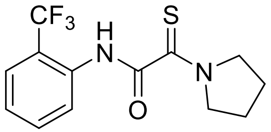

3k

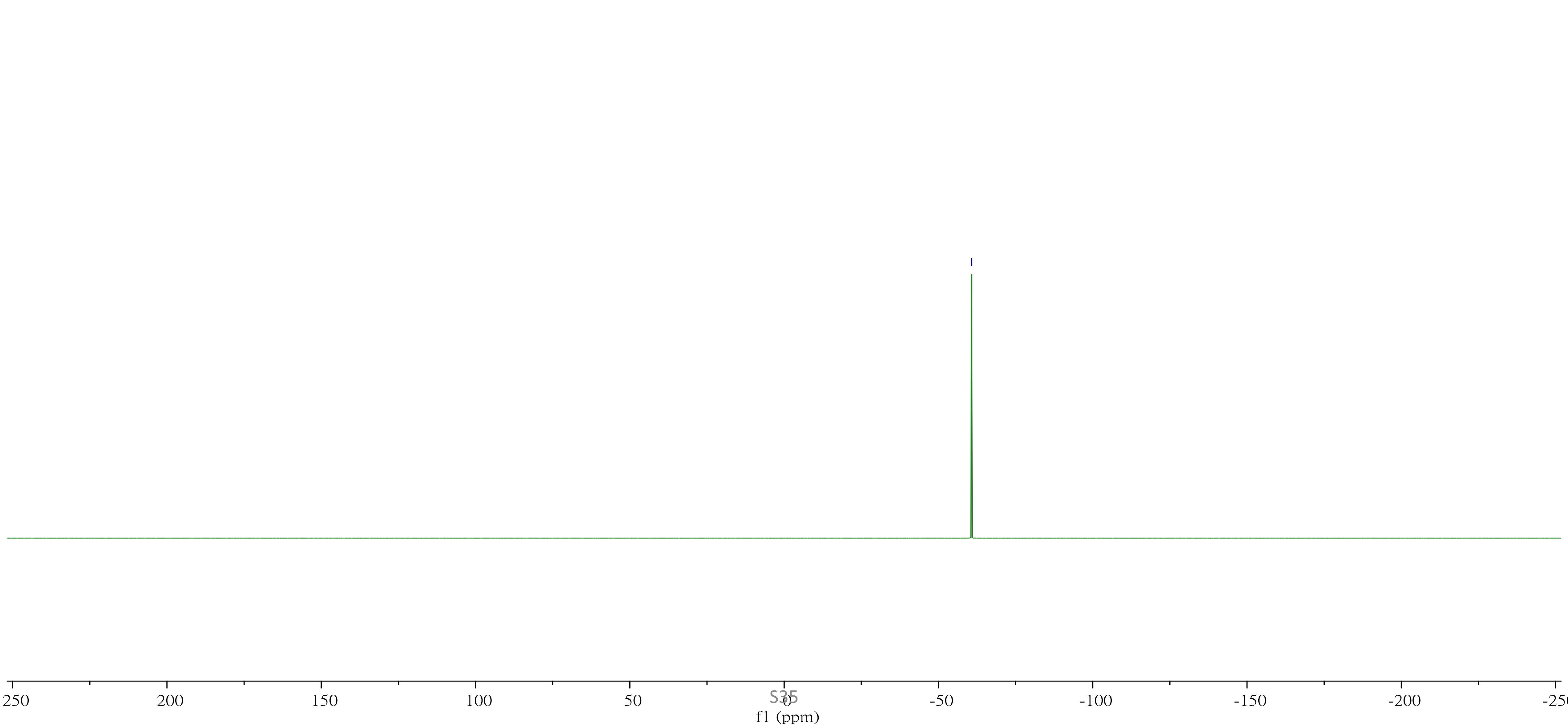

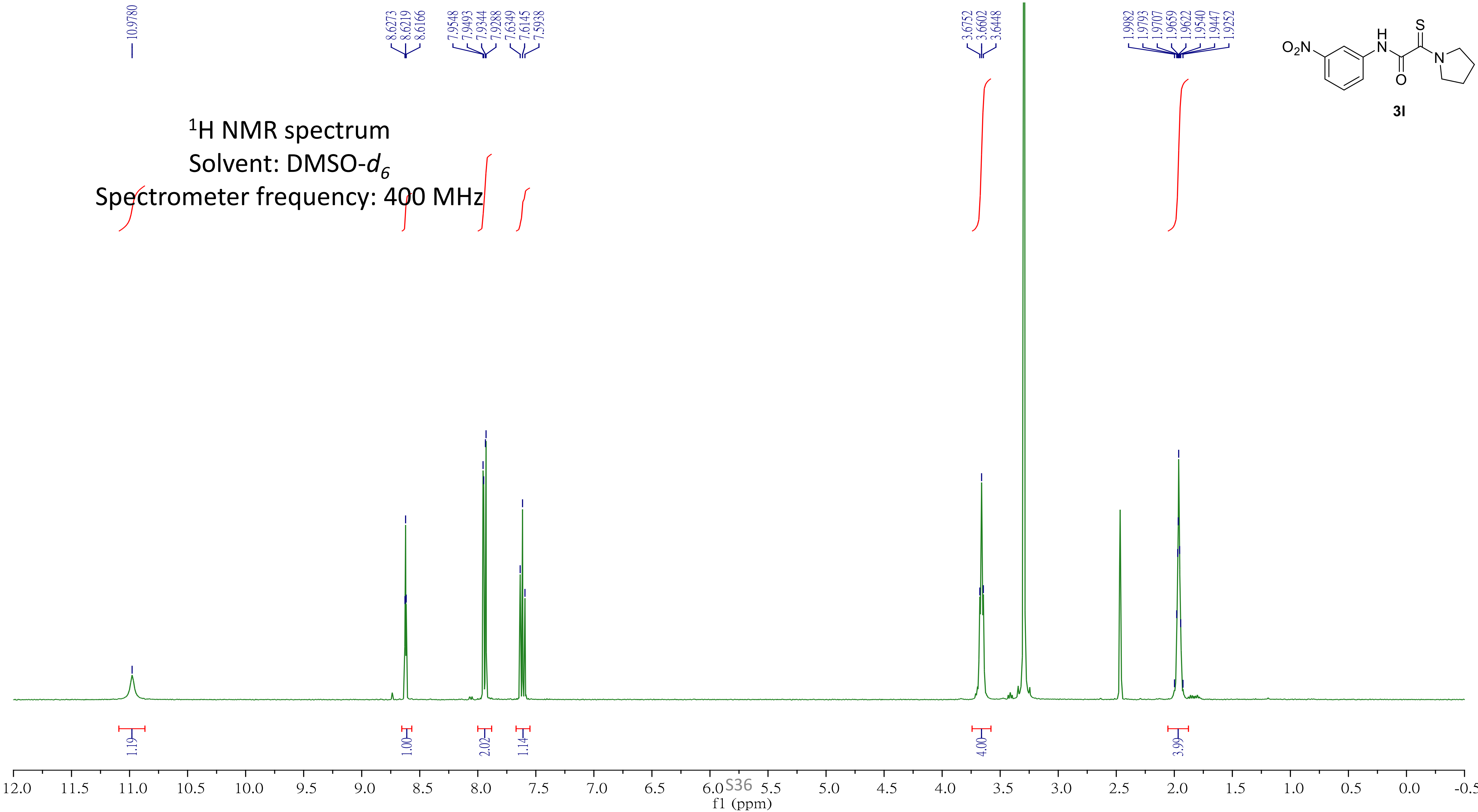

^{1}H NMR spectrum
Solvent: DMSO-d_6
Spectrometer frequency: 400 MHz
10.9780
8.6273
8.6219
8.6166
7.9548
7.9493
7.9344
7.9288
7.6349
7.6145
7.5938
3.6752
3.6602
3.6448
1.9982
1.9793
1.9707
1.9659
1.9622
1.9540
1.9447
1.9252
O_2N
H
N
S
N
O
3l
1.19
1.00
2.02
1.14
4.00
3.99
12.0 11.5 11.0 10.5 10.0 9.5 9.0 8.5 8.0 7.5 7.0 6.5 6.0 5.5 5.0 4.5 4.0 3.5 3.0 2.5 2.0 1.5 1.0 0.5 0.0 -0.5
f1 (ppm)

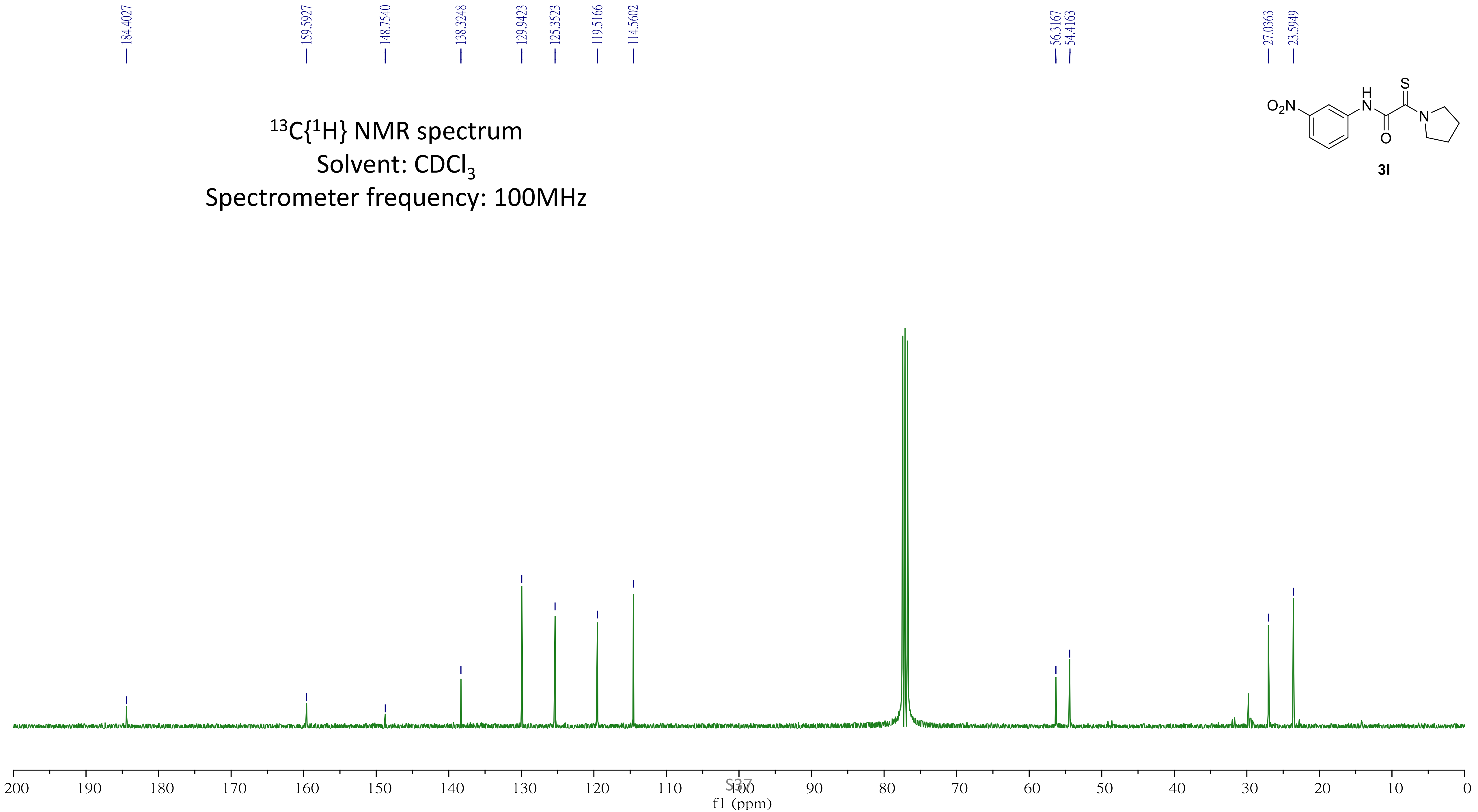
184.4027
159.5927
148.7540
138.3248
129.9423
125.3523
119.5166
114.5602
56.3167
54.4163
27.0363
23.5949
$^{13}C\{^1H\}$ NMR spectrum
Solvent: $CDCl_3$
Spectrometer frequency: 100MHz
O_2N
H
N
S
N
O
3l
200 190 180 170 160 150 140 130 120 110 100 90 80 70 60 50 40 30 20 10 0
f1 (ppm)

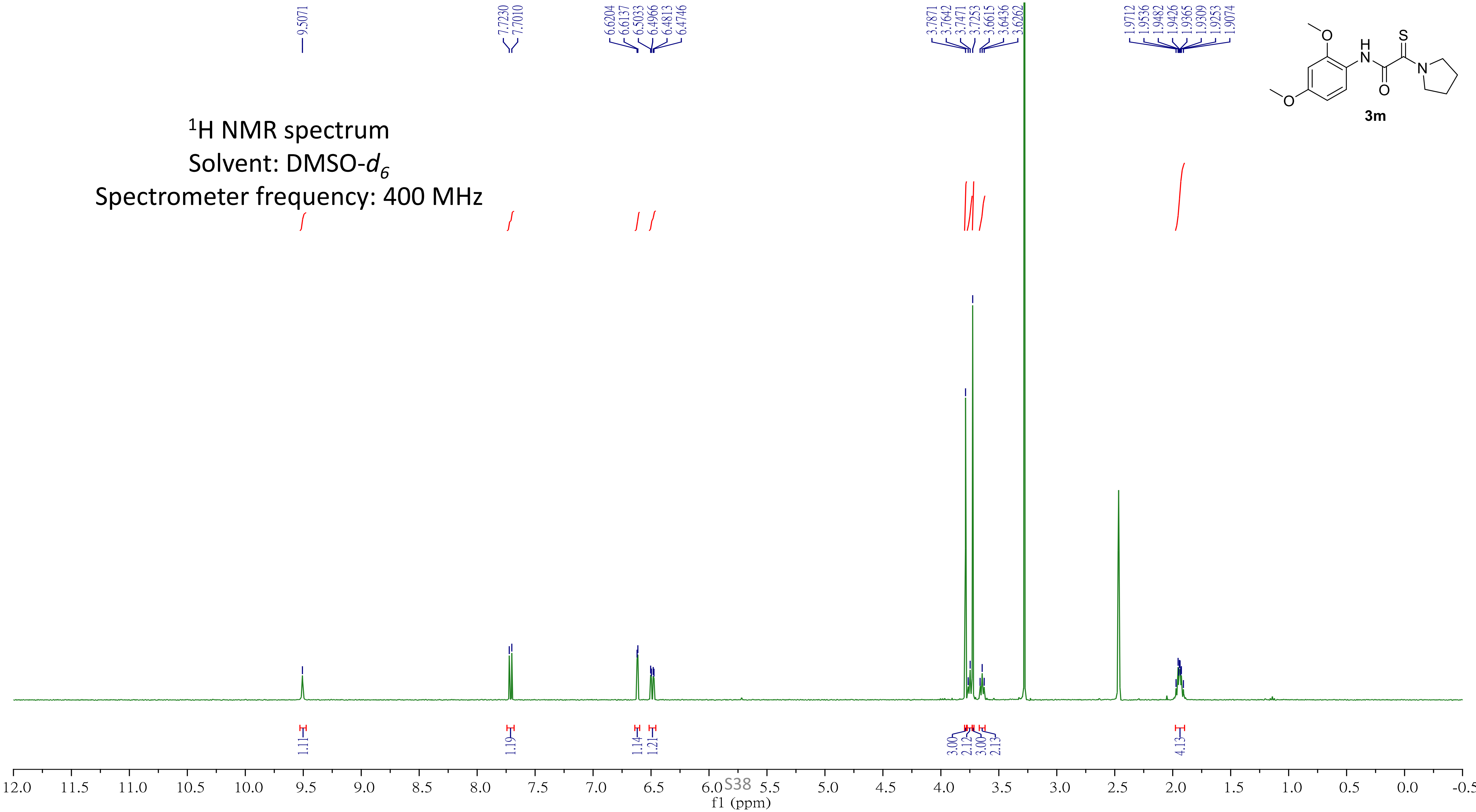

^{1}H NMR spectrum
Solvent: DMSO-d_6
Spectrometer frequency: 400 MHz
3m
9.5071
7.7230
7.7010
6.6204
6.6137
6.5033
6.4966
6.4813
6.4746
3.7871
3.7642
3.7471
3.7253
3.6615
3.6436
3.6262
1.9712
1.9536
1.9482
1.9426
1.9365
1.9309
1.9253
1.9074
1.11
1.19
1.14
1.21
3.00
2.12
3.00
2.13
4.13
f1 (ppm)

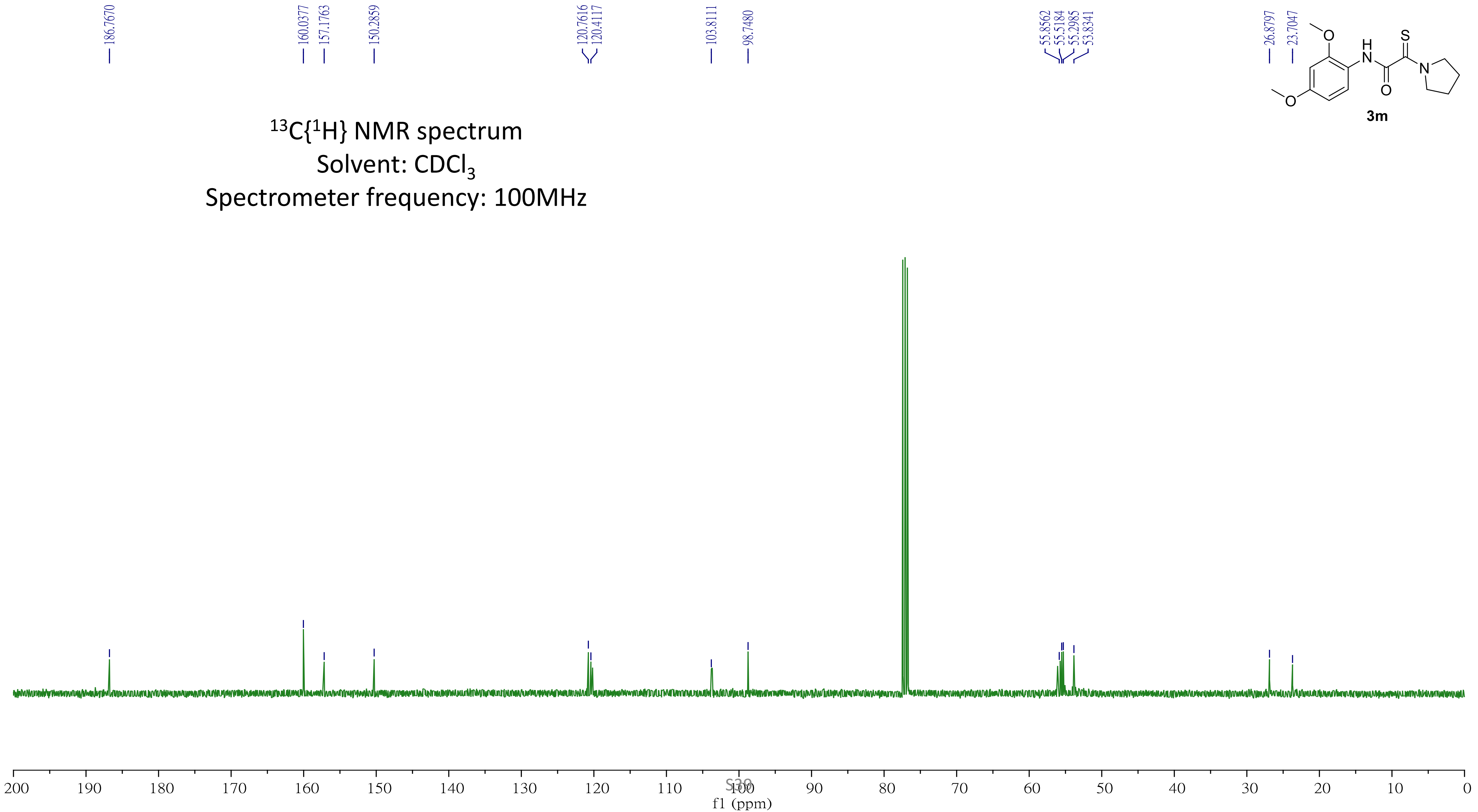

186.7670
160.0377
157.1763
150.2859
120.7616
120.4117
103.8111
98.7480
55.8562
55.5184
55.2985
53.8341
26.8797
23.7047
H
N
S
N
O
O
O
3m
13C{1H} NMR spectrum
Solvent: CDCl3
Spectrometer frequency: 100MHz
200 190 180 170 160 150 140 130 120 110 100 90 80 70 60 50 40 30 20 10 0
f1 (ppm)

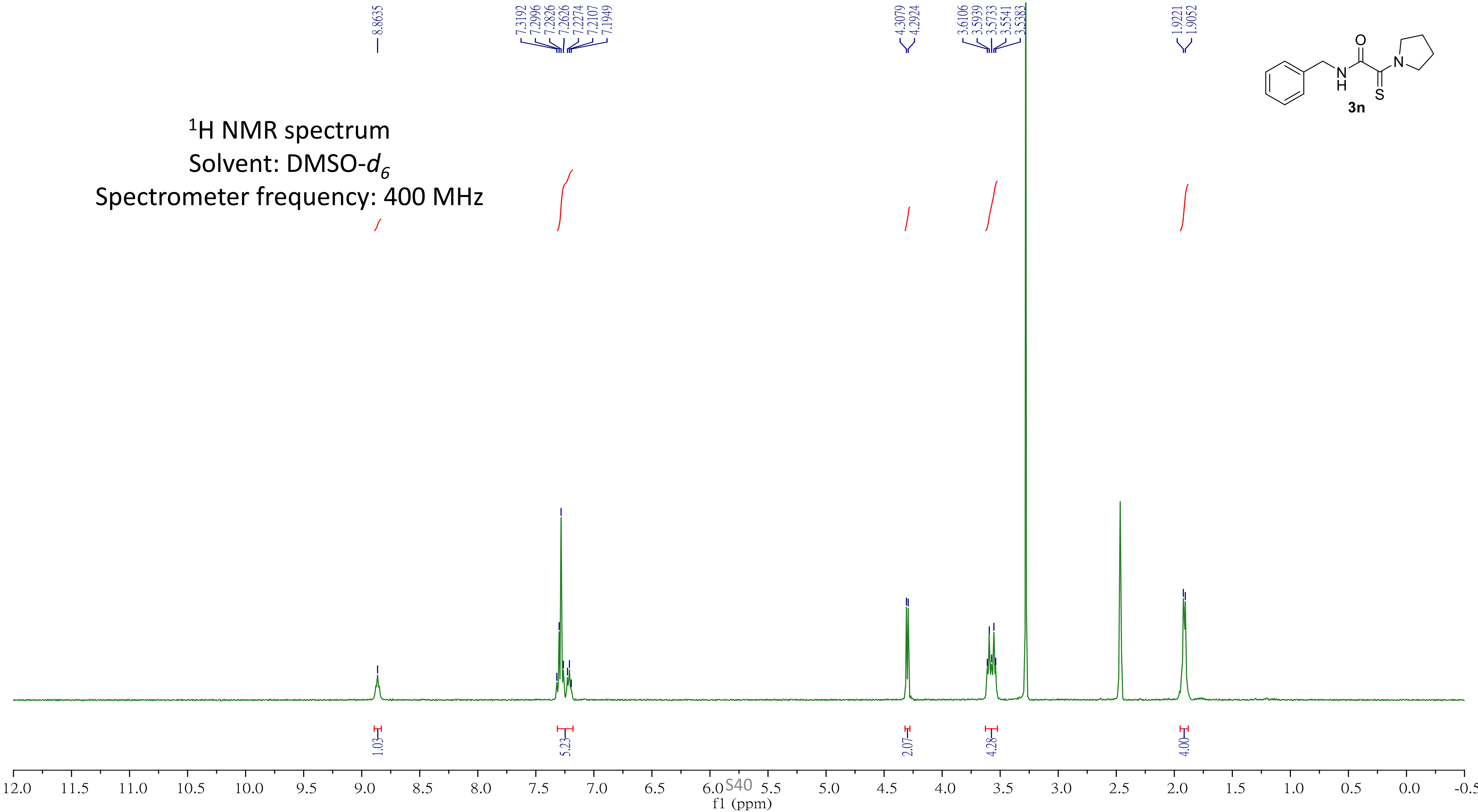

3n

^{1}H NMR spectrum
Solvent: DMSO-d_6
Spectrometer frequency: 400 MHz
8.8635
7.3192
7.2996
7.2826
7.2626
7.2274
7.2107
7.1949
4.3079
4.2924
3.6106
3.5939
3.5733
3.5541
3.5383
1.9221
1.9052
1.03
5.23
2.07
4.28
4.00
12.0
11.5
11.0
10.5
10.0
9.5
9.0
8.5
8.0
7.5
7.0
6.5
6.0
5.5
5.0
4.5
4.0
3.5
3.0
2.5
2.0
1.5
1.0
0.5
0.0
-0.5
f1 (ppm)

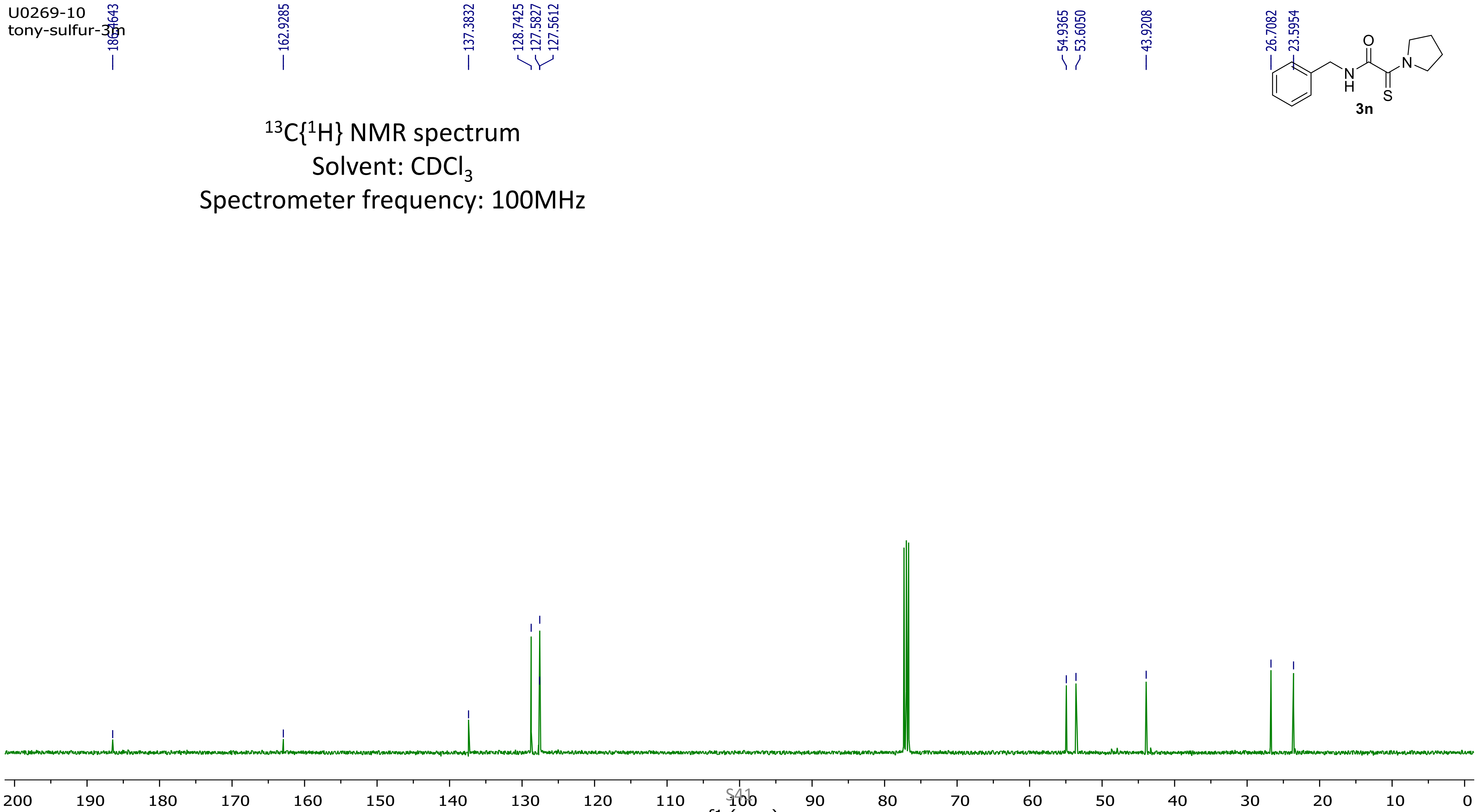
U0269-10
tony-sulfur-3m
162.9285
137.3832
128.7425
127.5827
127.5612
54.9365
53.6050
43.9208
26.7082
23.5954
O
N
H
N
S
3n
$^{13}C\{^{1}H\}$ NMR spectrum
Solvent: $CDCl_3$
Spectrometer frequency: 100MHz
200 190 180 170 160 150 140 130 120 110 100 90 80 70 60 50 40 30 20 10 0
f1 (ppm)

U0288-10
tony-sulfur-3n

^1H NMR spectrum
Solvent: $CDCl_3$
Spectrometer frequency: 400 MHz

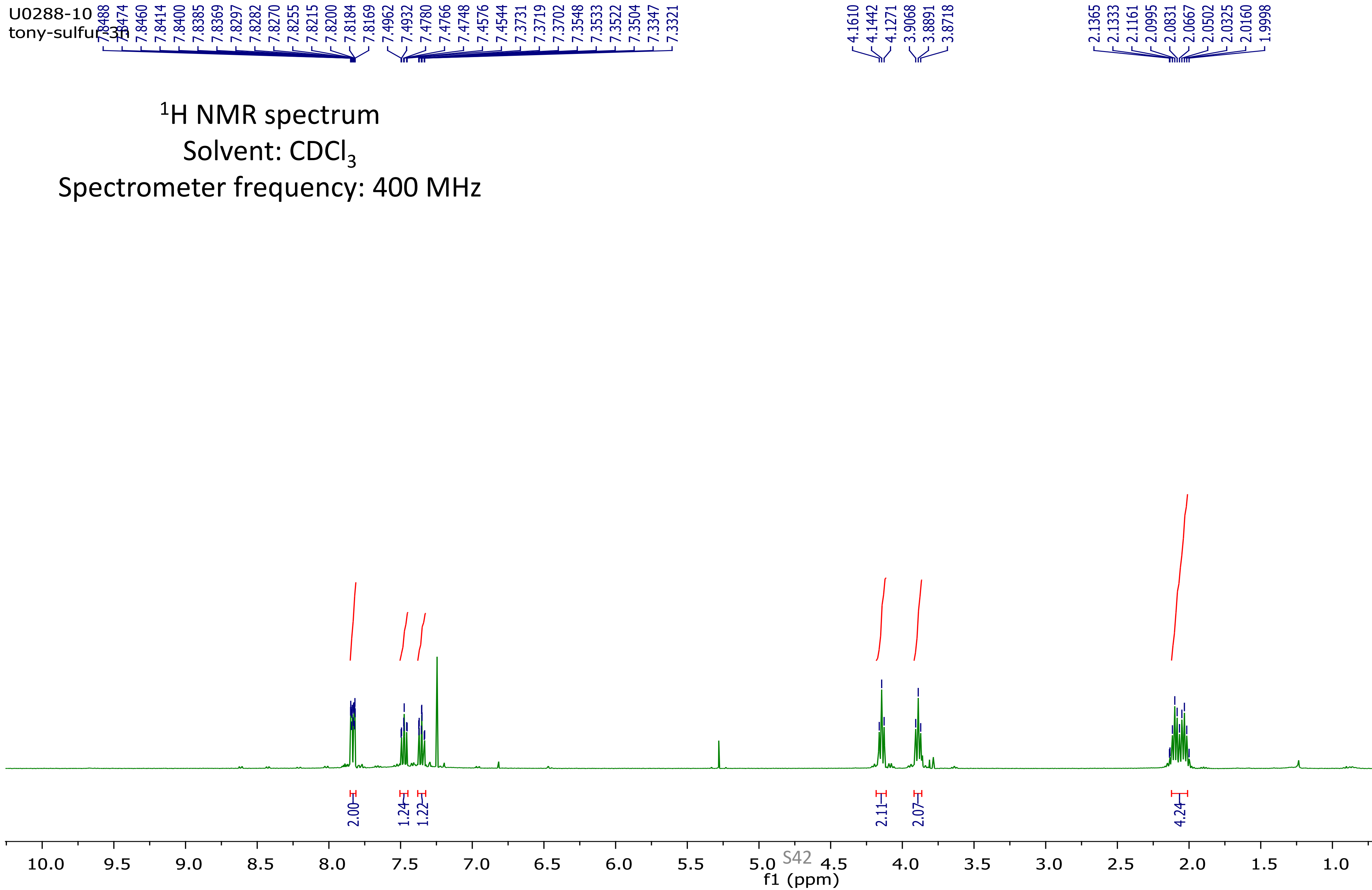

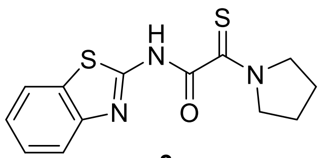

3o

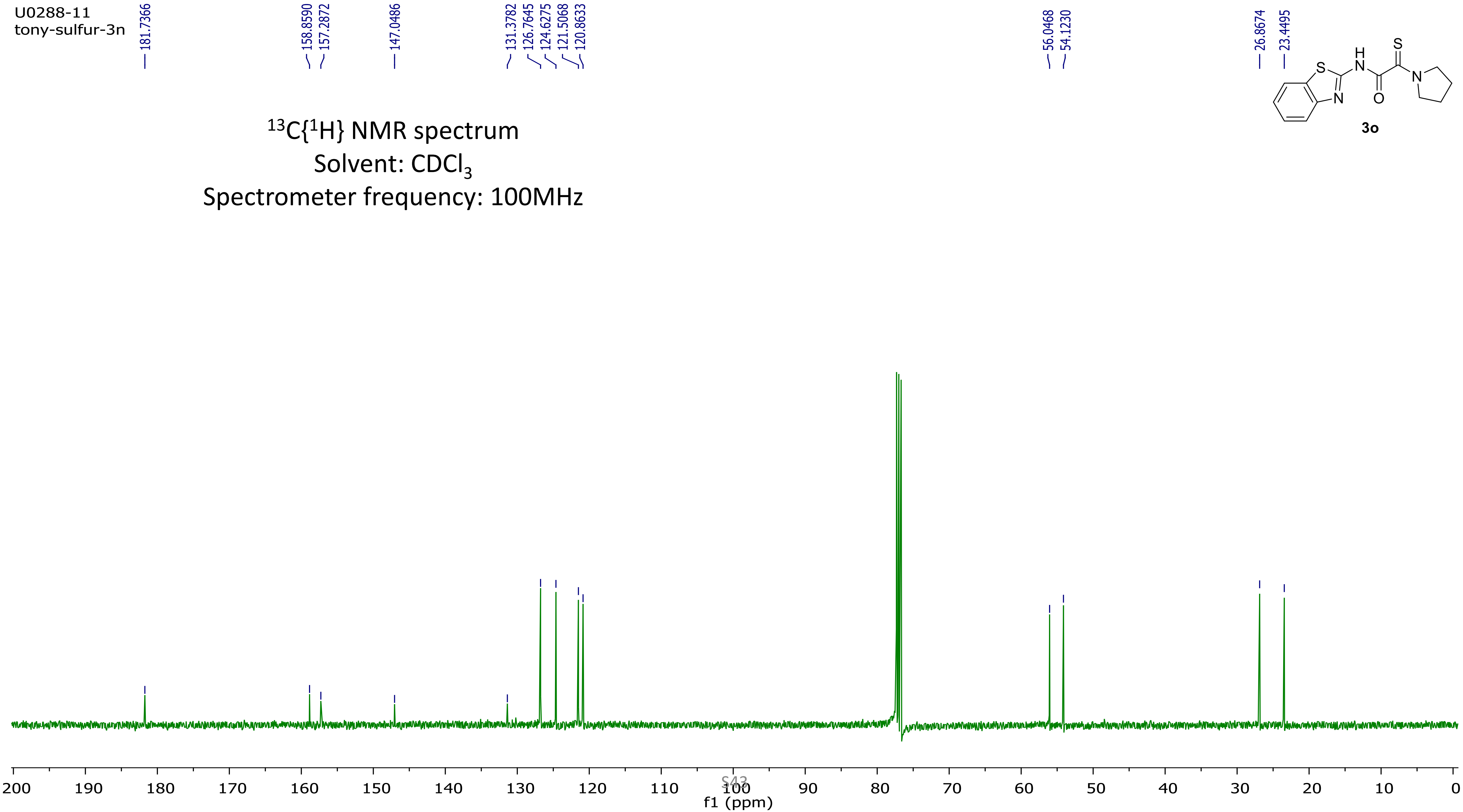
U0288-11
tony-sulfur-3n
181.7366
158.8590
157.2872
147.0486
131.3782
126.7645
124.6275
121.5068
120.8633
56.0468
54.1230
26.8674
23.4495
13C{1H} NMR spectrum
Solvent: CDCl3
Spectrometer frequency: 100MHz
200 190 180 170 160 150 140 130 120 110 100 90 80 70 60 50 40 30 20 10 0
f1 (ppm)
S
H
N
N
O
S
N

3o

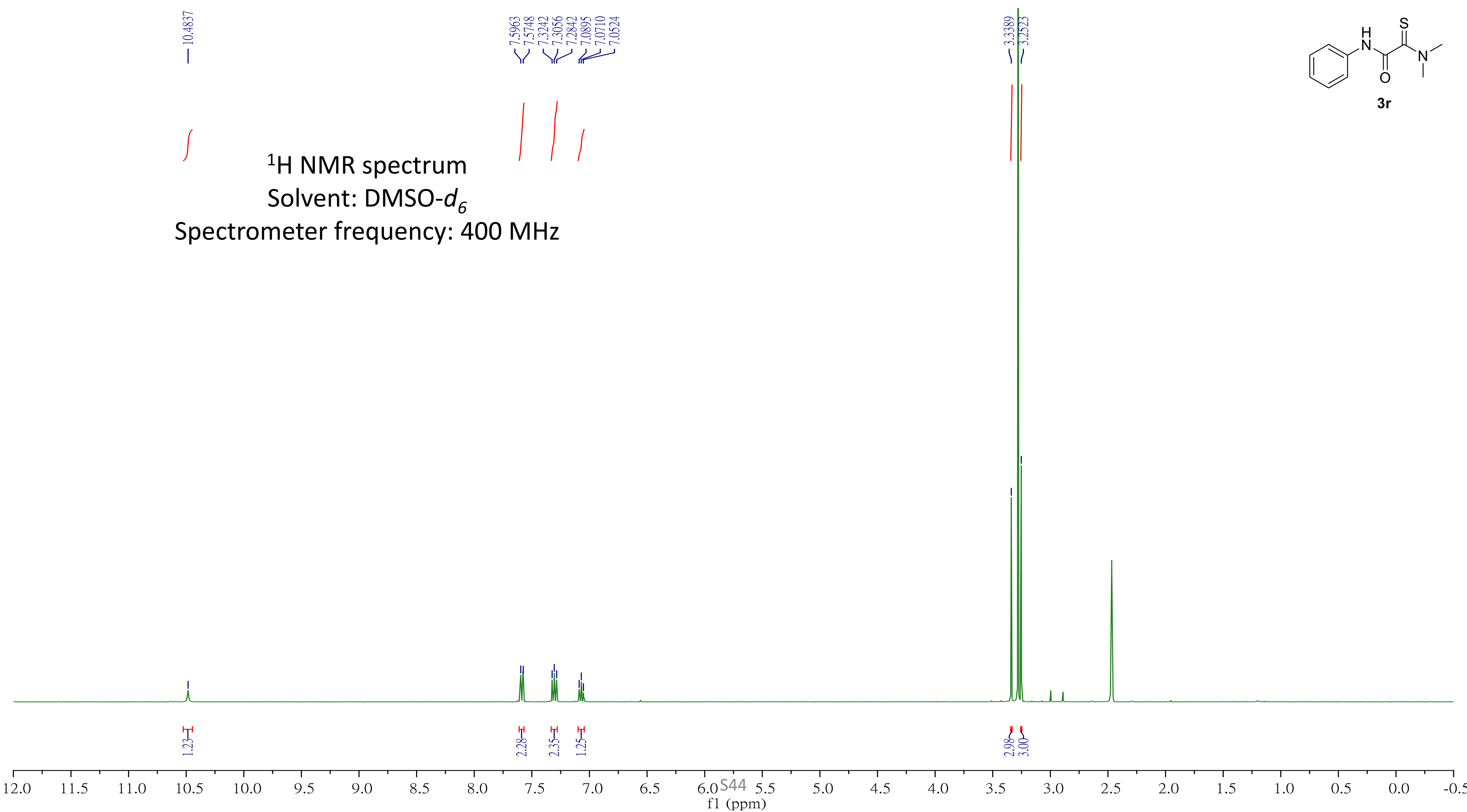

^{1}H NMR spectrum
Solvent: DMSO-d_6
Spectrometer frequency: 400 MHz
3r
10.4837
7.5963
7.5748
7.3242
7.3056
7.2842
7.0895
7.0710
7.0524
3.3389
3.2523
1.23
2.28
2.35
1.25
2.98
3.00
f1 (ppm)

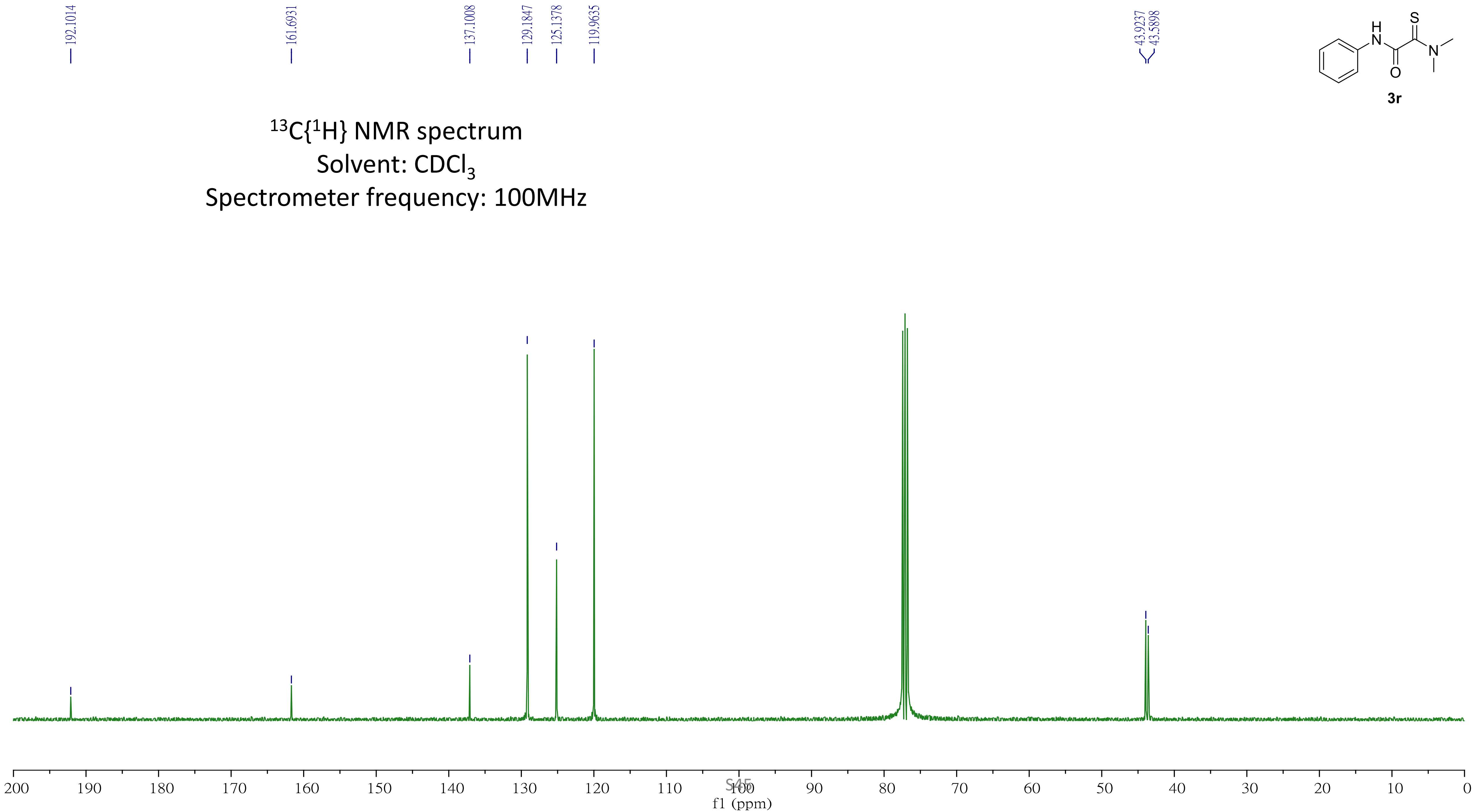

192.1014
161.6931
137.1008
129.1847
125.1378
119.9635
43.9237
43.5898
H
N
S
N
O
3r
$^{13}C\{^{1}H\}$ NMR spectrum
Solvent: $CDCl_3$
Spectrometer frequency: 100MHz
200 190 180 170 160 150 140 130 120 110 100 90 80 70 60 50 40 30 20 10 0
f1 (ppm)

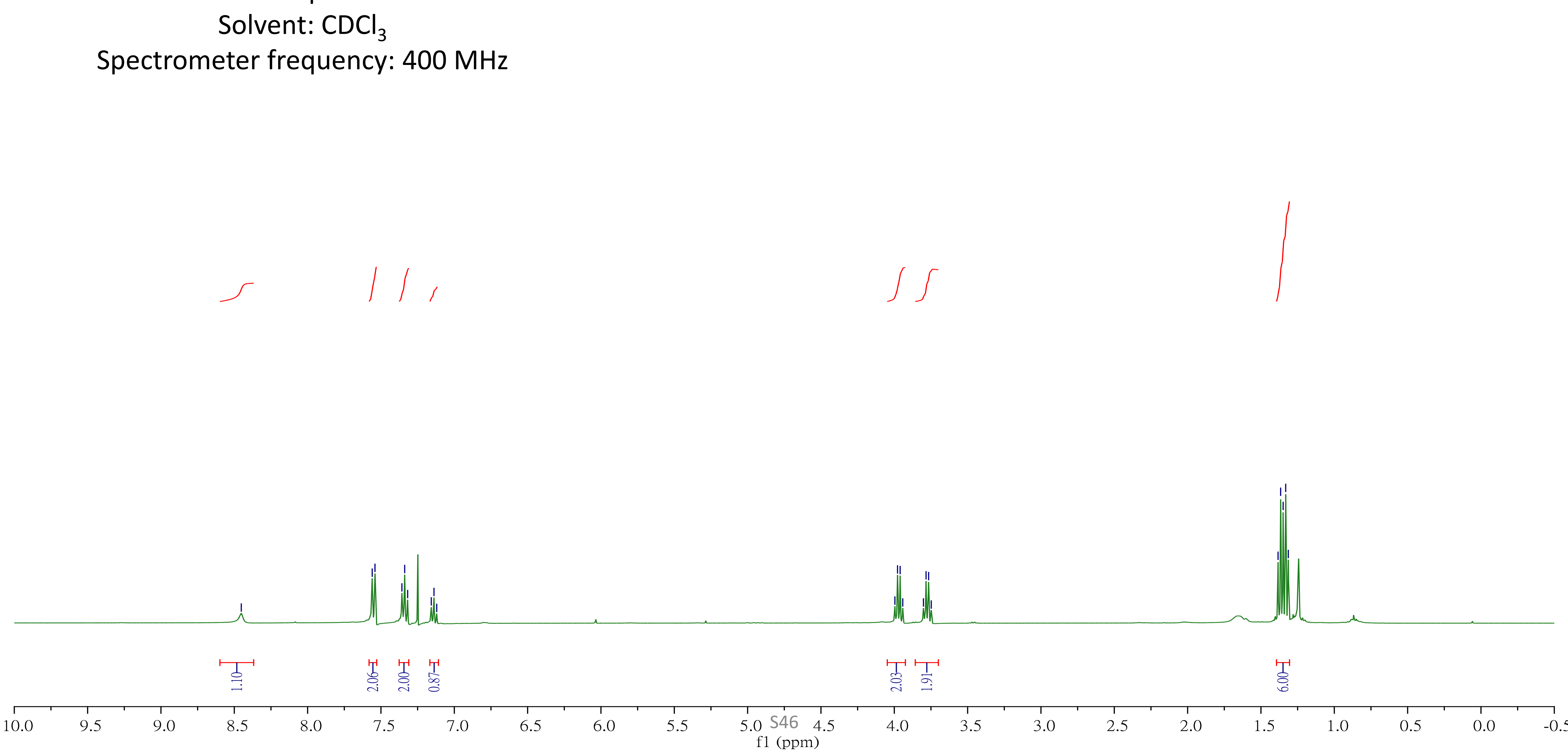
^{1}H NMR spectrum
Solvent: $CDCl_3$
Spectrometer frequency: 400 MHz
8.4524
7.5598
7.5407
7.3571
7.3381
7.3180
7.1570
7.1385
7.1198
3.9958
3.9779
3.9601
3.9423
3.8013
3.7835
3.7657
3.7480
1.3835
1.3658
1.3487
1.3313
1.3135
1.10
2.06
2.00
0.87
2.03
1.91
6.00
10.0 9.5 9.0 8.5 8.0 7.5 7.0 6.5 6.0 5.5 5.0 4.5 4.0 3.5 3.0 2.5 2.0 1.5 1.0 0.5 0.0 -0.5
f1 (ppm)

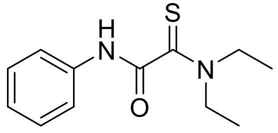
H
N
S
N
O
3s

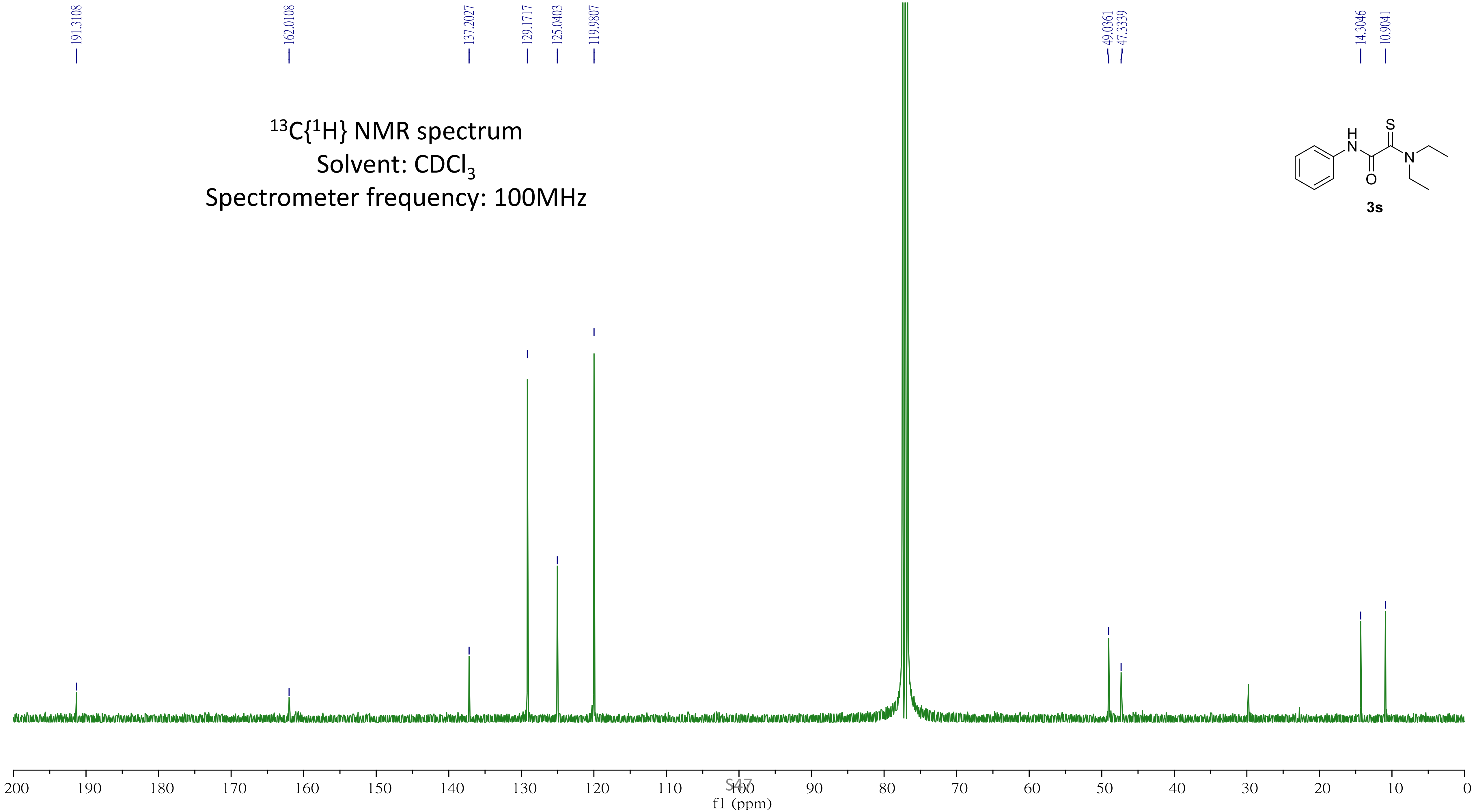

191.3108
162.0108
137.2027
129.1717
125.0403
119.9807
49.0361
47.3339
14.3046
10.9041
$^{13}C\{^1H\}$ NMR spectrum
Solvent: $CDCl_3$
Spectrometer frequency: 100MHz
H
N
S
N
O
3s
200 190 180 170 160 150 140 130 120 110 100 90 80 70 60 50 40 30 20 10 0
f1 (ppm)

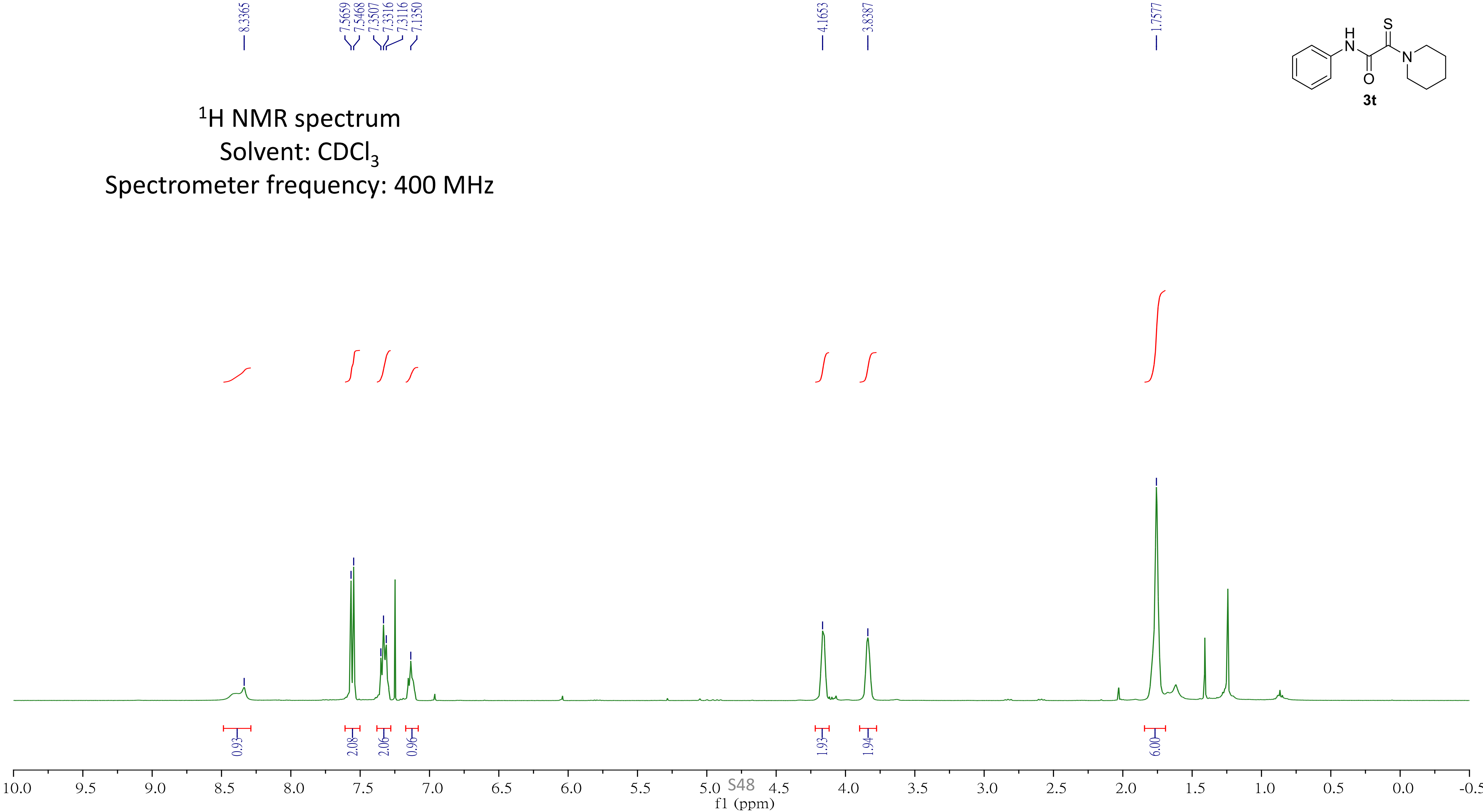

3t
^{1}H NMR spectrum
Solvent: CDCl3
Spectrometer frequency: 400 MHz
8.3365
7.5659
7.5468
7.3507
7.3316
7.3116
7.1350
4.1653
3.8387
1.7577
0.93
2.08
2.06
0.96
1.93
1.94
6.00
f1 (ppm)

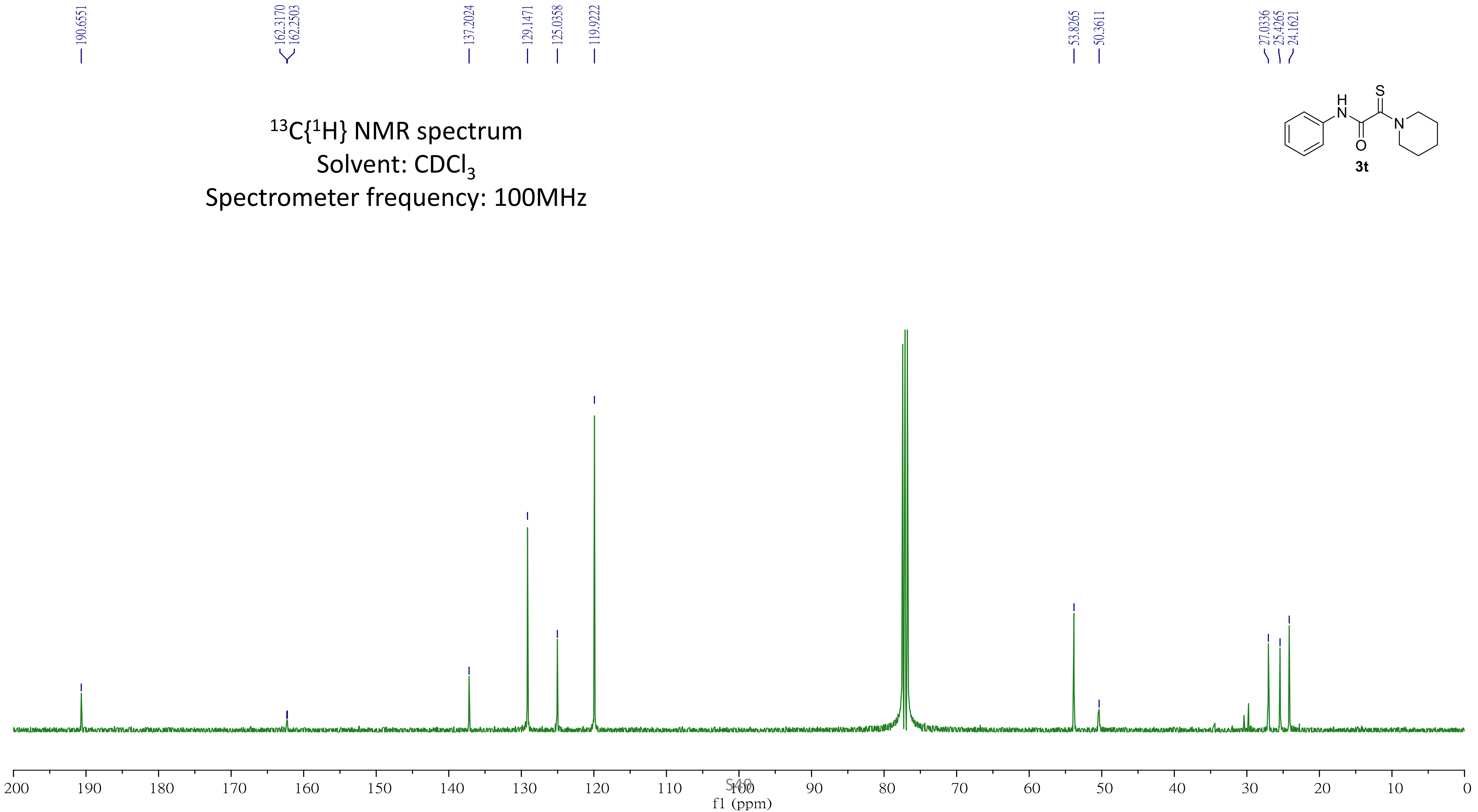
190.6551
162.3170
162.2503
137.2024
129.1471
125.0358
119.9222
53.8265
50.3611
27.0336
25.4265
24.1621
$^{13}C\{^1H\}$ NMR spectrum
Solvent: $CDCl_3$
Spectrometer frequency: 100MHz
H
N
S
N
O
3t
200 190 180 170 160 150 140 130 120 110 100 90 80 70 60 50 40 30 20 10 0
f1 (ppm)

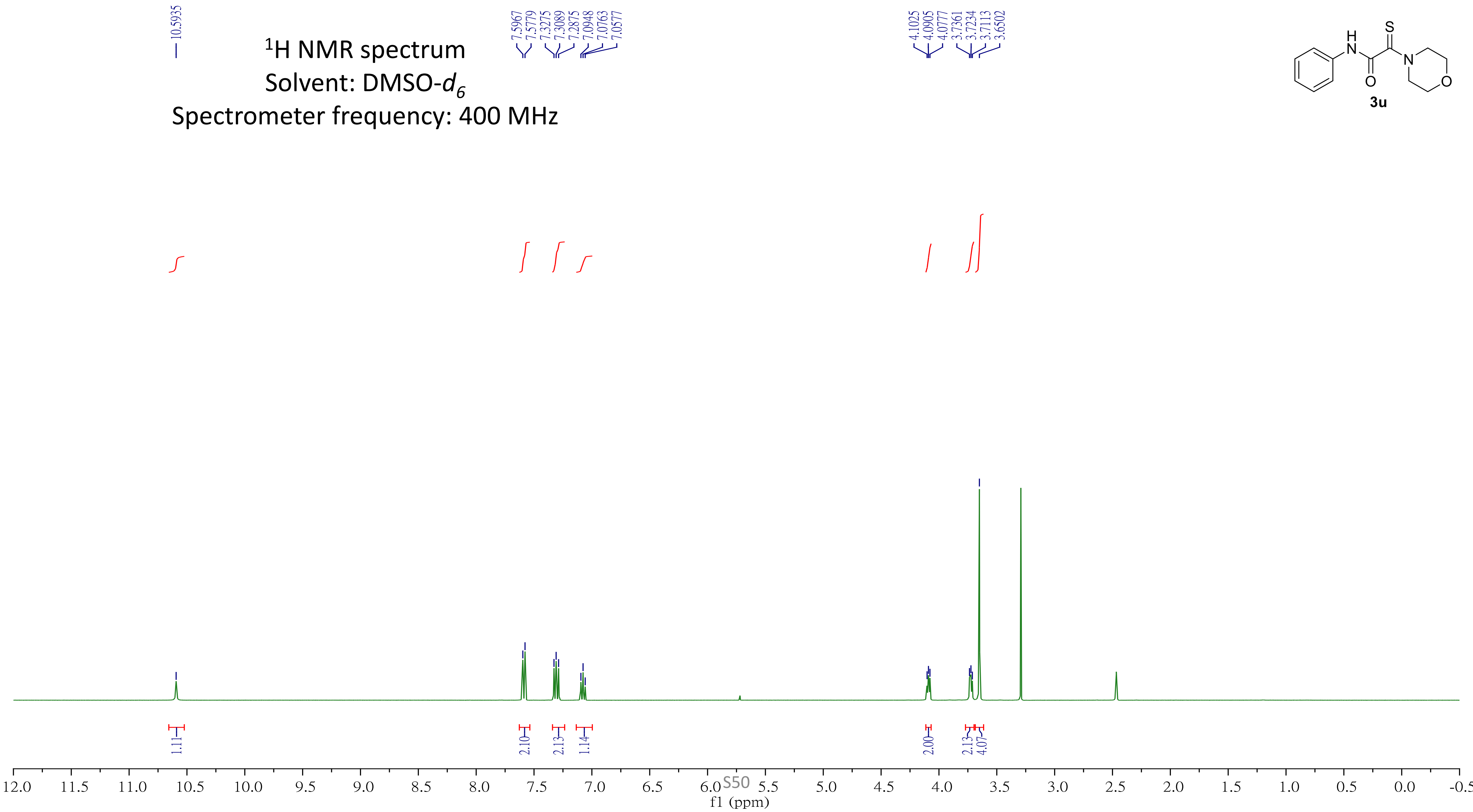

1H NMR spectrum
Solvent: DMSO-d6
Spectrometer frequency: 400 MHz
10.5935
7.5967
7.5779
7.3275
7.3089
7.2875
7.0948
7.0763
7.0577
4.1025
4.0905
4.0777
3.7361
3.7234
3.7113
3.6502
1.11
2.10
2.13
1.14
2.00
2.15
4.07
12.0 11.5 11.0 10.5 10.0 9.5 9.0 8.5 8.0 7.5 7.0 6.5 6.0 5.5 5.0 4.5 4.0 3.5 3.0 2.5 2.0 1.5 1.0 0.5 0.0 -0.5
f1 (ppm)
H
N
S
O
N
O
3u

AJ-V-91
single pulse decoupled gated NOE

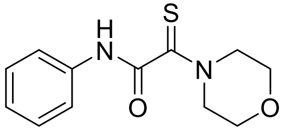

3u

$^{13}C\{^{1}H\}$ NMR spectrum
Solvent: DMSO-d_6
Spectrometer frequency: 100MHz

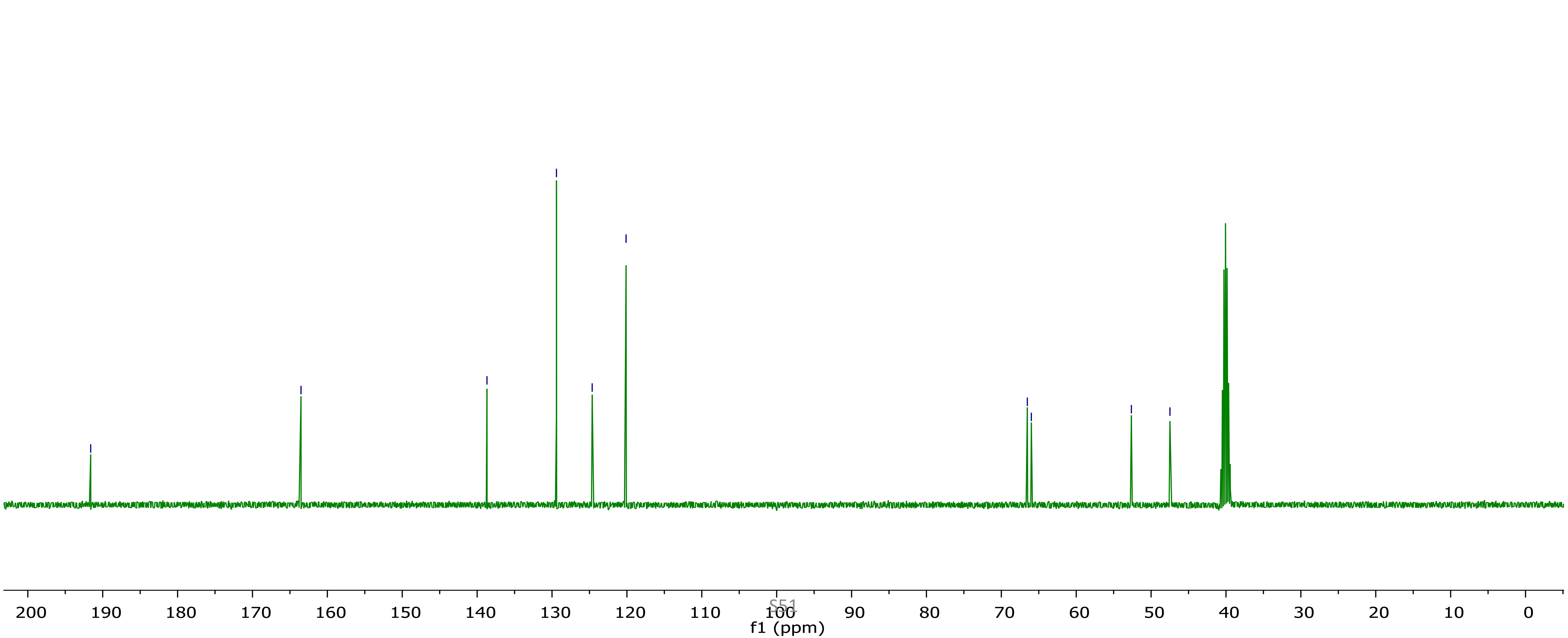

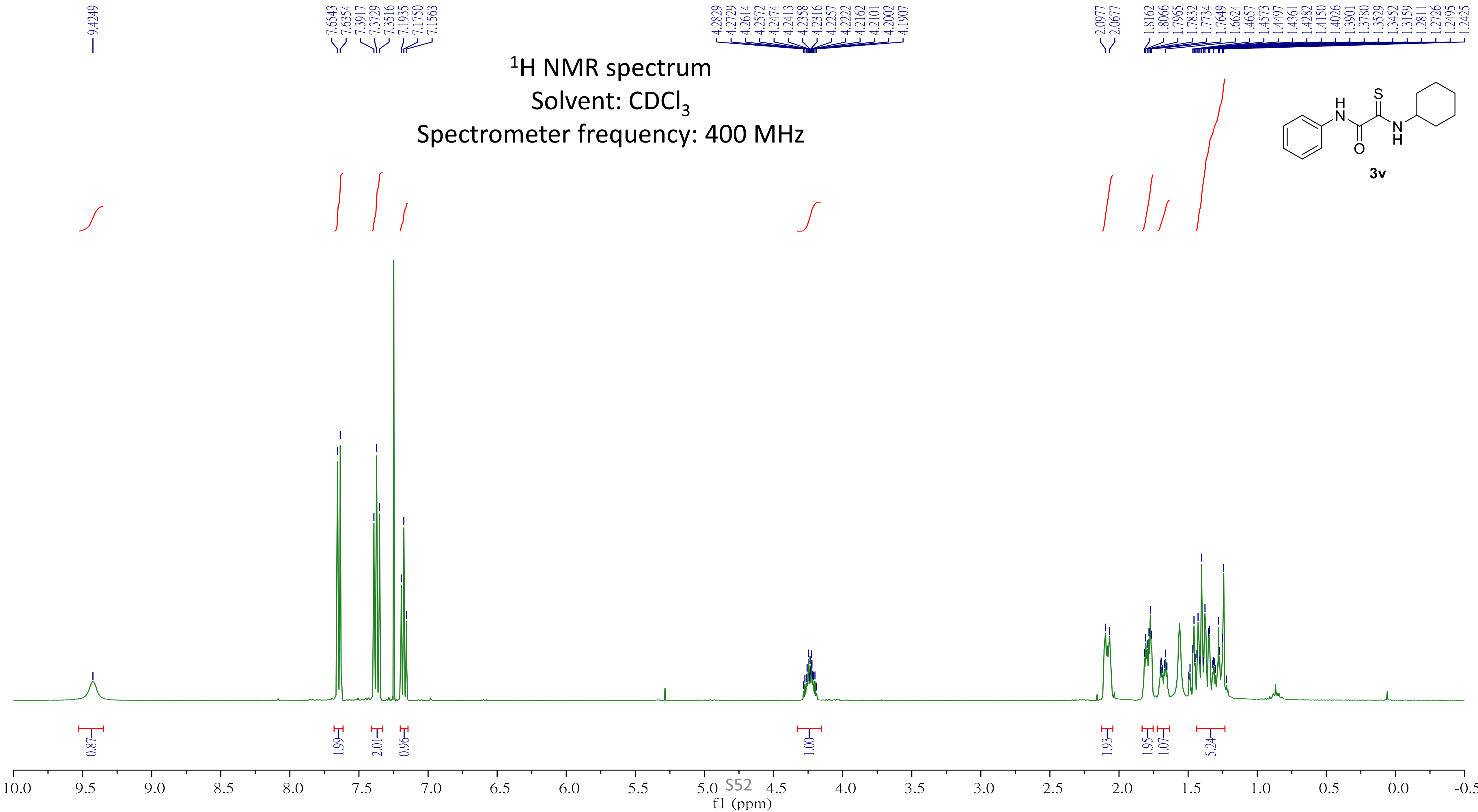

^{1}H NMR spectrum
Solvent: $CDCl_3$
Spectrometer frequency: 400 MHz
3v
f1 (ppm)

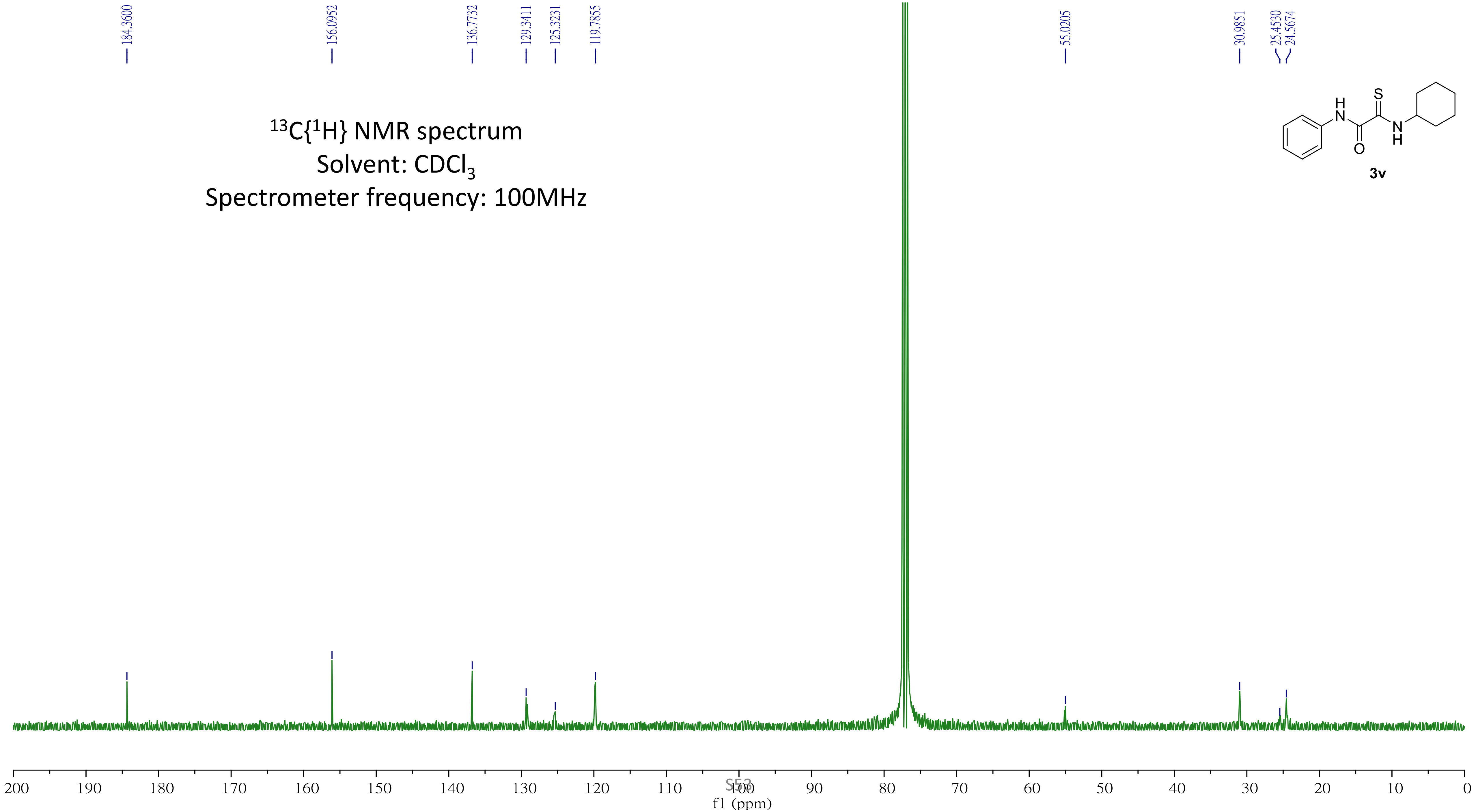

$^{13}C\{^{1}H\}$ NMR spectrum
Solvent: $CDCl_3$
Spectrometer frequency: 100MHz

3v

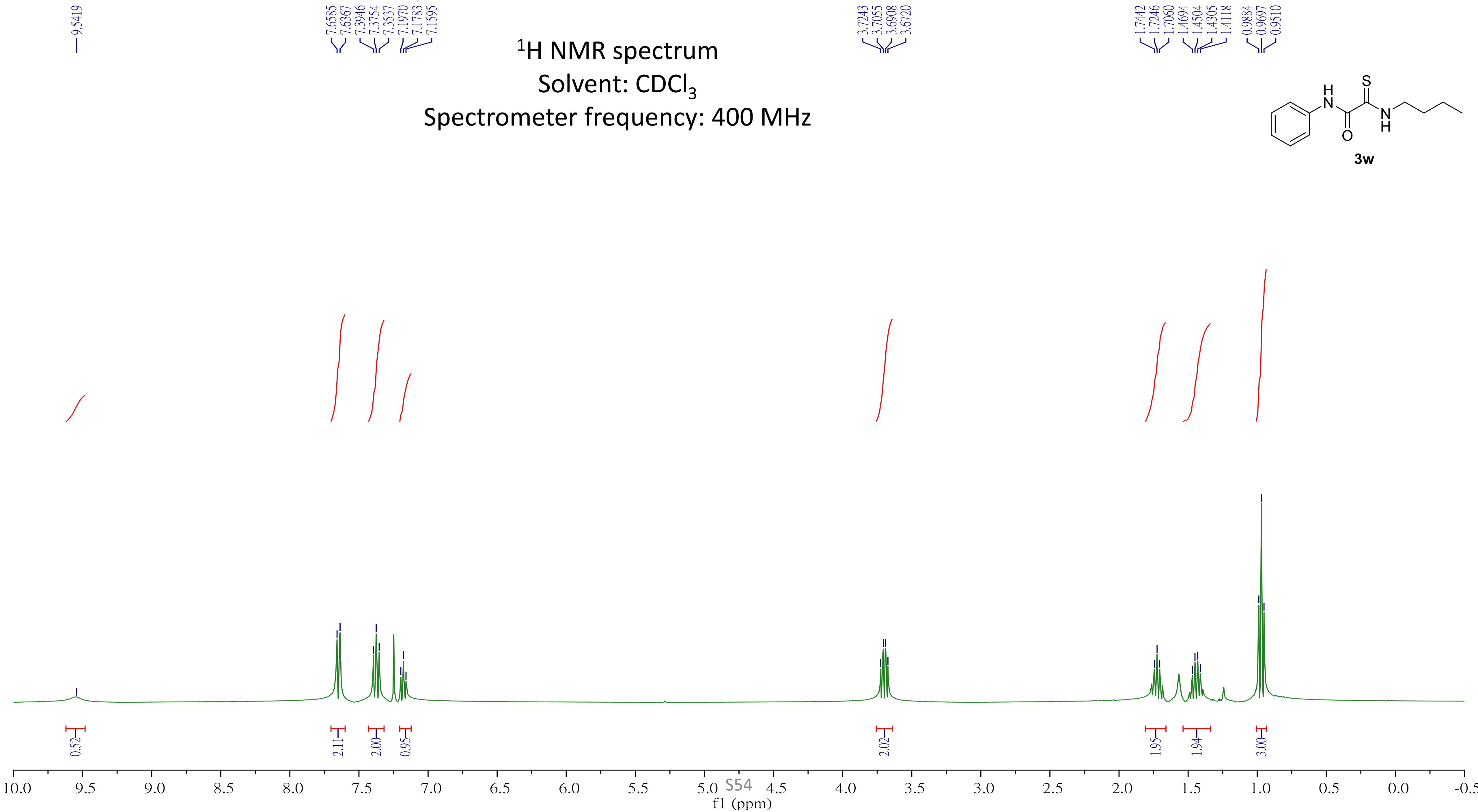

1H NMR spectrum
Solvent: CDCl3
Spectrometer frequency: 400 MHz
9.5419
7.6585 7.6367 7.3946 7.3754 7.3537 7.1970 7.1783 7.1595
3.7243 3.7055 3.6908 3.6720
1.7442 1.7246 1.7060 1.4694 1.4504 1.4305 1.4118 0.9884 0.9697 0.9510
S
H
N
N
H
O
3w
0.52
2.11
2.00
0.95
2.02
1.95
1.94
3.00
10.0 9.5 9.0 8.5 8.0 7.5 7.0 6.5 6.0 5.5 5.0 4.5 4.0 3.5 3.0 2.5 2.0 1.5 1.0 0.5 0.0 -0.5
f1 (ppm)

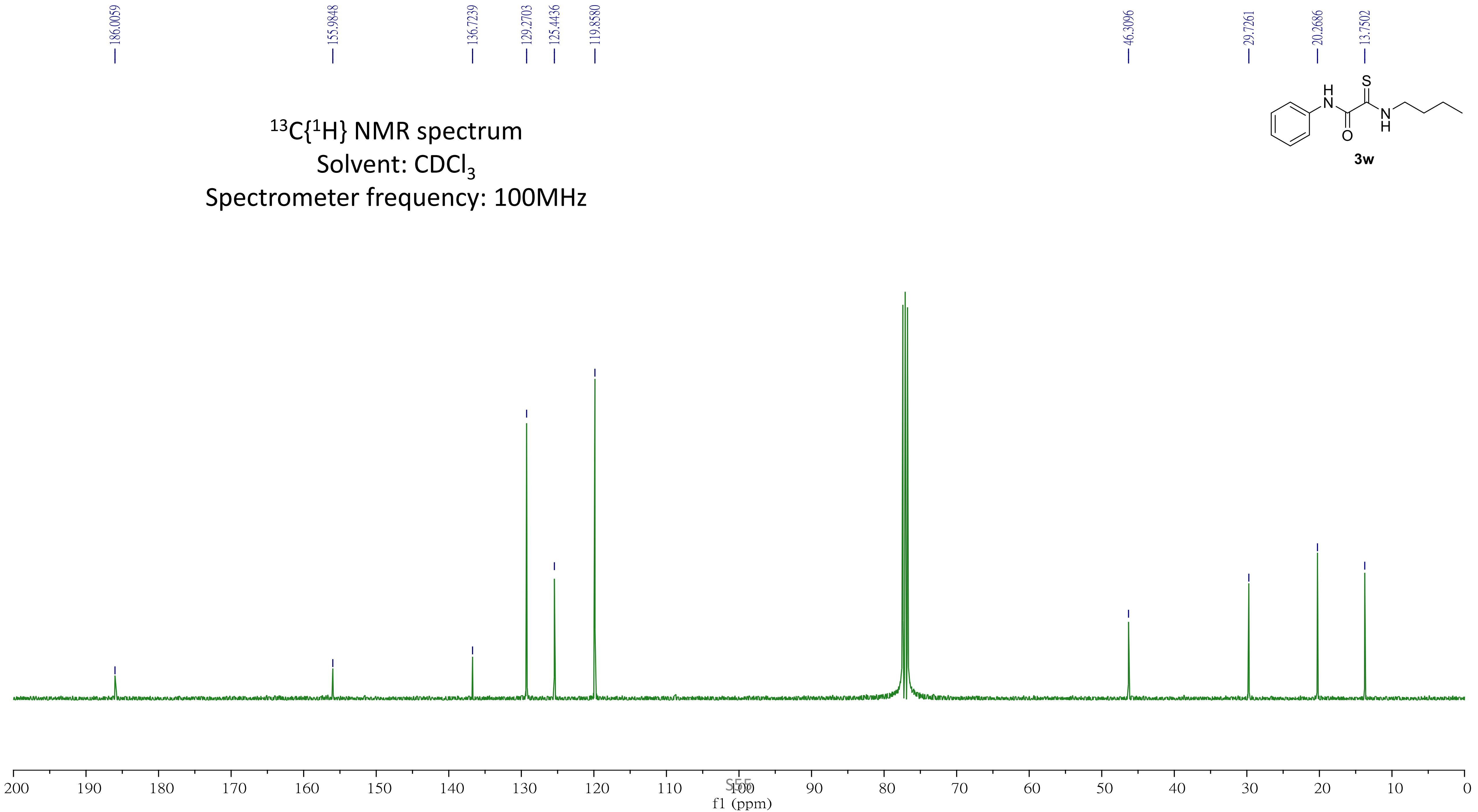
186.0059
155.9848
136.7239
129.2703
125.4436
119.8580
46.3096
29.7261
20.2686
13.7502
$^{13}C\{^1H\}$ NMR spectrum
Solvent: $CDCl_3$
Spectrometer frequency: 100MHz
3w
200 190 180 170 160 150 140 130 120 110 100 90 80 70 60 50 40 30 20 10 0
f1 (ppm)

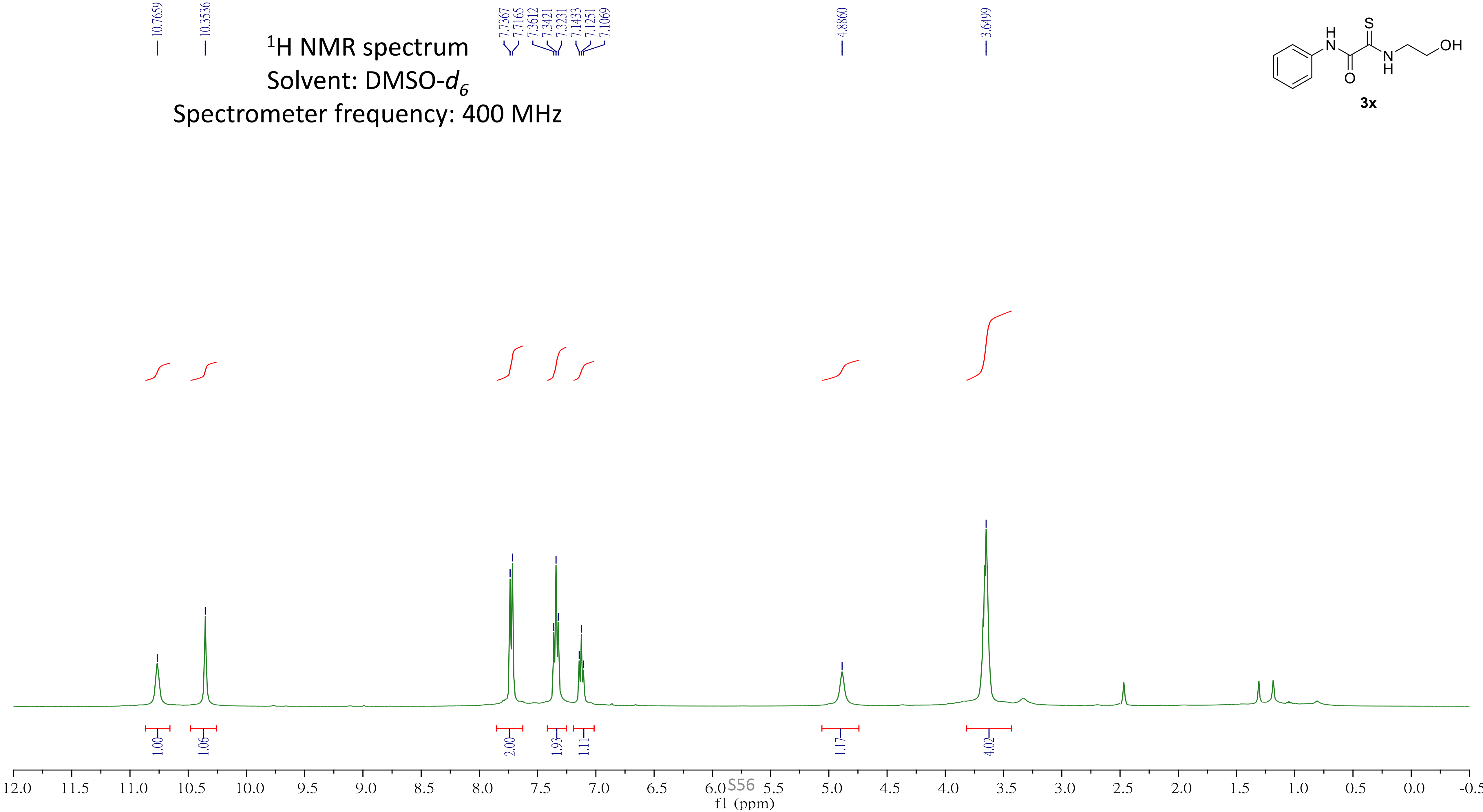

^{1}H NMR spectrum
Solvent: DMSO-d_6
Spectrometer frequency: 400 MHz
10.7659
10.3536
7.7367
7.7165
7.3612
7.3421
7.3231
7.1433
7.1251
7.1069
4.8860
3.6499
1.00
1.06
2.00
1.93
1.11
1.17
4.02
f1 (ppm)
3x

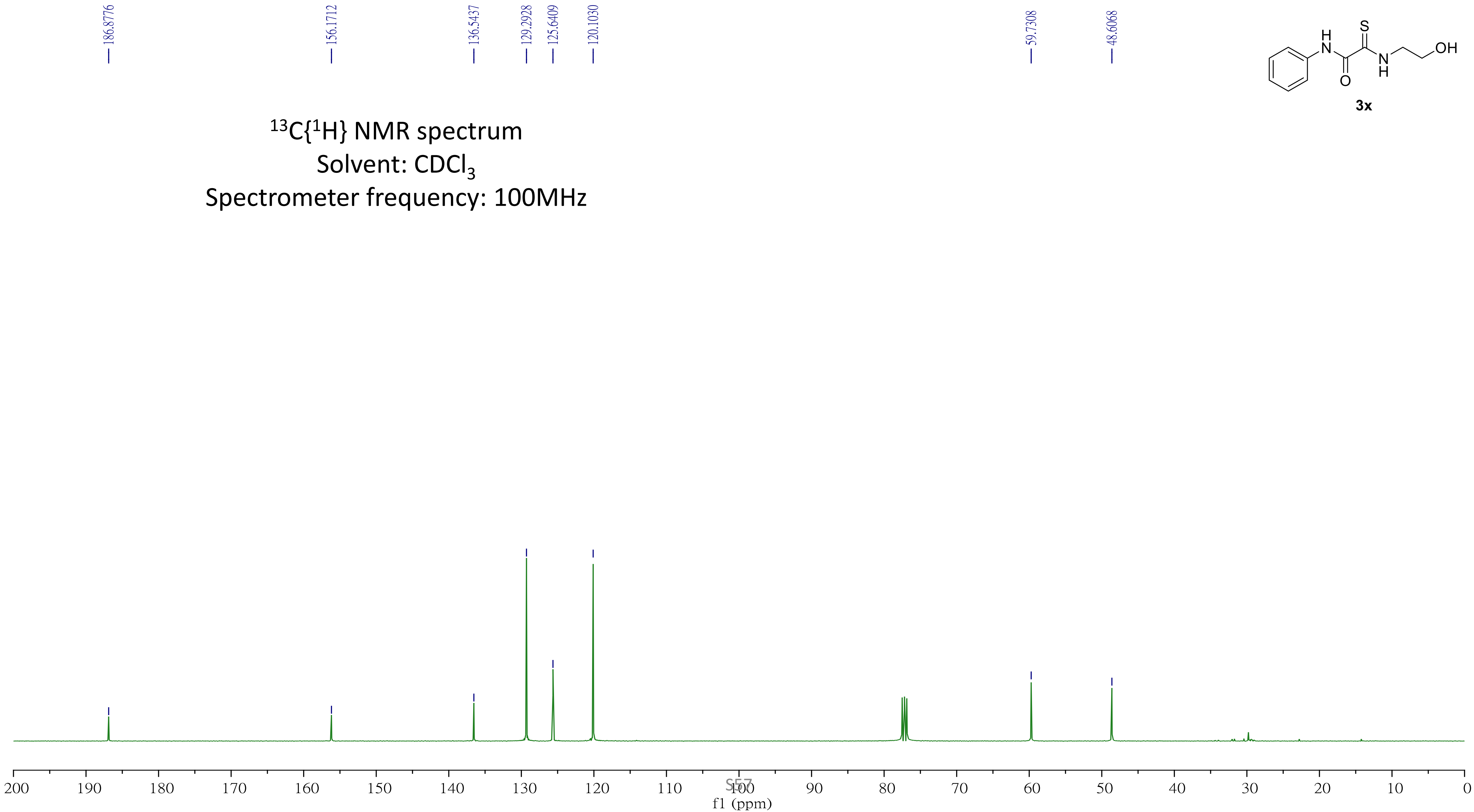
186.8776
156.1712
136.5437
129.2928
125.6409
120.1030
59.7308
48.6068
3x
$^{13}C\{^{1}H\}$ NMR spectrum
Solvent: $CDCl_3$
Spectrometer frequency: 100MHz
200 190 180 170 160 150 140 130 120 110 100 90 80 70 60 50 40 30 20 10 0
f1 (ppm)

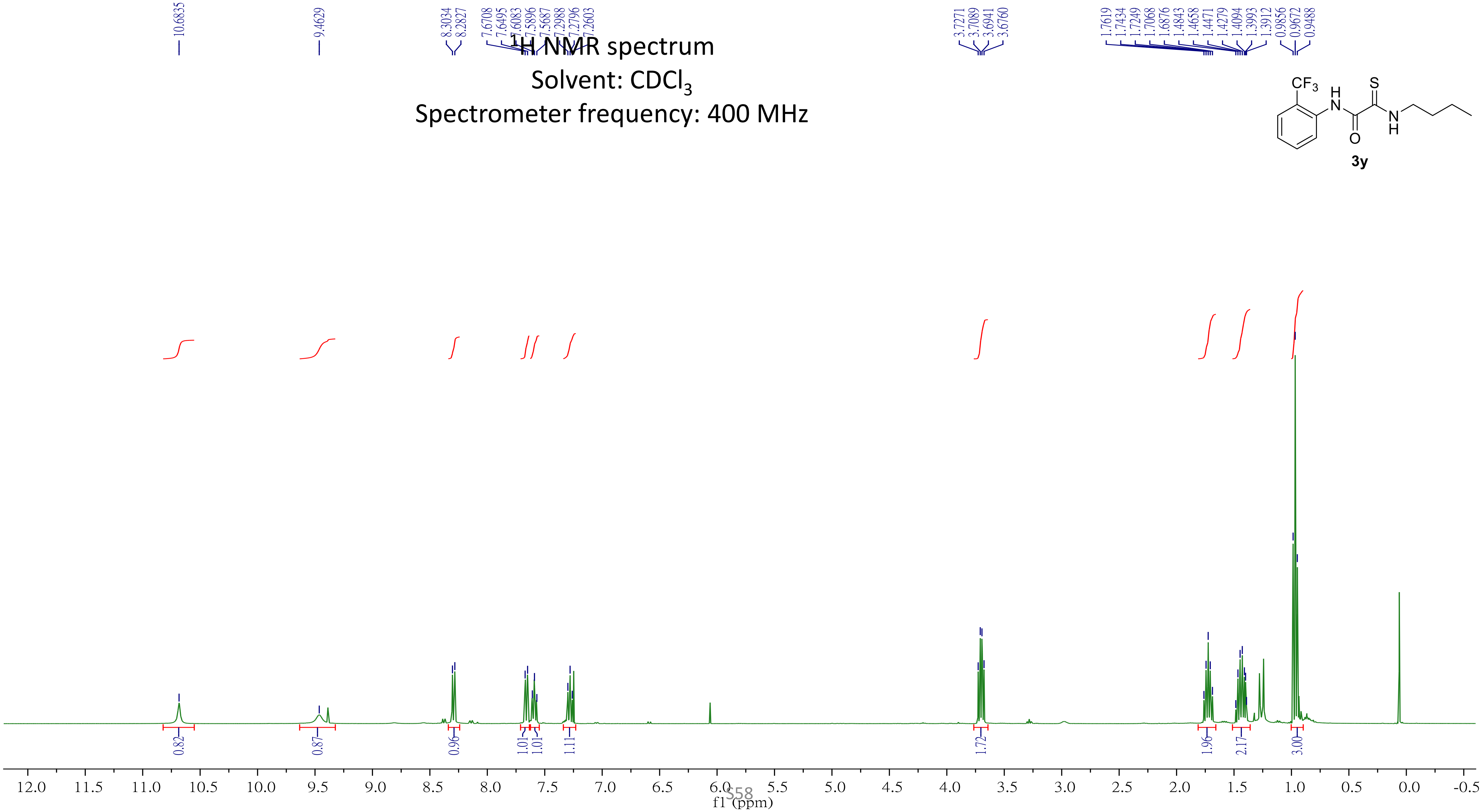

10.6835
9.4629
8.3034
8.2827
7.6708
7.6495
7.6083
7.5896
7.5687
7.2988
7.2796
7.2603
3.7271
3.7089
3.6941
3.6760
1.7619
1.7434
1.7249
1.7068
1.6876
1.4843
1.4658
1.4471
1.4279
1.4094
1.3993
1.3912
0.9856
0.9672
0.9488
1H NMR spectrum
Solvent: CDCl3
Spectrometer frequency: 400 MHz
0.82
0.87
0.96
1.01
1.01
1.11
1.72
1.96
2.17
3.00
12.0 11.5 11.0 10.5 10.0 9.5 9.0 8.5 8.0 7.5 7.0 6.5 6.0 5.5 5.0 4.5 4.0 3.5 3.0 2.5 2.0 1.5 1.0 0.5 0.0 -0.5
f1 (ppm)
CF3
H
N
S
N
H
O

3y

$^{13}C\{^{1}H\}$ NMR spectrum
Solvent: $CDCl_3$
Spectrometer frequency: 100MHz

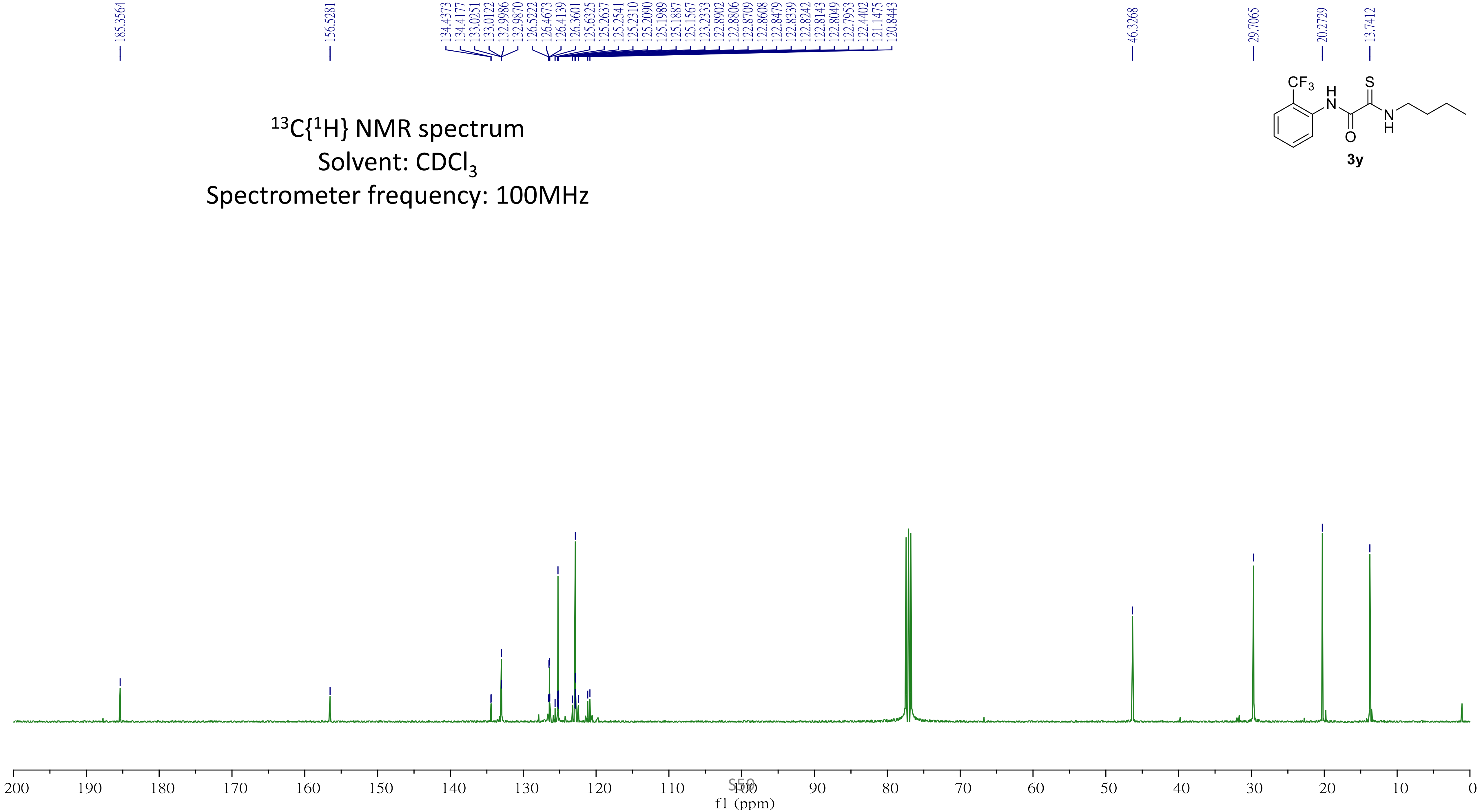

3y

$^{13}C\{^{1}H\}$ NMR spectrum
Solvent: $CDCl_3$
Spectrometer frequency: 100MHz

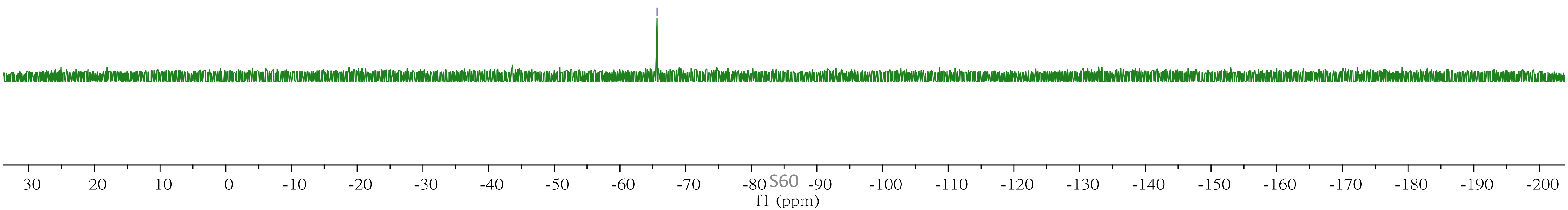

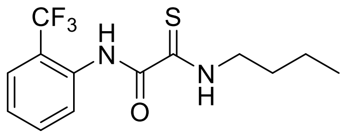

3y

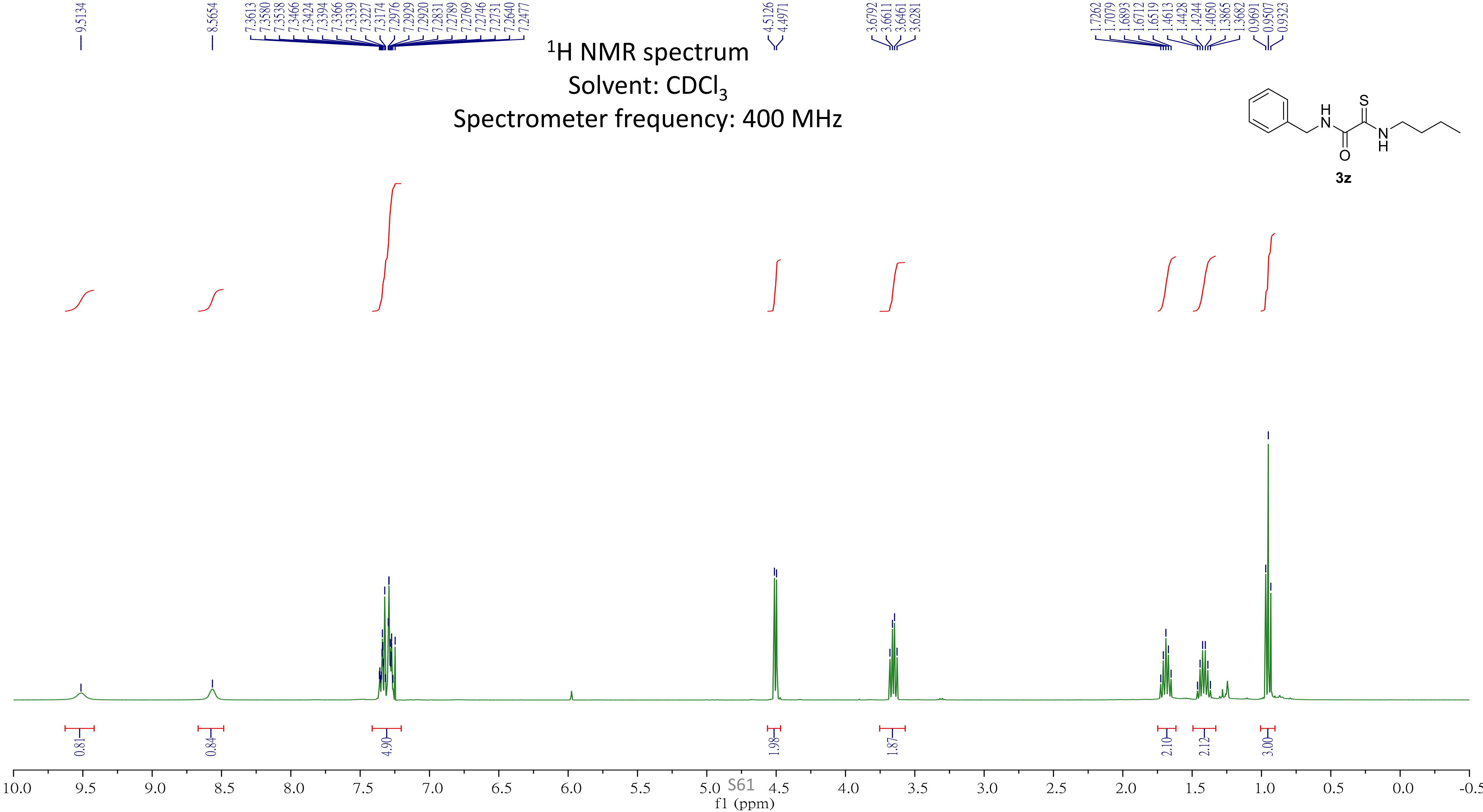

9.5134
8.5654
7.3613 7.3580 7.3538 7.3466 7.3424 7.3394 7.3366 7.3339 7.3227 7.3174 7.2976 7.2929 7.2920 7.2831 7.2789 7.2769 7.2746 7.2731 7.2640 7.2477
4.5126 4.4971
3.6792 3.6611 3.6461 3.6281
1.7262 1.7079 1.6893 1.6712 1.6519 1.4613 1.4428 1.4244 1.4050 1.3865 1.3682 0.9691 0.9507 0.9323
^{1}H NMR spectrum
Solvent: $CDCl_3$
Spectrometer frequency: 400 MHz
0.81
0.84
4.90
1.98
1.87
2.10
2.12
3.00
10.0 9.5 9.0 8.5 8.0 7.5 7.0 6.5 6.0 5.5 5.0 4.5 4.0 3.5 3.0 2.5 2.0 1.5 1.0 0.5 0.0 -0.5
f1 (ppm)
H
N
S
O
N
H

3z

$^{13}C\{^1H\}$ NMR spectrum
Solvent: $CDCl_3$
Spectrometer frequency: 100MHz

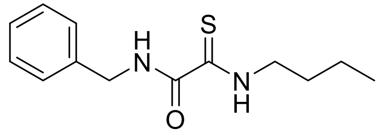

3z

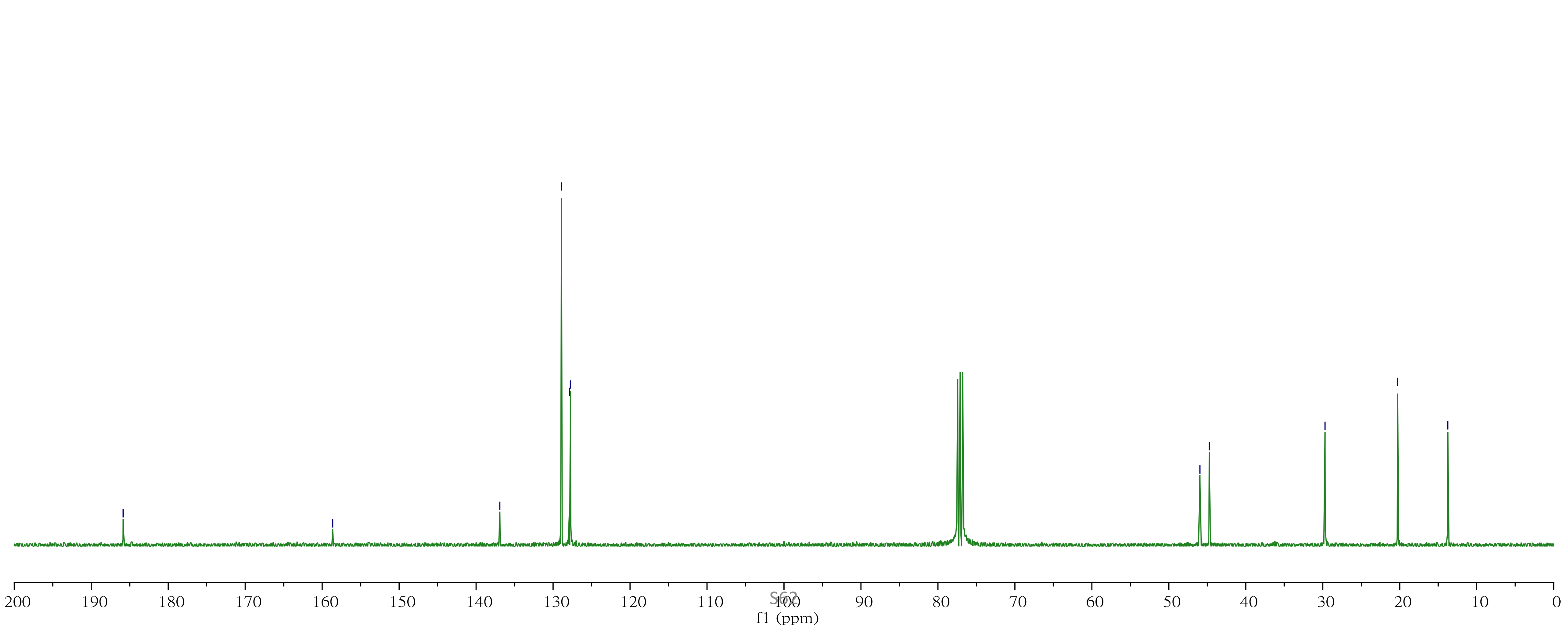

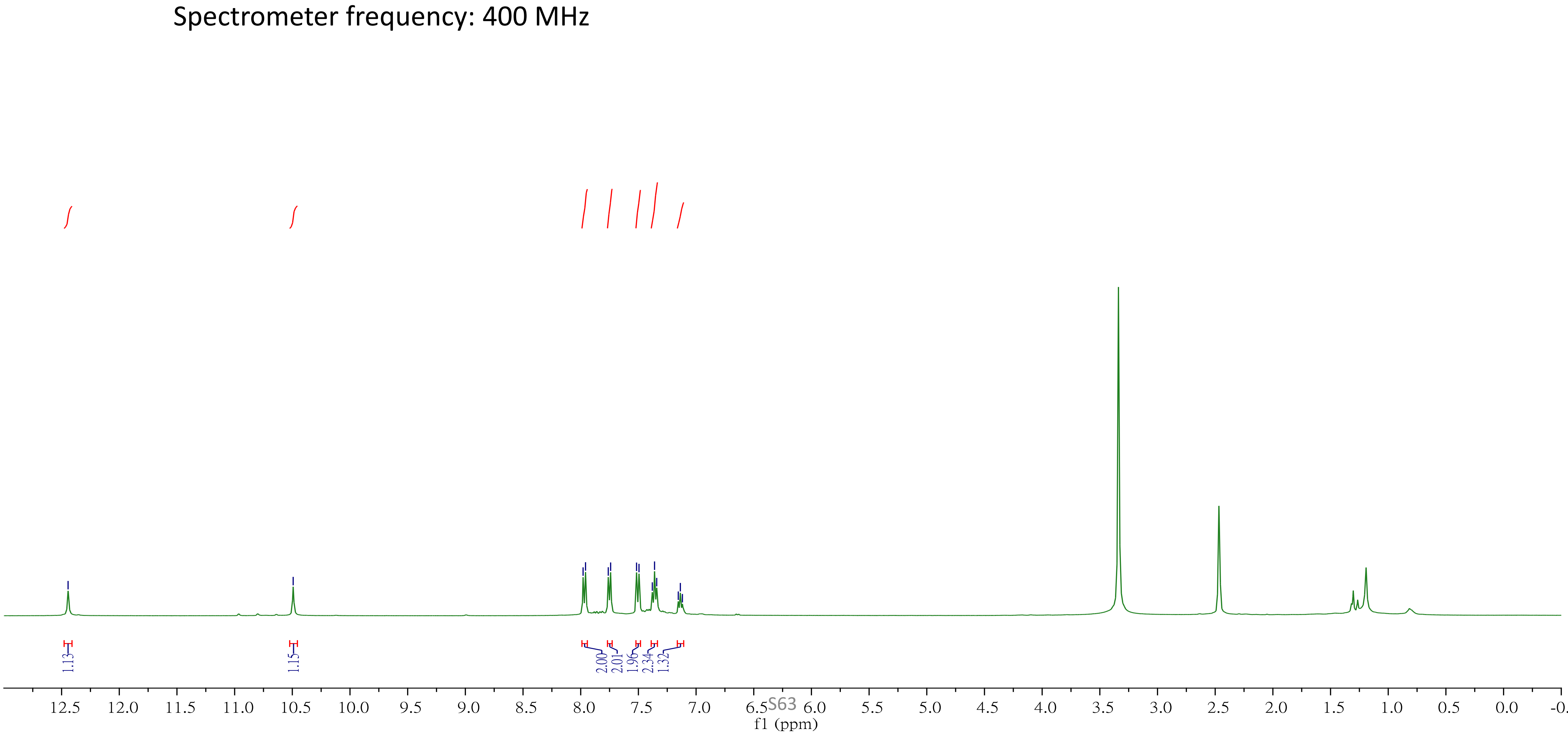

^{1}H NMR spectrum
Solvent: DMSO-d_6
Spectrometer frequency: 400 MHz
12.4438
10.4925
7.9794
7.9582
7.7611
7.7406
7.5159
7.4948
7.3793
7.3601
7.3412
7.1542
7.1363
7.1184
1.13
1.15
2.00
2.01
1.96
2.34
1.32
12.5 12.0 11.5 11.0 10.5 10.0 9.5 9.0 8.5 8.0 7.5 7.0 6.5 6.0 5.5 5.0 4.5 4.0 3.5 3.0 2.5 2.0 1.5 1.0 0.5 0.0 -0.5
f1 (ppm)

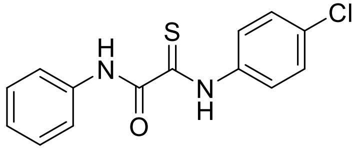

H
N
S
N
H
O
Cl

3aa

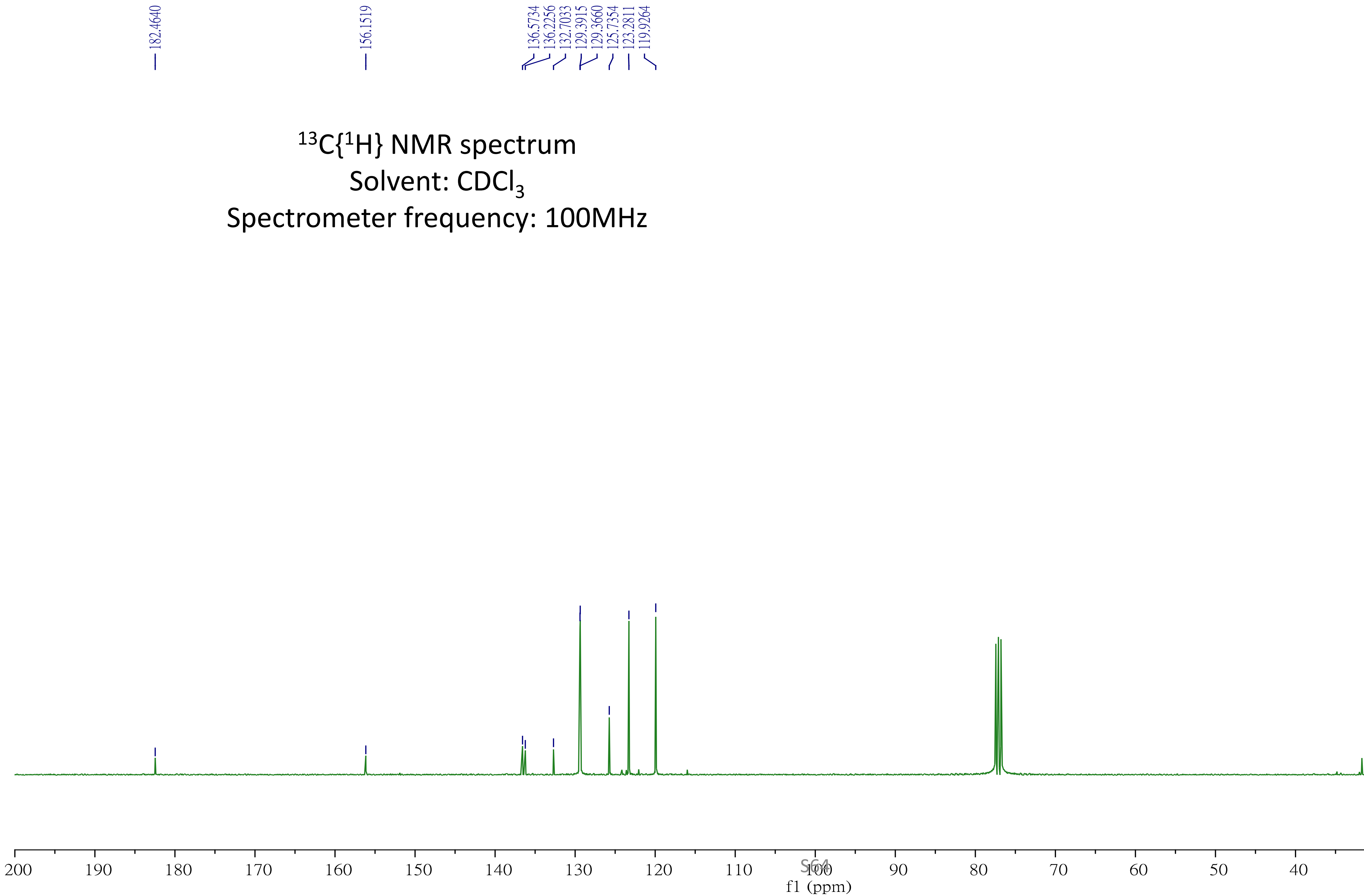

182.4640
156.1519
136.5734
136.2256
132.7033
129.3915
129.3660
125.7354
123.2811
119.9264
$^{13}C\{^{1}H\}$ NMR spectrum
Solvent: $CDCl_3$
Spectrometer frequency: 100MHz
200 190 180 170 160 150 140 130 120 110 100 90 80 70 60 50 40 30 20 10 0
f1 (ppm)

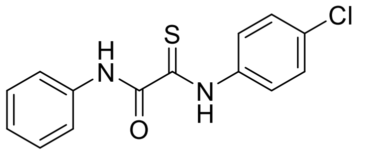

H
N
S
Cl
O
N
H

3aa

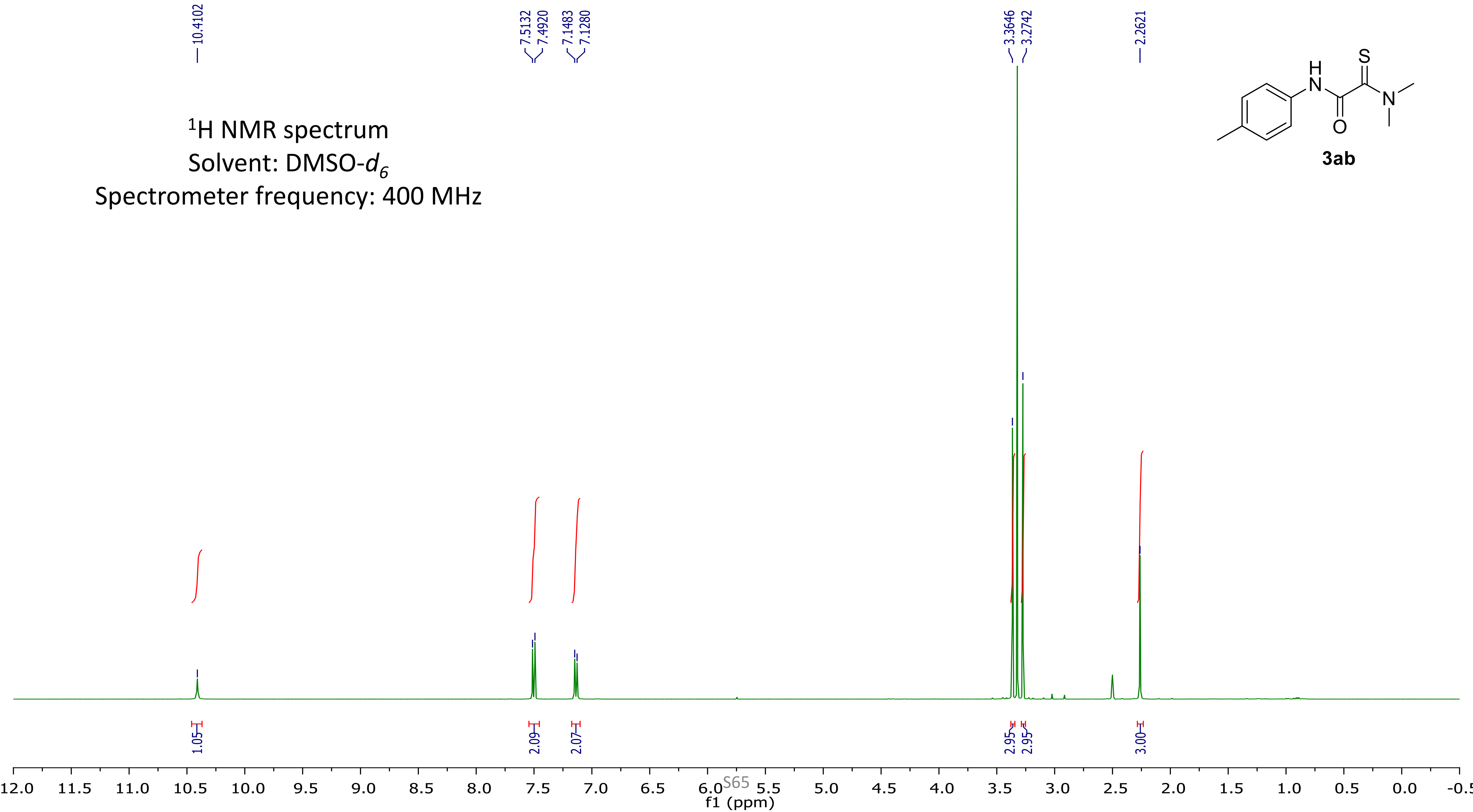

^{1}H NMR spectrum
Solvent: DMSO-d_6
Spectrometer frequency: 400 MHz
3ab
10.4102
7.5132
7.4920
7.1483
7.1280
3.3646
3.2742
2.2621
1.05
2.09
2.07
2.95
2.95
3.00
f1 (ppm)

$^{13}C\{^1H\}$ NMR spectrum
Solvent: DMSO-d_6
Spectrometer frequency: 100MHz

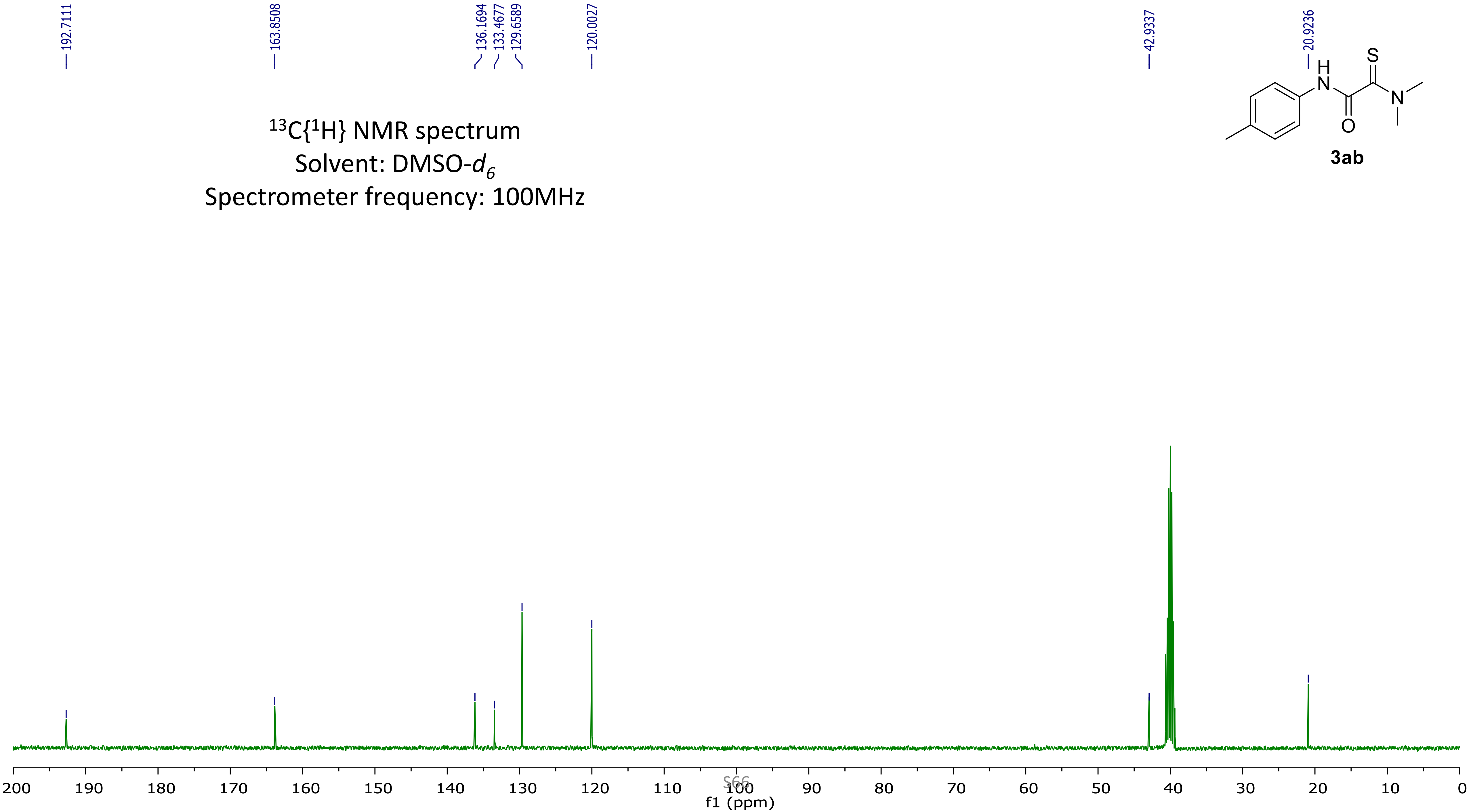

3ab

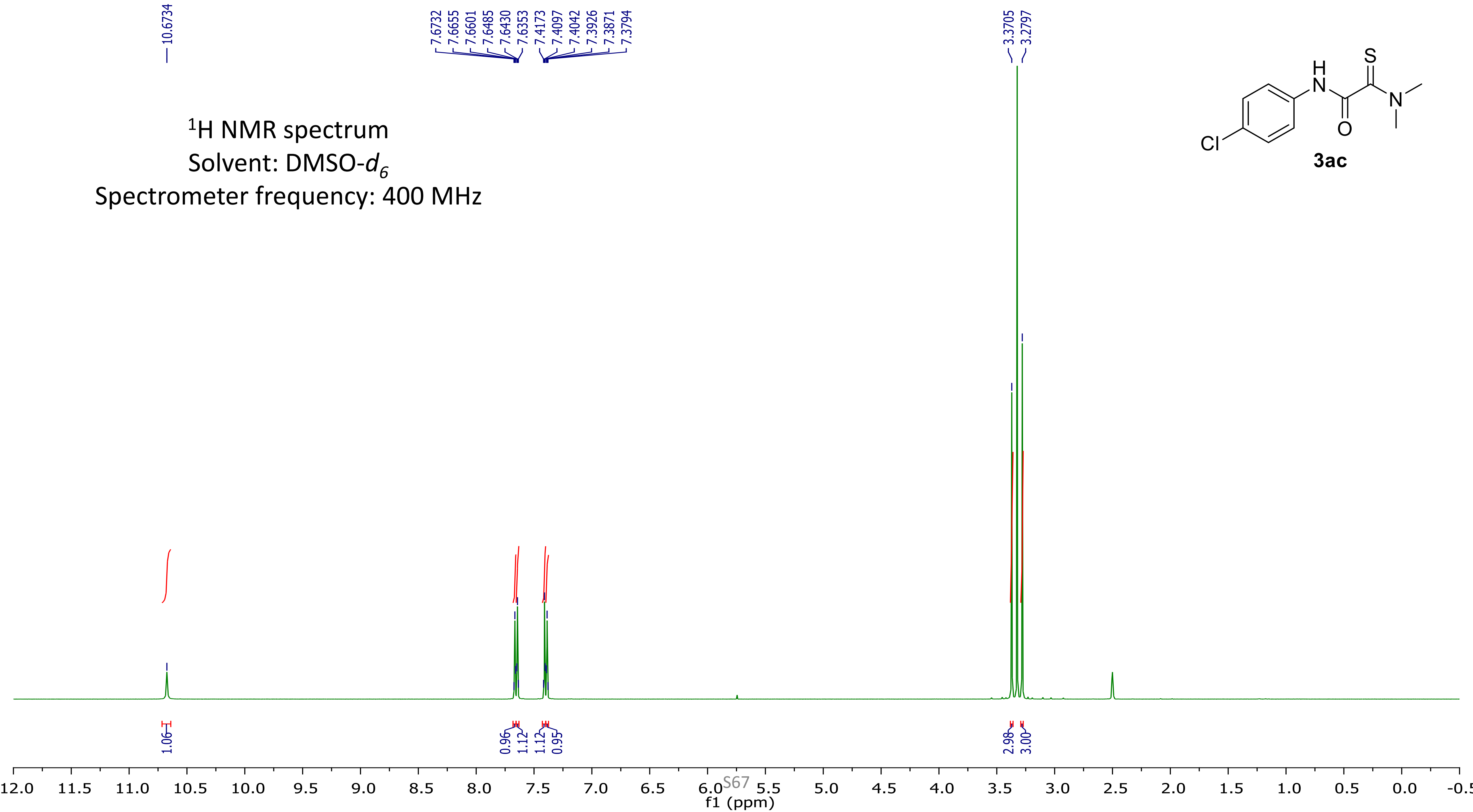
10.6734
7.6732
7.6655
7.6601
7.6485
7.6430
7.6353
7.4173
7.4097
7.4042
7.3926
7.3871
7.3794
3.3705
3.2797
¹H NMR spectrum
Solvent: DMSO-d6
Spectrometer frequency: 400 MHz
3ac
1.06
0.96
1.12
1.12
0.95
2.98
3.00
f1 (ppm)

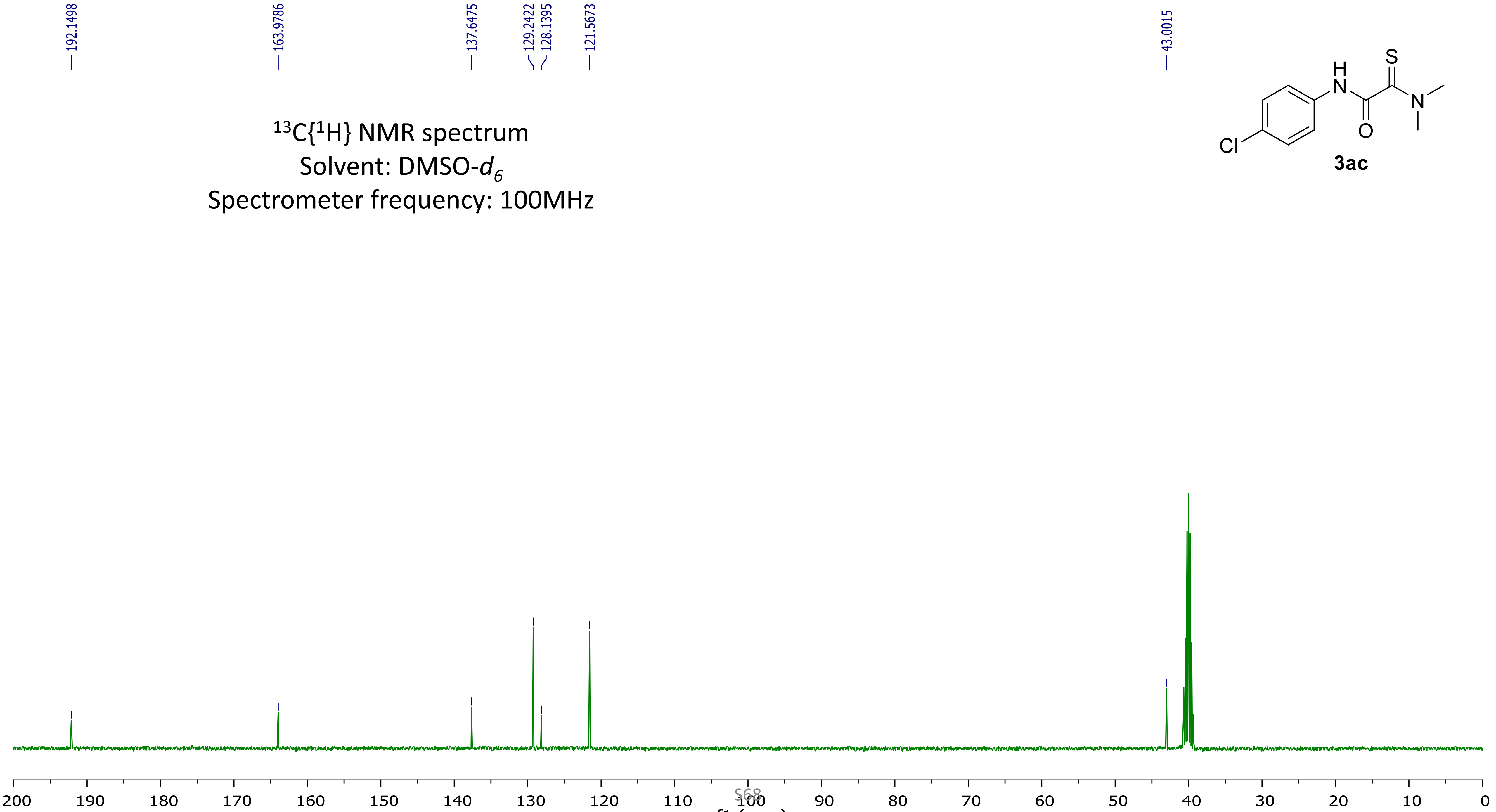

192.1498
163.9786
137.6475
129.2422
128.1395
121.5673
43.0015
$^{13}C\{^1H\}$ NMR spectrum
Solvent: DMSO-d_6
Spectrometer frequency: 100MHz
200 190 180 170 160 150 140 130 120 110 100 90 80 70 60 50 40 30 20 10 0
f1 (ppm)

3ac

^{1}H NMR spectrum
Solvent: $CDCl_3$
Spectrometer frequency: 400 MHz

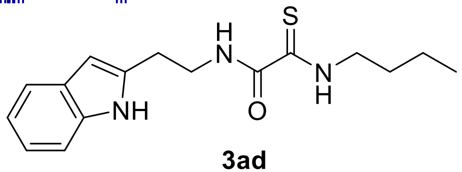

3ad

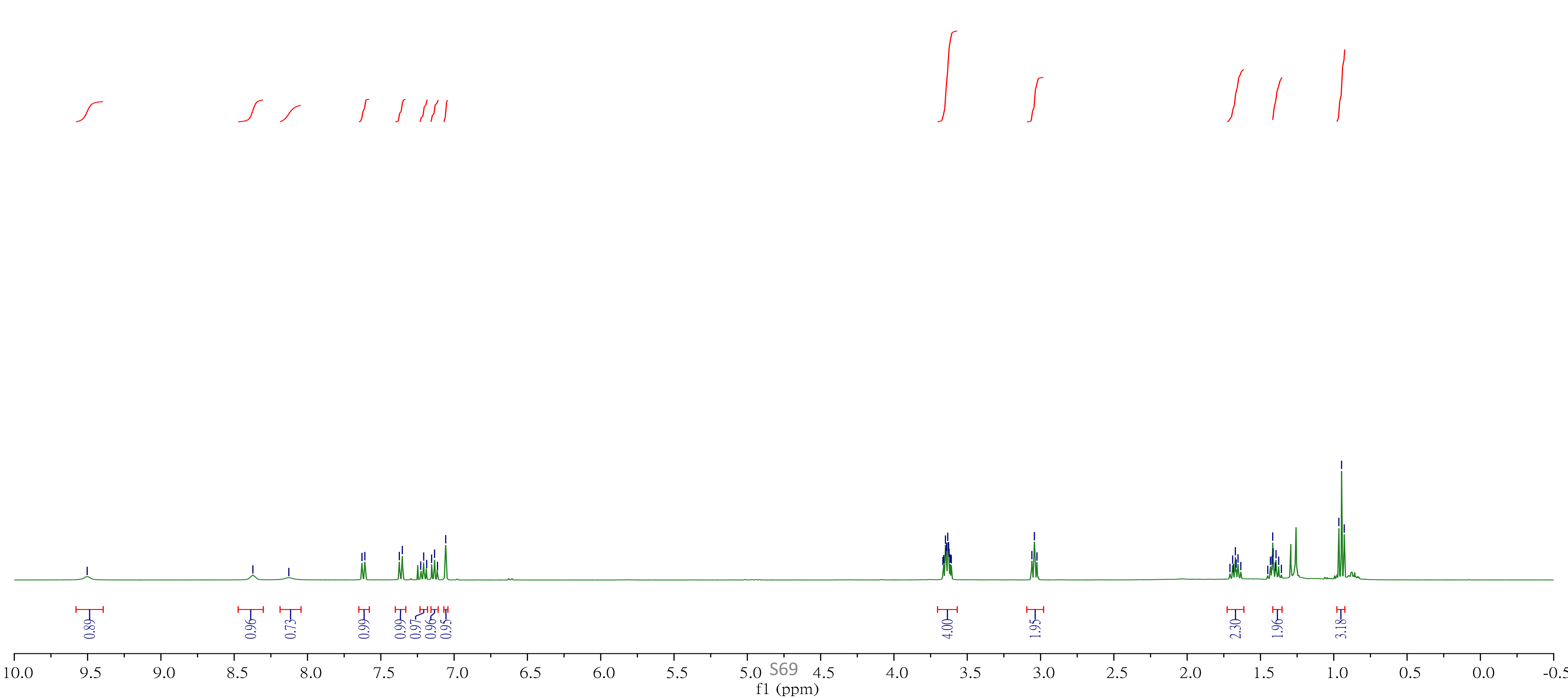

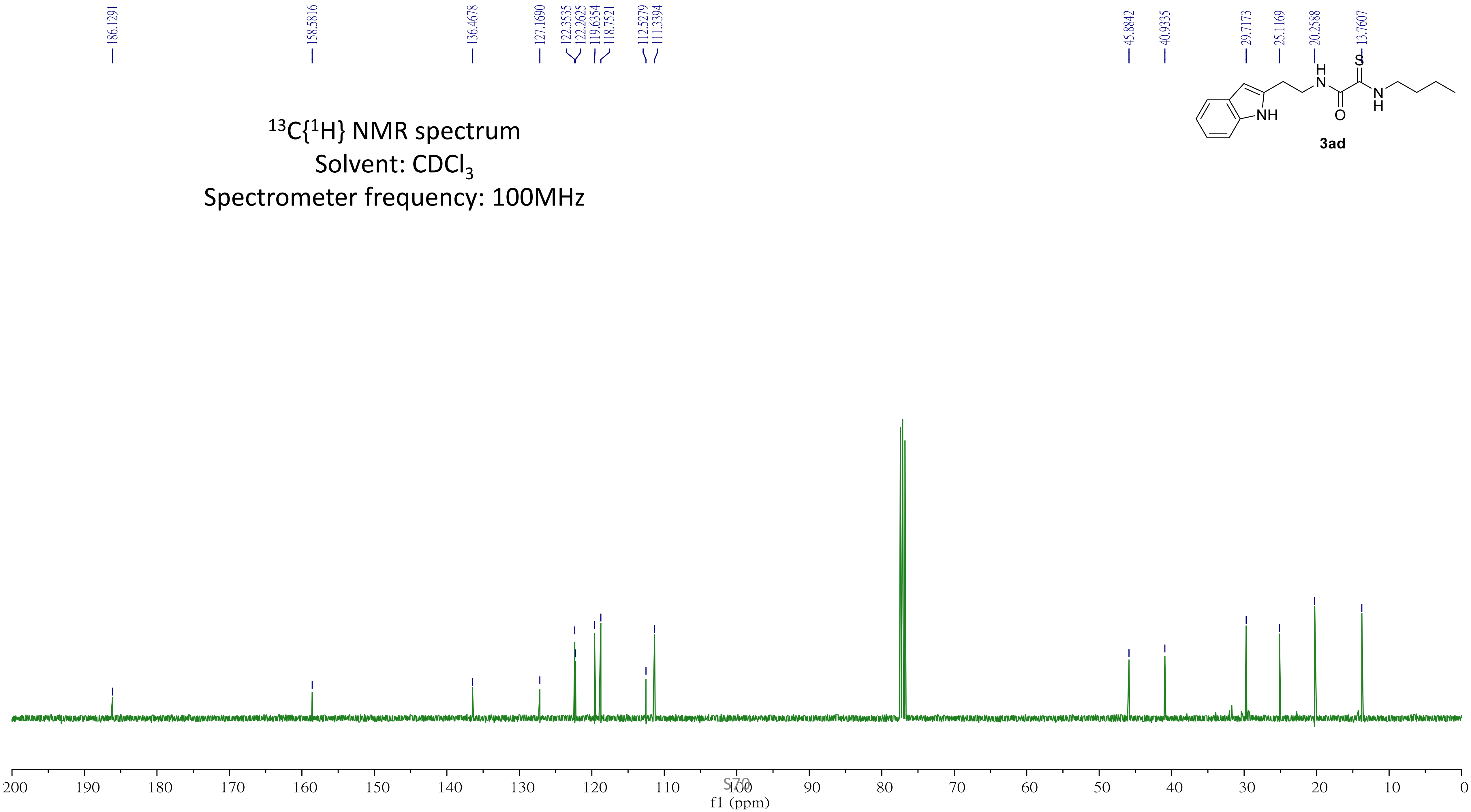

186.1291
158.5816
136.4678
127.1690
122.3535
122.2625
119.6354
118.7521
112.5279
111.3394
45.8842
40.9335
29.7173
25.1169
20.2588
13.7607
$^{13}C\{^1H\}$ NMR spectrum
Solvent: $CDCl_3$
Spectrometer frequency: 100MHz
3ad
200 190 180 170 160 150 140 130 120 110 100 90 80 70 60 50 40 30 20 10 0
f1 (ppm)

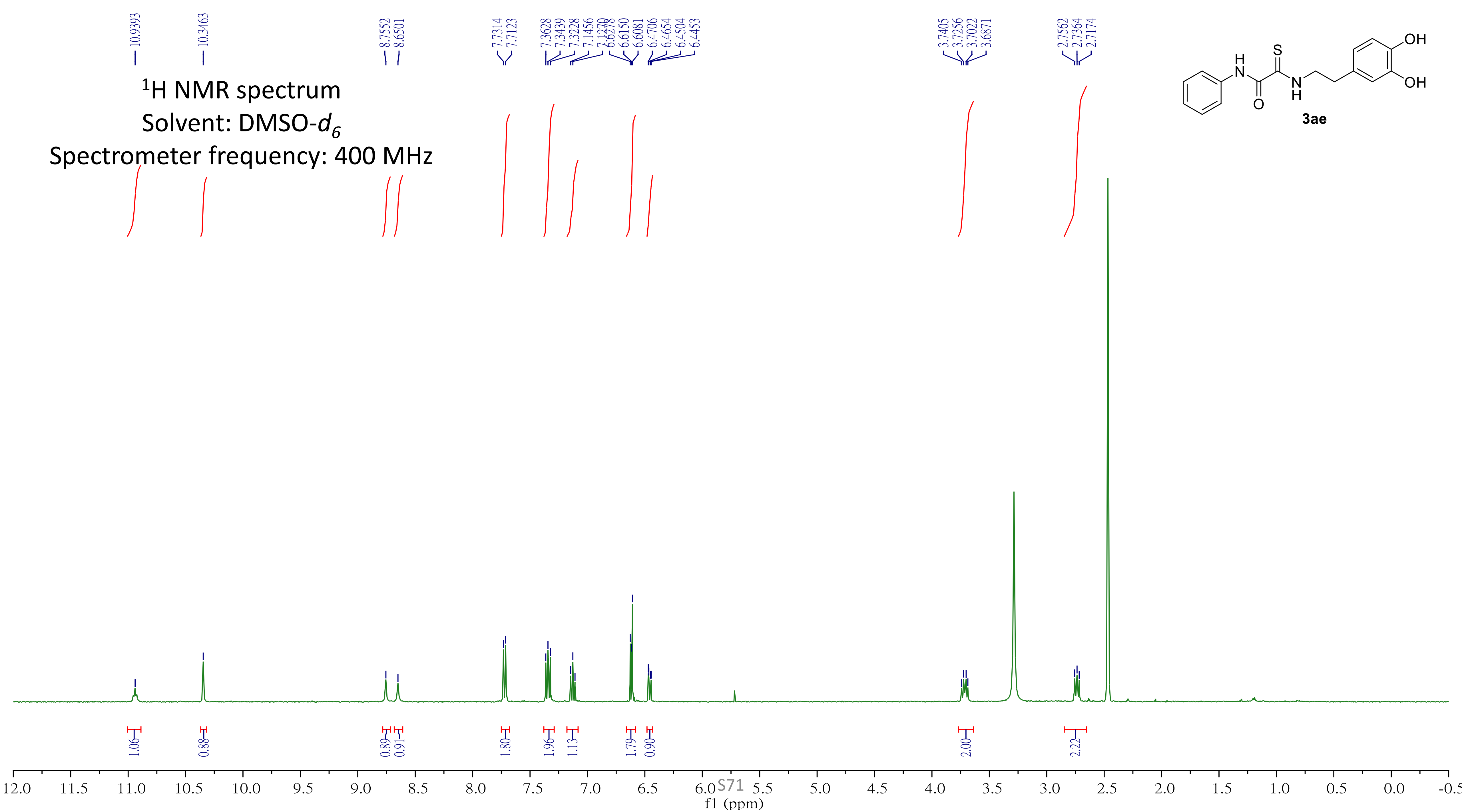

1H NMR spectrum
Solvent: DMSO-d6
Spectrometer frequency: 400 MHz
3ae
f1 (ppm)

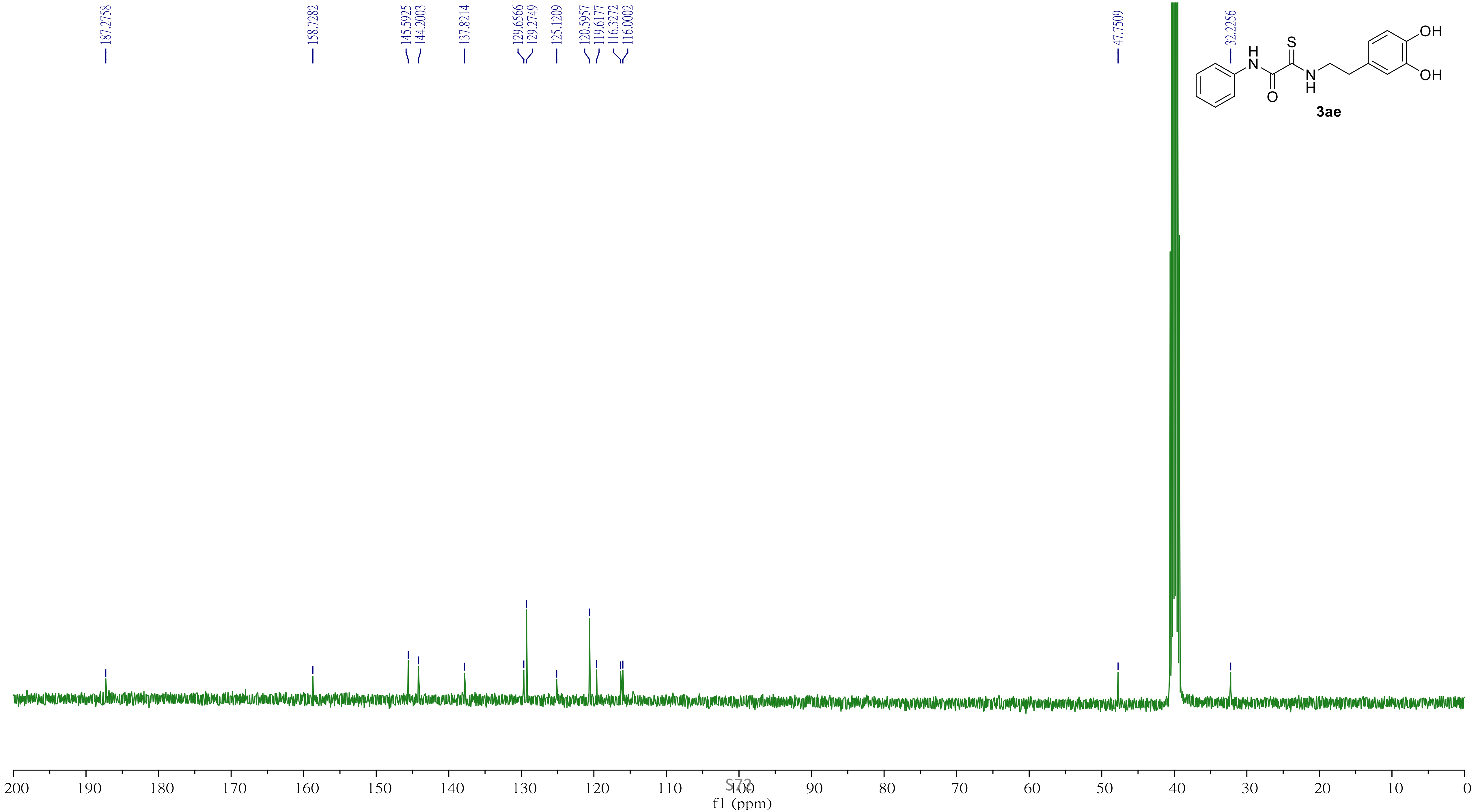
187.2758
158.7282
145.5925
144.2003
137.8214
129.6566
129.2749
125.1209
120.5957
119.6177
116.3272
116.0002
47.7509
32.2256
OH
OH
H
N
S
N
H
O
3ae
200 190 180 170 160 150 140 130 120 110 100 90 80 70 60 50 40 30 20 10 0
f1 (ppm)

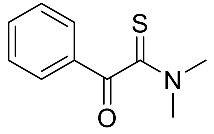

5a

^{1}H NMR spectrum

Solvent: DMSO-d_6

Spectrometer frequency: 400 MHz

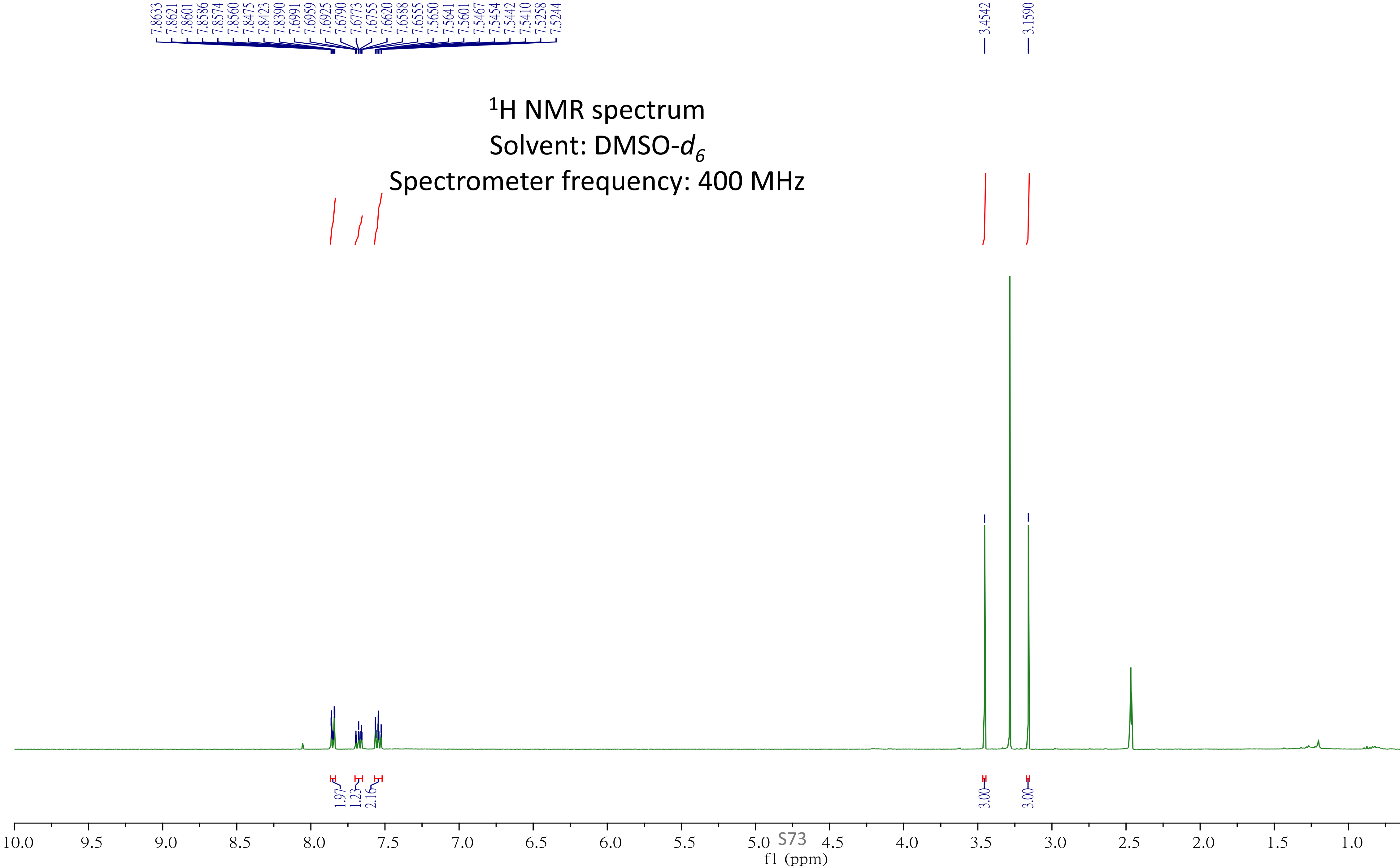

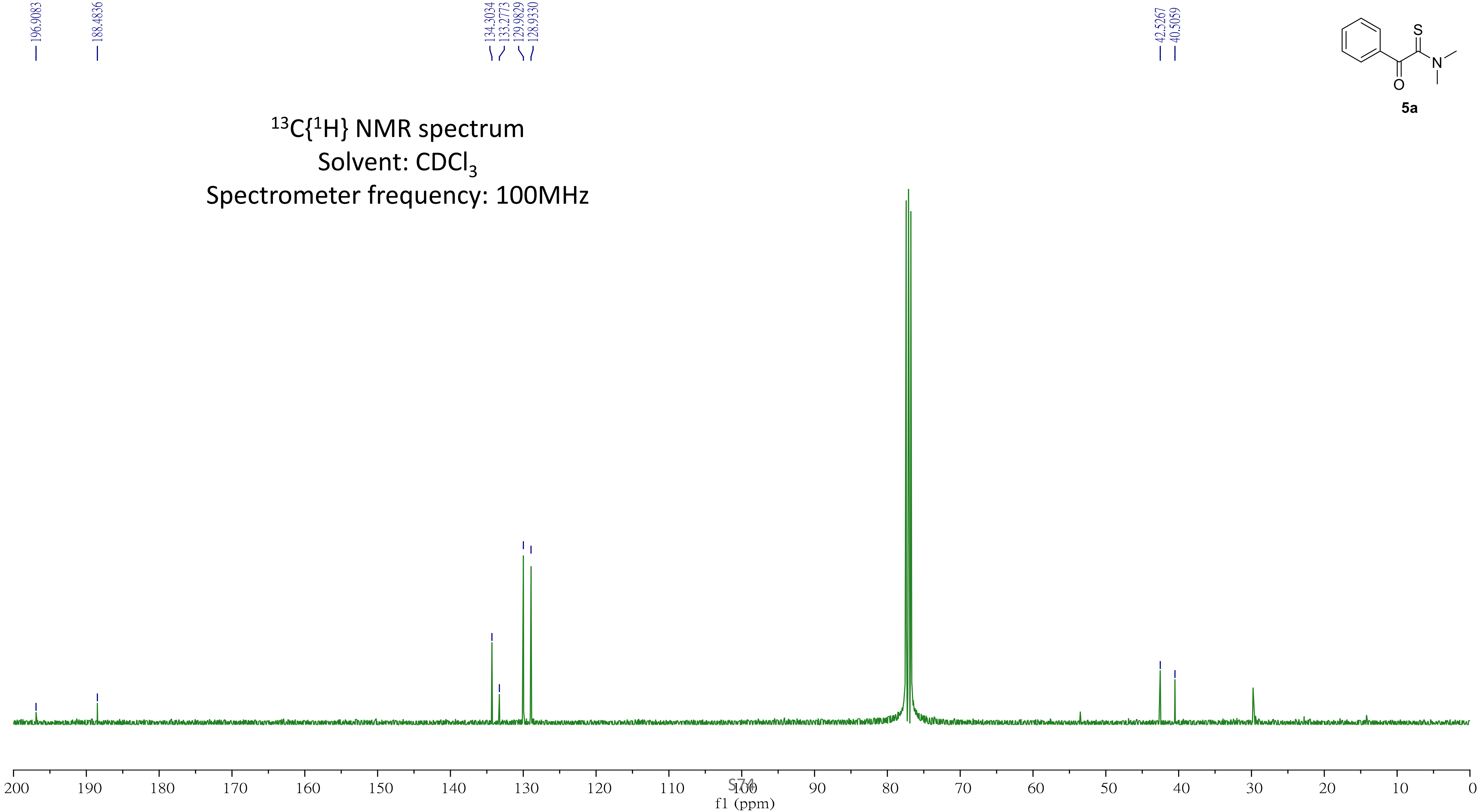

5a

$^{13}C\{^1H\}$ NMR spectrum
Solvent: $CDCl_3$
Spectrometer frequency: 100MHz

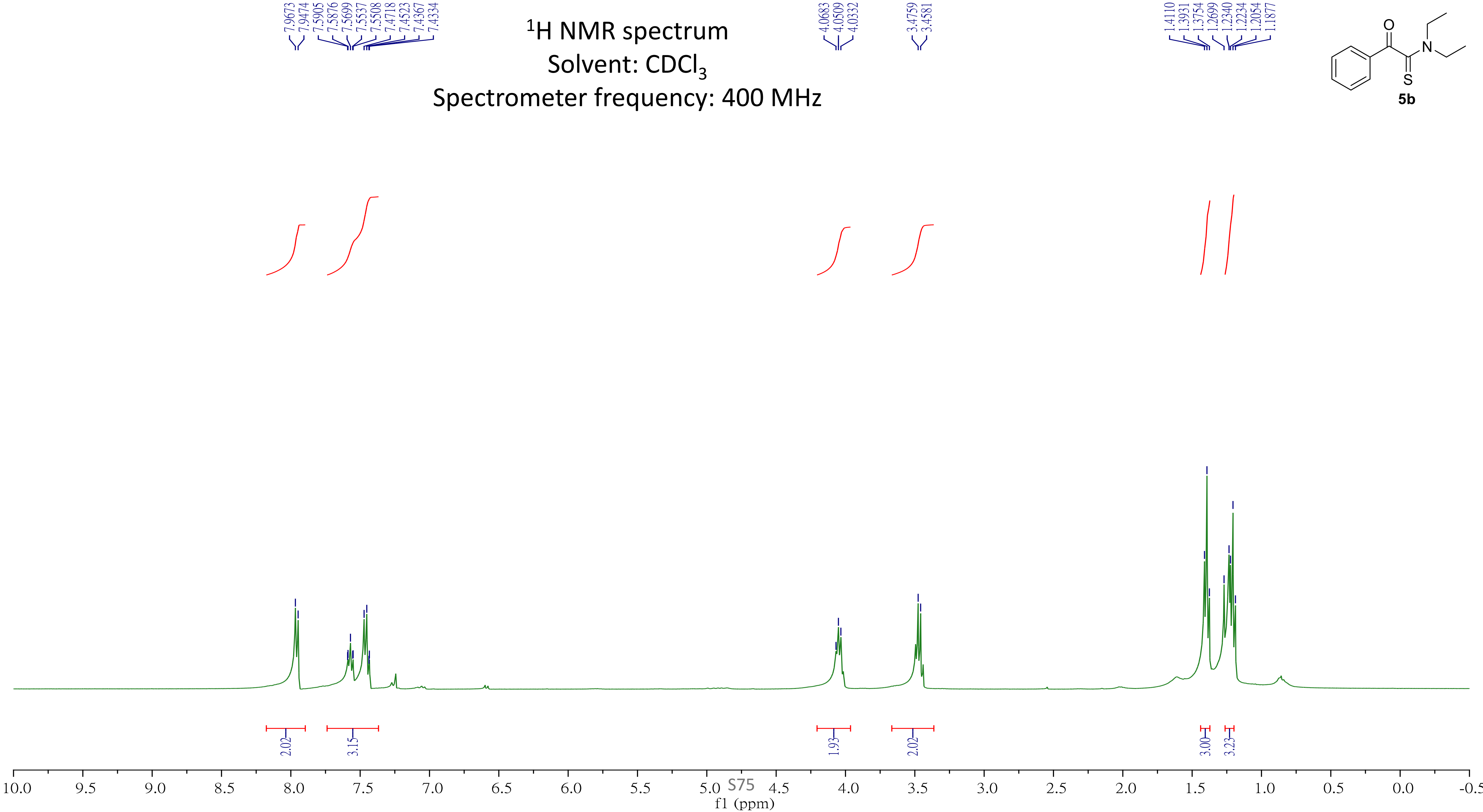

^{1}H NMR spectrum
Solvent: $CDCl_3$
Spectrometer frequency: 400 MHz
5b
f1 (ppm)

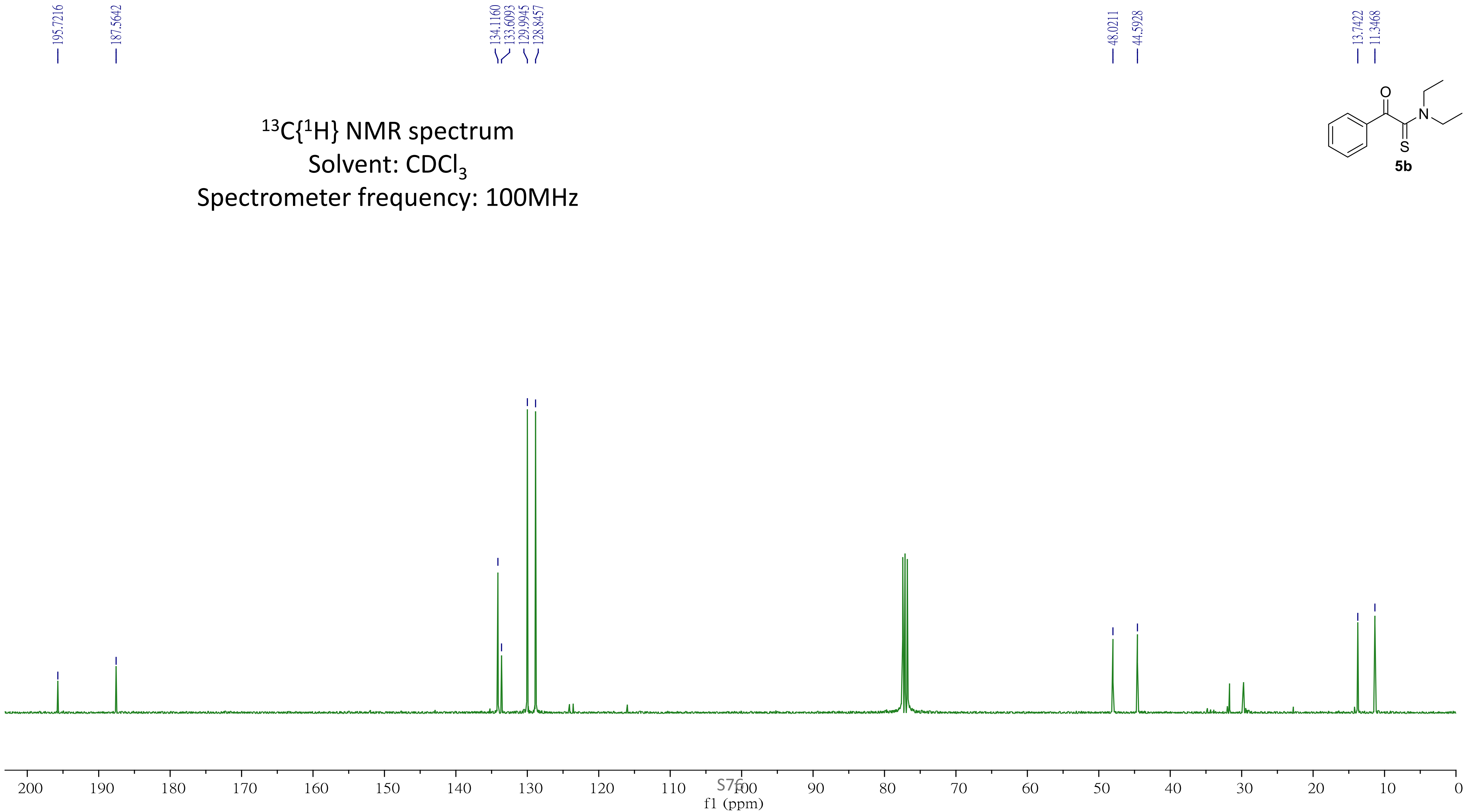
195.7216
187.5642
134.1160
133.6093
129.9945
128.8457
48.0211
44.5928
13.7422
11.3468
$^{13}C\{^1H\}$ NMR spectrum
Solvent: $CDCl_3$
Spectrometer frequency: 100MHz
O
N
S
5b
200
190
180
170
160
150
140
130
120
110
100
90
80
70
60
50
40
30
20
10
0
f1 (ppm)

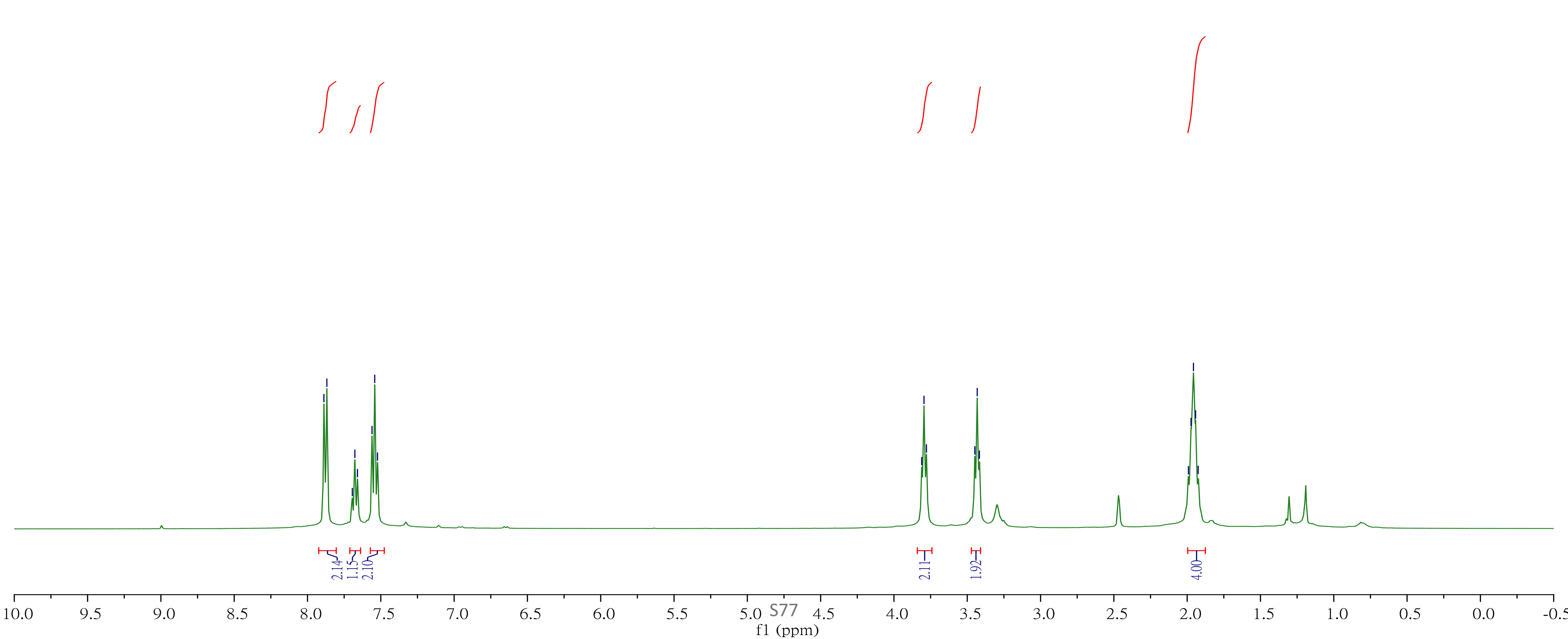
7.8881
7.8679
7.6942
7.6769
7.6593
7.5600
7.5415
7.5225
3.8106
3.7948
3.7782
3.4483
3.4320
3.4170
1.9913
1.9734
1.9572
1.9424
1.9249
^{1}H NMR spectrum
Solvent: DMSO-d_6
Spectrometer frequency: 400 MHz
2.14
1.15
2.10
2.11
1.92
4.00
10.0 9.5 9.0 8.5 8.0 7.5 7.0 6.5 6.0 5.5 5.0 4.5 4.0 3.5 3.0 2.5 2.0 1.5 1.0 0.5 0.0 -0.5
f1 (ppm)

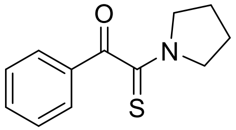
O
N
S

5c

$^{13}C\{^{1}H\}$ NMR spectrum
Solvent: DMSO-d_6
Spectrometer frequency: 100MHz

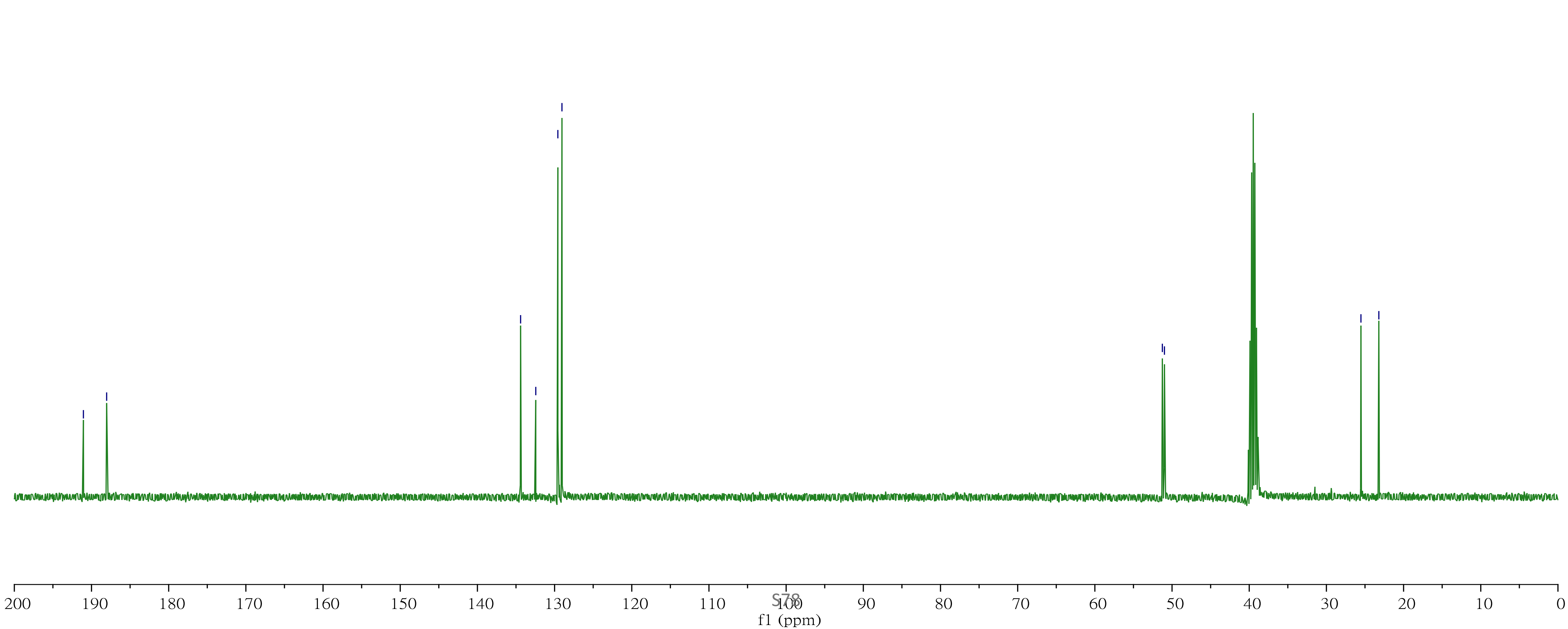

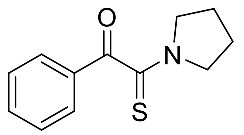

5c

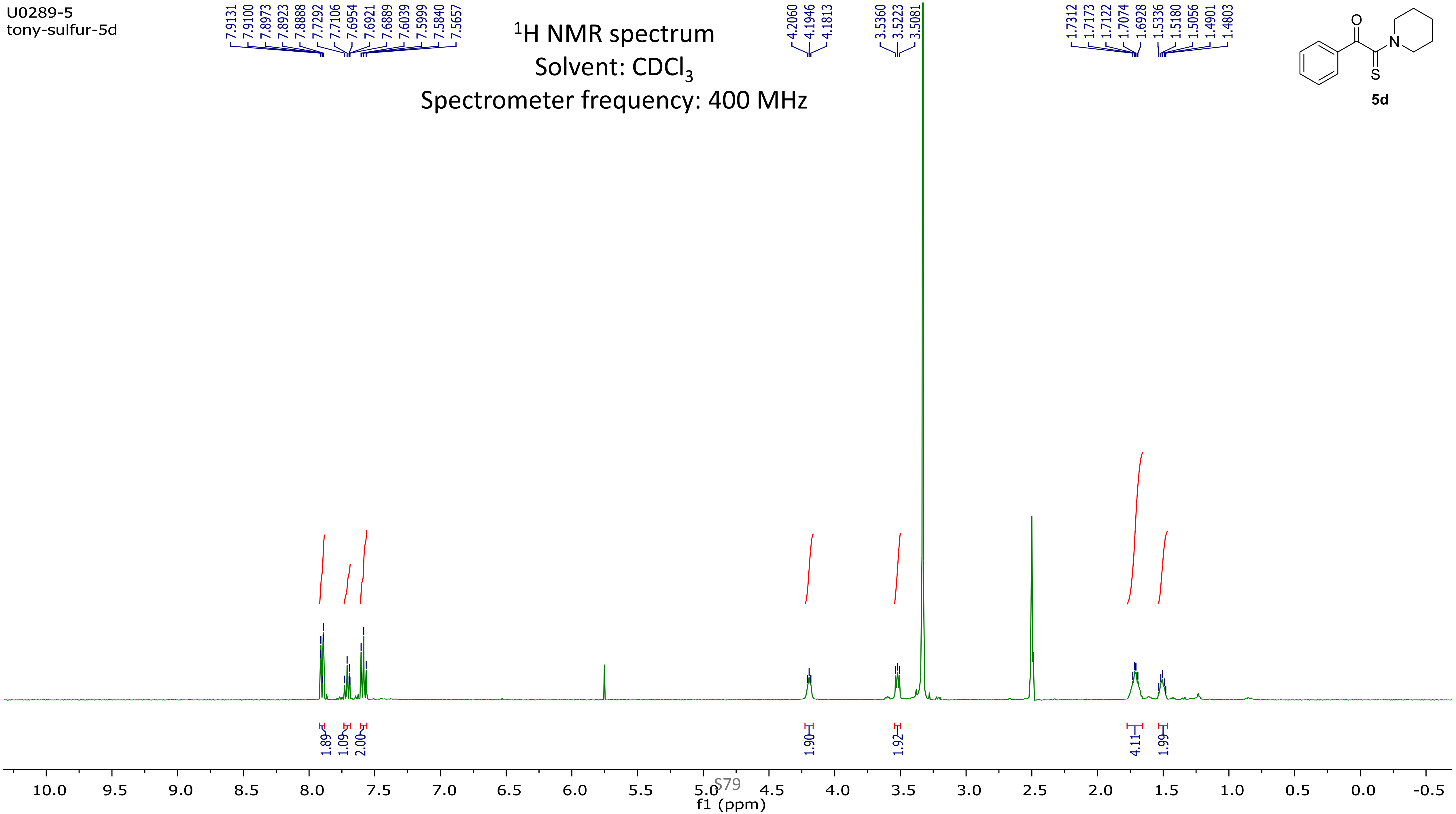
U0289-5
tony-sulfur-5d
1H NMR spectrum
Solvent: CDCl3
Spectrometer frequency: 400 MHz
7.9131 7.9100 7.8973 7.8923 7.8888 7.7292 7.7106 7.6954 7.6921 7.6889 7.6039 7.5999 7.5840 7.5657
4.2060 4.1946 4.1813
3.5360 3.5223 3.5081
1.7312 1.7173 1.7122 1.7074 1.6928 1.5336 1.5180 1.5056 1.4901 1.4803
O
N
S
5d
1.89 1.09 2.00 1.90 1.92 4.11 1.99
10.0 9.5 9.0 8.5 8.0 7.5 7.0 6.5 6.0 5.5 5.0 4.5 4.0 3.5 3.0 2.5 2.0 1.5 1.0 0.5 0.0 -0.5
f1 (ppm)

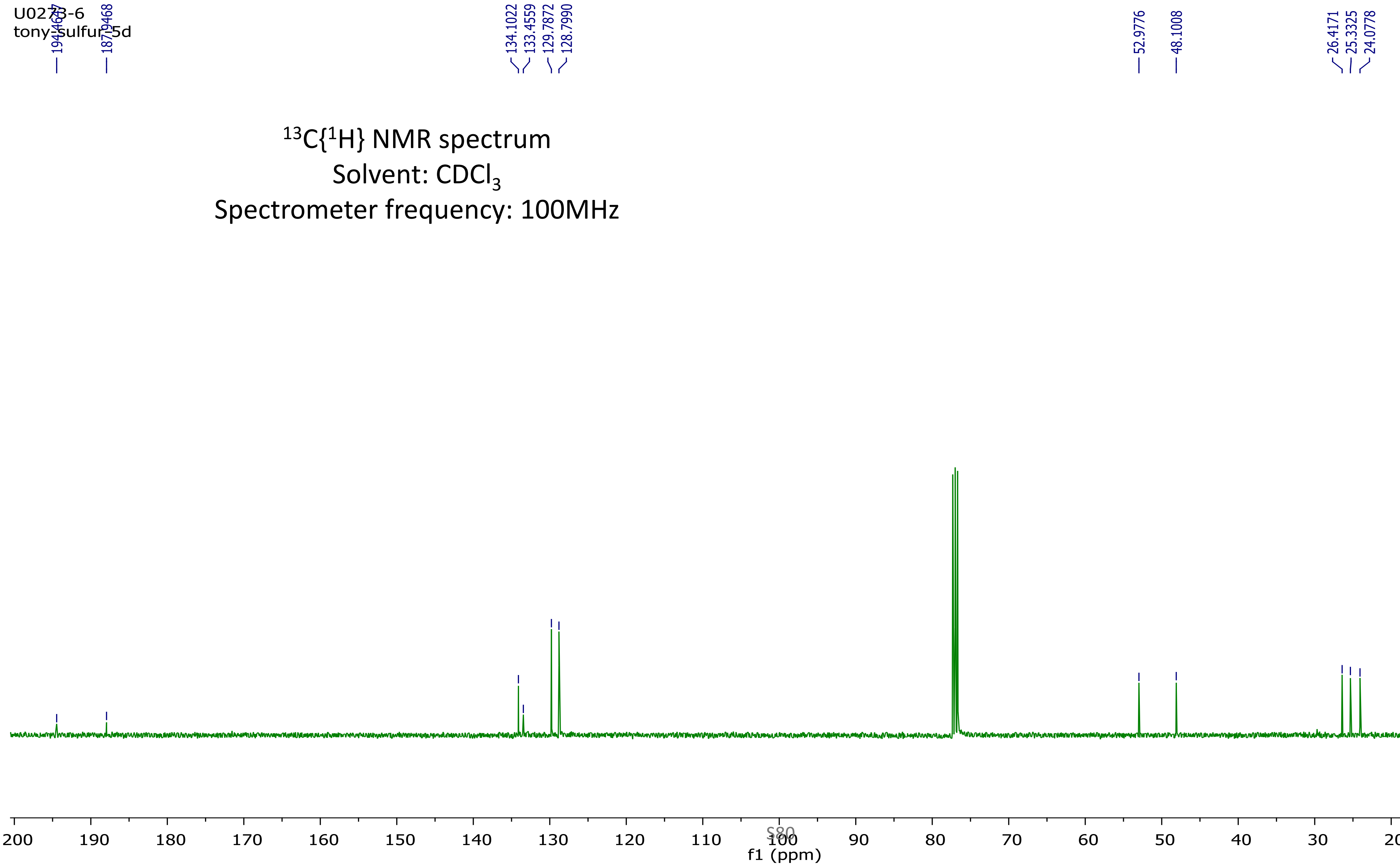
U0273-6
tony-sulfur-5d
194.4647
187.9468
134.1022
133.4559
129.7872
128.7990
52.9776
48.1008
26.4171
25.3325
24.0778
$^{13}C\{^1H\}$ NMR spectrum
Solvent: $CDCl_3$
Spectrometer frequency: 100MHz
200
190
180
170
160
150
140
130
120
110
100
90
80
70
60
50
40
30
20
10
0
f1 (ppm)

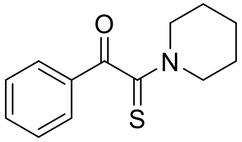
O
N
S

5d

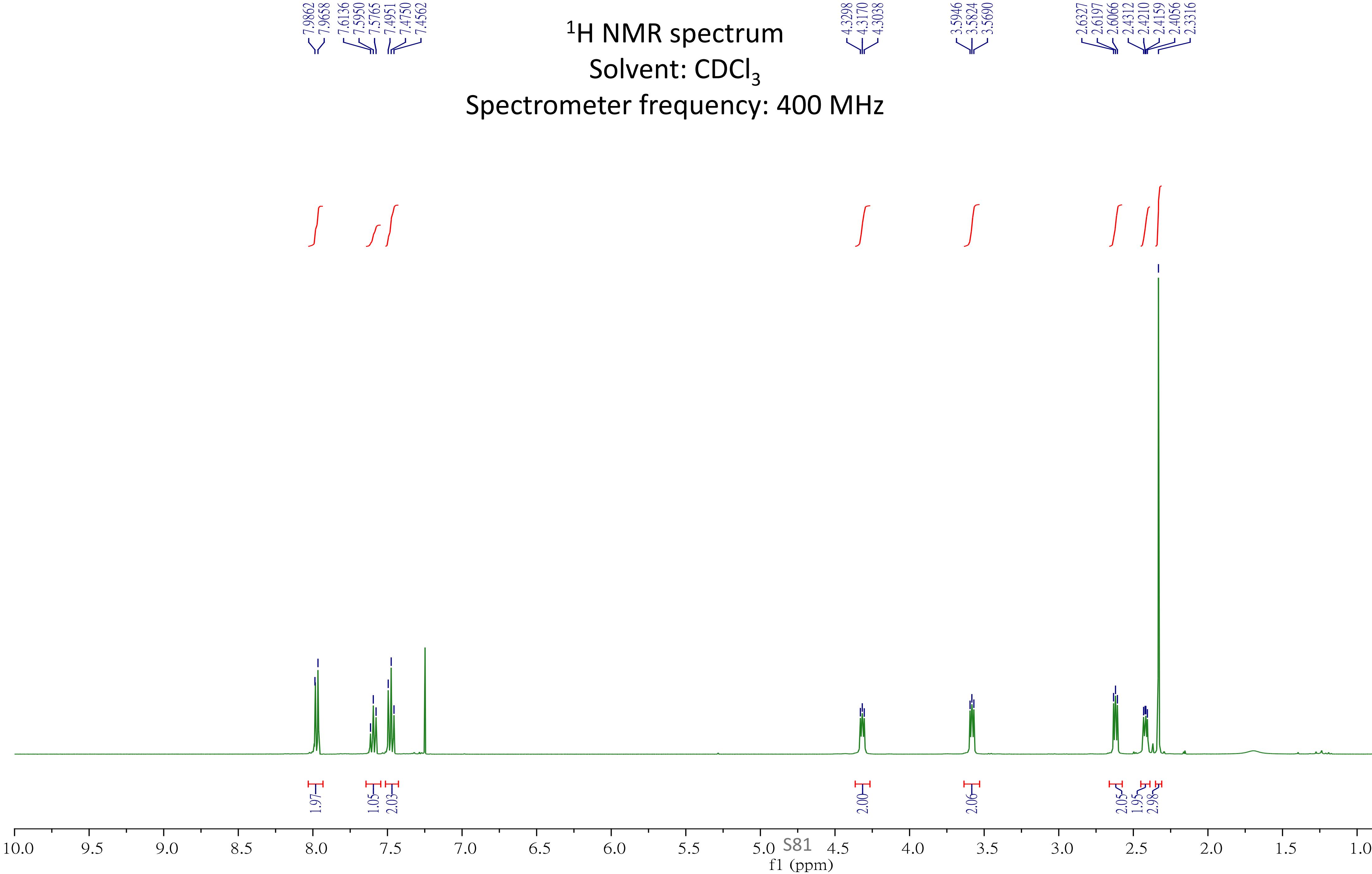

^{1}H NMR spectrum
Solvent: $CDCl_3$
Spectrometer frequency: 400 MHz
7.9862
7.9658
7.6136
7.5950
7.5765
7.4951
7.4750
7.4562
4.3298
4.3170
4.3038
3.5946
3.5824
3.5690
2.6327
2.6197
2.6066
2.4312
2.4210
2.4159
2.4056
2.3316
1.97
1.05
2.03
2.00
2.06
2.05
1.95
2.98
10.0 9.5 9.0 8.5 8.0 7.5 7.0 6.5 6.0 5.5 5.0 4.5 4.0 3.5 3.0 2.5 2.0 1.5 1.0 0.5 0.0 -0.5
f1 (ppm)

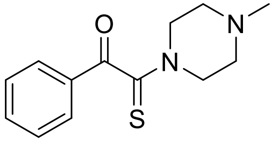

O
N
N
S

5e

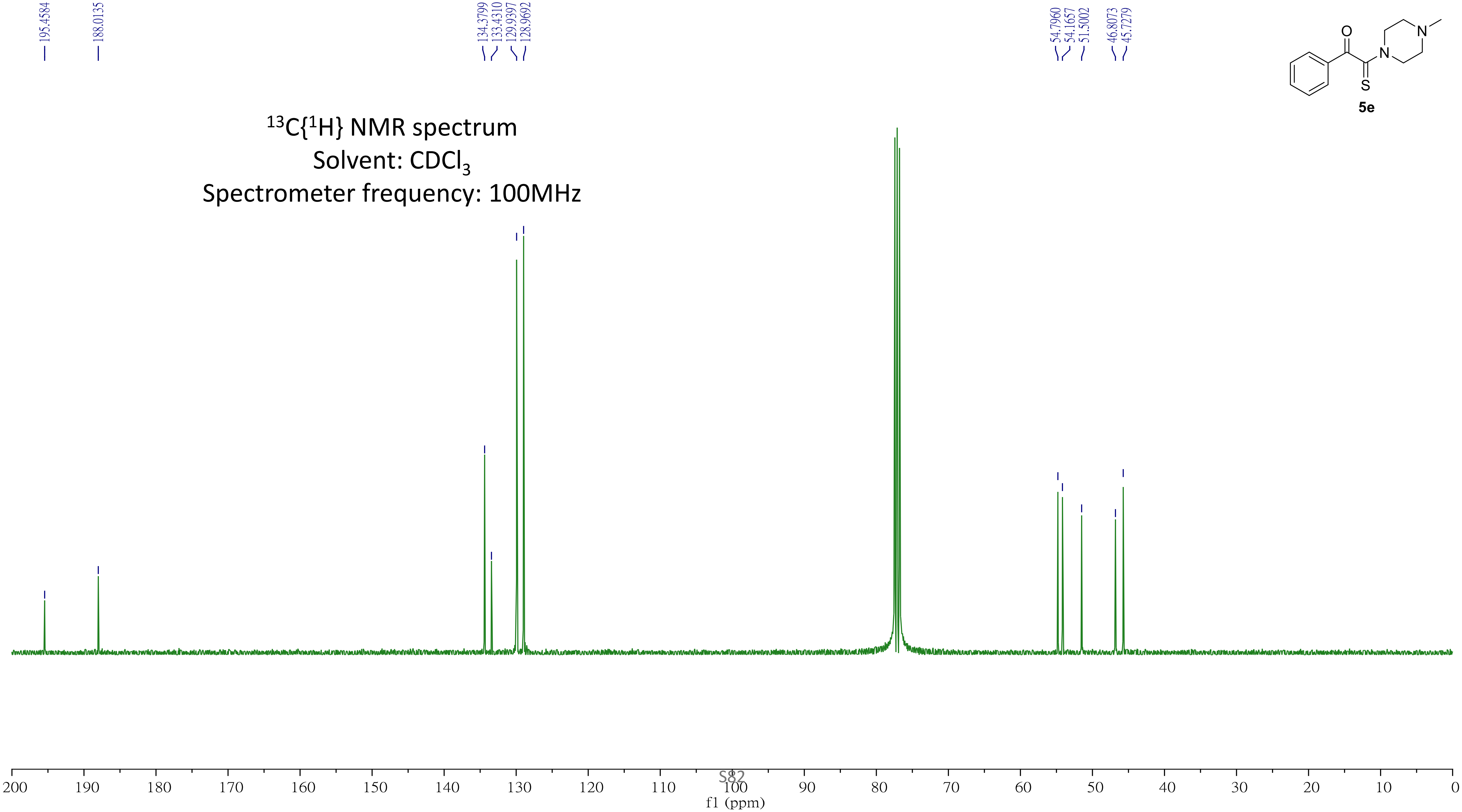

195.4584
188.0135
134.3799
133.4310
129.9397
128.9692
54.7960
54.1657
51.5002
46.8073
45.7279
$^{13}C\{^{1}H\}$ NMR spectrum
Solvent: $CDCl_3$
Spectrometer frequency: 100MHz
5e
f1 (ppm)

AJ-V-139
single_pulse

^{1}H NMR spectrum
Solvent: DMSO-d_6
Spectrometer frequency: 400 MHz

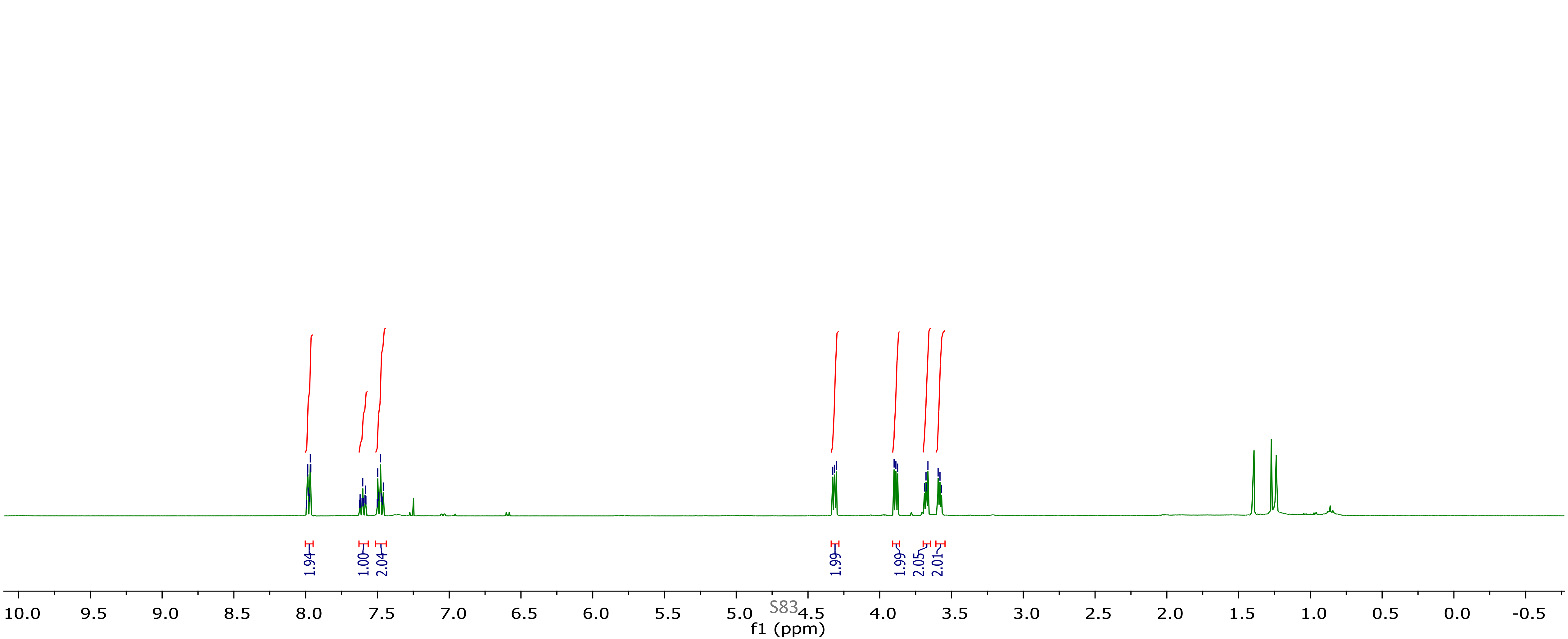

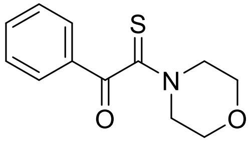

5f

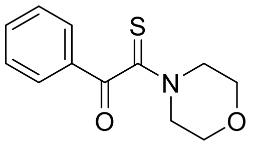

5f

$^{13}C\{^1H\}$ NMR spectrum
Solvent: $CDCl_3$
Spectrometer frequency: 100MHz

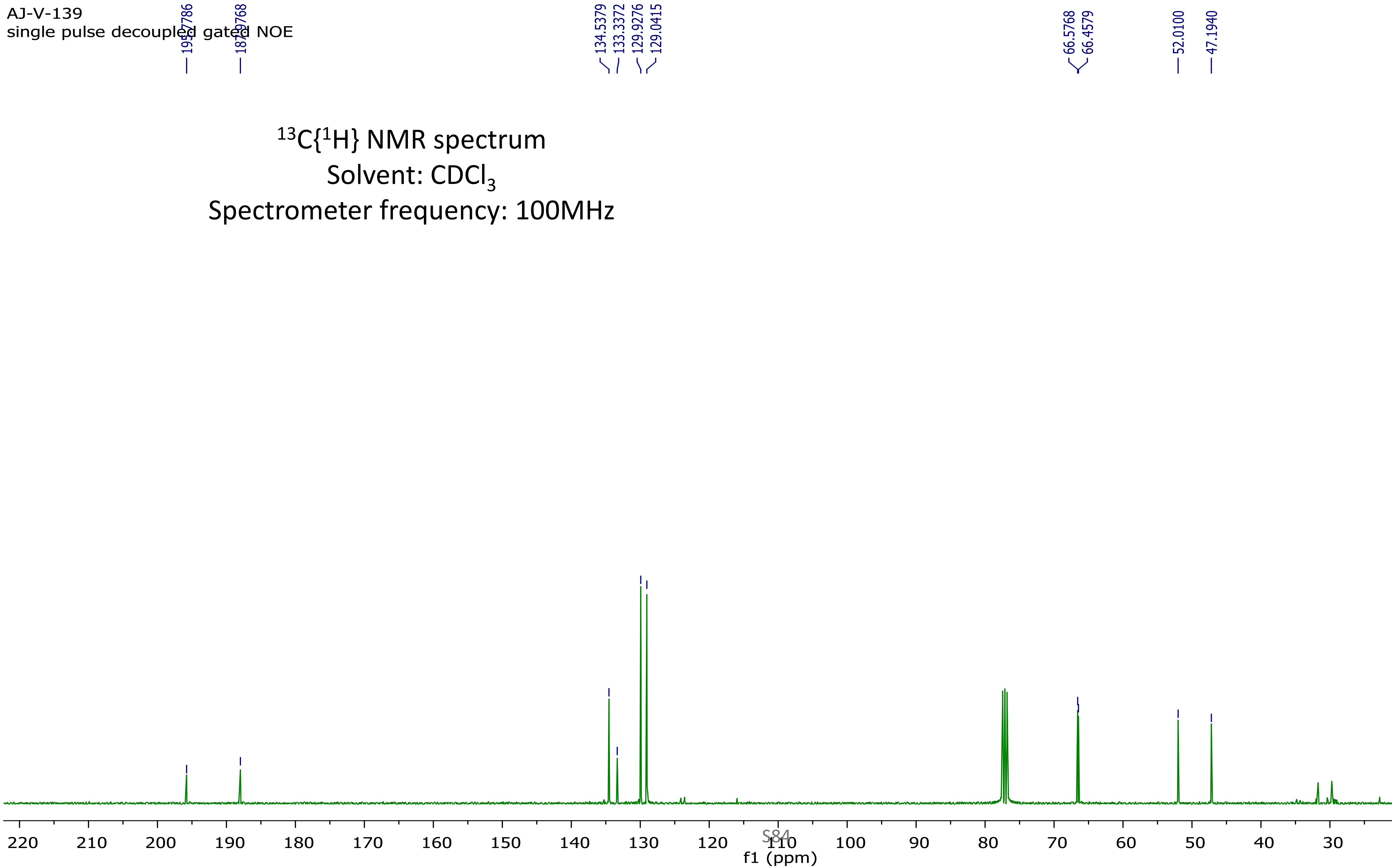

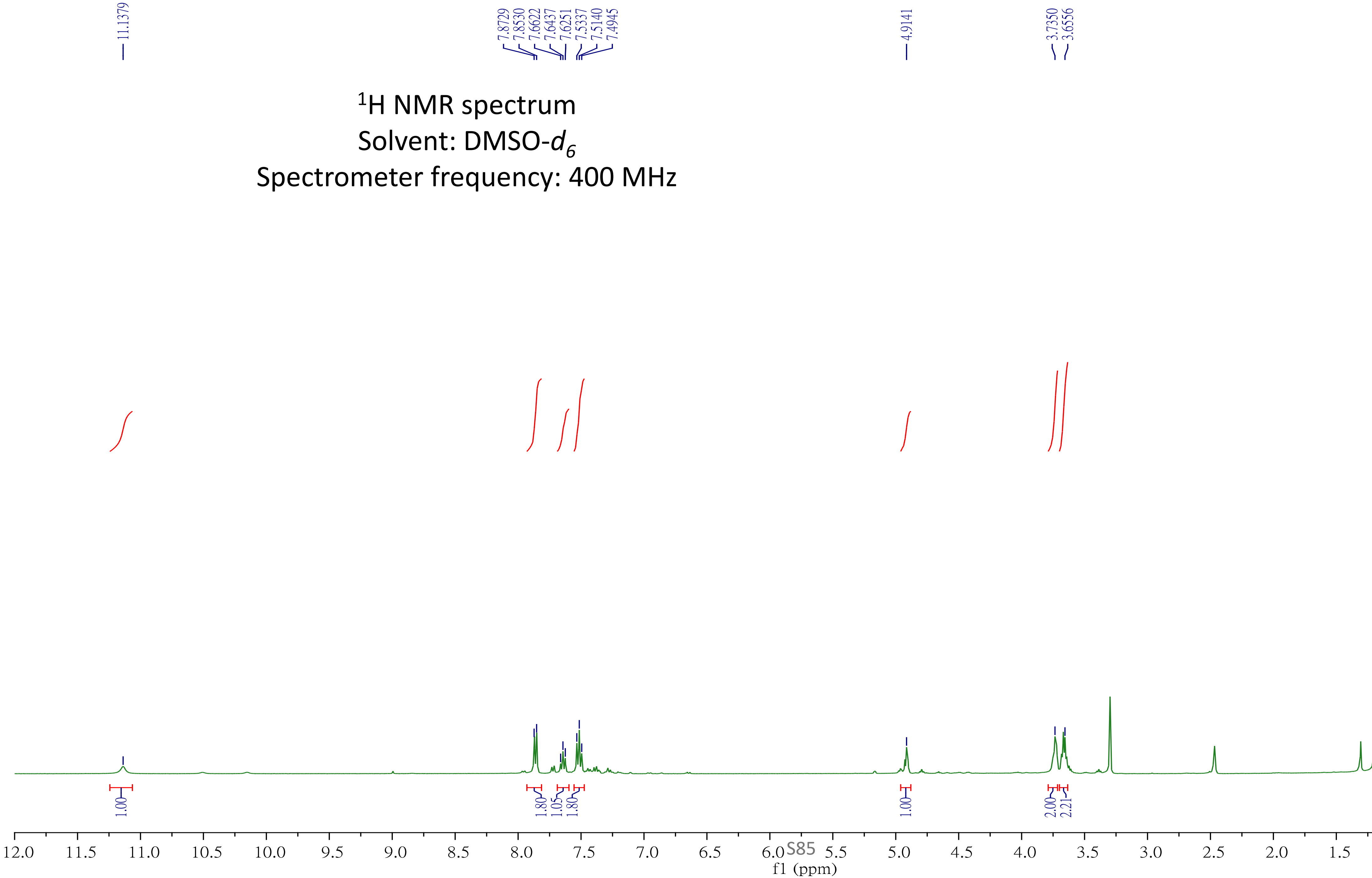

^{1}H NMR spectrum
Solvent: DMSO-d_6
Spectrometer frequency: 400 MHz
11.1379
7.8729
7.8530
7.6622
7.6437
7.6251
7.5337
7.5140
7.4945
4.9141
3.7350
3.6556
1.00
1.80
1.05
1.80
1.00
2.00
2.21
12.0 11.5 11.0 10.5 10.0 9.5 9.0 8.5 8.0 7.5 7.0 6.5 6.0 5.5 5.0 4.5 4.0 3.5 3.0 2.5 2.0 1.5 1.0 0.5 0.0 -0.5
f1 (ppm)

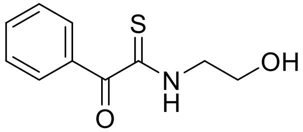

S
N
H
OH
O

5g

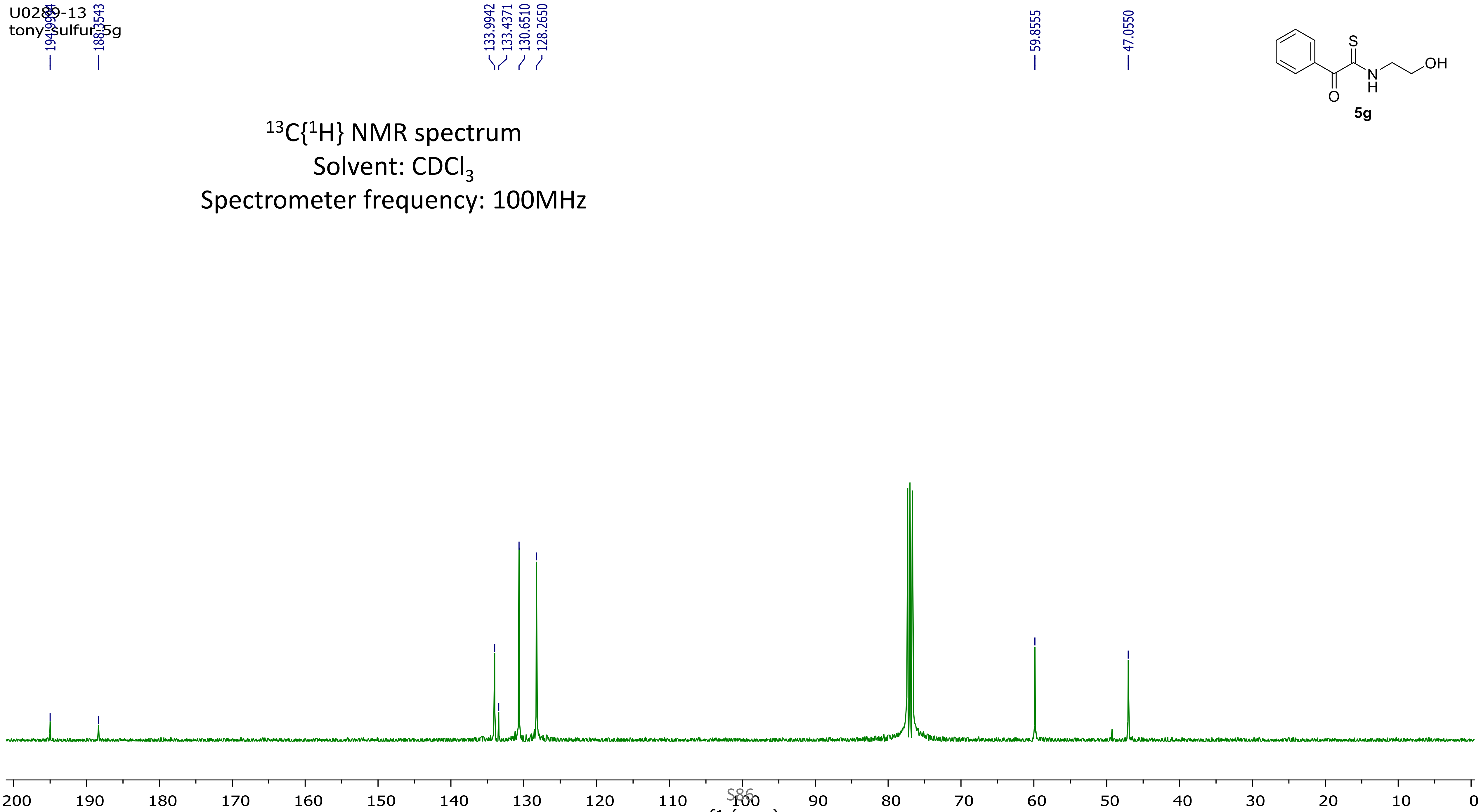
13C{1H} NMR spectrum
Solvent: CDCl3
Spectrometer frequency: 100MHz
133.9942
133.4371
130.6510
128.2650
59.8555
47.0550
S
OH
N
H
O
5g
f1 (ppm)
200
190
180
170
160
150
140
130
120
110
100
90
80
70
60
50
40
30
20
10
0

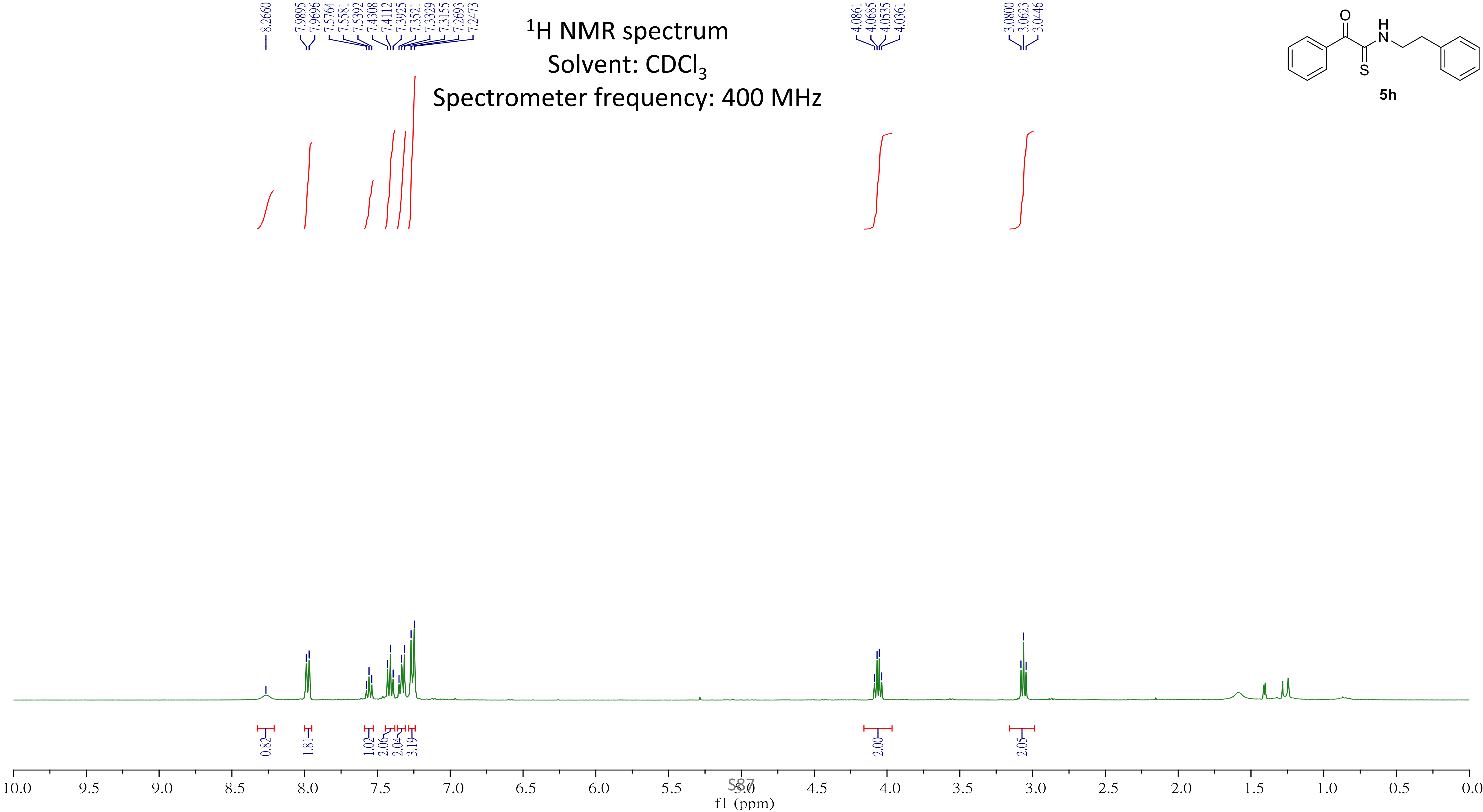

¹H NMR spectrum
Solvent: CDCl3
Spectrometer frequency: 400 MHz
8.2660
7.9895
7.9696
7.5764
7.5581
7.5392
7.4308
7.4112
7.3925
7.3521
7.3329
7.3155
7.2693
7.2473
4.0861
4.0685
4.0535
4.0361
3.0800
3.0623
3.0446
0.82
1.81
1.02
2.06
2.04
3.19
2.00
2.05
O
H
N
S
5h
10.0
9.5
9.0
8.5
8.0
7.5
7.0
6.5
6.0
5.5
5.0
4.5
4.0
3.5
3.0
2.5
2.0
1.5
1.0
0.5
0.0
f1 (ppm)

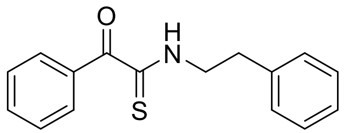

5h

$^{13}C\{^1H\}$ NMR spectrum
Solvent: $CDCl_3$
Spectrometer frequency: 100MHz

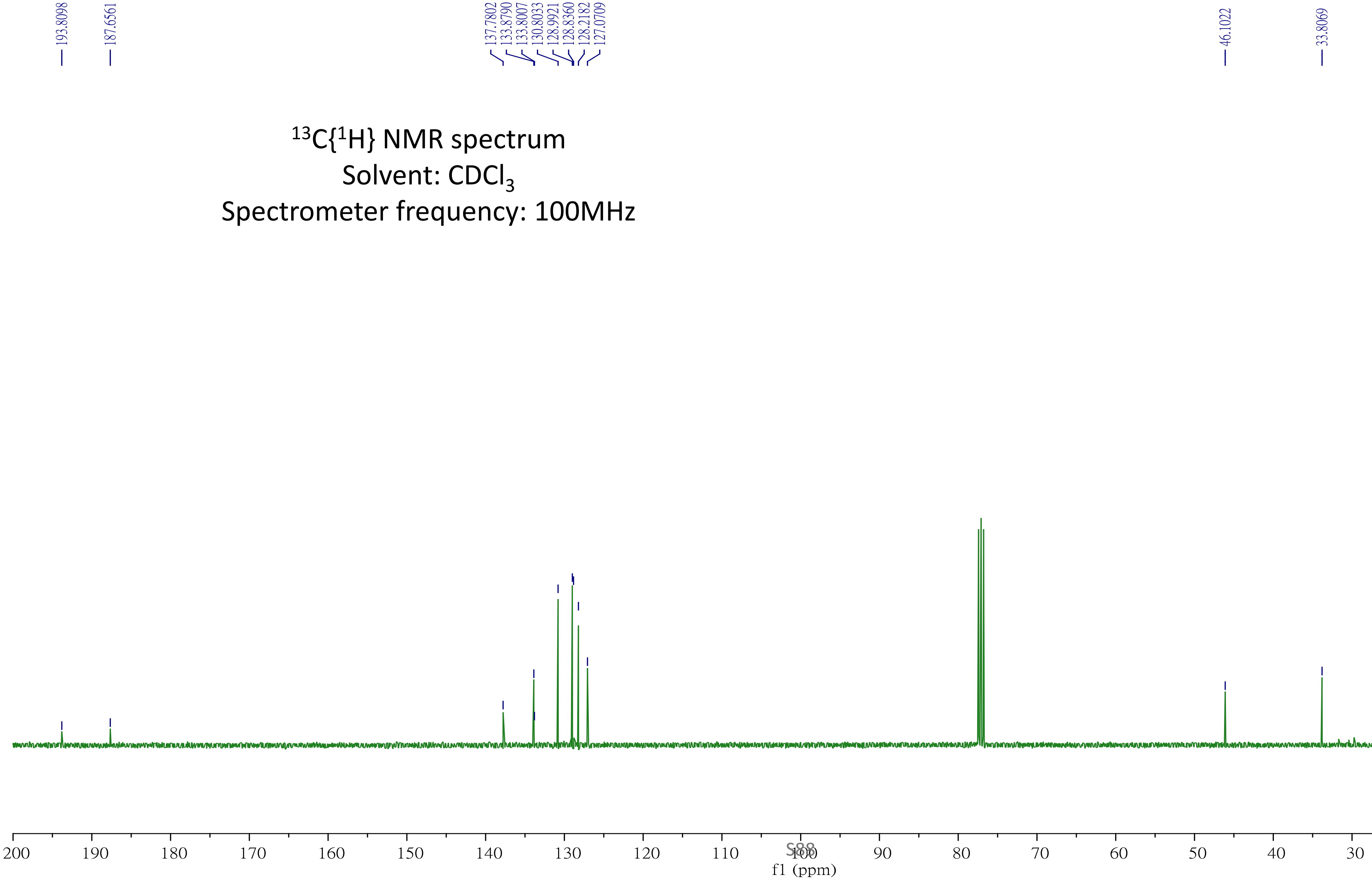

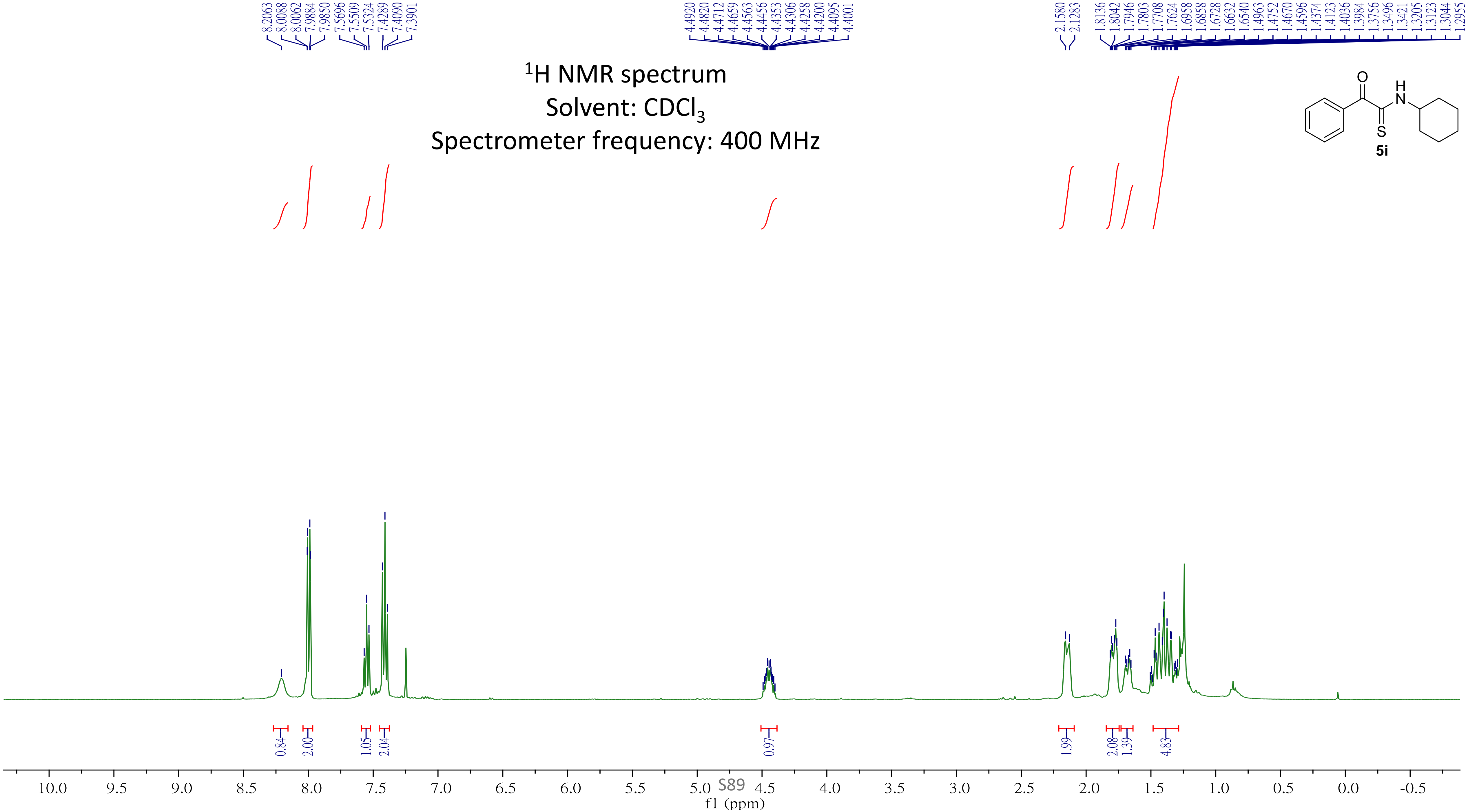

1H NMR spectrum
Solvent: CDCl3
Spectrometer frequency: 400 MHz
8.2063
8.0088
8.0062
7.9884
7.9850
7.5696
7.5509
7.5324
7.4289
7.4090
7.3901
4.4920
4.4820
4.4712
4.4659
4.4563
4.4456
4.4353
4.4306
4.4258
4.4200
4.4095
4.4001
2.1580
2.1283
1.8136
1.8042
1.7946
1.7803
1.7708
1.7624
1.6958
1.6858
1.6728
1.6632
1.6540
1.4963
1.4752
1.4670
1.4596
1.4374
1.4123
1.4036
1.3984
1.3756
1.3496
1.3421
1.3205
1.3123
1.3044
1.2955
0.84
2.00
1.05
2.04
0.97
1.99
2.08
1.39
4.83
10.0
9.5
9.0
8.5
8.0
7.5
7.0
6.5
6.0
5.5
5.0
4.5
4.0
3.5
3.0
2.5
2.0
1.5
1.0
0.5
0.0
-0.5
f1 (ppm)
O
H
N
S

5i

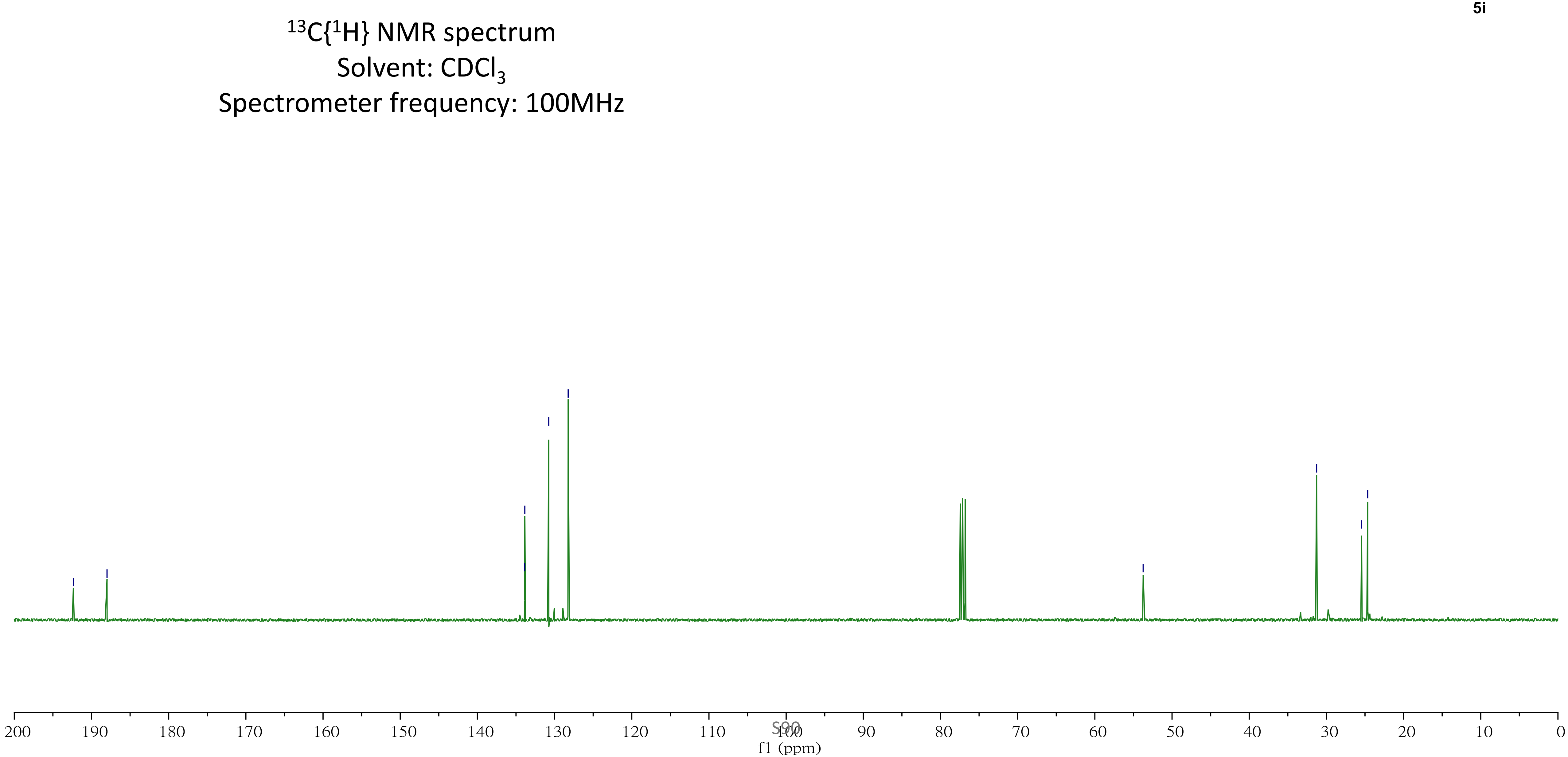
192.3513
187.9887
133.8749
133.8558
130.7535
128.2397
53.7413
31.2718
25.4392
24.6485
$^{13}C\{^{1}H\}$ NMR spectrum
Solvent: $CDCl_3$
Spectrometer frequency: 100MHz
200 190 180 170 160 150 140 130 120 110 100 90 80 70 60 50 40 30 20 10 0
f1 (ppm)

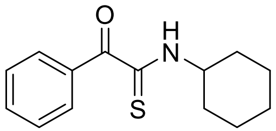
O
H
N
S

5i

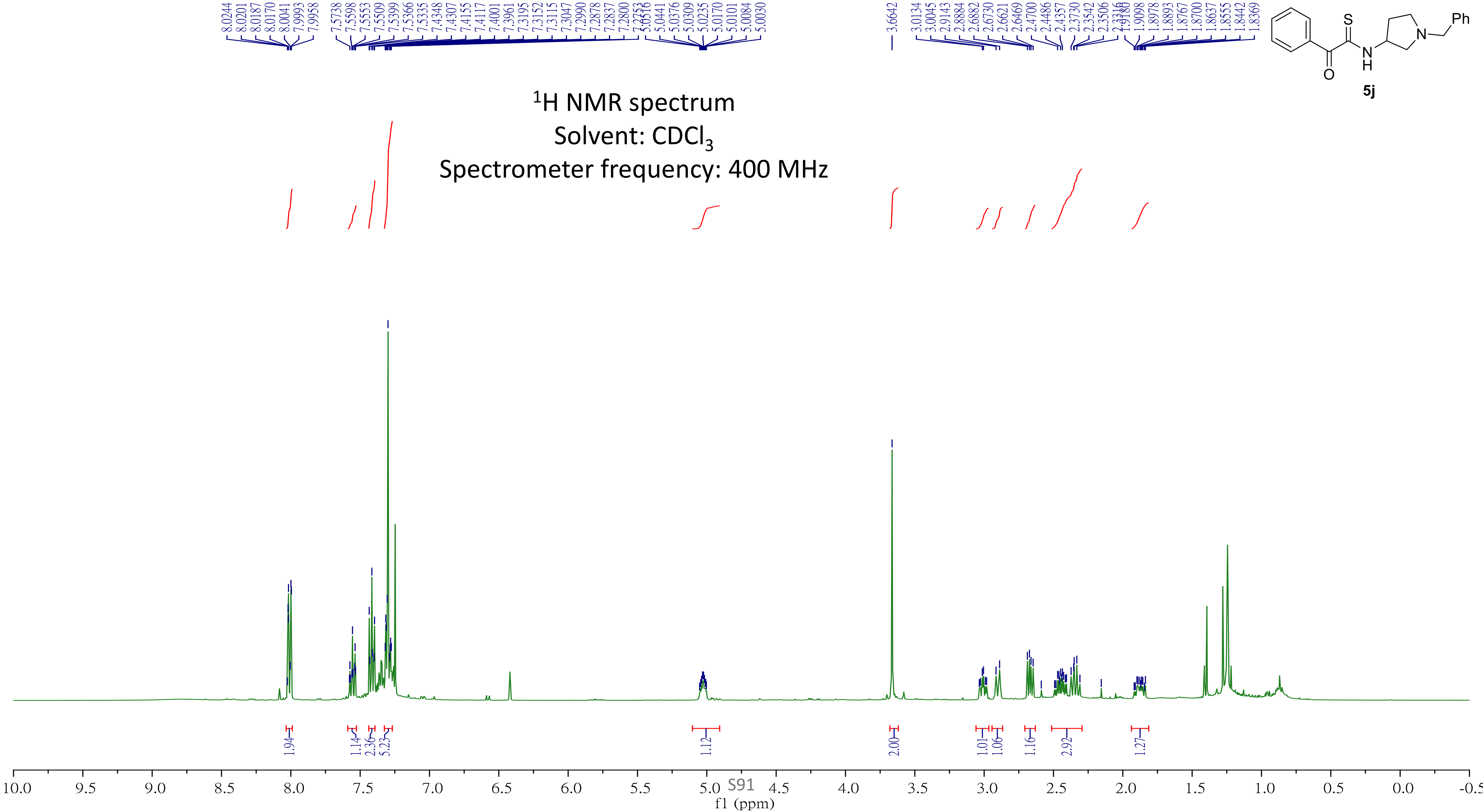
8.0244 8.0201 8.0187 8.0170 8.0041 7.9993 7.9958
7.5738 7.5598 7.5553 7.5509 7.5399 7.5366 7.5335 7.4348 7.4307 7.4155 7.4117 7.4001 7.3961 7.3195 7.3152 7.3115 7.3047 7.2990 7.2878 7.2837 7.2800 7.2753
5.0516 5.0441 5.0376 5.0309 5.0235 5.0170 5.0101 5.0084 5.0030
3.6642
3.0134 3.0045 2.9143 2.8884 2.6882 2.6730 2.6621 2.6469 2.4700 2.4486 2.4357 2.3730 2.3542 2.3506 2.3316
1.9180 1.9098 1.8978 1.8893 1.8767 1.8700 1.8637 1.8555 1.8442 1.8369
S
N
H
O
N
Ph
5j
^{1}H NMR spectrum
Solvent: $CDCl_3$
Spectrometer frequency: 400 MHz
1.94
1.14
2.36
5.23
1.12
2.00
1.01
1.06
1.16
2.92
1.27
10.0 9.5 9.0 8.5 8.0 7.5 7.0 6.5 6.0 5.5 5.0 4.5 4.0 3.5 3.0 2.5 2.0 1.5 1.0 0.5 0.0 -0.5
f1 (ppm)

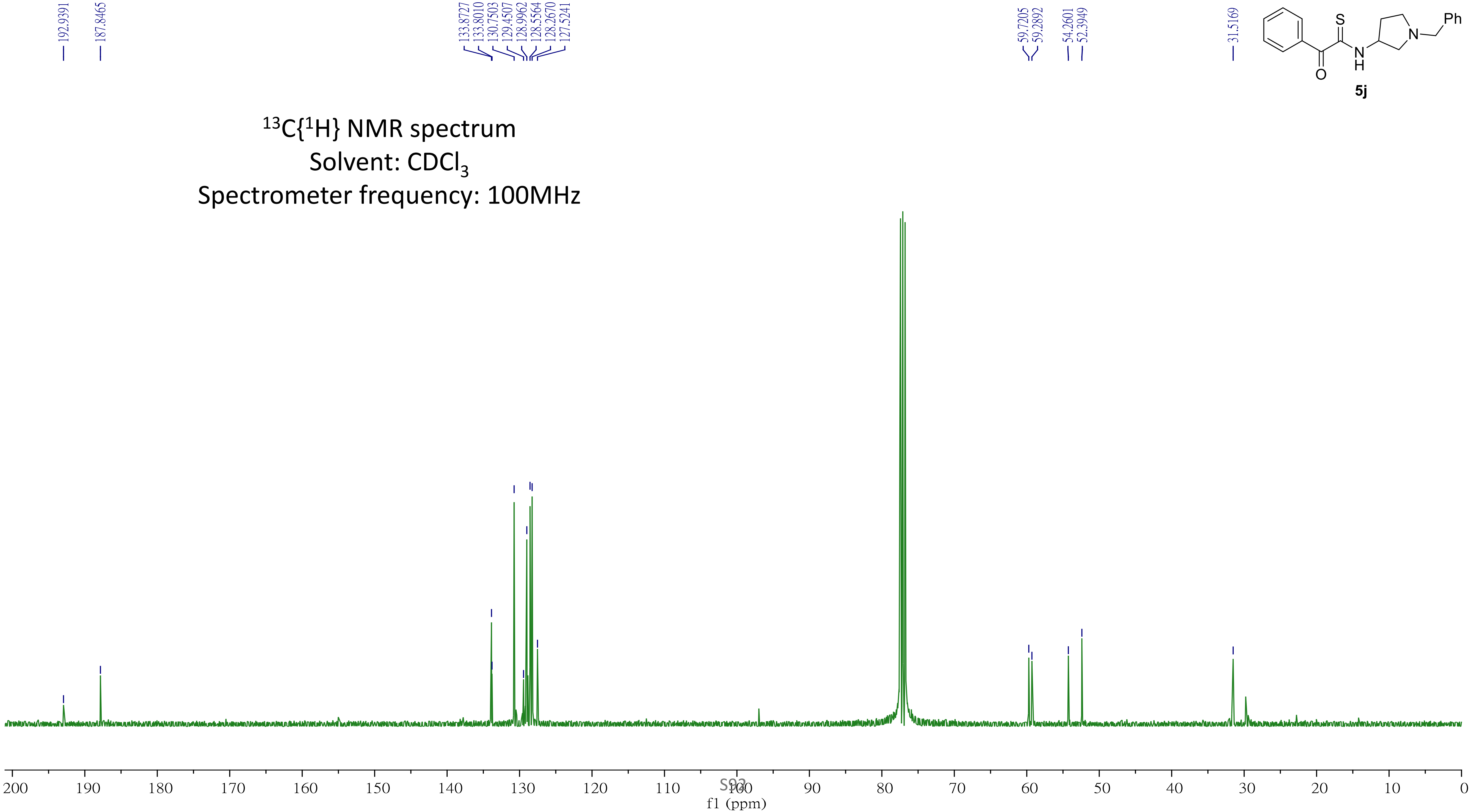
$^{13}C\{^1H\}$ NMR spectrum
Solvent: $CDCl_3$
Spectrometer frequency: 100MHz
192.9391
187.8465
133.8727
133.8010
130.7503
129.4507
128.9962
128.5564
128.2670
127.5241
59.7205
59.2892
54.2601
52.3949
31.5169
200 190 180 170 160 150 140 130 120 110 100 90 80 70 60 50 40 30 20 10 0
f1 (ppm)
S
O
N
H
N
Ph
5j

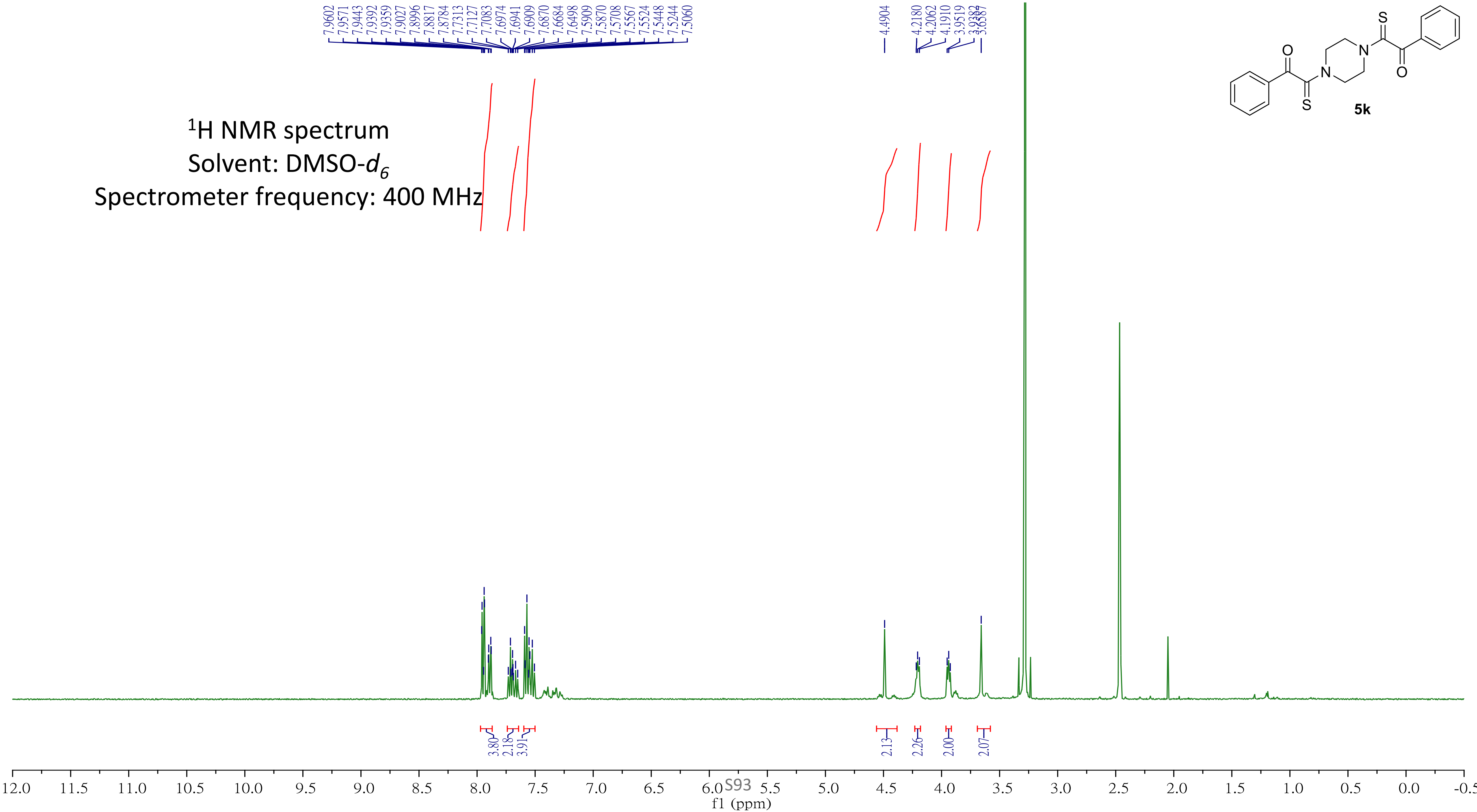

^{1}H NMR spectrum
Solvent: DMSO-d_6
Spectrometer frequency: 400 MHz
5k
7.9602
7.9571
7.9443
7.9392
7.9359
7.9027
7.8996
7.8817
7.8784
7.7313
7.7127
7.7083
7.6974
7.6941
7.6909
7.6870
7.6684
7.6498
7.5909
7.5870
7.5708
7.5567
7.5524
7.5448
7.5244
7.5060
4.4904
4.2180
4.2062
4.1910
3.9519
3.80
2.18
3.91
2.13
2.26
2.00
2.07
f1 (ppm)

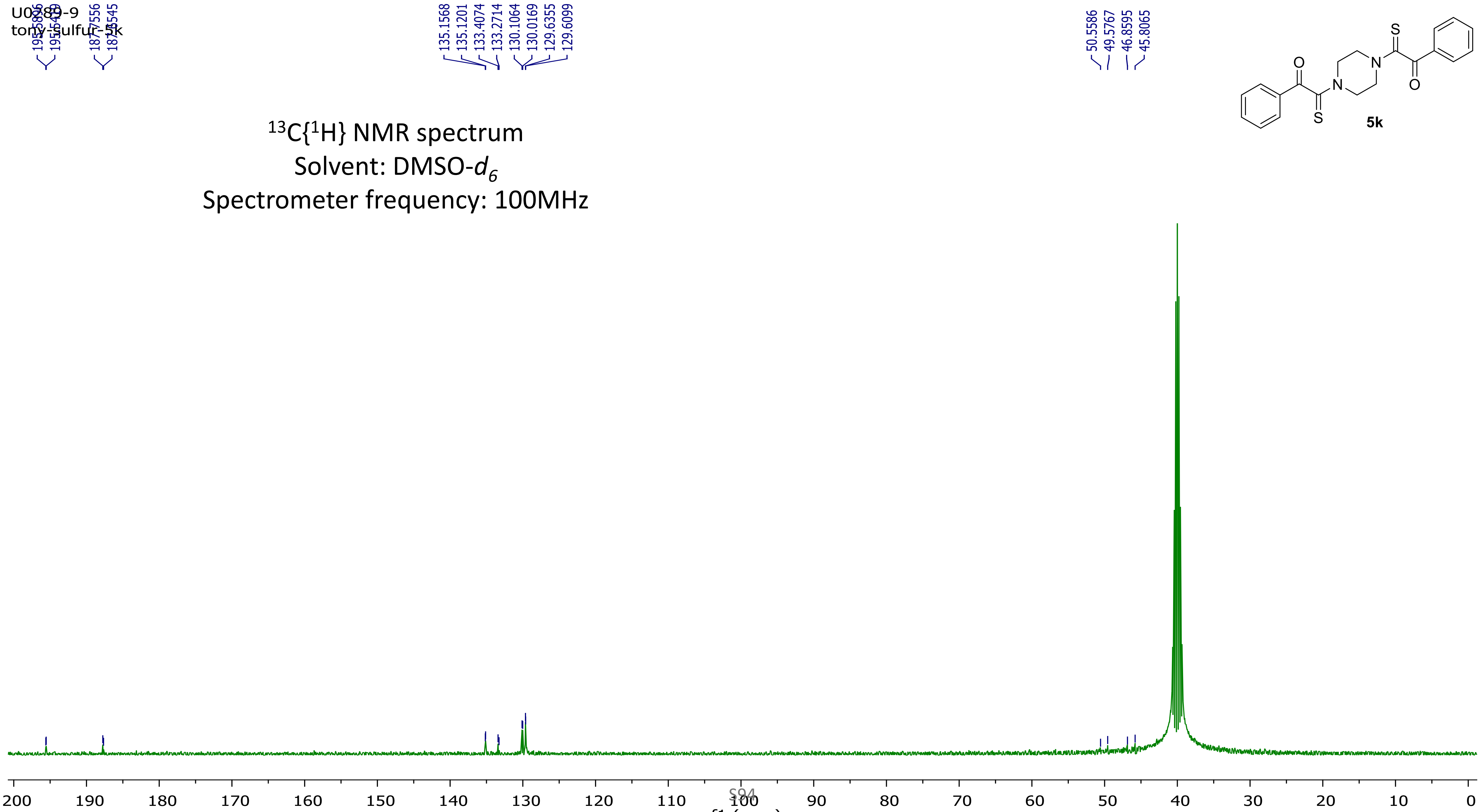
U0789-9
tony-sulfur-5k
187.7556
135.1568
135.1201
133.4074
133.2714
130.1064
130.0169
129.6355
129.6099
50.5586
49.5767
46.8595
45.8065
$^{13}C\{^1H\}$ NMR spectrum
Solvent: DMSO-d_6
Spectrometer frequency: 100MHz
5k
f1 (ppm)

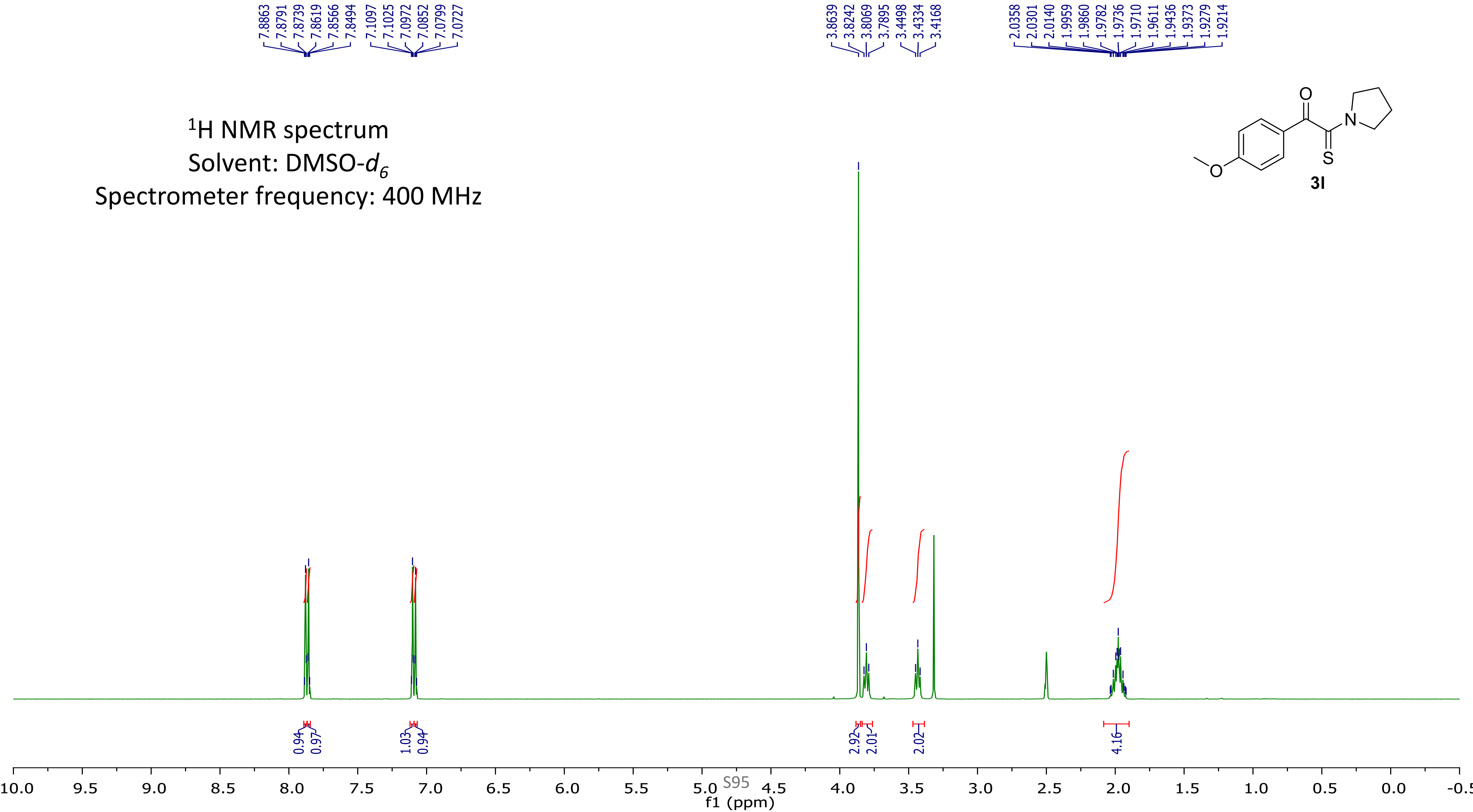

^{1}H NMR spectrum
Solvent: DMSO-d_6
Spectrometer frequency: 400 MHz
7.8863
7.8791
7.8739
7.8619
7.8566
7.8494
7.1097
7.1025
7.0972
7.0852
7.0799
7.0727
3.8639
3.8242
3.8069
3.7895
3.4498
3.4334
3.4168
2.0358
2.0301
2.0140
1.9959
1.9860
1.9782
1.9736
1.9710
1.9611
1.9436
1.9373
1.9279
1.9214
0.94
0.97
1.03
0.94
2.92
2.01
2.02
4.16
f1 (ppm)
O
N
S
O

3l

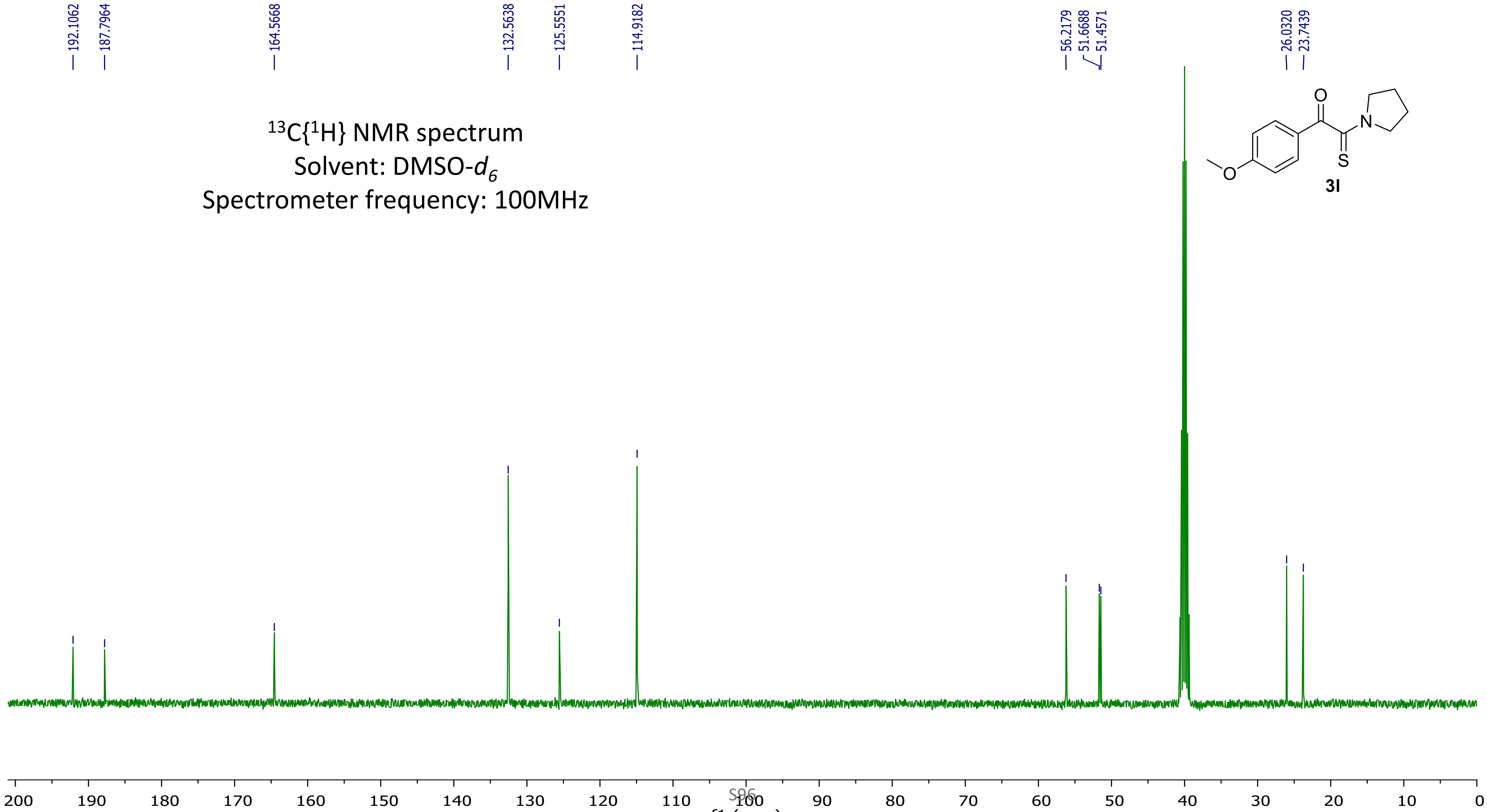
192.1062
187.7964
164.5668
132.5638
125.5551
114.9182
56.2179
51.6688
51.4571
26.0320
23.7439
$^{13}C\{^{1}H\}$ NMR spectrum
Solvent: DMSO-d_6
Spectrometer frequency: 100MHz
O
N
S
O
3l
200
190
180
170
160
150
140
130
120
110
100
90
80
70
60
50
40
30
20
10
0
f1 (ppm)

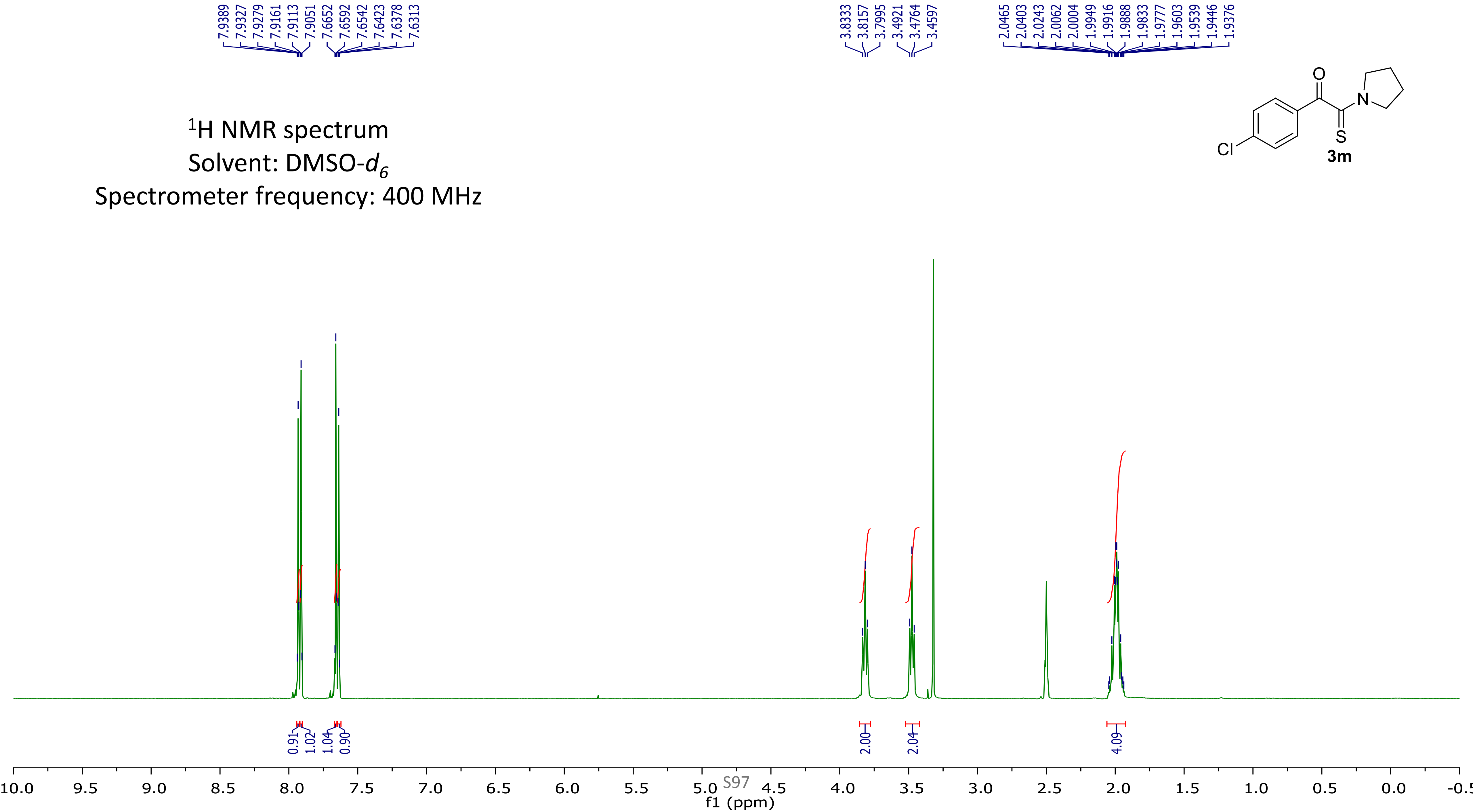

^{1}H NMR spectrum
Solvent: DMSO-d_6
Spectrometer frequency: 400 MHz
3m
7.9389 7.9327 7.9279 7.9161 7.9113 7.9051 7.6652 7.6592 7.6542 7.6423 7.6378 7.6313
3.8333 3.8157 3.7995 3.4921 3.4764 3.4597
2.0465 2.0403 2.0243 2.0062 2.0004 1.9949 1.9916 1.9888 1.9833 1.9777 1.9603 1.9539 1.9446 1.9376
0.91 1.02 1.04 0.90 2.00 2.04 4.09
10.0 9.5 9.0 8.5 8.0 7.5 7.0 6.5 6.0 5.5 5.0 4.5 4.0 3.5 3.0 2.5 2.0 1.5 1.0 0.5 0.0 -0.5
f1 (ppm)

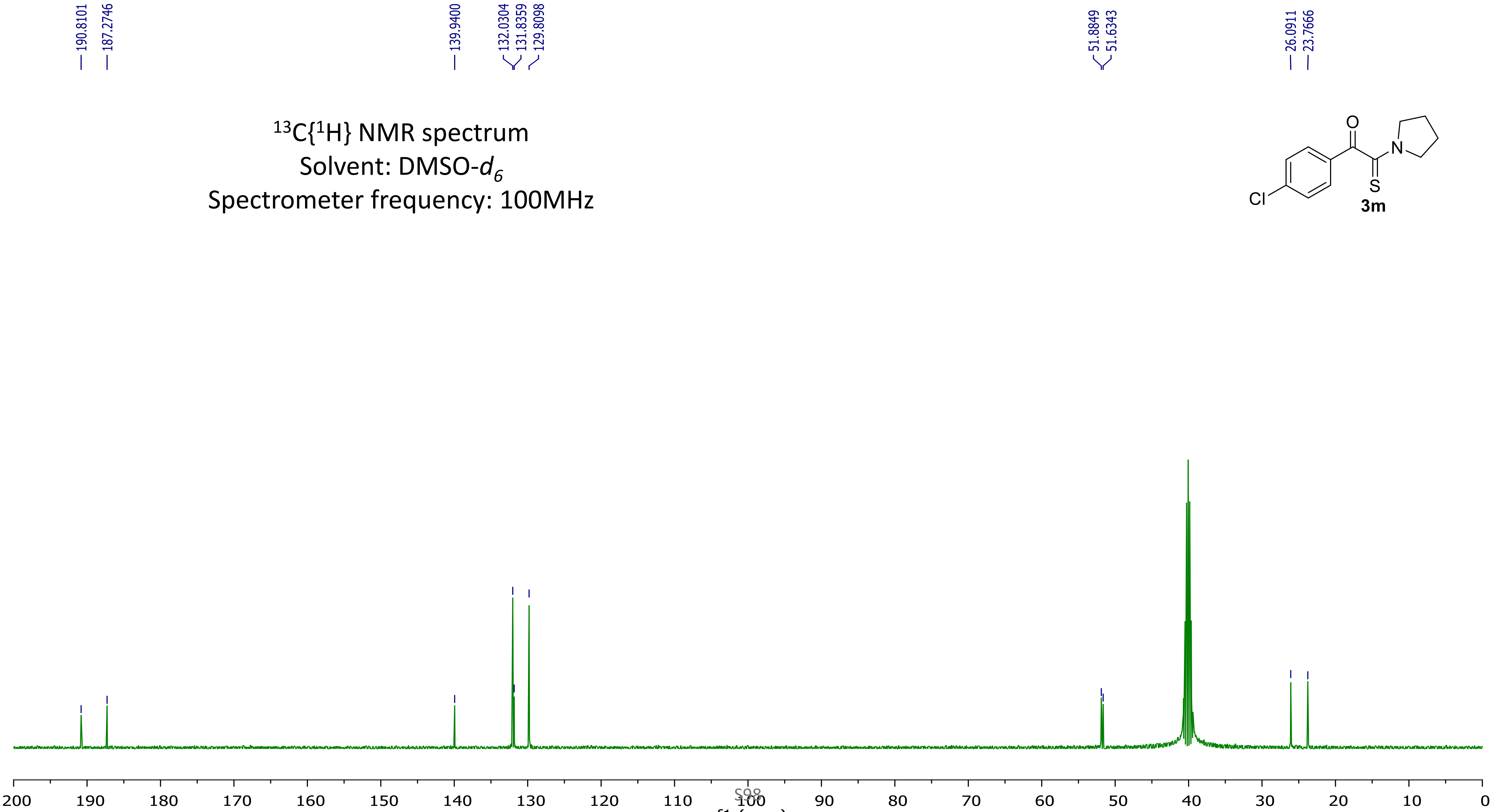
190.8101
187.2746
139.9400
132.0304
131.8359
129.8098
51.8849
51.6343
26.0911
23.7666
$^{13}C\{^1H\}$ NMR spectrum
Solvent: DMSO-d_6
Spectrometer frequency: 100MHz
O
N
S
Cl
3m
200 190 180 170 160 150 140 130 120 110 100 90 80 70 60 50 40 30 20 10 0
f1 (ppm)
